# Full mitochondrial and nuclear genome comparison confirms that *Onchocerca* sp. “Siisa” is *Onchocerca ochengi*

**DOI:** 10.1007/s00436-018-5783-0

**Published:** 2018-02-05

**Authors:** Tegegn G. Jaleta, Christian Rödelsperger, Babette Abanda, Albert Eisenbarth, Mbunkah D. Achukwi, Alfons Renz, Adrian Streit

**Affiliations:** 1 Department for Evolutionary Biology, Max Planck Institute for Developmental Biology, Max-Planck-Ring 9, 72076 Tübingen, Germany; 2 Present Address: Department of Pathobiology, School of Veterinary Medicine, University of Pennsylvania, 3800 Spruce Street, Philadelphia, PA 19104 USA; 3 Department of Comparative Zoology, Institute of Evolution and Ecology, University of Tübingen, Auf der Morgenstelle 28, 72076 Tübingen, Germany; 4 Programme Onchocercoses Field Station of the University of Tübingen, BP 65, Ngaoundéré, Cameroon; 5 Trypanosomosis Onchocerciasis Zoonoses Association for Research & Development, P.O. Box 59, Bambili-Tubah, Bamenda, Cameroon

**Keywords:** *Onchocerca ochengi*, *Onchocerca* sp. “Siisa”, Mitochondrial genome, Filarial nematode

## Abstract

*Onchocerca ochengi* is a nodule-forming filarial nematode parasite of cattle. It is the closest known relative of the human parasite *Onchocerca volvulus*, with which it shares the black fly vector *Simulium damnosum*. *Onchocerca* sp. “Siisa” was described in black flies and in cattle and, based on limited mitochondrial sequence information, appeared to be about equally phylogenetically distant from *O. ochengi* and *O. volvulus*. Based on molecular genetic markers and apparent interbreeding, we later proposed that *O.* sp. “Siisa” belongs to the species *O. ochengi*. However, we did not demonstrate directly that the hybrids were fertile, and we were still unable to resolve the phylogenetic relationship of *O. ochengi*, *O.* sp. “Siisa,” and *O. volvulus*, leaving some concerns with the conclusion mentioned above. Here, we present fully assembled, manually curated mitochondrial genomes of *O. ochengi* and *O.* sp. “Siisa,” and we compare multiple individuals of these two taxa with respect to their whole mitochondrial and nuclear genomes. Based on the mitochondrial genomes, *O. ochengi* and *O.* sp. “Siisa” are phylogenetically much closer to each other than to *O. volvulus*. The differences between them are well within the range of what is expected for within-species variation. The nuclear genome comparison provided no indication of genetic separation of *O. ochengi* and *O.* sp. “Siisa.” From this, in combination with the earlier literature, we conclude that *O. ochengi* and *O.* sp. “Siisa” should be considered one species.

**Electronic supplementary material:**

The online version of this article (10.1007/s00436-018-5783-0) contains supplementary material, which is available to authorized users.

## Introduction

The filarial nematode genus *Onchocerca* consists of about 30 described species that parasitize predominantly ungulates, but there are also species living in other hosts (Krueger et al. 2007; McFrederick et al. 2013). *Onchocerca ochengi* is a nodule-forming filarial nematode parasite of cattle and the closest known relative of *Onchocerca volvulus*, the causative agent of human onchocerciasis (Trees et al. 2000). It was hypothesized that *O. ochengi* and *O. volvulus* arose from a common ancestor as recently as 10,000 years ago, probably upon domestication of cattle (Krueger et al. 2007). Both *O. ochengi* and *O. volvulus* share the same black fly vector *Simulium damnosum* and they are very similar with respect to their biology and at the molecular and the antigenic levels. *O. ochengi* has therefore been established as an attractive animal model to study aspects of onchocerciasis (Makepeace and Tanya 2016; Trees et al. 2000). It must be noted, however, that unlike *O. volvulus*, *O. ochengi* does not cause any obvious pathology, probably because the co-existence with its host is much older and, as a consequence, more benign (Trees et al. 2000). Although not formally published, *O. ochengi* draft genomes were generated by the Blaxter laboratory, University of Edinburgh and the Welcome Trust Sanger Institute and are publicly available through http://www.nematodes.org/genomes/onchocerca_ochengi/, WormBase ParaSite (https://parasite.wormbase.org/index.html) and under accession numbers PRJEB1204 and PRJEB1809.

Krueger et al. (2007) isolated *Onchocerca* larvae from black flies in Uganda, which based on selected mitochondrial (nicotinamide adenosine dinucleotide dehydrogenase subunit 5 [*nad* 5], 12S rDNA, and 16S rDNA) and nuclear (5S rDNA intergenic spacer and O-150 tandem repeat) sequences, appeared to assume an “intermediate” phylogenetic position between *O. ochengi* and *O. volvulus*. These authors referred to these worms as *Onchocerca* sp. “Siisa,” leaving the exact taxonomic status to be determined by future studies. Later, based on partial sequences of the mitochondrial 12S, 16S, and cytochrome oxidase subunit 1 (*cox*1) genes, we showed that *Onchocerca* sp. “Siisa” was also present in black flies in Cameroon and that cattle are the, or one of the, definitive hosts of *Onchocerca* sp. “Siisa” (Eisenbarth et al. 2013). Later, we concluded that *Onchocerca* sp. “Siisa” belongs to the species *O. ochengi* and is one of two major mitochondrial clades of this species present in Cameroon (we referred to the two clades as *O. ochengi* variant ochengi and *O. ochengi* variant Siisa) (Hildebrandt et al. 2014). This claim was based on the following observations. (1) We found no morphological difference among the larvae (Eisenbarth et al. 2013), adults, or nodules (Hildebrandt et al. 2014) between the two types (notice, however, that in one later study (Eisenbarth et al. 2016), the small number of *O.* sp. “Siisa” found appeared marginally but weakly significantly shorter than the *O. ochengi* from the same sampling sites). (2) Both variants co-occur in the same intermediate (Eisenbarth et al. 2013) and definitive host individuals (Eisenbarth et al. 2013; Hildebrandt et al. 2014). (3) Within a definitive host individual carrying both types, we failed to detect any assorted mating (Hildebrandt et al. 2014). (4) Based on the limited nuclear sequence information, there appeared to be no genetic differentiation of the nuclear genomes between the two mitochondrial clades (Hildebrandt et al. 2014). (5) Based on the analysis of a limited number of single copy nuclear loci of individual adult males and females and of individual microfilariae, we found that variant-mixed pairs readily produced progeny, the fertility of which was, however, not demonstrated directly (Hildebrandt et al. 2014).

Furthermore, like Krueger et al. (2007), we were unable to resolve the mitochondrial phylogenetic relationships of *O. volvulus*, *O. ochengi*, and *Onchocerca* sp. “Siisa,” and *O. ochengi* and *Onchocerca* sp. “Siisa” appeared about equally distant from each other as either one of them from *O. volvulus* (Eisenbarth et al. 2013; Hildebrandt et al. 2014). *O. ochengi* and *O. volvulus*, in turn, are well-established as being separate species, even having different numbers of chromosomes (Doyle et al. 2016; Post 2005).

To clarify this issue, we decided to compare the entire mitochondrial and nuclear genomes of the two taxa. We present here two complete, manually finished and annotated mitochondrial genomes derived from one individual of each of *O. ochengi* and *Onchocerca* sp. “Siisa,” and we compare the whole mitochondrial genome sequences based on short read sequencing of an additional nine individuals representing both variants with these sequences. We show that (1) the separation into two mitochondrial clades is reflected throughout the genome and (2) that when considering the entire mitochondrial genomes, *O. ochengi* and *Onchocerca* sp. “Siisa” are clearly more similar to each other than either of them is to *O. volvulus* and the difference between the two is well within the range expected for within-species variability. Further, based on whole nuclear genome data, we show that there is no indication of nuclear genetic differentiation between the two mitochondrial clades, strongly suggesting that they interbreed and do not represent reproductively isolated populations.

## Materials and methods

### Isolation of adult *Onchocerca* sp. worms from skin nodules

The skin nodules containing adult *Onchocerca* worms were collected in the Ngaoundéré abattoir, Adamawa Region, Cameroon, as described (Wahl et al. 1994) between October 2013 and March 2014. The nodules were stored in 80% ethanol and shipped to Max Planck Institute (MPI) for Developmental Biology in Tübingen, Germany, for analyses.

### DNA extractions, PCR, and library preparation

Adult worms were isolated from the nodule tissue by collagenase digestion as described (Kläger et al. 1996). Briefly, nodules were incubated at 37 °C overnight in 0.2% collagenase with phosphate buffered saline (PBS) solution and then washed several times with PBS. DNA was extracted from single worms using the Epicenter DNA extraction kit (Epicenter, USA) according to the manufacturer’s instructions. The DNA was quantified using Qubit fluorimeter measurement (Invitrogen Life Technologies, USA). Before library preparation, partial *cox1*, *12S*, and *16S* were PCR amplified using the primer pairs and conditions reported earlier (Eisenbarth et al. 2013) and sequenced using one of the PCR primers. The sequencing reactions were done using the BigDye® Terminator v3.1 Cycle Sequencing Kit (Applied Biosystems) according to the manufacturer’s protocol, and the reactions were submitted to the in-house sequencing facility at the MPI for Developmental Biology at Tübingen for electrophoresis and base calling. The partial *cox1*, *12S*, and *16S* were compared with the nucleotide database entries using BLAST at the National Center for Biotechnology Information (NCBI) to determine if the worms were *O. ochengi* and/or *O.* sp. “Siisa”. DNA libraries were prepared from 50 ng of genomic DNA using the Low Input DNA library preparation kit (Rubicon Genomics, USA) according to the manufacturer’s instructions. The libraries were quantified using Qubit and Bioanalyzer (Agilent Technologies, USA) and then normalized to 2.5 nM. The samples were sequenced as 400 bp paired ends in one multiplexed lane using HiSeq2000 platform (Illumina Inc., USA) at MPI for Developmental Biology in-house genome facility.

### Assembly and analysis of the *O. ochengi* and the *O.* sp. “Siisa” reference mt genomes

For the two samples M1 (male *O*. sp. “Siisa”) and M3 (male *O. ochengi*), the mitochondrial DNA (mtDNA) sequences were extracted from the whole genome sequencing data (Jex et al. 2010). The extracted sequences were assembled manually and aligned to the complete mitochondrial genome sequence of *O. volvulus* (AF015193) (Keddie et al. 1998) using MUSCLE (Edgar 2004) and assembled using SeqBuilder (DNASTAR, Inc). Regions that could not be unambiguously assembled based on the short-read sequences were PCR amplified and sequenced by conventional Sanger sequencing as described above. The assembled mitochondrial genomes were aligned using MUSCLE (Edgar 2004). The protein-coding and rRNA genes of the *O. ochengi* mtDNA were annotated based on the published *O. volvulus* mitochondrial genes (Keddie et al. 1998). The codon usages in the 12 protein-coding genes were examined using the invertebrate mitochondrial genetic code as a reference, and the amino acid frequencies were compared with the nucleotide composition of the respective codon families (Singer and Hickey 2000). The transfer RNA (tRNA) genes were identified by ARWEN v1.2 (Laslett and Canback 2008) using the metazoan mitochondrial tRNA data set as source. Percent identities, sequence length, and transition to transversion ratio of nucleotide substitutions for these two isolates were calculated using MEGA6 (Tamura et al. 2013).

### Analysis of whole genome sequencing data

Raw reads were aligned to the *O. ochengi* reference genome (http://www.nematodes.org/genomes/onchocerca_ochengi/) and variants were called as described in Rödelsperger et al. (2014). While all three male samples showed at least 90-fold genome-wide coverage, only very few reads of the female samples could be aligned to the reference genome. Further investigation of a subset of unaligned reads by BLASTN searches against the NCBI nucleotide data base revealed large-scale contamination with bovine sequences. For this reason, only data for the highly covered mitochondrial genome was suited for further analysis of female samples.

To screen for evidence for recent admixture between the three male samples, we visualized the genotypes of variable sites and the frequencies of variants in 10-kb windows along selected contigs. The raw reads were deposited at the European Nucleotide Archive under the study accession PRJEB23566.

### Analysis of sequence polymorphisms

For all samples, the mtDNA sequence was extracted from the whole genome sequences (Jex et al. 2010). The nucleotide sequences to be compared (either the entire mitochondrial genome or only the protein-coding sequences, as specified in the text) were aligned using the MUSCLE algorithm (Edgar 2004). The nucleotide alignment was checked for translational reading frame shifts by translation and then visual inspection. Pairwise nucleotide diversity was calculated between the isolates using MEGA6 (Tamura et al. 2013). For some analyses, as specified in the text and the tables, *O. volvulus* (AF015193) and *Onchocerca flexuosa* (HQ214004) were included for comparison.

### Phylogenetic analyses

For the phylogenetic analysis, we considered the mitochondrial ribosomal DNA sequences plus the nucleotide sequences of the 11 of the 12 mt protein-coding genes that were available for all samples considered (nad4L is not included). In addition to the 11 new samples from this study, we also included *O. volvulus* (AF015193), *O. flexuosa* (HQ214004), *Onchocerca gutturosa* (PRJEB7568, unpublished, provided by M. Blaxter, University of Edinburgh), and *O. ochengi* (unpublished but downloadable from http://www.nematodes.org/genomes/onchocerca_ochengi/; see also https://www.biorxiv.org/content/early/2017/12/20/236539) and *Dirofilaria immitis* (AJ537512) as outgroup. The sequences were concatenated (Suppl. file 1) and aligned using MUSCLE (Edgar 2004). The molecular phylogenetic analysis was done using MEGA6 (Tamura et al. 2013). The evolutionary history was inferred by using the maximum likelihood method based on the Tamura-Nei model (Tamura and Nei 1993). Initial trees for the heuristic search were obtained by applying the neighbor-joining method to a matrix of pairwise distances estimated using a JTT model. The final phylogenetic tree was reconstructed using the maximum likelihood method with 1000 bootstrap replications (Tamura et al. 2013).

## Results and discussion

### *O. ochengi* variant ochengi and *Onchocerca* sp. “Siisa” reference mitochondrial genomes

**Fig. 1:**
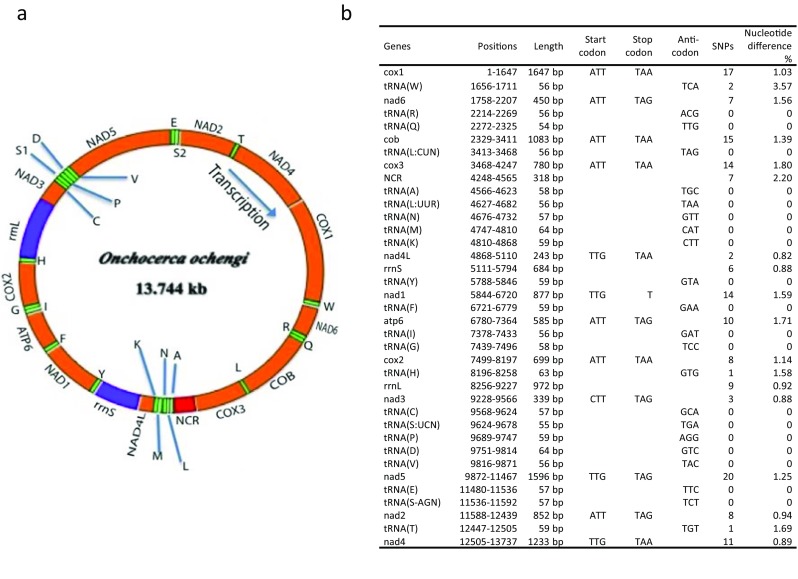
The mitochondrial genome of *O. ochengi* (KX181289). **a** Graphical representation. All genes are transcribed clockwise. Protein-coding and rRNA genes are indicated with the standard nomenclature. The tRNA genes are indicated with the one-letter code of their corresponding amino acids. There are two tRNA genes for leucine: L1 for codons CUN and L2 for UUR; and two tRNA genes for serine: S1 for codons UCN and S2 for AGN. “NCR” refers to the noncoding region. **b** Positions of the different genes. The first nucleotide of the start codon of the *cox*1 gene was set as one. For protein-coding genes, the initiation and the termination codons are indicated, and for tRNA genes, the anticodons are indicated. Columns SNPs and % difference refer to the comparison of the *O. ochengi* (KX181289) and the *O.* sp. “Siisa” (KX181290) genomes. Two SNPs are in small intergenic regions. Notice, five tRNA sequences overlap with the start of protein-coding genes by 1–3 nucleotides (tRNA(L: CUN)-*cox*3, tRNA(K)-*nad*4L, tRNA(Y)-*nad*1, tRNA(S:AGN)*-nad*2, tRNA(T)-*nad*4). tRNA(H) overlaps with the end *cox*2 by two nucleotides. Two tRNAs, tRNA(Y) and tRNA(H), overlap with *rrnS* and *rrnL* by 7 and 3 nucleotides, respectively. Three pairs of tRNAs share 1–7 nucleotides overlapping with each other (tRNA(L:UUR)-tRNA(N), tRNA(C)-tRNA(S:UCN), and tRNA(E)- tRNA(S:AGN))

We conducted short read whole genome sequencing experiments with 11 adult single *Onchocerca* worms (3 males, M1–M3, and 8 females, F1–F8). While the males yielded good coverage, the female samples were very heavily contaminated with bovine (host) sequences indicating that collagenase digestion did not remove all host tissue and resulting in a small proportion of *Onchocerca*-derived reads. Nevertheless, the available information was sufficient for extraction of the mitochondrial sequences. For two males, one *O. ochengi* and one *Onchocerca* sp. “Siisa” (based on their *cox*1, 12S, and 16S sequences; c.f. Eisenbarth et al. 2013), we manually assembled and annotated the full mitochondrial genomes including the AT-rich noncoding region. Regions that could not be unambiguously assembled based on the short-read data were PCR amplified and sequenced using conventional Sanger sequencing. The resulting reference genomes were deposited in GenBank under the accession numbers KX181289 (*O. ochengi*) and KX181290 (*Onchocerca* sp. “Siisa”). The *O. ochengi* mitochondrial genome is 13,744 bp long (Fig. 1). Compared to *O. volvulus* (AF015193) (Keddie et al. 1998) and *O. flexuosa* (HQ214004) (McNulty et al. 2012), the *O. ochengi* mitochondrial genome is slightly smaller due to shorter intergenic regions. The mitochondrial gene content and order (Fig. 1) is the same as in *O. volvulus* (Keddie et al. 1998) with 12 protein-coding genes, 22 tRNA genes, and coding units for the small (12S, *rrnS*) and the large (16S, *rrnL*) ribosomal subunit RNAs. Like all other nematode species whose mitochondrial genomes were sequenced (except for *Trichinella spiralis*; Lavrov and Brown 2001), *O. ochengi* lacks the protein-coding gene *atp*8. The AT-rich (83.02%) noncoding region is 318 bp and is located between the *cox*3 and the tRNA(A) genes*.* The 19 short intergenic regions vary in length from 1 to 46 bp.

The mitochondrial genome of *O. ochengi* is very A-T rich (overall 73.22%) and there is a bias for the nucleotide T being on the coding strand. Of G-C base pairs, the G is preferentially on the coding strand. The nucleotide composition of the coding strand is as follows: A = 2607 (18.97%), T = 7456 (54.25%), G = 2765 (20.12%), and C = 916 (6.66%). All codons are used except for Ala (GCC), Pro (CCC), Ser (TCC), and Thr (ACC). Codons composed of A and T nucleotides are predominantly used, reflecting the very strong bias toward A+T in the mitochondrial genome of *O. ochengi* (Suppl. Tab. 1)*.*

The *Onchocerca* sp. “Siisa” mitochondrial genome (KX181290) is identical with KX181289 in length and structure, but the two reference genomes differ at 157 single nucleotide positions (1.15% difference). Differences are present in all protein-coding and rRNA genes, in three of the tRNA genes, and in intergenic regions (Fig. 1b).

### Comparison of the mitochondrial protein-encoding genes of additional individuals and species

Next, we conducted pairwise nucleotide sequence comparisons of the protein-coding sequences (in total 10,407 bp) between the 11 *Onchocerca* individuals from this study and the published sequences of *O. volvulus* (AF015193) (Keddie et al. 1998) and *O. flexuosa* (HQ214004) (McNulty et al. 2012) (Table 1). This comparison was limited to protein-coding genes because they could be unambiguously aligned for all samples included. The pairwise nucleotide diversities among the *Onchocerca* individuals isolated from cattle for this study fell into two nonoverlapping groups ranging from 0.029 to 0.211% and from 1.24 to 1.36%. These two groups confirm the existence of the two mitochondrial clades within our sample and represent within- and between-clade comparisons, respectively. By virtue of mitochondrial sequence, we consider male 1 and female 5 *Onchocerca* sp. “Siisa” and all the others *O. ochengi*.

**Table 1:** Pairwise nucleotide diversity in protein-coding mitochondrial genes

	Sample.	1	2	3	4	5	6	7	8	9	10	11	12
1	F1												
2	F2	0.096											
3	F3	0.048	0.048										
4	F4	0.096	0.048	0.076									
5	F5	1.29	1.27	1.24	1.29								
6	F6	0.086	0.067	0.057	0.076	1.28							
7	F7	0.067	0.086	0.038	0.096	1.28	0.076						
8	F8	0.105	0.029	0.057	0.057	1.28	0.076	0.096					
9	M1	1.28	1.26	1.25	1.27	0.144	1.25	1.27	1.27				
10	M2	0.211	0.182	0.172	0.190	1.36	0.172	0.192	0.190	1.34			
11	M3	0.086	0.029	0.057	0.038	1.28	0.096	0.076	0.038	1.25	0.173		
12	*O. volvulus*	3.29	3.28	3.30	3.30	3.36	3.28	3.32	3.28	3.35	3.37	3.29	
13	*O. flexuosa*	10.28	10.26	10.27	10.28	10.27	10.28	10.29	10.26	10.29	10.30	10.30	10.40

Numbers 1–11 are the individuals isolated for this study (*F* female, *M* male). Number 12 is *O. volvulus* (AF015193). Number 13 is *O. flexuosa* (HQ214004). The numbers are pairwise nucleotide differences in percent. For values < 1, three decimal positions, and for values > 1, two decimal positions are listed. M1 and M3 are the individuals the reference sequences were derived from

The pairwise nucleotide diversities between *O. volvulus* and either *O. ochengi* or *Onchocerca* sp. “Siisa” were very similar to each other and clearly larger than between the latter two (the largest difference between *O. ochengi* and *Onchocerca* sp. “Siisa” individuals was 1.36%, while the smallest difference between *O. volvulus* and any *O. ochengi* or *Onchocerca* sp. “Siisa” individual was 3.28%)*. O. flexuosa* with differences to *O. ochengi*, *Onchocerca* sp. “Siisa,” and *O. volvulus* of around 10.3% is clearly more phylogenetically distant.

**Fig. 2:**
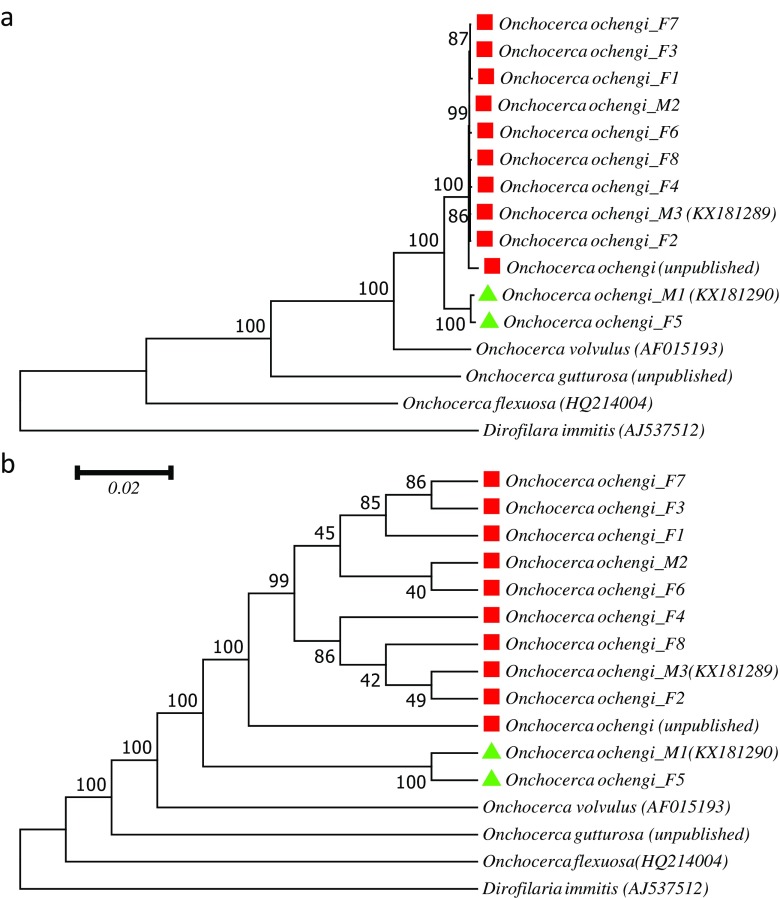
Maximum likelihood reconstruction of a phylogenetic tree based on the concatenated nucleotide sequences of 11 protein-coding mitochondrial genes and the ribosomal rDNA sequences (Suppl. file 1). In total 11,701 positions were considered, and gaps and missing data were eliminated. Included are the same *Onchocerca* samples as in Table 1 plus the unpublished *Onchocerca ochengi* sequence downloaded from http://www.nematodes.org/genomes/onchocerca_ochengi/ and *D. immitis* as outgroup. Sequence accession numbers are given in (). The raw data for F1–F8 and M2 are available under the accession number PRJEB23566. The percentage of trees among 1000 bootstrap repetitions in which the associated taxa clustered together is shown next to the branches. If the ribosomal sequences, which had been described to show a high degree of intraspecific variation in nematodes (Hu and Gasser 2006) were excluded, the topology of the tree did not change, as far as nodes with high bootstrap support are concerned (data not shown). **a** The tree is drawn to scale, with branch lengths measured in the number of substitutions per site. **b** The same tree as in **a** but displayed as cladogram in order to better show the topology and bootstrap values

To further evaluate the phylogenetic relationship of *O. ochengi*, *Onchocerca* sp. “Siisa,” and *O. volvulus*, we reconstructed a maximum likelihood (ML) phylogenetic tree (Fig. 2). We included the same samples as in the nucleotide comparison (Table 1) plus the *O. ochengi* mitochondrial genome sequence available from the Blaxter laboratory (http://www.nematodes.org/genomes/onchocerca_ochengi/; see also https://www.biorxiv.org/content/early/2017/12/20/236539), for which also a nuclear genome is available, and the sequence of *O. gutturosa* (PRJEB7568, unpublished, provided by M. Blaxter, University of Edinburgh) plus *D. immitis* (AJ537512) as outgroup sequences. The phylogenetic tree is based on the 11 mitochondrial protein-coding genes for which the sequence was available for all samples included and the ribosomal sequences (Suppl. file 1). This analysis confirms the existence of two separate groups corresponding to *O. ochengi*, and *Onchocerca* sp. “Siisa”, which, however, clearly group together in comparison with *O. volvulus*.

Taken together, our phylogenetic analyses based on the entire mitochondrial genomes clearly show that *Onchocerca* sp. “Siisa” (Krueger et al. 2007) is more closely related to *O. ochengi* than to *O. volvulus* and does not assume an “intermediate” position between these two taxons or even group with *O. volvulus* as it may have appeared based on the limited sequence information considered in earlier studies (Eisenbarth et al. 2013; Krueger et al. 2007).

It was found that the mitochondrial pairwise nucleotide differences between individuals of closely related nematode species (species within the same genus) are typically around 10 to 20% while differences within a species average below 1% and surpass 2% only in exceptional cases, which can go up to 6% (Blouin 2002). The differences we observed between *O. ochengi* and *Onchocerca* sp. “Siisa” are therefore well within the range expected for within-species variation.

### Comparison of the nuclear genomes of representatives of the mitochondrial variants “ochengi” and “Siisa”

The mitochondrial genome is usually only maternally inherited and does not undergo meiotic recombination (Mishra and Chan 2014; Sato and Sato 2011). Therefore, the sequence comparison between the mitochondrial genomes of two individuals provides information about the time elapsed since the last common female ancestor to which both individuals are connected entirely through females but it does not reflect if the two matrilineages did or still do interbreed and therefore belong to the same species or not. We therefore compared the nuclear genomes of the three males, which had good sequence coverage.

**Fig. 3:**
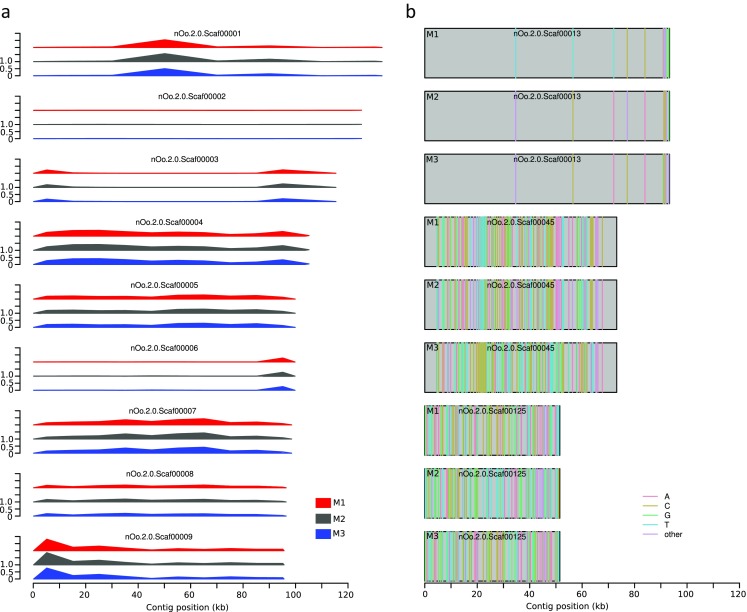
Analysis of the nuclear genome. **a** Variant (from the reference genome) frequency (%) is shown in nonoverlapping 10-kb window across the nine largest *O. ochengi* contigs. **b** The rectangles represent three selected contigs and their genotypes at variable positions in all three male samples. The nucleotides at polymorphic positions are color coded; “other” indicates small insertion/deletions or heterozygous positions. All three contigs show different phylogenetic relationships between the samples indicating toward recent admixture

The frequency of variants ranged between 0.001 and 0.009 per site for the nine largest contigs (Fig. 3a). With regard to the *O. ochengi* reference genome, all three individuals showed varying but correlated distance profiles, suggesting that the three males isolated at the same time and place for our study are more similar to each other than either one of them is to the reference individual, which is of mitochondrial type ochengi (Fig. 2) but was collected at a different time and place, however, also in Cameroon. Hence, the degree of mitochondrial sequence variability is not predictive for the extent of nuclear sequence variability. To further screen for evidence of recent admixture between isolates that were classified as “Siisa” and “ochengi,” we searched for genomic regions that would give rise to different phylogenetic relationships between the isolates (Fig. 3b). While the genotypes on contig nOo.2.0.Scaf00013 clearly separates the Siisa male M1 from the ochengi males M2 and M3, two other contigs show different genealogies indicating toward recent recombination between the genetic lineages.

## Conclusions

Of course, we cannot exclude that somewhere in Africa a very recent speciation event occurred and the mitochondrial sequences of this newly formed species is more similar to one of the two mitochondrial clades of *O. ochengi* described here than to the other. However, for the moment, taken all our results together and also considering the results of Hildebrandt et al. (2014), at least for our study area, there is no indication for any form of reproductive isolation between what was described as *O. ochengi* and as *Onchocerca* sp. “Siisa” based on mitochondrial DNA sequence. Therefore, there is no reason to postulate that they represent different subpopulations or even species, and therefore, *Onchocerca* sp. “Siisa” should be considered *O. ochengi*. It is interesting that, so far, all but one individual *O. ochengi* we isolated in Northern Cameroon and genotyped for three previous studies (Eisenbarth et al. 2013, 2016; Hildebrandt et al. 2014) and for this study could be clearly assigned to one of the two mitochondrial clades described. In total, these were 472/473 individuals, isolated as adults from nodules or as larvae from black flies (microfilariae that might have been the progeny of already counted adults are not included in this number). This result indicates that ultimately almost the entire population of *O. ochengi* in this area is derived from two different females, whose progeny immigrated simultaneously or sequentially or that were the survivors of a dramatic population bottleneck. It will be interesting to see if in other parts of Africa additional mitochondrial clades exist.

## Electronic supplementary material

Suppl Table 1 Codon usage in the mitochondrial genome of *O. ochengi* (KX181289). (PDF 50 kb)

Suppl File 1 Concatenated Sequences used for the phylogenetic analysis. (PDF 158 kb)
