# Supplementary material for: Full mitochondrial and nuclear genome comparison confirms that Onchocerca sp. “Siisa” is Onchocerca ochengi
Source: Parasitol Res. 2018 Feb 5;117(4):1069–77. doi: 10.1007/s00436-018-5783-0 (PMC5978932; doi:10.1007/s00436-018-5783-0)
Supplement: Supplementary file 1 — Codon usage in the mitochondrial genome of O. ochengi (KX181289). (PDF 50 kb) [file 436_2018_5783_MOESM1_ESM.pdf]

Suppl. Tab 1

Codon usage in the mitochondrial genome of *O. ochengi* (KX181289)

Amino Acid	Codon	Number	Codon Frequency
Ala	GCG	6	0.173
	GCA	3	0.086
	GCT	75	2.16
Cys	TGT	98	2.82
	TGC	3	0.086
Asp	GAT	83	2.40
	GAC	3	0.086
Glu	GAG	41	1.20
	GAA	23	0.66
Phe	TTT	634	18.3
	TTC	4	0.115
Gly	GGG	34	0.98
	GGA	17	0.49
	GGT	191	5.50
	GGC	6	0.173
His	CAT	50	1.44
	CAC	3	0.086
Ile	ATT	176	5.07
	ATC	5	0.144
Lys	AAG	67	1.93
	AAA	9	0.26
Leu	TTG	311	8.96
	TTA	137	3.95
	CTG	3	0.086
	CTA	1	0.03
	CTT	23	0.66
	CTC	1	0.03
Met	ATG	111	13.71
	ATA	30	3.20
Asn	AAT	79	2.30
	AAC	4	0.115
Pro	CCG	6	0.173
	CCA	2	0.0576
	CCT	68	1.96
Gln	CAG	38	1.095
	CAA	13	0.37
Arg	CGG	10	0.228
	CGA	1	0.03
	CGT	42	1.21
	CGC	1	0.03
Ser	AGG	23	0.66
	AGA	14	0.40
	AGT	113	3.26
	AGC	4	0.11
	TCG	5	0.14
	TCA	3	0.086
	TCT	159	4.58
Thr	ACG	2	0.0576
	ACA	2	0.0576
	ACT	83	2.40
Val	GTG	44	1.30
	GTA	24	0.69
	GTT	263	7.58
	GTC	2	0.0576
Trp	TGG	45	1.30
	TGA	31	0.89
Tyr	TAT	222	6.40
	TAC	4	0.11
Stop	TAG	6	0.176
	TAA	7	0.20
