# Supplementary material for: Full mitochondrial and nuclear genome comparison confirms that Onchocerca sp. “Siisa” is Onchocerca ochengi
Source: Parasitol Res. 2018 Feb 5;117(4):1069–77. doi: 10.1007/s00436-018-5783-0 (PMC5978932; doi:10.1007/s00436-018-5783-0)
Supplement: Supplementary file 2 — Concatenated Sequences used for the phylogenetic analysis. (PDF 158 kb) [file 436_2018_5783_MOESM2_ESM.pdf]

Supplemental File 1
Concatenated sequences used for the phylogenetic reconstruction in Figure 2.

>Dirofilaria immitis(AJ537512)
ATTTTTTGTGGAATGACTTTTGGTAATAGAATGAAACAGAGTATTTTTAGTACTGTTAATCATAAAA
CTATTGGTACTTTTTATATTGTTTTGGGTTATTGAGCAGGATTGGGAGGTTCTGTTTTGTCTATATT
GATTCGTTTTGAATTGTCTAGTCCTGGTGGTTATTTGTTTTTTGGAAGTGGTCAGGTTTATAATTCT
GTTTTAACTATGCATGGTGTTTTGATGATTTTTTTTATAGTTATGCCTATTTTGATTGGTGGTTTTG
GTAATTGGATGTTGCCTTTGATATTGGGTGCCCCTGAAATGGCTTTTCCTCGTGTTAATGCTTTATC
TTTTTGGATTACTTTTGTTGCGTTGTTGATGGTTTATCAATCTTTTTTTATTGGGGGGGTCCTGGG
AGTAGTTGAACTTTTTATCCTCCTTTGAGTGTAGAGGGTCAGCCTGAGTTATCTTTGGATAGAATGA
TTTTAGGTCTTCATACTGTTGGTATTGGTTCTTTATTAGGTGCTATTAATTTTATGGTTACTGTTCA
GAATATACGTTCTACTGCTGTAACTTTAGATCAGATTAGTATGTTTGTTTGAACTTCTTATTTAACT
TCTTTTTTATTGGTATTGTCAGTGCCTGTTTTGGCTGGTTCTTTATTATTTTTGTTGTTGGATCGTA
ATTTTAATACTTCTTTTTATGATGCTAATAAGGGGGGTAATCCTTTATTGTATCAGCATTTGTTTTG
GTTTTTTGGACATCCTGAGGTTTATGTTATTATTTTACCGGTGTTTGGGATTGTTAGTGAATGTGTT
TTATTTTTGACTGATAAGGATCGTTTGTTTGGCCAGACTAGTATGACTTTTGCTTCTATTTGGATTG
CTGTATTGGGGACTTCTGTTTGGGGTCATCATATGTATACAGCTGGTTTGGATATTGATACTCGTAC
TTATTTTAGTGCTGCCACTATGATTATTGCTATTCCAAGGGCTGTTAAGATTTTTAATTGATTGGGT
ACTTTTTTTGGTTCTCATCAAAAAATTCAGCCTCTTTGATGTTGAACTTATAGTTTTATTTTTCTTT
TTACTATTGGAGGTTTGAGCGGAATTATTTTAAGTTCTGCTAGTTTGGATATTATTCTTCATGATAC
TTATTATGTTGTGGCTCATTTTCATTATACTTTGAGCTTAGGAGCTGTTTATGGTATTTTTTGTGGG
TTTTGTTTATGGTTACCTTATATGTATGGTATTTCGTTTGATAGTTTGATAATAATGGCTGTGTTTG
TTTGTTTTTTTTTTGGTACTAATATAACTTTTTTTCCTATGCATTTTGCTGGTTTGCAAGGTATGCC
TCGTAAAATTTTAGATTATCCTGATTGTTTTTCAACTTTTCAGATTATTTCTTCGTTGGGTTCGGTG
ATTACTTTTGTTGGTTTTATTTTGTTTAATTATTTGATGATTGATTCTGTTTTTTTGTCTTGTTTTT
TGGGTGTTTCTTTTTATAATTATCATAGGCCGGCTTATACTATTAATGTCCCTCCTTTGTCTGATTC
TTTCACTGAGGAAACTTTTATTATAGGACTTCATTGGAAGGTTATTAGAAGGGATACTCCTTCTTAT
TCTTATCGTCGAGTTGGTTATGGTTATCATAGTAAGTAGTATTTTTTTTATTGTAGTTTTTTTTTTG
CTTTTTTTTTTTTTTGTTTGAGGTTTTTAGAGTGGGATTCTTTGAAGAGTTGTGTTATGATGTGTTT
AGGCATTGTTTTTATAAGTTGTTATTTATCTTTTGGCATTCATGTTTGATATTCTTATTTTATTGTT
TTGGTTTTTTTTAGTGGTATTTTTTCTTTGTTAACTTATTTTTGTAGTATGATTAACATTAATTATT
ATTATAATTATTATTATTTTTTTTTTTTTTTGTTTTTTTTTTTGTTTTTTTTTTTTTTTTTTGATTA
TGATGTTTTTTTTTTTTTTTTTTTTTTGATGGTAATTTTTTGTATGTTTATTATGATTTTAATTTTTGT
TATGTTTTTTGGATTATTTTTGTTTTGTTTTTGTTGTTGATTATAATTAGGTTTAGTTTTAACGGGG
GTGGTTATATGCGTGGGTTGTAGGTTGGTGTTTTTAATTCTTTAGTGTTTCTTCCTGCTAGGTTTTC
TTTGAGTTATATATGGAATTTTGGAAGTATATTGTTTGTTATGTTGGTTTCTCAGATTTTTACTGGA
TTTTTTTTGACTTTTTATTATACTTCTGGGGGGGCTTTTGCTTCTGTTCAGTATATTATATTTGAGG
TTAATATGGGTTGGTTTTTGCGTATTTTGCACTCTAATGGGGCTTCTATGTTTTTTTTGTTTATTTA
TTTTCATATTTTTAAGGGTTTAGTTTATGGTAGGTATCGTCTTCGTTTTGTTTGATTAAGTGGTATT
TTTATTTATTTTTTACTTAAGGGAGTTGCTTTTACTGGTTATGTTTTGATTTGAGGTCAGATGAGTT
ATTGAGCTGCTGTTGTTATTACTAGTTTGATAACTTCTGTGCCTTATTTAGGCAAATATTTGGTTTG
ATGAATTTGGGGTAGTTTTAGTGTTTGTGATAATACTTTGAAGTTTTTTTATTCTATTCATTTTATT
TTGCCTTGATTTTTGTTGTTGTTGGTTGTTGTTCATTTGTTTTTTTTACATTTTACTGGTTCTAGTT
CTGTTATTTATTGTCATGGTGATTATGATAAGGTTCATTTTTTTCCGGGCTATTGGTTGAAGGATGG
TTTAGATATTTTGTTTTATTTTTTTTTTTGTTTTGTTTAGATTGTATTTTTCTTTTAATTTGAGTGAT
CCTATGATTTTTGTGGAGTCGGATTCTATGGTTAGTCCTACTCATGTTGTTCCTGAATGGTATTTTT
TGTTGACTTTTACTATTTTGCGTTCTGTTCCTGACAAGTTATTGGTGGTTGTTTTAATGTTTAGTTC
TGTGTTTGTTTTAGCTATTCTTATTTGACCGGGTGCGTATTATTCTATTTTGGATAATTTTTTGTAT
TTTTTTGTTATGTGTTTTGTTTGGATTTTTTTTTGATTAACTTGAGCTGGGTATTATCCTACTGATT

ATCCTTTTAATTATTTTAATTTGTTTTGTACTTTTTTTTTATTTTTGTTTTATTTTTTTTTATTTGTTT
AGTGAATTATGTTGGTTGTAAAATTTTTAGTTATTTTGTTAAAGTTTCGTAAGTTTCATAAGATGGA
TTATAGTTATTATCCTTTGATGTTTAGTTTTGGTGTTTTTGGTGTTGATGTAGGTTTAGTTTTGTTT
ATGTGCTTAGGTATGTATTATCCTTTTTTTTTTTTGTTTTTTGTATTTAATTTATGTCTCTTTTTTGT
GGGTTAAAGATTTGCTTTTGGAGGATGTTAGTGGCAGTATTCTTTTTATGATTATCGTATTTTTGC
TCAGGGTTTTCGTTTGTTTTTGTTTAGTGAGTTGACTTTGTTTTTTTCTATTTTTCGAACTTTTCTG
GATTCTGCTTTGGGTCCTTTGACTTGATTGGGAGGTGTTTGATCTCCTTTAGGTATTTTGTCACCTG
ATTATCTTGGATTGAATGGCACTGCTAGTTTATTTTTGATAATGAATAGTCAATTTTTAAAGTATTC
TCGTCGTTATTTATGTTTAAATAGTTGTAAGTGTGAGTTGTTTTTGTTGTTTTGTATTTTTGTTGGT
TCTGGATTTTTGTGTTTTCAATTTTATGAATATAGTGATAATTGTTTTGGTATAAATGATAGTATTT
ATGGCAGTATTTTTTATGTTGGGACAGGTTTGCATGGTTTTCATGTTTTGGTTGGAGTTTTTTTTCT
TATGGTAAATTTTTTTCGTATTAAATTGTTTAATTTTAATTGGTATCATATTCAGGCTTATGATATG
TCTATTGATTATTGACGTTTTTTGGAGTGAATGTGAGGGATTATGTTTAGTTTATTATATGTTTGAG
GTTCTTAATTGTTTTTTTTATTTTATTTTGGTTTTTTGATTATGATTGTTTTTATTTTGCAGTCTGT
TGCTTTTTTGACTTTGTTGGAGCGTCATTTTTTAGGTGGTTCTCAGTGTCGAATTGGTCCTAATAAG
GTTGCTTATTGTGGTTTTTTTCAAGCTTTTTTTGATGGTTTGAAGTTGTTGAAAAAGGAACAATTAG
TTTTTTTTTGCTCTAGTTGGATTTCTTTTTTGTTTGTTCCTGTTTGTGGGTTTGTTTTGATAATTTT
TTTTTGGTTTACTTTGCCTTATTTTTTTTCTTTTTTGACTTTTGAGTATTCTGGTGTTTTTTTATTT
TGTTTGATGGGGGTTTCTGTATATTTTATTATGTTGTCAGGTATTTTTAGTGGTGGTAAATATTCTT
TTTTGGGGGGTATTCGTTCTTGTGCTCAGAGATATTCTTATGAGATTGCTTTTTCTGTTTATTTGTT
GGTTTTTTTTTTGTTTAATAAGAGTCTTTGTTTGTTTTTTAGTTTTAGTTTTTTTTTTTTTTTGTTTT
TTATTTCCTTTTTCTTGTTTGGTACTTGTTGATTTGCATCGGGCTCCTTTTGATTTTTCTGAGTGTG
AGAGTGAGTTGGTTAGTGGTTATAATGTTGATTATTCTAGTATGGGTTTTGCTTTTTTGTTTTTGGG
GGAGTATGGAAATTTGTTATATTTTAACTGTTTGGTTTCCAGGTTGTTTTTTGATATGAGTTTTTTT
TTTTTTTATTTTTTTGTTTGTTTTATTATTTTTTCTCGTAGTTCTTATCCTCGTTTTCGTTTTGATA
TGTTAATGAGTTTGTGTTGGTTTGTTTTTTTACCTTTTGGTTTTTATTTTTTTGGTTTGTCTTTTGT
TGTTTTTTTATTTTGCTTATTTAGTTTAATTTTTTTTGTTGTTTGTTTTTTTTTTGGTTTTTTTTGGT
TTTTTATTTATTTTATATGGAGTTGTCTAAGTTTGTGTTGGTGGGTGGTTTTTTTTTTGATGTTATG
ATTTTTAGTTTTTTGCATCAGGGGCTTCAGTCTAGTTTTTTTTTTAAGTTTGCTTGTTTTTTTCTTG
TAATGTTTTGGATGAGTGGGTTATTTTTTCCTTTGTTTAGTCCTTGGGCCTGTGTTGGTTTTTTGTT
TTTTTTAACTAATTTTTCTTGGTTGGGAGCTCGTGTTTTGACTTTGTCTATGGATAGTTTTTTGGTG
TTTTTTGAGGATGATCATTCTTGGGATTGGGTGTCTAGTTTAGTTATGTTTTTTTCTCATTTTTTGA
GTTTTTTTATGAGTGGTGTTGCTTTGACTTTGCGTATCAGTATTATTTTTTTGATTGGTCATTTTTT
GATGTTTACTTTTTTGGATTTTGGTTTTTTTTGTTCTTTTTTTTCTTTATTGTTGTTGGTTCCTGTT
GAATTGTTTTTTGCTTTTTTGCAGAGTTATATTTTTTTGACTTTGATTTGTATGTTTTTGTGTAATA
TGATTTAAATTTATTTTCAGAATTATATTTTTCCTAATCCTGGTAGTTCTTATGTATATTGTTGTTA
TTATATTCATAATTATTATTCTCATGTTATTTTTTTTTGGTTTTTTTTTAATGTTGTTAGTTTGTATT
GGGGTTTATTTTTTTGGTAGTTCTTTTAAGTTTAATTTGAAGCGGAGGGATAGACGTATGATTGAAT
TGGTTTTGCAGGTGTTGATTGTTAATTTTTTGATTATGATAGCTGGTCCTGGTTTTTGGTTAATTCA
GTATCAGGGTCGTATATTTCGTCAGTCTGAGTTGACTTTGAAGGTTATTGGTCATCAGTGGTATTGG
AGTTATGAGTATGGTGATAGGGGGAAGTTATGTTTTGATTCTTTTATGAAATCTTTGGATGATTTGT
CGTTGGGTGATTATCGTTTATTTGAGGTTGATAATCGTTGTGTTTTACCTGTTGGAGTTAATGTAGG
TATTTATTGTACTTCTAGTGATGTTATTCATTCTTTTGCTGTTCCTAAGTGTTTTGTTAAGATGGAT
GCTTTGAATGGTTTGTTGACTAAGATTACTTGTAATTTTTCTTGTTCTGGTTTATTTTATGGTCAGT
GTTCTGAGATTTGTGGGGCTAATCATAGTTTTATGCCTATTGTTCTCGAGTTAACTTCTATGGAGTG
TTGAAAGGGTTGATGTGTTAATTTTTTATTGGTTTCTTTTTTTAGATTTTTTTGTTGTTTTTTTATT
TTCTTTTTTTGTTTCGTTATTGATGTATTTTTTTTCAATATTTGTTTCTTATAAGGATTTTTTTGAG
AGTAAAGTAAGATCTTATGAGTGTGGGTTTGATGTTTGTAAAAAGGTTCATGTTGGTTTTAATTTGG
TTTTTTTTTCTATTGTGTTGTTGTTTGTTGTTTTTGAATTGGAGGTTATTATTTTTGTTTTTTTGGT
TCAGGGTGATTTGTTTAGTGTGTTTTCTTTTTTTATGTTTTTTTTTTATGTTGTTTTAAGTTTTTAT
ATAGAATGGAGTTTTGGTAAGTTGATTTGAATTTGTTTTGTTATTGTTTGTTTGGTATATTTTGTTT
TTTTATTTTTTTTTGTTTTCTGTTATTTTTTTTTTTGCCCTATGGTAAGTGAGTTTTTAATATAGGTT

TTAATGATTTTTTTAGTTTTATTCTGGTTTTTAATTTTGAGATTTGTTTGTTAATTTTTGTTTTATT
GTTGGTTTCTTTTATGGTGTTTATTTATGGGTCTTTTTATATGTTGGGTGTTTCTCGTTTGTTTTAT
TTTTTTTTTATTTTATTTTTATTTGTTTTTAGTATGTGTGGTCTTATTGTTTTTAGGGGGAGTGTTG
TTTTAACTTTGATTTTTTGGGATTTTTTGGGTGTTAGTAGTTTTTTTTTGGTTTTATTTTATGGGAA
TGTTGGTTCTCGTAATGGTGCCATGAGAACTGTTTTTACTAATCGTATTGGTGATTTTTGTATTTTT
TTTTTTTTTAATGGGTTTGTTCTTTTTTCTTTGGGGCATTTTTCTTATCAGTTTTTTAGGTCTTTGA
TAATTTTTATGTTGTTTTTGTCTTCTTTTATTAAGGGGGGTCAGTATCCTTTTGGTAGTTGGTTGCC
TAAAGCTATGGCTGCTCCTACTCCTGTTAGTTGCTTGGTTCATAGTAGTACGTTAGTTACTGCTGGT
GTTATGTTAATGGATTGTTATGTATATGTTTCTATAAGTGCTGATGTTTTATCTTTTGTTTTTTTATG
TAGGATTTTTTACTATATTAGTTTCTGGTGTTTGTTCTTTATTTGAGAGTGATGTTAAGAAAGTTAT
TGCTTTGAGAACTATGTCTCAAATTGGTTTTTGTTTTTTGGCTATTGGTAGGGGTTTGCATTATTTG
TCTTATGTTCATATGATTAGTCATTCGTTATTTAAAAGTTTATTATTTATGCAGGTTGGCTATGTTA
TTTATATTAATTTAGGTCAGCAGGATTATCGAGGATATTCTTTTTATAATTTATGTTGTCCTGTTTT
GGTTCAGTTGCAAGTGTTTTTGTCTGTTGTTTGTTTATGTGGATTGTTGTTTACTAGAGGGGGCTGT
AGTAAGGAGTATTTTATGTCTCGGTTTTATTATGATTCTTTTAGTTTTTTTTTGGTTTTTTTTTATT
TTTTGGGGGTATTTTTAACTTTTTGTTATTGTTATCGTATGTTGTTTTTGTTTCGGGTTGGAATTTC
TGGTTTGGATTATGTGGGTTTTTCTAGTAAGTTGTTTTATGGTTCTTGTTTTTTTTTAGTTTTTTTT
TCTGTGGTTTTTACTTTTTGGTGAATTTTTGGTTTGCTTTCTTTTTCTGTTGCTTTTAATCGTTTTG
AGTTTTTGGTTGTTAATTTTTATTTTTTTTTTGTTTATTGTTTTTTAAATTATTTTTTTCGTTATTT
TTTTTTGGAGTTTAAGAATAAGTTTTTTATGGATAGTTATTCTTTTATTGTTTTTAAGATTGTTCCT
AGTTTTTTTTATTTTGATAGTCTTGCTATGGGTTTTAATTATTTTTTTTTTTGGTATTGCGCGTTTTA
TTTCTTTTTTTTTTTTTTCTTTGTTTCGTGGGTATTATCATACGGGTGTTTTGATTATTTTTTTTTT
TATGTTGTTTTTTTTGTTTTTTTAGATTGTGGTGTTTTTTGTTTTTTTTGTGATAGTGTTTTTGAGA
TGTGTTAATTTTTGTGATGTGGATTATATTATTTGGTGGAGGGTTTTTGTTGTTTGTACTTTTGTTT
TTATTTTTTTTGTTTGTAATAATAGATTGGATAATTTGGGTTGTATGGTTAATTACTATGTTGTTCA
GGAGATTTGTGGTTATTATTTTTTGCTTTTTGATAGTTGAAAGTTGCAGTTTTTGTTTTTAATGCTA
AAGTCTGGTTCATCACCTTTTCATTTTTGGGTGTTTAGAGTTTTGGGTGGTTTGAAAAAGTGGTTTG
TTTTGTGGTTTTTGACTTTGCAGAAATTGCCTTATTTTGTTGTGTTAATTAATTTTTGTAGTGATTT
TTTTTTTTTTTTTTTGTTTTTTGGGATGGTTGTGTGTTATTTACAGTTTTTTTTATTGCGGAATTAT
AGTGATGTGGTTATTATTGGCTCTGTTGAGTCATTTAATTGATTACTTTTGTTGGGGATTTTTTCTT
TTAATGAGGGGTTTGTTTTTTTTTTTTTTTTATTATGTTACTATATTTTTTGTTATTTCTTATGTTTA
TAGTGGATTTTTGAGTTTTTTTAGTTTGGAAATGTTAATAGTGTTTTTTAATGTTCCTTTGAGTATT
ACTTTTTTTCTAAAGGTTTTGTTATTGTTTGGCTCTGGTTTTTTTGTTGGGTTTTATTATTATTTTT
TGTTGTTAATTATACCTTTGATATCTTTGAGGATTGGTTATTTATTTTTTTTGATTTCTATGTCGGG
GTTTAATCAGGGTTTGAAGTATTATGATTTTTTTGTTTGAATTTTGTTATGTATTGGTTTTTTGTCT
TATTTTTAGTTGTGTTTATTTTTTTTTTGTGGTTTTGTCTTTGTTTTTTACTCCTTTTTTATTTTTTG
TTTTTTTTATGTTTTTCATTTTGTTTGGTTTTTTTGATTTTTCTTGAAGGGGTTTGTTTTTTTTTTT
TGATTCTTTTAATTTTGTTTTTTTGTCTTTTATAAGTGTTTTTGTTTTGGGGTTTATTTGTGTGTCT
GAAATTTTGGTTGGTTTGGTTTTTTATAGTTGTCTTGTTGTTTTTTTTTAGTGTTTGTTTTTTTTTATT
CTGGTAGATTTTTGGTTTTGTATGTATTTTATGAGCTGACTATAATTCCTATGTTGTTTTGTTTGTT
GGGTTATGGTCGTCAGGTTGAGAAGATTAGTGCTTGTTATTATTTAATTTTTTATACTTTGTTTTTT
GGTATACCTTATTTGTTTTTGTATAGTCATGTGTTTTTTTTTATAAATTTTGTTTATTATGACTTTT
TTTTTTCTTATGAGTTTATTTTTTTTATTGAGTTTGTGTTTTTTTGGTTAAATTTCCTGTTTATTTTTT
ACATGTTTGGCTGCCTAAGGTTCATGTTGAGGCGCCTACTAGTTCTAGTATAATTTTGGCGGGTGTT
ATGTTAAAGTTAGGTGGTGCTGGTGTTTATCGTATTAGTAAGTCTTTTAATTATTATAATTTTGAGT
TTTTGATTTTTTTTTTCTTTGGTTAGTATAATTTTTTGTTCTTTTATTTGTATGGTACAGAGGGATTG
TAAGTCTTTGGCGGCTTATTCTTCTATTTGTCATATGGGTTTTGTGTTATTGTCTGAGTTAAGTATG
GTTTATTATGGAAAGTCTATGGCTTTGGTTATGATATTATCTCATGGTTATACTTCTGTTTTAATGT
TTTATTTTATTGGTGAGTTTTATCATATTGCTAATAGTCGGTTGGTTTATTATTTACGAGGTTTTTT
TTGTGTTAGTATGTTGTTTTGTTTGATATTTTCTTTGACTATGTTGTCTAATTTTGGGTTTCCTAGC
TCTATTACTTTTTTTTTCTGAGTATTTGATGTTTAATTGATTTAGTTCTATTTTTTTATATTAGAGTTT
TGTTTTTTTTTTTTTTATTATTTGTTGTCTTTTTATTATTCTATTTATGTTTTGATTTGTTTTTTTGT

TGGCAATAAGTTTAGTTATGTGTTTGATGGGCGGGGTATTGTTTGTCTGCCTGTGATGTTTATAATG
TATAATTTTTTTTGGTTTATTTTTGTTATTTAGTATTAAGTTTGATTTTGGTTTTAGTTGTATTAAG
ATAGTATTACTTATTTTTAGTTTATCGAGTGTGTAATTTTTTGTTCACTGGTAGTTTTTTAATTGTT
TTATTGATGTTCCAGAATAATCGGCTATACATTTTAATTTTTAACTCTATTTGTTGTAATATTACGA
TTTTTTTTGTTTTTTTGTTTTATGTTTTTTTTTGTAAAATATTTAAATTTATTTATGTTTTTTTGTA
ATATTGAAAATTTGGTGTTTGAACTGGATTAGTACCCAGGTAATCAAAATTTATTAATTCGGGAGTA
AAGTTTTGTTTAAACCGAAAAAATATTGACTGACTTTAGATTTTTCTTTGGAATATGTGTTTTTTTG
GAGAGCCCTCTTTTATAGTGAATTTTGTTGGCGCATGTATGATTGTTTAGTTTTACTTTTTTGGTA
ATGCTTTGTGTTTTATACATTTAAAACAGATATATATTTGGCTTATGGATTTATTTTTCATGTGTTA
CTATTGTTAATTTTCTTTGGATTAATTTTTAATTTTTTTGTTGAAATTGGAAAAGAAAGTAATTTTT
TCTTAATGTAATAATGAATTTAATAAATAAAGTGGTACAAACCATCCGTCAATGGCCTAAAGGGGCG
TAAGTTGTAGTATGGTAGAGGTAAGGAAACTTGTTTCTATTTTTTTTTGGTATTTTTTTTTATTTTT
TTTATTTTGGTATTGCATATCAATGGATGTTGTTATCATAAATATGAAAAATAGAATTGAAATGTAA
AAATTTGGATATGTTTTTTTTTTACGAAATTAAATATATTTTTTTTTTGTTATTGATATAACGTATT
TCTATTTCTGTTTGTTACTTTGTTATGGTTTAAGTTATTTTATTTTTTTTATTTGTTTTGAGTTTTT
TATGTAAATGTTTTTTGTTTTTTTGCTTATATTTTTTTTTGTTTTGTTTTTTGTTGTTTTAGTTAAT
CTTTTAACTATTTTATAATTTCTATTTTAGTAAAATATTTTTTGTGTTGTTTTTTTTTTTAATTTTT
ATTTTTGAACTTGGTGTATTGTTAAATGTTTATTAAAAACTTAGGTTTTTATGTAAAGTTGTCTTCT
GCTCTATGATTTTTTTTAAATGGCAGCCTTAGCGTGATGGCATAAAAGTAGCGTAAGTGATTTGTTT
TTTTATTGATTTCAAGTATGAATGAAGTTTTAACAATTTTTTTGTTTTTATTTTTTTTTGAATTATT
TTTTTAATTAAAAATTATTAGTTATAATATAACAAATATAAGTCTTCGGAAATTTTTTTTTAATAAT
TTATTTTTTTAATTGTTAATATTTTCTTGGGAATGGATTTTATAAAATATTTTTTTTACTATTTTTA
TTTTTTTAAAAATTACTCCGGAGTTAACAGGGTTGTGGACATATAAATAGTTTTTATATTAGTGTGC
TGCGCTACATCGATGTTGTATATTTTTTGTGAGAAGAGAGAGATATTTTTTTTTTTGAGACTGTTCT
TCTTGTATAAAAATTGAACTTGATATTAGTTTAGTTCGTCGTGAGACAGAGCGGTTTATCTTGTATA
TTTTTTTTTTTTTGCGGTATTAGTACGAAAGGAAAGTGATGCAGGTTAATATTTATGACCTTTTTAT
TTGGATGGATTTT

>O.flexousa(HQ214004)
ATTTTTTGTGGAATGACTTTTGGCAATAGTATGAAACAGAGTATTATTAATACTGTTAATCATAAGA
CTATTGGTACTTATTATATTATTTTAGGTTATTGAGCAGGATTGGGAGGTTCTGTTTTGTCTATATT
AATTCGTTTTGAGTTATCTAGACCTGGTGGATATTTGTTTTTTGGTAGTGGACAAGTTTATAATTCT
GTTCTTACTATGCATGGTGTTTTAATGATTTTTTTTTTGGTTATGCCTATTTTAATTGGTGGTTTTG
GTAATTGGATATTACCTTTAATATTGGGGGCTCCAGAAATGGCTTTTCCTCGTGTGAATGCTTTATC
TTTTTGATTTACTTTTGTGGCTTTGTTGATAGTTTATCAATCTTTTTTTATTGGGGGTGGTCCAGGT
AGTAGTTGAACTTTTTATCCTCCTTTAAGAGTAGAAGGTCAGCCTGAATTGTCTTTGGATACTATGA
TTTTGGGTTTGCATACTGTTGGTATTGGTTCTTTGTTAGGTGCTATTAATTTTATAGTCACTACTCA
GAATATGCGTTCTACTGCTGTGACTTTGGATCAAATTAGTATGTTTGTTTGGACTTCTTATTTGACT
TCTTTTTTATTGGTTTTATCTGTTCCTGTTTTAGCAGGATCTTTGTTGTTTTTATTGTTGGATCGTA
ATTTTAATACCTCTTTTTATGATACAAAAAAGGGTGGTAATCCTCTTTTATATCAGCATTTATTTTG
ATTTTTTGGTCATCCTGAAGTTTATGTTATTATTTTACCGGTTTTTGGTATTATTAGAGAGGCTGTT
TTTGTTTTGACTGATAAGGATCGTTTGTTTGGTCAGACTAGAATAACTTTTGCTTCTATTTGAATTG
CTGTTTTAGGTACTTCTGTTTGAGGTCATCATATGTATACAGCTGGTTTGGATATTGATACTCGTAC
ATATTTTAGGGCTGCTACTATAATTATTGCTATTCCTAGAGCTGTTAAGATTTTTAATTGGTTGGGT
ACTTTTTTTGGTTCTAATCAGAAAATACAGCCTTTGTGATGTTGAACTTATAGTTTTATTTTTCTTT
TTACAGTAGGTGGTTTAAGAGGAATTATTTTGAGTGCTGCTAGTTTGGATATTATTTTACATGATAC
TTATTATGTTGTAGCTCATTTTCATTATACTTTGAGTTTAGGTGCTGTTTATGGTATTTTTTGTGGT
TTTTGTTTGTGACTTCCTTATATGTACGGTATTTCTTTTGATGGTATTATGATGATAGCTGTATTTG
TATGTTTTTTTGTTGGTACTAATATGACTTTTTTTCCTATACATTTTGCTGGTTTACAAGGTATACC
TCGTAAGATTTTAGATTATCCTGATTGTTATTCTACTTTTCAGATTATTTCTTCTTTGGGTTCTGTT
ATTACTTTTGTTGGTTTTATTTTATTTAATTATTTATTGGTTGATTCTATTTTTTCTTCTCGTTTTT
TAGGTGTTTCTTTTTATAATTATCATAGTCCAGCTTATGCTCTTAATGTTCCTCCGTTACCGATTC

TTTTGTTGAAGAGTCTTTTATTATAGGTCTTCATTGGAAGATTATTAGTAAGAATACTCCTTTTTAT
AGTTATCGTCGTGTTGGTTATGGTTATCATAGTAAGTAAATTTTTTTTTACATAAGAATTTTTTTG
CTTTTATATTTTTTTGTTTGAGCTTTTTGGATTGGGATCCTTCAAAGAGTTGTATTATAATATGTTT
AGGTATTCTGTCTATAAGTTGTTATATTTCATTGGGTGTACATGTATGATATTCTTATTTTGTCGTT
TTGATTTTTTTTAGTGGTATTTTTTCTTTGTTAACTTATTTTTGTAGTATAACTAATTTTGTTTTTT
ATTACAATTATTATTTTTTTTTTTTTTTTTTTTTGATTGTTTTTTTTTTTTTGTGTTTATATTAGATTA
TGATTTTTTTTCTTTTTTTTTTCGATTTTAATTTTTTGTCAATTTGTTATGATTTTAGTTATTATTAT
GTTTTTTGAGTTGTTTTTATTTTGTTTTTGTTTTTAATTATAGTTAGTTTTAGGCTTAATGGTTCTG
GATTTATACGTAGTTTGTAAATTTTTTTAGTTCTTTTTAATTCTTTAATTTTTTTACCTGCTAGTTT
TACTTTAAGTTATATGTGAAATTTTGGTAGTATGTTGGGTATTATGTTGATATCTCAGATTTTGACT
GGTTTTTTTTTGACTTTTTATTATACTGCAGGTGATGCTTTTAGTTCTGTTCAATATATTATATTTG
AAGTTAATTTAGGTTGGTTGTTGCGTATTATACATTCTAATGGGGCTTCTATGTTTTTTTTGTTTAT
TTATTTACATATTTTTAAGGGTTTGATTTATGGTAGATATCGTCTTGTTGGTGTTTGGTTGAGTGGT
GTTGTTGTTTATTTTTTGTTAATAGGAATTGCTTTTACTGGTTATGTTTTGATTTGAGGGCAGATAA
GTTATTGAGCAGCTGTGGTTATTACTAGTTTGATAACTTCTGTTCCTTATTTGGGTAAATATTTAGT
TTGGTGAATTTGGGGTAGTTTTAGTGTTTGTGAGAATACTTTAAAGTTTTTTTATTCTATTCATTTT
ATTTTACCTTGATTGTTAATGGTTTTAGTTATTGTTCATTTATTTTTTTTTACATTTTACTGGTTCTA
GTTCTAGTTTATATTGTCATGGTGATTATGATAAGATTCATTTTTTTCCTAGTTTTTGGCTGAAGGA
TGGTCTTGATATTTTTTTTTATTTTTTTTTGGTTTTATTTAGTCTTTATTTCTCTTTTAATTTAAGT
GATCCTATGATTTTTGTTGAGTCTGATTCTATAGCTAGTCCTGCTCATGTTGTCCCTGAATGGTATT
TTTTGTTTGCTTTTACTATTTTACGTTCTGTCCCTAGTAAGTTATTTGGTGTTATTTTGATGTTTAG
TTCTGTTTTTGTTTTAGTTGCTTTGATTTGGCCTGGTAGTTATCAACCTATTTTGGATAGGTTTTTG
TATTTTTTTGTTATATGTTTTGTTTGAATTTTTTTTTTGGTTAACTTGGGCTGGTCATTATCCTACTG
ATTATCCTTTTAATTATTTTAATTTGTTTTGTACTCTTTTTTTATTTTTGTTGTATTTTCTTTATGTG
TTTAATTAATTTTATTAGTGAGTTGTAAATTTTGCTAAAGTTTCGTAAGTTTCATAAGATGGAATAT
AGTTATTACCCTTTGATGGTCGGGGCTGGTATTTTAGGATTTGATGTGAGTTTAGTTCTGTTTATAA
ATATGGGTATGTTTTATTCTATTTTTATTTGTCTTTTGTATTTAATTTATGTTTTTTTTTTTATGGAT
AAAGGATGTTATATTGGAAGATGTTAGTGGTCAGTATTCTTATTATGATTATCGTATGTTTAATCAG
GGTTTTCGTTTGTTTCTTTTTAGTGAATTAACATTGTTTGCTTCTATTTTTTTGGACTTTTTTAGATA
CTGCTTTGTGCCCTTTAACTTGGTTGGGGGGAGTTTGATCGCCTTTGGGAATTTTGTCTCCTGATTA
TTTAGGTTTAAATGGTATAGCTAGTTTATTTTTAATGATGAATAGGCAGGTATTAAAGTATTCTCGC
CGTTATTTATGTTTAAATAACCATAAGTGTGAAACTTTTTTAATGATTTGTATTTTTATTGGAGCGG
GGTTTTTATGTTTTCAGTTTTATGAGTATAGTAATAATTCTTTTGTTATGAGAGATAGTGTTTATGG
TAGTATTTTTTATATAGGTACTGGTTTACATGGTTCACATGTTTTAGTAGGTGTTTGTTTTTTTAATT
ATTAATTTTTTTCGTATTAAATTGTTTAATTTTAATTGGTATCATATGCAGGCTTATGATATATCTA
TTGATTATTGGCGATTTTTGGAGTGAATGTGGGGTATTATGTTTAGTTTATTATATGTTTGAGGTTC
TTAGTTTGGTTTTTTTTTATTATTTGGGCTTGTTGATTATAATTGTTTTCATTTTGCAGGCTATTGC
TTTTTTGACTTTGTTGGAGCGTCATTTTTTAGGTGGGTCTCAGTGTCGTATTGGTCCTAATAAGGTT
GGTCTTTCTGGTATTTTGCAGGCTTTGTTTGATGGTTTGAAGTTGTTAAAGAAGGAGCAGTTGCTGT
TGTGTTTTTCTTCTTGATTGTCTTTTTTATTTATACCTATTTGTGGTTTTGTTTTGATGATTTTTTT
TTGGTTTACTTTGCCTTATTTTTTTGTTTTTTTGTCTTTTGAGTATTCTGGTATTTTTTTGTTTTGT
CTTATGGGGGTTTCTGTTTATTTTATTATGCTTTCTGGTATTTTTAGTGGTAGTAAATATTCTTTTA
TTGGAGGTTTGCGTGCTTGTGTTCAGAGTTATTCTTATGAAATTGCTTTTTCTGTTTATTTATTGAT
TTTTTTATTGTTTAATAAGGGTTTGTGTTTATCTTTTAGTTTTTGTTTGTTTTTTTTTTTGGTTTTT
TTTCCTTTTTTCTGTTTAGTCCTTGTTGATTTGCATCGGGCTCCTTTTGATTTTTCTGAGTGTGAGA
GAGAGTTAGTTAGTGGTTTTAATGTAGAGTATTCTAGAGTTGGTTTTGCTGCTTTGTTTTTGGGGGA
GTATGGTAATTTGCTTTATTTTAGATGTTTAGTTTCTAGTTTGTTTTTTAATATAAGTTTTTTTTTT
TTCTATTTTATTGTTTGTCTTATTATTTTTTCTCGTAGAGCTTATCCTCGATTTCGTTTTGATATAT
TGATGGGAGTTTGTTGATTTTTGTTTTTGCCTATTGGTTTTTATTTTTTTGGTTTATCGTTTGTTGT
TTTTATGTTGTGCTTATTTAGTTTAAATTTTTTATTTTGTTTTTTTCTCTTATTTGGTTTTGGTTAA
TTTTTTACTTATTTTATATGGAGTTGTGTAAGTTTAATAGTTTGGGTTTTTTGAGTGGTTTTTTAGG
TATTTTGGTTTCTAGCTTTAGCCATCAAAGTTTTCAGTCTAGTGTTTTTTTTAAGTTTGTAGTTTTT

TTTCTTGTAGTTTTTTGGATGAGTGGTTTATTATTTCCTTTATTTAGACCTTGGGCTTGTGTTGGTT
TTTTATTTTTTCTTACTAATTTTTCTTGGTTAGGGGTTCGTACTTTTACTTTGGCTGTTGATAGTTT
TTTGGTTTTTTTTGAGGAGGATCATTCTTGAGACTGATTTTCTAGTTTAGTTATATTTTTTTCTCAT
TGATTAAGTTTTTTGATGAGGGGAGTAGCTTTAACTTTACGTATTAGTATTATTTTTTTAATTGGTC
ATTTTTTGATGTTTACTGTTTTAGATATGGGTATGTTTTATTCTTTTTTTTTATTATTATTTGTAAT
TCCTGTGGAGTTATTTTTTGCTTTTTTGCAGAGTTATATTTTTTTGACTTTAATTTGTATGTTCTTG
TTAAATATAATTTAAATTTATTTACAAAATTATGTTTTTCCTACTCCTGGGAGTTCTTATGTTTTTT
GTTGTTATTATATCCATAATTATTATTCCCATATTATTTTTTTTGGGTTTTTTGTAATGTTTTTAGT
TAGCAGAGGAGTGTATTTTTTTGGTAACTCTTTTAAGTTTAATTTGAAGCGTAGTGATAGTCGTATG
ATTGAGTTGGTTTTACAGGTTTTAATTGTTAATTTTTTGATTATAATGGCTGGTCCTGGGTTTTGGT
TGATTCAATATCAGGGTCGTATATTTCGTCAATCTGAGTTGACTTTAAAGGTTATTGGTCATCAGTG
ATATTGGAGTTACGAGTATGGTGATAGAAGTAAGTTATGTTTTGATTCTTTTATGAAGTCTTTGGAT
GATTTGTCTGCTGGGGAATTTCGGTTATTTGATGTTGATAATCGTTGTGTTTTACCTGTTGGTGTTA
ATGTTGGTGTTTATTGTACTTCGGGTGATGTTATTCATTCTTTTGCTGTTCCTAAGTGTTTTATTAA
GATAGATGCTTTGAATGGATTGTTGACTAAGGTTACTTGTAATTTTTCTTGTTCTGGTTTGTTTTAT
GGTCAATGTTCTGAGATTTGTGGTGCTAATCATAGGTTTATGCCCATTGTGTTGGAGGTGACCTCTT
TGGAGTGTTGAAAGGGTTGATCTGTTAGTTATTTACTGAGTTAACCTTTTTTGAATTTTGTTGTTGT
TTTTTTTTTTTCTTTTTTGGTTCCTTTTGGTATGTATGTTATGTCTTTTTTTGTTTCTTTTAAGGAT
TTTTTTGATGCTAAGTTAAGTTCTTATGAGTGTGGTTTTGATGTAGTTAAGAAGGTTCATGTTGGTT
TTAGTTTGGTTTTTTTTTCTATTGTTTTGTTGTTTGTTGTTTTTGAGTTGGAAGTATTGATTTTTAT
TATTTTAATTCAAGGTGATTTTTATAGTATGTTGTCTTTTTTTTTATTTTTTTTTTATGTTGTTTTT
AGTTTTTATATGGAGTGATATTTTGGTAAGTTGATTTGGTTTTGTTGGTTATTGTTGTTTGTTTGGT
ATATTCTTATTTTTTATTTTTTTTTACTTGTTGTTTTTTTTTTTGTTCCTTATGGTAAGTGAAGATA
TAGTTTTGGTTATAGTGATTTTTTTAATTTTACTTTTACTTATAATTTTGAGGTGTGTTTATTTTTT
TTAGTTTTGTTATTGGTTTCTTTTATGGTTTTTGTTTATGGTTCTTTTTATATATTGGGTGTTTCTC
GTTTGTTTTATTTTTTTTTTTTTCTTTTTTTTATTTGTTTTGAGCATAGGCGGTTTGATTGTTTTTAG
TGGTAGAATTATTTTGACTTTGATTTTTTGAGATTTTTTGGGGGTTAGTAGTTTTTTTTTGGTTTTG
TTTTATGGTAATGTTGGTGCTCGTAGGGGTGCTATAAGTACTGTATTTACTAATCGTATTGGTGACT
TTTGTATTTTTTTGTTTTTTAATGGTTTTGTATTGTTTTCTATGGGTTCTTTGTCTTATCAGTTTTT
TGGTTCTTTGATGGTTTTTATGTTGTTTGTTTCTGCTTTTATTAAGGGTGGTCAATATCCTTTTGGT
AGTTGGTTGCCTAAGGCTATAGCTGCTCCTACTCCTGTTAGTTGTTTGGTTCATAGTAGAACTTTAG
TAACTGCAGGGGTAATGTTGATGGATTGTTATGTTTATGTCTCTTTGAGTTCTGATGTTTTATCTTT
TGTTTTTTATGTTGGTTTTTTCACTATGGTTTTTTCTGGTTTTTGTGCTTTGATTGAAGAGGATGCT
AAGAAGATTGTTGCTTTGAGTACTATATCTCAAATTGGTTTTTGTTTTTTGGCTATTGGTAGTGGTT
TACATTATTTGTCTTATGTCCATATGATTGGTCATTCTTTTTTTAAAAGATTATTGTTTATACAGAT
AGGTTATTTAATTTTTATTAATTTTGGTCAGCAGGATTATCGTGGTTATTCTTTTTTTGGTTTTTGT
GCTCCTGTTTTAGTTCAATTACAAATTTTTTTGTCTGTTTTTAGTTTGTGTGGTTTGTTGTTTACTA
GTGGGAGATGTAGTAAGGAATATTTTATGTCTCGTTTTTATTATGATTCTTATGGTTTTTTTTTAGT
TTTTTTTTATTTTTTTGGTATTTTTTTGACTTTTTGTTATTGTCACCGTATGATATATTTGTTTCGT
GTTGGAGTTTCTGGTTTTGACTATGTTGGTTTTTCTAGTAAGTTGTTTTATTATTCTTGTTTTTTTT
TGGTGTTTTTTTCTGTTGTTTTTACTTTTTGGTGGGTTGTGAATTTGTTGTCTTTTCCTTTAGCTTT
TAATCGTTTTGAGTTTATAGCTGTTTATTTTTATCTATTTTTTGTTTATTGTTTTTTTAATTATTTT
TTTCGTTATTTTGTTGTTGAGCTTAAGGGTAAGTTTTTTATGGATCGTTATGCTCGTTTTATTTATA
AGTTTTTTCCTAGTCTTTTTTATTTTGATAGTTTCATTATAGGTTTTAATTATTTTTTTTTTGGTTT
GTTTCGTTTGTTTTCTTTTTTTTTTTTTTCTTTGTTTCGTGGGTTTTACCATATTGGTGTTTTGATG
GTTCTTTTTTTTTATGTTGTTTTTCTTGTTTTTTTAAATTTTGTTGTTTTTTATTTTTTTTTTGTTGT
TGTTTTTGAGATTTGTTAATTTTTGTGTTATTGATTGTGTTGTTTGGTGAAGAGTGTTTGTTATTTG
TACTTTTGTTTTTATTTTTTATGTTGGTGGTAGTAGGATAGGGTTTAGAGGTTGTTTGATTAATTAT
TATGTTATTCAAGAGGTTTGTGGTTATTATTTTTTAGTTTTTGATAATTGAAAGTTGCAGTTTTTGT
TACTTATGTTGAAGTCCGGGTCTTCTCCTTTTCATTTTTGGCTTTTTAGAGTTCTAGGTAATTTAAA
TAAGTGGTTTATTTTGTGATTTTTGACTTTGCAGAAGTTGCCTTATTTTGTTGTTTTAGTTAACTTT
TGTGGTGATTTTTTTTTTTTGTTTTTGTTTTTTGGTATAATTTTTTGTTATTTTCAGTTTTTTTTAT

TACGTAATTATTGTGATTTGTTGGTTGTTGGCTCTACTGAGTCTTTTAATTGGTTATTGTTGCTTAG
AATTTTTTCTTTTAATGAGGTTTTTGTTTTATTTTTTTTTTTATTACTTTGTTATGTTTTTTGTTATC
TCTTATGTTTATAACGGCTTTTTGAGTTTTTTGAGTTTGGAGATGCTGATGGTTTTTTTTAATGTTC
CTTTGAGTATTACTTTTTTTTTGAAGGTTTTTTTATTGTTTGGTACTGGTTATTTTGTGGGTTTTTA
TTATTTTTTTTTGTTGTTGGTTATACCTTTAATGTCTTTGGGTGTTGGTTATTTATTTTTTTTTGGTT
TCTATAATAAGTTATAATTATGGTTTAAAGTATTATGATTATTTTGTTTATGTTTTGTTTTGTATTG
GTTTGTTGTCTTGTTTTTAGTTGTTATGCTTGTTTTTTTTTGTTACTTTGTTGTTTTTTTTTAGTCC
TTTTTTGTTTTTTGTTTTTTTTATGTTTTTTGTTTTGTTTGGGTTTTTTGACTATTCTTGATGTGGT
AGTTTGTTTTTTTTTGATTCTTTTAATTTTGTTTTTTTGTCTTTTATGAGTGTTTTTGTTATGGGTT
TTGTTTGTGTGTCTGAGTTATTGAGTGGTTTAGTTTTTTATAGTTGTTTGGTGGTTTTTTTTAGTGT
TTGTTTTTTTTATTCTGGTAGTTTTTTAATGCTGTATGTTTTTTATGAGTTGACTATAGTACCTATT
TTGTTTTGTTTACTGGGTTATGGTCGTCAAATTGAAAAGGTTAGTGCTTGTTATTATTTGATTTTTT
ATACTTTGTTTTTTGGTATGCCATATTTATTCTTGTATAGCCATGTTTTTTGTTTTTTGAATTTTGT
TTATTATGATTTTTTTGTTTCTTATGAGTTTGTTTTTTTGTTGAGTTTATGTTTTTTGGTTAAGTTT
CCGGTTTATTTTTTTCATGTTTGGTTACCTAAGGTGCATGTTGAGGCTCCAACTAGTGCTAGTATGA
TTTTGGCTGGAGTTATGTTGAAGTTGGGAGGGGCTGGTGTGTATCGTATTAGTAAGTCTTTGAATTT
TTTTGGCTTTGAAATATTGGTTTTTTTTTCTTTGATTAGTATAGTTTTTTGTTCTTTTATTTGCATG
ATTCAGAGTGATTGTAAGTCTTTAGCTGCTTATTCTTCTATTTGTCATATAGGTTTTGTTTTGCTTT
CTGAGGTGAGTATAGTTTATTATGGTAAGTCTATGGCTTTGGTGATGATATTGGCTCATGGTTATAC
TTCAGTTTTAATATTTTATTTTATTGGGGAGTTTTACCATATTGCTAATAGTCGTTTGATTTATTAT
TTGCGAGGGTATTTTAATGTAAGTATGTTGTTTTGTTTGATATTTAGTTTAACTATGGTATCTAATT
TTAGTTTTCCCACTTCTATTTCTTTTTTTTCTGAGTATATAATGTTAAATTTTTTTAGTTCTGTTTT
TTATATGGGTTTTTTGTTTTTGTTTTTTTATTATTTAGTTTCTTTTTATTATTCTGTTTATATTTTA
GTTTGTTTTTTGATTGGTAATAAGGTTAGTTATGTTTATGATGGTCGTAGTGTTATTTGTTTACCTT
TGGTTTTTATGATATATAATTTTTTTTGGTTTGTTTTTGTTATTTAGTTTTAAGTTTGATTTTGGTT
TAGGTTGTATTAAGATAGTATTACTTATTTTTAGTTTATTTAGTGTGTTATTTTTTGTACACTGGTA
GTTTTTTAGTTGTTTTATTAGCGTTCCAGAATAATCGGCTATGCGTTTTAATTTTTAACTCTATTTG
TTGTGATATCATAGTTTTATTTTTTGTTTTTAAGTTATTTTTTGTAAAATATTTTAATTTTTATTAT
TTTTTGTTGTGGTATCAAAAATTTGTTTTTTGAACTGGATTAGTACCCAGGTAATCAAAATTTATTA
ATTCGGGAGTAAAGTTTTGTTTAAACCGAAAAAATATTGACTGACCTTAGATTTTTCTTTGGAATAT
GTGTTTTGTTGGAGAGCCCTCCTTATTAGTGAATTTTGTTGGCACATGTATGATTGTTTAGTTTTTA
TTTTATTTTGTAATGCTTTTTGTTTTGGCATTTAAAACAGATATATACTTGGCTTATAAATTTATTT
TTCATGTATTACTATTATAAATTTTTTTTGGATTAATTTTTTATTTTTTTTTGAAATTGGAAAAGAA
AGTAATTTTTTTTTAATGTTTTAATGAATTTAATAAATAAGGTGGTACAAACCATCCGTCAATGGCC
TAAAGGGGCGTAAGTTGTAGTATGGTAGAGGTAAGGAAACTTGTTTCTATTTTTTGAAGTTTTTATT
TTTTTTAATTTTATTTTTTGGTATTGCATATCAGTATAGGTTTTTATCATAGTTTATGAATAATAGA
ATTTAAGTGTCAAGACTGGATTATTTTTTTTTTTTACGAAATTAATGATATCCTTGTTTAGTATTGAT
ATAACGTATTTTTATTTCTGTTTTTTTAATTTTTATGAGTTAAGTTATATTTTTTTTGTGTGTGTTT
TAAAATTTTTTTTGTTGTTTTAATGAAGTGTTGTGTGTTTTACATAGATTTTTGTTTTTTTTTTTTT
TTTTTTTGATTAATTTTTTAATTAATTTATAATCTTTGCTTTAGTAATTTTTTTATTTATTTGTGTTT
TTGTTTTTGAATTTTTGTTTTTGAACTGGTTTTGTTGCTAAATGTTTATTAAAAACTTAGGTTTTTA
TATAAAGTTGTCTTCTGCTCTATGATTTTTTTAAGTGGCAGCCTTAGCGTGATGGCATAAAAGTAGC
GTAAGTGATTTGTTTTTTTTAATGTTTTCAAGTATGAATGAAGTTTTTAGCAACTTTTTTGCTTATAT
TTTATTTGAATTATCTTTTTGATTAAAAATTATTGGTTAGAGTATTACAAAGATAAGTCTTCGGAAA
TTTTGTTTTGAATTTTAAAATTTTTAATTTTTAATATTTTCTTGGGGATGGATTTTATGAAATTTTT
ATACTATTATTATTTTTAAAAATTACTCCGGAGTTAACAGGGTTGTAGACATATAAATAGGTTTTTA
TATTAGTGTGCTGCGCTACATCGATGTTGTATATTTTTTTTGATAATGGAGAGGTTTTTTTTGTTTT
GAGACTGTTCTTCTTGTATAAAAAATTGACTTGATATTAGTTTAGTTCGTCGTGAGACAGAGCGGTT
TATCTTGTATATTTTTTGTATTTGATGGTGTTAGTACGAAAGGAACGCAATATGGATTTATATTTAT
GATCTTTTTATTTTGATGGAATTTT

>O.gutturosa(M.Blaxter)

ATTTTTTGTGGAATGACTTTTGGTAATAGTATGAAGCAGAGTATTATTAATACTGTGAATCATAAGA
CTATTGGTACTTATTATATTGTTTTGGGTTATTGAGCTGGTTTGGGTGGTTCTGTTTTGTCTATGTT
GATTCGTTTTGAGTTATCTAGTCCAGGTGGTTATTTGTTTTTTGGTAGAGGTCAGGTTTATAATTCT
GTTCTTACAATACATGGTGTTTTGATGATTTTTTTTTTGGTTATGCCTATTTTGATTGGTGGTTTTG
GTAATTGAATGTTACCTTTGATATTGGGGGCTCCTGAGATGGCTTTTCCTCGGGTTAATGCTTTGTC
TTTTTGGTTTACTTTTGTAGCTTTGTTGATGGTTTATCAGTCTTTTTTTATTGGTGGTGGTCCTGGT
AGAAGTTGGACTTTTTATCCTCCTCTTAGGGTTGAGGGTCAACCGGAGTTGTCTTTGGATACTATAA
TTTTGGGTTTGCATACTGTAGGTATTGGTTCTTTGTTGGGTGCTATTAATTTTATAGTTACTACTCA
GAATATGCGATCTACTGCTGTAACTTTGGATCAGATTAGTATGTTTGTTTGGACTTCTTATTTGACT
TCTTTTTTGTTGGTTTTATCTGTGCCTGTTTTAGCTGGTTCTTTGTTATTTTTGTTGTTGGATCGTA
ATTTTAATACTTCTTTTTATGATACTAAGAAGGGGGGTAACCCTTTGTTGTATCAGCATTTGTTTTG
ATTTTTTGGTCATCCTGAGGTTTATGTTATTATTTTGCCTGTTTTTGGTATTATTAGAGAGGCGGTT
TTGTTTTTGACTGATAAGGACCGTTTGTTTGGTCAAACTAGGATGACTTTTGCTTCTATTTGGATTG
CTGTTTTAGGTACGTCTGTTTGGGGTCATCATATATATACTGCTGGTTTGGATATTGATACTCGTAC
TTATTTTAGTGCAGCTACTATGATTATTGCTATTCCTAGAGCTGTTAAGATTTTTAATTGGTTAGGA
ACTTTTTTTGGTTCTAATCAAAAAATACAGCCTTTGTGGTGTTGGACTTATAGTTTTATTTTTCTTT
TTACTGTAGGTGGGTTGAGTGGAATTATTTTGAGAGCTGCTAGTTTGGATATTATTTTGCATGATAC
TTATTATGTTGTAGCTCATTTTCATTATACTTTGAGTTTGGGTGCAGTTTATGGTATTTTTTGTGGT
TTTTGTTTGTGGCTTCCTTATATGTATGGTATTTCTTTTGATAGGGTTATAATGATGGCTGTTTTTG
TTTGTTTTTTTGTTGGTACTAATATGACGTTTTTTCCTATACATTTTGCTGGTTTGCAGGGTATGCC
TCGTAAGATTTTGGATTATCCTGATTGTTATTCTACTTTTCAGATTGTTTCTTCTTTAGGTTCTGTT
GTTACTTTTGTAGGTTTTGTTTTGTTTAACTATTTGTTGATTGATTCTATTTTTTTTTCTCGTTTTT
TGGGGGTTTCTTTTTATAATTATCATAGTCCGGCTTATGCTTTGAATGTTCCTCCTTTGCCGGATTC
TTTTACTGAGGAGGCTTTTATTATGGGTCTTCATTGGAAGATTATTAGTAAGGATACTCCTTCTTAT
AGATATCGTCGGGTTGGTTATGGTTATCATAGTAAGTAAGTCTTTTTTTATGTTAGGGTGTTTTTTG
CTTTTATTTTTTTTTGTTTAAGGTTTTTAGATTGAGATCCATTGAAGAGTTGTGTTATGATGTGTTT
GGGTATTATGTCTATGAGTTGTTATATTTCTTTGGGTATTCATGTTTGGTATTCTTATTTTATTGTT
TTAATTTTTTTTAGTGGTATTTTTTCTTTGTTGACTTATTTTTGTAGTATGGGTAACTTTGTTTTTT
ATTATAACTATTGTTTTTTTTTTTCTTTGATTTTAGTTAGTATTTTTTTTTTGATTTTTATAGATTT
TGACTTTTTTTTGTTTTTTTCTGATTTTAATTTTTTGTATGTTTGTTACGATTTTAGCTATTATTAT
GTTTTTTGAGTTGTTTTTGTTTTGTTTTTGTTCTTGGTTTTGGTTAGGTTTAGACTTAATGGTTTTG
GTTTTATACGTAGTTTGTAGGTTGTTCTTTTTAATTCTTTAATTTTTTTGCCTGCTAGTTTTACTTT
GAGTTATATGTGAAATTTTGGTAGTATATTGGGTATTATGTTGATATCTCAGATTTTGACTGGTTTT
TTTTTGACTTTTTATTATACAGCTGGTGAGGCTTTTAGGTCTGTTCAATATATTATATTTGAGGTTA
ATTTAGGTTGATTGTTGCGTATTATGCATTCTAATGGGGCTTCTATGTTTTTTTTATTTATTTATTT
GCATATTTTTAAGGGGTTAATTTATGGTAGGTATCGTCTTATTGGTGTGTGATTGAGTGGTATTTTT
ATTTATTTTTTGTTGATGGGTATTGCTTTTACTGGTTATGTTTTAATTTGGGGTCAGATGAGTTATT
GGGCAGCGGTAGTTATTACTAGTTTGATGACTTCTGTTCCTTATTTGGGTAAGTATTTGGTTTGGTG
GATTTGAGGAAGTTTTAGTGTTTGTGAGAACACTCTGAAGTTTTTTTATTCTGTTCATTTTATTTTG
CCTTGGTCTTTAATAGTTTTAATTGTTTTTCATTTGTTTTTTTTGCATTTTACTGGTTCTACTTCTA
GTTTGTATTGTCATGGGGATTATGATAAGGTTCATTTCTTTCCTAGTTTTTGGTTAAAGGATGGTCT
TGATATTTTTTTTTACTTTTTTTTGGTTTTGTTTAGGCTTTATTTTTCTTTTAATTTAAGTGATCCT
ATGATTTTTGTGGAGTCAGATTCTATGGCTAGTCCTGCTCATGTTGTCCCCGAGTGGTATTTTTTGT
TTGCTTTTACTATTTTACGTTCTGTTCCTAGGAAGTTGTTGGGGGTAATTTTGATGTTTAGTTCTGT
TTTTGTTTTAGTTATTCTTATTTGACCTGGTGGTTATCAGTCTATTTTAGATAATTTTTTGTATTTT
TTTGTTATGTGTTTTGTTTGGATTTTTTTTTGGCTAACTTGGGCAGGTCATTATCCTACTGATTATC
CTTTTAATTATTTTAATTTGTTTTGTACTTTTTTTTATTTTTGTTGTGTTTTTTTTATTTGTTTGAC
TAATTTTTTTAGTGATAAGTTGTTTAGTTAAATTTTGTTGAAGTTTCGTAAGTATCATAAAATGGAG
TATAGTTATTATCCTTTGATAGTTGGAGTAGGTGTTTTGGGGTTTGATGTTAGTTTGGTTTTGTTTA
TGGGTATGGGTATGTTTTATTCTATTTTTATTTGTTTTTTGTATTTGGTTTATGTTTTTTTTCTTTG
GGTTAAGGATGTCGTTTTAGAGGATATTAGTGGTCAGTATTCTTTTTATGACTATCGTATGTTTAAC
CAGGGTTTTCGTTTATTTCTTTTTAGTGAATTGACTTTGTTTGTTTCTGTTTTCTGAACTTTTTTGG

ATACTGCTTTGTGTCCTTTGACTTGGTTAGGTGGGGTTTGATCTCCTTTGGGTATTTTGTCTCCTGA
TTATTTGGGTTTGAATGGTATGGCTAGTTTGTTTTTGATGATGAATAGCCAGATTTTGAAGTATTCT
CGTCGTTATTTGTGTTTGAATAACGCTAAGTGTGAGGAGTTTTTGTTGGTTTGTATTTTTATTGGGG
TTGGTTTTTTGTGTTTTCAGTTTTATGAATATAGCAATAATTCTTTTGTTATAAGAGATAGTATTTA
TGGTAGTATTTTTTATATAGGTACTGGTTTACATGGTTTGCATGTTTTTGTTGGTGTTTGTTTTCTT
GTTGTTAATTTTTTTCGTATTAAGTTGTTTAATTTTAATTGATATCATATTCAAGCTTATGATATGT
CTATTGATTATTGGCGTTTTTTAGAATGAATGTGAGGTGTTATGTTTTGTTTGTTATATGTTTGGGG
TTCTTAATTGGTTGTTTTTTATTATTTGGGGTTGCTGGTTATAATTGTTTTTATTTTGCAGGCTATT
GCCTTTTTGACTTTGTTGGAGCGTCATTTTTTGGGCGGCTCTCAGTGTCGTGTTGGTCCCAATAAGG
TTGGTTATTCTGGTGTTTTGCAGGCTTTATTTGATGGTTTGAAGTTGTTGAAAAAGGAACAGTTGTT
GTTGTGTTTTTCTTCTTGGTTGTCTTTTTTGTTTATACCTATTTGTGGTTTTGTTTTGATGGTTTTT
TTTTGGTTTACTTTACCTTATTTTTTTTCTTTTTTGTCCTTTGAGTATTCCGGTGTTTTTTTGTTTT
GTCTTATGGGGGTTTCTGTTTACTTTATTATGCTTTCTGGTGTATTTAGTGGTGGTAAGTATTCTTT
TGTTGGTGGATTGCGTGCTTGTGCTCAGAGTTATTCTTATGAGATTGCTTTTTCTGTTTATTTGTTG
ATTTTTTTGTTGTTTAATAAGGGTCTCTGTTTGTCTTTTAGTTTTTGTTTATTTTTTTTTTTTGTTTT
TTTTTCCTTTTTTTTGCTTAGTTTTAGTTGATTTGCATCGGGCTCCCTTTGATCTTTCTGAATGTGA
AAGTGAATTAGTTAGGGGTTTTAATGTTGAGTATTCGAGTGTTGGTTTTGCCGCCTTGTTTTTGGGT
GAGTATGGTAATTTACTTTATTTTAGTTGTTTGACTTCTAGTTTGTTTTTTGATATAAGTTTTTTTT
TTTTTTATCTTATTGTGTGTTTGGTTATTTTTTCTCGTAGAGCTTATCCTCGTTTTCGTTTTGATAA
ATTGATAAGTGTTTGTTGGTTTTTGTTTTTGCCTGTTGGTTTTTATTTTTTTGGTTTGTCTTTTGTT
GTTTTTATGCTGTATTTTATTGTTTTTTTTTATTTGGTTTTGGTTAGTTTTTTATTTATTTTATATG
GAGTTGAGTAAGTTTAATGGTTTTGGTGTTTTGGGTGGTTTTATTGGTGTTTTAGTTTCTAGTTTTT
CTCATCAGGGTTTTCAGTCTAGTGTATTTTTTAAGTTTGCTGTTTTTTTTTCTTGTGATTTTTTGGAT
GAGTGGGTTGTTGTTTCCTTTGTTTAGTCCTTGGGCTTGTGTGGGGTTTTTGTTTTTTCTTACTAAT
TTTTCTTGGTTAGGTGTTCGTACTTTTACTTTAGCTGTTGATAGTTTTTTGGTTTTTTTTGAGGAGG
ATCATTCTTGGGAGTGGTTTTCTAGATTGGTTATATTTTTTTCTCATTGGTTGAGGTTTTTGATGAG
TGGGGTGGCTTTAACTTTGCGTATTAGTATTATTTTTTTGATTGGGCATTTTTTAATGTTTACTGTC
TTGGATATGAGTGTATTTTGTTCTTTTTTTTTTTTTGTTGTTTGTGGTTCCGGTAGAGTTGTTTTTTG
CTTTTTTGCAAAGTTATATTTTTTTAACTTTGATTTGTATATTTTTACTTAATATGATTTAGATTTA
TTTGCAGAATTATGTGTTTCCTATCCCTGGGAGTTCTTATGTGTTTTGTTGTTACTATATCCATAAT
TACTATTCTCATATTATTTTTTTTTGGTTTTTTTGTAATGTTTTTAGTCAGTAGCGGAGTTTATTTTT
TTGGTAATTCTTTTAAGTTTAATTTAAAGCGTAGTGATAGTCGCATAATTGAGTTGGTTTTGCAGGT
ATTGATTGTTAATTTTTTGATTATGATGGCTGGTCCTGGTTTTTGGTTGATCCAATATCAGGGTCGT
ATGTTTCGTCAATCGGAGTTGGCTTTGAAGGTTATTGGTCATCAGTGGTATTGAAGGTATGAGTATG
GAGATAGTGGAAAGTTGTGTTTTGATTCTTTTATGAAATCTTTGGATGATTTGTCTGTAGGGGATTT
TCGTTTGTTCGATGTTGATAATCGGTGTGTTTTACCAGTAGGTGTGAATGTTGGTGTTTATTGTACT
TCTAGTGATGTTATTCATTCTTTTGCTATTCCTAAGTGTTTTATTAAGATAGATGCTTTGAATGGTT
TATTAACTAAGGTTACTTGTAACTTTTCTTGTTCTGGTTTGTTTTATGGACAATGTTCTGAAATTTG
TGGTGCTAATCATAGGTTTATGCCTATTGTTTTGGAATTAACTTCTTTAGAGTGTTGAAAGGGGTGG
TCTGTTAATTATTTATTGAGTTAACTTTTTTTAAATTTTGTTGTTATTTTTTTTTTTTTCTTTTTTAG
TTCCTTTTGGTATGTATTTTTTTGTCTTTTTTTTGTATCTTTTAAGGATTTTTTTGGTGCTAAGTTGAG
TTCTTATGAATGTGGTTTTGATGTTGTTAGGAAGGTTCATGTTGGTTTTAATTTGGTTTTTTTTTCT
GTTGTTTTGTTATTTGTTGTTTTTGAGTTGGAGGTTTTGATTTTTATTATTTTGATTCAGGGTGATT
TTTATAGTTTGTTGTCTTTTTTTTTGTTTTTTTTTTTATGTTGTTTTTAGGTTTTATATGGAGTGGTA
TTTTGGTAAGTTGATTTGATTTTGTTAGTTGTTGTTGTTTGTCTGATACGTTCTTGTTTTTTACTTT
TTTTTGGCTGTTATTTTTTTTGTTTGTTCCTTATGGGAAGTGGAGTTATAGTTTTGGTTTTAGTGATT
ATTTTAATTTTACTTTTATTTATAGTTTTGAGGTGTGTTTGTTTTTTTTTGGTTTTGTTATTAGTTTC
TTTTATGGTTTTTGTTTATGGTTCTTTTTATATGGTGGGGGTTTCTCGTTTGTTTTATTTTTTTTTT
TTTCTTTTTTTTGTTTGTTTTGAGGATAGGTGGTTTGATTGTTTTTAGTGGTAGTATTATTTTGACTT
TAGTTTTTTGGGATTTTTTGGGGGTTAGTAGTTTTTTTTTGGTTTTGTTTTATGGAAATATTAGTGC
TCGAAGGGGTGCTATGAGTACTGTTTTTACTAATCGTATTGGTGATTTTTGTATTTTTTTTGTTTTTT
AATGGTTTTGTTTTGTTTTCTATGAGTTTTTTATCTTATCAATTTTTTGGTTCTTTGTTAGTTTTTA

TGTTGTTTATTTCTTCTATTGTTAAGGGTGGTCAGTATCCTTTTGGTAGTTGGTTGCCTAAGGCTAT
GGCTGCTCCTACTCCTGTTAGTTGTTTGGTTCATAGTAGTACTTTGGTTACTGCGGGTGTTATGTTG
ATGGATTGCTATGTTTATGTTTCTTTGAATTCTGATGTTTTATCTTTTGTTTTTTATGTTGGTTTTT
TTACTATGGTTTTTTCTGGTTTTTGTGCTTTAGTGGAGGAGGATGCTAAGAAGATTGTTGCTTTGAG
TACTATGTCTCAAATTGGTTTTTGTTTTTTGGCTATTGGTAGTGGGTTGCATTATTTGTCTTATGTA
CATATGATTAGCCATTCTTTTTTTAAGAGTTTGTTGTTTATGCAGATAGGTTACTTGATTTTTATTA
ATTTTGGCCAGCAGGATTATCGTGGTTATTCTTTTTTTAGTTTTTGTGCTCCAATTTTGGTTCAGTT
GCAGATTTTTTTGTCTGTTTTTTGTTTGTGTGGTTTGTTGTTTACTAGTGGGAGTTGTAGTAAGGAG
TATTTTATATCTCGTTTTTATTATAGTTCTTATGGTTTTTTTTTGGTTTTTTTTTATTTTTTTGGGA
TTTTTTTGACTTTTTGTTACTGTTATCGTATGTTGTGTTTGTTTCGTGTAGGTGTTTCTGGTTTTGA
TTATGTTGGTTTTTCTAGTAAGTTGTTTTATTATTCTTGTTTTTCTTTGGTTTTTTTTTCTGTTGTT
TTTACTTTTTGGTGAATTGTTAGTTTGTTGTCTTTTTCTGTAGCTTTTAATCGTTTTGAGTTTTTGG
CTGTTTATTTTTATTTGTTCTTTGTTTATTGTTTTTTTGGTTATTTTTTTCGTTATTTTGTGGTTGA
GCTTAAAAGTAAGTTTTTTATGGATCATTATGCTCGTGTTGTTTATAAGGTTTTTCCTAGTTTTTTT
TATTTTGATGTTTTTATTGTTGGTTTTAATTATTTTTTTTTTTGGATTGGCTCGTTTGTTTTCTTTTT
TTTTTTTTTCTTTGTTTCGAGGTTTTTATCATGTTGGTGTTTTGATAATTTTTTTTTTTATGTTATT
TTTCTTGTTTTTTTAGATTTTGCTGTTTTTTGTTTTTTTTTTGTTGTTGTTTTTGAGTTTTATTAAT
TTTTGTGTTGTTGATTATATTGTTTGGTGGAGGATTTTTGTTATTTGTACTTTTGTTTTTGTTTTTT
TTGTTGGCAGTGAGATAGGGTTTAGAGGTTGTTTGGTTAATTATTATGTTATTCAGGAGGTTTGTGG
GTATTATTTTTTGGTATTTGATGGTTGGAAGTTACAGTTTTTGTTGCTTATGTTGAAGTCGGGTTCT
TCTCCTTTTCATTTTTGGCTTTTTAGTGTTTTGGGTAGTTTGGGTAAGTGGTTTGTTTTGTGGTTTT
TGACTTTGCAAAAGTTGCCTTATTTTGTTGTTTTGGTTAATTTTTGTAGTGATTTTTTTTTTTTGTT
TTTGTTTTTTTGGTATGATTTTTTGTTATTTTCAGTTTTTTTTGTTACGTAGTTATCGTGATTTGTTG
GTTGTAGGTTCTGCTGAGTCTTTTAATTGGTTGTTATTGTTGGGTATTTTTTCTTTTAATGAGGTTT
TTGTTTTGTTTTTTTTTTACTATTTTGTTATGTTTTTTATTGTTTCTTATTTGTATGGAGGTTTTTT
GAATTTTTTAAGTTTGGAGATGTTGATATTTTTTTTTAATGTTCCTTTGAGGATTACTTTTTTTTTG
AAGGTGATTTTGTTGTTTGGTTCTTCTTTTTTTGTTGGTTTTTATTATTTGTTTTTGTTATTGTTTA
TGCCTTTGATGTCTTTGGGTGTGGGTTATTTATTTTTTTTGGTTTCTATGATAAGTTTTAATTGTGG
TTTTAAGTATTATGATTATTTTATTTATGTTTTGTTTTGTGTTGGGTTGTTGTCTTGTTTTTAGTTG
TTGTATTTGTTTTTTTTCGTTGTTTTGTTGTTTTTTTTTAGCCCTTTTTTGTTTTTTATTTTTTTTA
TATTTTTTGTTTTATATGGTTTTTTTGATTACTCTTGATGTGGTTGTTTGTTTTTTTTTGATTCTTT
TAATTTTGTTTTTTTGTCTTTTATGAGTGTTTTTGTTATGGGTTTTATTTGTGTTTCTGAGTTGTTG
AGTGGTTTAGTTTTTTATAGTTGTCTTGTTGTGTTTTTTAGTGTTTGTTTTTTTTACTCTGGTAGTT
TTTTGATGTTGTATGTTTTTTATGAGTTAACTATGGTTCCTATTTTGTTTTGTTTGTTGGGTTATGG
TCGACAGGTGGAGAAGGTTAGTGCTTGTTATTATTTAATTTTTTATACTTTGTTTTTTGGTATACCT
TATTTGTTTTTGTATAGTCATGTTTTTTTTTTTTTTGAATTTTGTTTATTATGATTTTTTTGTTTCTT
ATGAATTTGTTTTTTTTGTTGAGTTTGTGTTTTTTAGTTAAGTTTCCTGTTTACTTTTTTCATGTTTG
ATTGCCTAAGGTTCATGTTGAGGCTCCTACTAGTGCTAGTATGATTTTGGCTGGTGTTATGTTGAAG
TTAGGGGGGGCAGGTGTATATCGTATTAGTAAGTCTTTGAATTTTTTTGGTTTTGAGGTTTTGGTTT
TTTTTTCCTTGATTAGGATGGTGTTTTGTTCTTTTATTTGTGTTGTTCAGAGAGATTGTAAGTCTTT
AGCGGCTTATTCTTCTATTTGTCATATGGGATTTGTGTTGCTTTCTGAGGTTAGTATGGTTTATTAT
GGTAAGTCTATAGCTTTGGTGATGATATTAGCTCATGGTTATACTTCGGTTTTGATGTTTTATTTTA
TTGGTGAGTTTTATCATATTGCTAATAGGCGGTTGATTTATTATTTGCGAGGTTATTTTAATGTTAG
TATGTTGTTTTGTTTGATGTTTTGTTTGACTATGGTTTCTAATTTTAGTTTTCCTGTGTCTATTTCT
TTTTTTTCTGAGTATTTGATATTGAATTTTTTTAGGTCTGTTTTTTATGTTGGTTTTTTTGTTTTTGT
TTTTTTATTATTTGGTTTCTTTTTATTACTCTGTTTATATTTTGGTTTGTTTTTTGATTGGTGATAA
GGTAAGTTTTGTTTGTGATGGTCGTAGTGTTGTTTGTTTGCCTTTAATTTTTATGGTGTATAATTTT
TTTTGATTTGTTTTTGTTATTTATTTTAAGTTTGATTTTGGTTTGGGTTGTATTAAGATAGTATTAC
TTATTTTTAGTTTATTTAGTGTGTTATTTTTTGTACACTGGTAGTTTTTTGATTGTCTTATTAACGT
TCCAGAATAATCGGCTATGCGTTTTAATTTTTGACTCTATTTGTTGTGATGTTATAATTTTTAGTTT
GTTTTTATGTTATTTTTTTGTAAAATGTTTTAGTTTTTTTTTTGTTTTGTTGTGATATCAAAAATTTGT
TTTTTGAACTGGATTAGTACCCAGGTAATCAAAGTTTAATAATTCGGGAGTAAAGTTTTGTTTAAAC

CGAAAAAAATATTGACTGACTTTAGATTTTTCTTTGGAATATGTGATTTGCTGGAGAGCCCTCTTTCT
TGGTGAGTTTTATTGGCACATGTATGATTGTTTAGTTTTTATTTTATTTTGTAATGCTTTTTTGTTT
TGGCATTTAAAACAGATATATATTTGGCTTATGAGTTCATGTTTCATGTGTTACTATTATGAATTTT
TTTTGGATGATTTTTTTATTTTTTTTTGAAATTGGAAAAGAAAGTAATTTTTTCTTTATGTTTTAAT
GAATTTAATGAATAAAGTGGTACAAACCATCCGTCAATGGCCTAAAGGGGCGTAAGTTGTAGTATGG
TAGAGGTAAGGAAACTTGTTTCTATTTTTTGAAGTTTTTTTATTTTTTCTAGGTTTTATTATTTGGT
ATTGCATATCAGTAGAAGTTTTTATCATAGTTATGGGTAATAGAATTAAAATGGTTAAAATTGGATT
TGTTTTTTTTTTACAAAATTAATGATATTTTTATTTAGTATTGATATAACGTATTTTTATTTCTGTT
TTTTATTTTTTATAGGTTAAGTTATATGTTTTTGTTTGTAGATTTTGAAATTTTTATTGTTGTTCGT
AAATGTTGTGTGTTTTACATAGATTTTTATTTTTTTATATAATCCATGTTTGAAAAAATGAGTCATT
AATTTATGATTTTTACTTTAGTAATTTTTTTGTTTTTGAGGTTTTTGTTTTTGAATTTTTGTTTTTG
AACTGGTTTTGTTGTTAAATGTTTATTAAAAACTTAGGTTTTTATATAAAGTTGTCTTCTGCTCTAT
GATTTTTTAAATGGCAGCCTTAGCGTGATGGCATAAAAGTAGCGTAAGTGATTTGTTTTTTTAATGG
TTTCAAGTATGAATGAAGTTTTTAACAACTTTTTTGCTTATTTTTTGTTTGAAATATTTTTTTGATT
AAAAATTATTAGTTAGAGTATTACAAAGATAAGTCTTCGGAAATTTTGTTTTAAATTTTAAAATTTT
TGTTTTTAATTTTTTCTTGGGGATGGATTTTAAGAAAATTTTATACTATTTTTATTTTTAAAAATTA
CTCCGGAGTTAACAGGGTTGTAGACATATAAATAGATTTTTATATTAGTGTGCTGCGCTACATCGAT
GTTGTATATTTTTTTTGATAATAGAGAGGTTTTTTTTGTTTTGAGACTGTTCTTCTTGTATAAAAAA
TTGACTTGATATTAGTTTAGTTCGTCGTGAGACAGAGCGGTTTATCTTGTGTACTTTTGTTTTTTGG
CGGTGTTAGTACGAAAGGAATGCAATGTGGGTTTATATTTATGACTTTTTTATTTTGATGGATTGTT

>O.volvulus(AF015193)
ATTTTTTGTGGAATGACTTTTGGCAATAGTATGAAGCAGAGTATTATTAATACTGTGAATCATAAGA
CTATTGGTACTTATTATATTGTTTTAGGCTATTGGGCTGGTTTAGGTGGTTCTGTTTTATCTATGTT
GATTCGTTTTGAGTTGTCTAGTCCTGGTGGTCATTTGTTTTTTGGAAGTGGTCAGGTTTATAATTCT
GTTCTTACTATGCATGGTGTTTTGATGATTTTTTTTTTAGTTATGCCTATTTTGATTGGTGGTTTTG
GTAATTGGATGTTGCCTTTGATGTTGGGGGCTCCTGAGATGGCGTTTCCTCGGGTAAATGCTTTATC
TTTTTGGTTTACTTTTGTGGCTTTATTGATAGTTTATCAGTCTTTTTTTATTGGGGGTGGTCCTGGT
AGGAGTTGGACTTTTTATCCTCCTCTTAGGGTTGAAGGTCAACCAGAATTGTCTTTAGATACTATGA
TTTTAGGTTTACATACTGTAGGAATTGGTTCTTTGTTGGGTGCTATTAATTTTATGGTAACTACTCA
GAATATACGGTCTACTGCTGTGACTTTGGATCAAATTAGTATGTTTGTTTGGACTTCTTATTTGACT
TCTTTTTTATTAGTTTTATCTGTGCCTGTTTTGGCTGGTTCTTTATTGTTTTTGTTGTTGGATCGTA
ATTTTAATACTTCTTTTTATGATACTAAGAAGGGGGGTAATCCTTTGTTGTATCAGCATTTGTTTTG
ATTTTTTGGTCATCCTGAGGTGTATGTTATTATTTTACCTGTTTTTGGTATTATTAGGGAGGCTGTT
TTATTTTTGACTGATAAGGATCGTTTGTTTGGTCAGACTAGGATGACTTTTGCTTCTATTTGGATTG
CTGTTTTAGGTACTTCTGTATGGGGCCATCATATGTATACGGCTGGTTTGGATATTGATACTCGTAC
TTATTTTAGTGCTGCTACTATGATTATTGCTATTCCTAGGGCTGTTAAGATTTTTAATTGATTGGGT
ACTTTTTTTGGTTCTAATCAAAAGATGCAGCCATTATGATGTTGAACTTATAGTTTTATTTTTCTTT
TTACTGTGGGTGGATTAAGTGGAATTATTTTGAGGGCTGCTAGTTTGGATATTATTTTGCATGATAC
TTATTATGTTGTGGCTCATTTTCATTATACTTTGAGTTTGGGTGCTATTTATGGTATTTTTTGTGGT
TTTTGTTTGTGACTTCCTTATATGTACGGTATTTCTTTTGATAGGGTTATGATAATAGCTGTTTTTG
TTTGTTTTTTTGTTGGTACTAATATAACGTTTTTTCCTATGCATTTTGCTGGTTTACAGGGTATGCC
TCGTAAGATTTTGGATTATCCTGATTGTTATTCTACTTTTCAAATCATTTCTTCTTTAGGTTCTGTT
ATTACTTTTGTTGGTTTTGTTTTGTTTAATTATTTGTTGATTGATTCTATTTTTTTTTTCTCGTTTTT
TGGGAGTTTCTTTTTATAATTATCATAGTCCGGCTTATGCTTTAAATGTTCCTCCTTTGCCGGATTC
TTTTACTGAAGAGGCTTTTATTATAGGTCTCCATTGGAAGATTATTAGTAAGGATACTCCTTCTTAC
AGTTATCGTCGGGTTGGTTATGGTTATCATAGTAAGTAGATTTTTTTTTATGTTAGGGTATTTTTTG
CTTTTGTTTTTTTTTGTTTGAGTTTTTTGGATTGGGATCCGTTGAAAAGTTGTGTTATAATGTGTTT
AGGTGTTATGTCTATAAGTTGTTATGTTTCTTTAGGTGTTCATGTATGGTATTCTTATTTTGTTGTT
TTAATTTTTTTTAGTGGTATTTTTTCTTTGTTGACTTATTTTTGTAGTATGAGTAATTTTATTTTTT
ATTATAATTATTTTTTTTTTTTTTTCTTTGTTTTTGGTTAGTTTTTTTTTTTGTATTTGTTGTAGATTT
TGATTTTTCTTTGTTTTTTTCTGATTTAATTTCTTTATGTTTGTTATGATTTTAGTTATTATTAT

GTTTTTTGAGTAGTTTTTATTTTGTTTTTATTTTTGATTTTGATTAGATTTAGACTTAATGGTTTCG
GTTATATGCGTAGTTTGTAGATTGCTATTTTTAATTCTTTAGTTTTTTTGCCTGCTAGTTTTACTTT
AAGTTATATGTGGAATTTTGGTAGTATGTTAGGCATTATGTTGATGTCTCAGATTTTAACTGGTTTT
TTTTTGACTTTTTATTATACGGCGGGGGAAGCTTTTAGGTCTGTTCAGTATATTATGTTTGAGGTTA
ATTTGGGCTGGTTGCTGCGTATTATGCATTCTAATGGGGCTTCTATGTTTTTTTTATTTATTTATTT
ACATATTTTTAAGGGTTTAATTTATGGTAGATATCGTCTTATTGGTGTGTGATTGAGTGGTATTTTT
ATTTATTTTTTATTAATAGGTATTGCCTTTACTGGTTATGTTTTGATTTGAGGCCAAATAAGTTATT
GGGCGGCAGTAGTTATTACTAGTCTGATGACTTCTGTTCCTTATTTGGGCAAGTATTTGGTTTGATG
GATTTGGGGGAGTTTTAGTGTTTGTGAGAACACTTTGAAGTTTTTTTATTCTGTTCATTTTATTTTA
CCTTGGTCTTTGATAGTTTTAGTTGTTTTTCATTTGTTTTTTTTGCATTTTACTGGTTCTAGTTCTA
GTTTGTATTGTCATGGAGATTATGATAAGATTCATTTTTTTCCTAGTTTTTGATTGAAGGATGGTTT
TGATATTTTTTTTTATTTTTTTTTGATTTTGTTTAGACTTTATTTTTCTTTTGATTTAAGTGATCCT
ATGATTTTTGTGGAGTCTGATTCTATGGCTAGTCCTGCGCATGTTGTACCTGAGTGGTATTTTTTAT
TCGCTTTTACTATTTTACGTTCTGTTCCTAGTAAGTTATTGGGGGTTATTTTAATATTTAGTTCTGT
TTTTGTGTTAACTATTCTTATTTGACCTGGTAGTTATCGTTCTATTTTAGATAATTTTTTATATTTT
TTTGTTATGTGTTTTGTTTGGGTTTTTTTTTGGTTAACTTGAGCTGGTCATTATCCTACTGATTATC
CTTTTAACTATTTTAATTTGTTTTGTACTTTTTTTTTATTTTTGTTGTATTTTTTTTGTTTGTTTGAT
TAATTTTTTTAGTGATAAGTTGTTTAGTTAAATTTTGTTGAAGTTTCGTAAATATCATAAAATGGAG
TATAGTTATTATCCTTTGATAGTTGGGGTGGGTATTTTAGGTTTTGATGTTAGTTTGGTTTTATTTA
TAGGTATAGGTATGTTTTATTCTATTTTTATTTGTTTTTTGTATTTGGTTTATGTTTTTTTTTTGTG
GATTAAGGATGTTATTTTAGAGGATATTAGTGGTCAATATTCTTTCTATGATTATCGTATGTTTAAC
CAAGGTTTTCGTTTGTTTCTTTTTAGTGAATTAACTTTGTTTGTTTCTATTTTTTGAACTTTTTTGG
ATACGGCTTTGTGTCCTTTAACTTGGTTAAGTGGGGTTTGGTCCCCATTGGGGATTTTATCACCTGA
TTATTTGGGTTTGAATGGTATGGCTAGTTTATTTTTAATAATGAATAGGCAAGTTTTGAAGTATTCT
CGTCGTTATTTGTGTTTGAATAGCTCTAAGTGTGAAGAGTTTTTGTTAGTTTGTATTTTTATTGGAG
TTGGTTTTTTGTGTTTTCAGTTTTATGAATATAATAACAATTCGTTTGTTATGAGTGATAGTGCTTA
TGGTAGTATTTTTTATATAGGTACTGGTTTGCATGGTTTGCATGTTTTTGTTGGTGTTTGTTTTCTT
ATTGTTAATTTTTTTCGTGTTAAGTTGTTCAACTTTAATTGATATCATATCCAGGCTTATGATATAT
CTATTGATTATTGGCGTTTTTTAGAGTGAATGTGAGGTGTTATGTTTTGTTTATTATATGTTTGAGG
TTCTTAATTGGTTATTTTTTATTATTTGGGGTTGTTGGTTATGATTGTTTTTATTTTGCAGGCTATT
GCCTTTTTAGTTTTGTTAGAGCGTCATTTTTTGGGTGGTTCCCAGTGTCGTGTTGGTCCTAATAAGG
TGGGTTATTGTGGTGTTTTGCAGGCTTTATTTGATGGCTTAAAGTTGTTAAAGAAGGAGCAGTTGTT
GTTGTGTTTTTCTTCTTGGTTATCTTTTTTGTTTATGCCTATTTGTGGTTTTGTTTTGATGGTTTTT
TTTTGATTTACTTTACCTTATTTTTTTTCTTTTTTGTCTTTTGAATATTCTGGTGTTTTTTTGTTTT
GTCTTATAGGAGTTTCTGTTTATTTTATTATGCTTTCTGGTGTTTTTAGTGGTAGTAAGTATTCTCT
TATTGGTGGGTTGCGTGCTTGTGTTCAGAGTTATTCTTATGAGATTGCTTTTTCTATTTATTTGTTA
GTTTTTTTGTTGTTTAATAAGGGTTTATGTTTGTCTTTTAGTTTTTGTTTATTTTTTTTTTTTGTTTT
TTTTTCCTTTTTTTTGTTTGGTTCTTGTTGATTTGCATCGGGCCCCTTTTGATCTTTCTGAGTGTGA
AAGTGAGCTGGTAAGGGGGTTTAATGTTGAGTACTCTGGAGTTGGTTTTGCTGCTTTGTTTTTAGGG
GAGTATGGTAATTTACTTTATTTTGGTTGTTTGACTTCTAGTTTGTTTTTTGGTATAAGTTTTTTTT
TTTTTTATTTTATTGTGTGCATGATTGTTTTTTCTCGTAGAGCTTATCCTCGTTTTCGTTTTGATAA
GTTGATGGGTGTTTGTTGGTTTTTGTTTTTGCCTATTGGTTTTTATTTTTTTGGTTTGTCTTTTGTT
GTTTTTATGTTGTGCTTATTTAGTTTAATTTTTTAGATTTTGTTATTTTTTTTTTATTTGATTTTGAT
TGGTTTTTTATTTGTTTTATATGGAGTTGAGTAAGTTTAGTAGTTTGGGAATTTTAGGTGTTTTTGT
TAATGTTTTAGTTTCTAGATTTTCTCATCAGGGTTTTCAGTCTAACGTTTTTTTTTAAGTTTGTTGTT
TTTTTTCTCTTGGTTTTTTGAATGAGAGGGTTATTGTTTCCTTTGTTTAGTCCTTGAGCTTGTGTGG
GTTTTTTGTTTTTTATTACTAATTTTTCTTGATTGGGTGTTCGTACTTTTACTTTAGCTGTTGATAG
TTTTTTAATTTTTTTTGAAAGGATCATTCTTGGGAGTGGTTTTCTAGGTTAGTTATGTTTTTTTCT
CATTGGTTAAGATTTTTGATGAGGGGGGTAGCTTTAACTTTGCGTATTAGTATTATTTTTTTAATTG
GTCATTTTCTAATGTTTACTGTTTTGGATATGAGTGTATTTTATTCTTTGTTTTTTTTGTTGTTAGT
TGTTCCGGTGGAGTTGTTTTTTGCTTTTTTACAGAGTTATATTTTTTTGACTTTGGTTTGTATGTTT
TTACTTAATATGATTTAGATTTATTTGCAGAATTATGTGTTTCCTATTCCCGGAAATTCTTATGTAT

TTTGTTGTTATTATATTCATAATTACTATTCTCACATTATTTTTTTTGGTTTTTTTGTAATGTTTTT
GGTTAGTGGCGGGATTTATTTTTTTGGTAATTCTTTTAAGTTTAATTTGAAGCGTAGAGATAGTCGT
ATAATTGAGTTAATTTTACAGGTGTTAATTGTTAATTTTTTAATTATGATGGCTGGTCCTGGTTTTT
GGTTGATTCAATATCAGGGACGTATATTTCGTCAATCTGAGTTGACTTTGAAGGTTATTGGTCACCA
ATGGTATTGAAGTTATGAATATGGAGATAGTGGAAAATTATGTTTTGATTCATTTATGAAGTCTTTA
GATGATTTGTCTTTAGGGGATTTTCGGTTATTTGATGTTGATAATCGGTGTGTTTTGCCTGTAGGTG
TAAATGTTGGTGTGTATTGTACTTCTAGTGATGTTATTCATTCTTTTGCGATTCCTAAGTGTTTTAT
TAAGATGGATGCTTTAAATGGTTTGTTAACTAAGGTTACTTGTAGTTTTTCTTGTTCTGGTTTGTTT
TTTGGGCAGTGTTCTGAAATTTGTGGTGCTAATCATAGGTTTATGCCTATTGTGTTGGAGTTGACTT
CTTTGGAGTGTTGGAAGGGTTGATCAGTTAATTATTTATTGGGTTAACTTTTTTTAAATTTTATTGT
TATTTTTTTTTTTTTCTTTTTTGGTTCCTTTTGGTATGTATTTGTTGTCTTTTTTTGTGTCTTTTAAG
GATTTTTATGGTGCTAAATTAAGTTCTTATGAGTGTGGTTTTGATGTTGTGAAGAAGGTTCATGTTG
GTTTTAATTTGGTTTTTTTTTCTATTGTTTTGTTATTTGTTGTTTTTGAGTTGGAAGTTTTGATTTT
TATTATTTTGATTCAGGGTGATTTTTATAGTTTGTTGTCTTTTTTTGTTTTTTTTTTTTATGTTGTT
TTTAGTTTTTATATGGAGTGGTGTTTTGGTAAGTTGATTTGGTTTTGTTAGTTGTTATTGTTTGTTT
GATATGTTCTTATTTTTTATTTTTTTTTGGTTGTTATTTTTTTGTTTGTTCCTTATGGTAAGTGGAG
TTATAGTTTTGGTTTTAGTGATTATTTTAATTTTACTTTTATTTATAATTTTGAGGTTTGTTTGTTT
TTTTTAGTTTTGTTGTTGGTTTCTTTTATGGTTTTTGTTTATGGTTCTTTTTATATGGTTGGGGTTT
CTCGTTTGTTTTATTTTTTTTTTTTTTTATTTTTGTTTGTGTTGAGGATGGGTGGTTTGATTGTTTT
TAGGGGTAGTATTGTTTTAACTTTGGTTTTTTGAGATTTTTTGGGGGTTAGTAGTTTTTTTTTGGTT
TTGTTTTATGGTAATGTTAGTGCTCGGAGGGGTGCTATGAGTACTGTGTTTACTAATCGTATTGGTG
ATTTTTGTATTTTTTTGTTTTTTAATGGTTTTGTTTTGTTTTCTATGAGTTTTTTGTCTTATCAGTT
TTTTGGTTCTTTGTTAGTTTTTATGTTGTTTGTTTCTTCTATTATTAAGGGTGGTCAGTATCCTTTT
GGTAGTTGGTTGCCTAAGGCTATGGCGGCTCCTACTCCTGTTAGTTGTTTGGTTCATAGTAGTACTT
TAGTTACTGCTGGTGTTATGTTGATGGATTGTTATGTTTATGTTTCTTTGAATTCTGATGTTTTGTC
TTTTGTTTTTTATGTTGGTTTTTTTACTATGGTTTTTTCTGGTTTTTGTGCTTTGGTAGAGGAGGAT
GCTAAGAAGATTGTTGCTTTGAGTACTATGTCTCAAATTGGTTTTTGTTTTTTGGCTATTGGTAGGG
GTTTGCATTATTTGTCTTATGTTCATATGATTAGACATTCTTTTTTTAAGAGGTTATTGTTTATGCA
GATGGGTTATTTGATTTTTATTAATTTTGGTCAACAGGATTATCGTGGTTATTCTTTTTTTGGTTTT
TGTGCTCCGGTTTTGGTTCAGTTGCAGATTTTTTTATCTGTGTTTTGTTTGTGTGGTTTGTTGTTTA
CTAGGGGTAGTTGTAGTAAGGAATATTTTATGTCTCGTTTTTATTATGATTCTTATGGTTTTTTTTT
AGTTTTTTTTTATTTTTTTGGTGTATTTTTGACTTTTTGTTATTGTTATCGGATGTTTTTTTTGTTT
CGTGTGGGAGCTTTTGGTTTTGATTATGTGGGTTTTTCTAGTAAGTTGTTTTATTTTTCTTGTTTTT
TTTTGGTTTTTTTTTCTGTTGTTTTTACTTTTTGGTGGGTTTTTAGTTTATTGTCTTTTTCTGTGGC
TTTTAATCGTTTTGAGTTTTTGGTTGTTTATTTTTATTTGTTTTTTGTTTATTGTTTTTGTGGTTAT
TTTTTTCGTTATTTTGTGGTTGAGTTTAAGGGTAAATTTTTTATAGACCATTATGCTTGTTTTATTT
ATAAGATTTTTCCTAGTTTTTTTTATTTTGATGTTTTTATTATGGGTTTTAATTATTTTTTTTTTGG
TTTATTTCGGTTATTTTCTTTTTTTTTTTTTTCTTGGTTTCGGGGGTTTTATCATGTTGGTGTTTTA
ATTGTTTTTTTTTTTTATGTTGTTTTTTTTGTTTTTTTAGATTTTGTTATTTTTTGTTTTTTTTTTAT
TATTGTTTTTGAGTTTTATTAATTTTTGTGTTGTTGATTATATTGTTTGGTGGAGGATTTTTGTTAT
TTGTACTTTTGTTTTTGTTTTTCTTGTTGGTGGTGAGTTGGGATTTAGAAGTTGTTTGGTTAATTAT
TATGTTATTCAAGAAGTTTGTGGTTATTATTTTTTGGTTTTTGATGGTTGGAAGTTGCAATTTTTAT
TGCTTATGTTGAAGTCAGGTTCTTCTCCTTTTCATTTTTGACTTTTTAGTGTTTTGGGTGGTTTGAA
TAAGTGGTTTGTTTTGTGGTTTTTAACTTTGCAAAAATTGCCTTATTTTGTTGTTTTGGTTAATTTT
TGTGGTGATTTTTTTTTTTTTGTTTTTGTTTTTTGGTATGATTTTTTGTTATTTTCAATTTTTTTTGT
TGCGTAGTTATCGTGATTTGCTGGTTGTGGCTCTGCTGAATCTTTTAATTGGTTGTTATTATTGGG
TATTTTTTTCTTTTAATGAAGTGTTTGTTTTGTTTTTTTTTTTATTATTTTGTTATATTTTTTGTTATT
TCTTATGTGTATGGGGGATTTTTGGGTTTTTTGAGTTTAGAGATATTGATGTTTTTTTTTAATGTTC
CTTTGAGGATTACTTTTTTTTTTGAAGGTTATTGTGTTGTTTGGTTCTTCTTTTTTTGTTGGTTTTTA
TTATTTATTTTTGTTGTTGTTTATGCCTTTGATGTCTTTGGGTGTGGGTTATTTGTTTTTTTTTGGTT
TCTATGATGAGTTTTAATTGTGGTTTTAAGTATTATGATTATTTTGTTTATGTTTTGTTTTGTATTG
GGCTGTTGTCTTGTTTTTAGTTGTTGTATTTGTTTTTTTTTTGTTGTTTTGTTGTTTTTTTTTAGTCC

TTTTTTATTTTTTGTTTTTTTTATGTTTTTTGTTTTATATGGGTTTTTTGATTATTCCTGGTTTGGT
TGTTTGTTTTTTTTTGATTCTTTTAATTTTGTTTTTTTGTCTTTTATGAGTGTTTTTGTTATGGGGT
TTATTTGTGTATCTGAATTGTTGAGTGGTTTAGTTTTTTATAGTTGCCTTGTAGTGTTTTTTAGTGT
TTGTTTTTTTTATTCTGGTAGTTTTTTGATATTGTATGTTTTTTATGAGTTGACTATAGTACCTATT
TTGTTTTGTTTGTTAGGGTATGGTCGTCAGGTGGAGAAGATTAGGGCTTGTTATTATTTAATTTTTT
ATACTTTATTTTTTGGGATGCCTTATTTGTTTTTGTATAGTCATGTTTTTTTTTTTTTGAATTTTGT
TTATTATGATTTTTTTGTTTCTTATGAATTTATTTTTTTGTTAAGTTTGTGTTTTTTGGTAAAGTTC
CCTGTTTATTTTTTTCATGTTTGATTGCCTAAGGTTCATGTTGAGGCCCCCACTAGTGCTAGAATGA
TTTTGGCTGGTGTTATGTTGAAGTTGGGGGGAGCAGGTGTTTATCGTATTAGTAAGTCTTTAAATTT
TTTTGGTTTTGAAATGTTGATTTTTTTTTCTTTAATTAGGATGGTTTTTTGTTCTTTTATTTGTGTT
GTTCAGAGGGATTGTAAGTCTTTGGCTGCCTATTCTTCTGTTTGTCATATGGGTTTTGTGTTGCTTT
CTGAGATTAGTATGGTTTATTATGGTAAGTCTATAGCTTTGGTGATGATGTTGGCTCATGGTTATAC
TTCTGTTTTGATGTTTTATTTTATTGGTGAGTTTTATCATATTGCTAATAGGCGTTTAATTTATTAT
TTGCGTGGATATTTTAATGTTAGTATGTTGTTTTGTTTGATGTTTTGTTTGACTATGGTTTCTAATT
TTAGTTTTCCTGTGTCTGTTTCTTTTTTTTTCTGAGTATTTGATGTTGAATTTTTTTAGATCTGTTTT
TTATGTTGGTTTTTTGTTTTTGTTTTTTTATTATTTGGTTTCTTTTTATTATTCTGTTTATATTTTG
GTTTGTTTTTTGGTCGGCGGTAAGGTGAGTTATGTTTGTGATGGTCGTAGTGTAGTTTGTTTACCTT
TGGTTTTTATGATATATAATTTTTTTTGGTTTATTTTTGTTATTTAATTTTAAGTTTGATTTTGGTT
TAGGTTGTATTAAGATAGTATTACTTATTTTTAGTTTACTTAGTGTGTTAATTTTTGTACACTGGTA
GTTTTTTGATTGTTTTATTAACGTTCCAGAATAATCGGCTATGCGTTTTAATTTTTGACTCTATTTG
TTGTGGTGCTATGAGTTTTTAGTTTGTTTTTATATTGTTTTTTGTAAAATATTTTAATTTTTTTTTT
AGTTTCTTATGGTATCAAAAATTTGTTTTTTGAACTGGATTAGTACCCAGGTAATCAAAATTTAATA
ATTCGGGAGTAAAGTTTTATTTAAACCGAAAAAATATTGACTGACTTTAGATTTTTCTTTGGAATAT
GTGATTTGCTGGAGAGCCCTCTTTTTTGGTGAATTTTGTTGGCACATGTATGATTGTTTAGTTTTTA
TTTTATTTTGTAATGCTTTATTGTTTTGGCATTAAAAACAGATATATATTTGGCTTATGAATTTATG
TTTCATGTGTTACTATTATGAATTTTTTTTGGATTAGTTTTTTATTTTTTTTTGAAATTGGAAAAGA
AAGTAATTTTTTCTTAATGTTTTAATGAATTTAATAAATAAGGTGGTACAAACCATCCGTCAATGGC
CTAAAGGGGCGTAAGTTGTAGTATGGTAGAAGTAAGGAAACTTGTTTCTATTTTTTGAAGTTTTTTT
GTTTTTTAAGTTTTATTATTTGGTATTGCATATCAGTAGAAGTTTTTATCATAGCTATGAGTAATAG
AATTAAAATGGTTAAATTGAATTTGTTTCTTTTTTACGAAATTAATAATATTTTTATTTAGTATTGA
TATAACGTATTTTTTATTTCTGTTTATTTGTTATTTATAGGTTAAGTTATATTATTTTTTTGTTTGTAA
ATTTTTTAAATTTTTGTTGTTGTTTTATAAATGTTGTGTGTTTTACATAGATTTTTATTTTTTTTTT
TTTTTTTTTTTTTTGATTAATTTTTTAATTGATTTATGATTTTTACTTTAGTAATTTTTATTGTTTT
GATAGGTTTGTTTTTGAATTTGTGTTTTTGAACTGGTTTTGTTGCTAAATGTTTATTAAAAACTTAG
GTTTTTATGTAAAATTGTCTTCTGCTCTATGAGTTTTTAAATGGCAGCCTTAGCGTGATGGCATAAA
AGTAGCGTAAGTGATTTGTTTTTTTAATGGTTTCAAGTATGAATGAAGTTTTTAGCAGCTTTTTTAT
TTACTTTTTGTTTGAATTATTTTTTTAATTAAAAATTATTAGTTAAGGTATTACAAAGATAAGTCTT
CGGAAATTTTGTTTTGAATTTTGAAATTTTTATTTTTAATTTTTTCTTGGGGATGGATTTTAAGAAA
GTTTTATACTATTGTTATTATTAAAAATTACTCCGGAGTTAACAGGGCTGTAGACATATAAATAGGT
TTTTATATTAGTGTGCTGCGCTACATCGATGTTGTATATTTTTTTTGATAATGGAGAGGTTTTTTTT
GTTTTGAGACTGTTCTTCTTGTATAAAAAATTGACTTGATATTAGTTTAGTTCGTCGTGAGACAGAG
CGGTTTATCTTGTGTATTTTTGGTGCTTGGCGGTGTTAGTACGAAAGGAATGCAATGTGGGTTTATA
TTTATGACTTTTTTATTTTGATGGATTTTT

>O.ochengiM2
ATTTTTTGTGGAATGACTTTTGGTAATGGTATGAAGCAGAGTATTATTAATACTGTGAATCATAAGA
CTATTGGTACTTATTATATTGTTTTAGGTTATTGGGCTGGTTTAGGTGGTTCTGTTTTATCTATGTT
GATTCGTTTTGAATTGTCTAGTCCTGGTGGTTATTTGTTTTTTGGAAGTGGTCAGGTTTATAATTCT
GTTCTTACTATGCATGGTGTTTTGATGATTTTTTTTTTGGTTATGCCTATTTTGATTGGTGGCTTTG
GTAATTGGATGTTGCCTTTAATATTAGGGGCTCCTGAGATGGCGTTTCCTCGGGTAAATGCTTTATC
TTTTTGATTTACTTTTGTGGCTTTGTTGATAGTTTATCAGTCTTTTTTTATTGGGGGTGGCCCTGGT
AGAAGTTGGACTTTTTATCCTCCTCTTAGGGTTGAAGGTCAACCAGAATTGTCTTTAGATACTATGA

TTTTAGGTTTACATACTGTNGGAATTGGTTCTTTGTTGGGTGCTATTAATTTTATGGTAACTACTCA
GAATATACGGTCTACTGCTGTGACTTTGGATCAAATTAGTATGTTTGTTTGGACTTCTTATTTGACT
TCTTTTTTGTTAGTTTTGTCTGTGCCTGTTTTGGCTGGTTCTTTATTGTTTTTGTTGTTGGATCGTA
ATTTTAATACTTCTTTTTATGATACTAAGAAGGGGGGTAATCCTTTGTTGTATCAGCATTTGTTTTG
ATTTTTTGGTCATCCTGAGGTGTATGTTATTATTTTACCTGTTTTTGGTATTATTAGGGAAGCGGTT
TTATTTTTGACTGATAAGGATCGTTTGTTTGGTCAGACTAGGATAACTTTTGCTTCTATTTGGATTG
CTGTTTTAGGTACTTCTGTGTGAGGTCATCATATGTATACGGCTGGTTTGGATATTGATACTCGTAC
TTATTTTAGTGCTGCTACTATGATTATCGCTATTCCTAGAGCTGTTAAGATTTTTAATTGGTTAGGT
ACTTTTTTTGGTTCTAGTCAAAAGGTGCAGCCGTTATGATGTTGAACTTATAGTTTTATTTTTCTTT
TTACTGTGGGTGGATTAAGTGGAATTATTCTGAGGGCTGCTAGTTTGGATATTATTTTGCACGATAC
TTATTATGTTGTGGCTCATTTTCATTATACTTTGAGTTTGGGTGCTATTTATGGTATTTTTTTGTGGT
TTTTGTTTGTGACTTCCTTATATGTATGGTATTTCTTTTGATAGGGTTATGATAATAGCTGTTTTTG
TTTGTTTTTTTGTTGGTACTAATATGACATTTTTTCCTATGCATTTTGCTGGTTTGCAGGGTATGCC
TCGTAAGATTTTGGATTATCCTGATTGTTATTCTACTTTTCAGATTATTTCTTCTTTAGGTTCTGTT
ATTACTTTTGTTGGTTTTGTTTTGTTTAATTATTTGTTGGTTGATTCTATTTTTTTTTTCTCGTTTTT
TGGGGGTTTCTTTTTATAATTATCATAGTCCGGCTTATGCTTTAAATGTTCCTCCTTTGCCGGATTC
TTTTACTGAAGAGGCTTTTATTATAGGTCTTCATTGGAAGATTATTAGTAAGNATACTCCTTCTTAT
AGGTATCGTCGGGTTGGTTATGGTTATCATAGTAAGTAAATTTTTTTTTATGTTAGGGTATTTTTTG
CTTTTGTTTTTTTTTGTTTGAGTTTTTTGGATTGGATCCGTTGAAGAGTTGTGTTATGATGTGTTT
GGGTATTATGTCTATAAGTTGTTATGTTTCTTTGGGTATTCATGTATGATATTCTTATTTTGTTGTT
TTAATTTTTTTTAGTGGTATTTTTTCTTTGTTGACTTATTTTTGTAGTATGAGTAATTTTGTTTTTT
ATTATAATTATTTTTTTTTTTTTTCTTTGTTTTTGGTTAGTTTTTTTTTTGTATTTGTTGTAGATTT
TGATTTTTTTTTGTTTTTTTGTGATTTTAATTTTCTTTATGTTTGTTATGATTTTAGTTATTATTAT
GTTTTTTGAGTAGTTTTTGTTTTGTTTTTGTTTTTGGTTTTGGTTAGATTTAGGTTTAATGGTTTTG
GTTATATGCGTAGNTTGTAGATTGTTATTTTTAATTCTTTAGTTTTTTTGCCTGCTAGTTTTACTTT
GAGTTATATGTGAAATTTTGGTAGTATGTTGGGTATTATGTTGATGTCTCAGATTTTAACTGGTTTT
TTTTTGACTTTTTACTATACGGCTGGGGAAGCTTTTAGGTCTGTTCAGTATATTATGTTTGAGGTTA
ATTTGGGTTGGTTGTTGCGTATTATGCATTCTAATGGGGCTTCTATGTTTTTTTTGTTTATTTATTT
ACATATTTTTAAGGGTCTGATTTATGGTAGATATCGTCTTATTGGTGTATGGTTGAGTGGTATTTTT
ATTTATTTTTTATTGATAGGTATTGCTTTTACTGGTTATGTTTTGATTTGAGGTCAAATAAGTTATT
GGGCGGCAGTAGTTATTACTAGTTTAATAACTTCTGTTCCTTATTTAGGTAAGTATTTAGTTTGATG
GATTTGGGGGAGTTTTAGTGTTTGTGAGAACACTTTAAAGTTTTTTTATTCTGTTCATTTTATTTTA
CCTTGGTCTTTGATAGTTTTAGTTGTTTTTCATTTGTTTTTTTTGCATTTTACTGGTTCTAGTTCTA
GTTTGTATTGTCACGGGGATTATGATAAGATTCATTTTTTTCCTAGTTTTTGATTGAAGGATGGTTT
TGATATTTTTTTTTATTTTTTTTTTGATTTTGTTTAGACTTTATTTTTCTTTTGATTTAAGTGATCCT
ATGATTTTTGTGGAGTCTGATTCTATGGCTAGTCCTGCGCATGTTGTACCTGAGTGATATTTTTTAT
TTGCTTTTACTATCTTACGTTCTGTTCCTAGTAAGTTATTAGGGGTTATTTTAATATTTAGTTCTGT
TTTTGTATTGACTATTCTTGTTTGACCTGATAGTTATCAGTCTATTTTGGATAATTTTTTATATTTT
TTTGTTATGTGTTTTGTTTGGATTTTTTTTTGGTTAACTTGAGCTGGTCATTATCCTACTGATTATC
CTTTTAACTATTTTAATTTGTTTTGTACTTTTTTTTATTTTTGTTGTATTTTTTTTGTTTGTTTAAT
TAATTTTTTTAGTGNTAAGTTGTTTAGTTAAATTTTGTTGAAGTTTCGTAAGTACCATAAAATGGAG
TATAGTTATTATCCTTTGATAGTTGGGGCGGGTATTTTAGGTTTTGATNTTAGTTTGGTTTTATTTA
TAAGTATAGGTATGTTTTATTCTATTTTTATTTGTTTTTTGTATTTGGTTTATGTTTTTTTTTTGTG
AATCAAGGATGTTATTTTAGAGGACATTAGTGGTCAATATTCTTTTTATGACTATCGTATGTTTAAT
CAAGGTTTTCGTTTGTTTCTTTTTAGTGAGTTAACTTTGTTTGTTTCTATTTTTTGAACTTTTTTGG
ATACAGCTTTGTGTCCTTTAACTTGGTTAGGTGGGGTTTGGTCTCCATTTGGGATTTTATCTCCTGA
TTATTTGGGTTTGAANGGTATGGCTAGTTTGTTTTTAATGATAAATAGGCAAGTTTTGAAGTATTCT
CGTCGTTATTTGTGTTTGAGTAGTTCTAAGTGTGAAGAGTTTTTGTTAGTTTGTATTTTTGTTGGAG
TTGGTTTTTTATGTTTTCAGTTTTATGAATATAATAACAATTCGTTTGTTATGAGTGATAGTGTTTA
TGGTAGTATTTTTTATATGGGTACTGGTTTACATGGTTTGCATGTTTTTATTGGTGTTTGTTTTCTT
ATTGTTAATTTTTTTCGTGTTAAGTTGTTCAATTTTAATTGATATCATGTTCAGGCTTATGATATAT
CTATTGATTATTGGCGTTTTTTAGAATGAATGTGAGGTGTTATGTTTTGTTTATTATATGTTTGAGG

TTCTTAATTGGTTATTTTTTATTATTTGGGGTTGTTGGTTATGATTGTTTTTATTTTGCAGGCTATT
GCTTTTTTAACTTTGTTGGAGCGNCATTTTTTGGGTGGTTCTCAGTGTCGTGTTGGTCCTAATAAGG
TGGGTTATTCTGGTGTTTTGCAGGCTTTATTTGATGGTTTAAAGTTGTTAAAGAAGGAGCAGTTGTT
GTTGTGTTTTTCTTCTTGATTATCTTTTTTGTTTATGCCTGTTTGTGGTTTTGTTTTGATGGTTTTT
TTTTGATTTACTTTGCCTTATTTTTTTTCTTTTTTGTCTTTTGAGTACTCTGGTGTTTTTTTGTTTT
GNCTTATAGGGGTTTCTGTTTATTTTATTATGCTTTCTGGTGTTTTTAGCGGTAGTAAATATTCTTT
TGTTGGTGGATTGCGTGCTTGCGTTCAGAGTTATTCTTANGAGATTGCTTTTTCTATTTATTTGTTG
GTTTTTTTGTTGTTTAATAAGGGTCTATGTTTGTCTTTNAGTTTTTGTTTATTTTTTTTTTTGTTTT
TTTTTCCTTTTTTTTGTTTAGTTCTTGTTGATTTGCATCGGGCTCCTTTTGATTTTTCTGAGTGTGA
AAGTGAGTTGGTAAGGGGGTTTAATGTTGAGTATTCGGGAGTTGGTTTTGCTGCTTTGTTTTTAGGG
GAGTATGGTAATTTACTTTATTTTGGTTGTTTGACTTCTAGTTTGTTTTTTGGTATAAGTTTTTTTT
TTTTTTATTTTATTGTATGTATGATTGTTTTTTCTCGTAGAGCTTATCCTCGTTTTCGTTTTGATAA
GTTGATGGGTGTTTGTTGGTTTTTGTTTTTGCCTGTCGGTTTTTATTTTTTTGGTTTGTCTTTTGTT
GTTTTTATGTTGTGCTTATTTAGTTTAATTTTTTAGATTTGTTATTTTTTTTTATTTGGTTTTGAT
TAGTTTTTTATTTGTTTTATATGGAGTTGAGTAAGTTTAGTAGTTTGGGAGTTTTAGGTGTTTTTGT
TNATGTTTTAGTTTCTGGATTTTCTCATCAGGGTTTTCAGTCTAGTGTTTTTTTTAAGTTTGTTGTT
TTTTTTCTTTTGGTTTTTTGAATGAGTGGGTTATTGTTTCCTTTGTTTAGTCCTTGGGCTTGTGTGG
GTTTTTTGTTTTTTGTTACTAATTTTTCTTGATTGGGTGTTCGTACTTTTATTTTAGCTGTAGATAG
TTTTTTGATTTTTTTTGAGGGGGATCACTCTTGGGAGTGGTTTTCTAGGTTAGTTATGTTTTTTTCT
CATTGGTTGAGATTTTTGATAAGGGGGGTGGCTTTAACTTTGCGTATTAGTATTATTTTTTTAATTG
GTCATTTTTTAATGTTTACTGTTTTGGATATGAGTGTATTTTATTCTTTGTTTTTTTTGTTGTTTGT
GGTTCCGGTGGAGTTGTTTTTTGCTTTTTTACAGAGTTATATTTTTTTGACTTTGGTTTGTATGTTT
TTACTTAATATGATTTAGATTTATTTGCAGAATTATGTGTTTCCTATTCCTGGAAATTCTTATGTGT
TTTGTTGTTATTATATCCATAATTATTATTCTCATATTATTTTTTTTGGTTTTTTTGTGATGTTTTT
GGTTAGTGGTGGGGTTTATTTTTTTGGCAATTCTTTTAAGTTTAACTTGAAGCGGAGAGATAGTCGT
ATAATTGAATTAGTTTTACAGGTGTTGATTGTTAATTTTTTGATTATGATGGCAGGTCCTGGTTTTT
GGTTGATTCAGTATCAGGGACGTATGTTTCGTCAATCTGAGTTGGCTTTGAAGGTTATTGGTCATCA
ATGGTATTGGAGTTATGAGTATGGTGATAGTGGAAAATTATGTTTTGATTCATTTATGAAGTCTTTA
GATGATTTGTCTTTAGGGGATTTTCGGTTATTTGATGTTGATAATCGGTGTGTTTTGCCTGTAGGTG
TGAATGTTGGAGTGTATTGTACTTCTAGTGATGTTATTCATTCTTTTGCNATTCCTAAGTGTTTTAT
TAAGATGGATGCTTTGAATGGTTTGTTAACTAAGGTTACTTGTAATTTTTCTTGTTCTGGTTTGTTT
TTTGGGCAGTGTTCTGAAATTTGTGGTGCTAATCATAGGTTTATGCCTATTGTGTTGGAGTTGACTT
CTTTGGAGTGTTGGAAGGGTTGATCAGTTAATTATTTGCTGGGTTAACTTTTTTTAAATTTTATTGT
TATTTTTTTTTTTTCTTTTTTGGTTCCTTTTGGTATGTATTTGTTGTCTTTTTTTGTGTCTTTTAAG
GATTTTTATGGTGCTAAATTAAGTTCTTATGAATGTGGTTTTGATGTTGTGAAGAAGGTTCATGTTG
GTTTTAATTTGGTTTTTTTTTCTATTGTTTTGTTGTTTGTTGTTTTTGAGTTGGAAGTTTTAATTTT
TATTATTTTGATTCAGGGTGATTTTTATAGTTTATTGTCTTTTTTTTGTTTTTTTTTTTATGTTGTT
TTTAGTTTTTATATGGAGTGGTATTTTGGTAAGTTGATTTGGTTTTGTTAGTTGTTATTGTTTGTTT
GATATGTTCTTATTTTTTATTTTTTTTTGGTTGTTGTTTTTTTGTTTGTTCCTTATGGTAAATGGAG
TTATAGTTTTGGTTTTAGTGATTATTTTAATTTACTTTTGTTTATAATTTTGAAGTTTGTTTGTTT
TTTTTAGTTTTGTTGTTGGTTTCTTTTATGGTTTTGTTTATGGTTCTTTTTATATGGTTGGGGTTT
CTCGTTTGTTTTATTTTTTTTTTTTTTTATTTTTGTTTGTGTTGAGGATGGGTGGCTTGATTGTTTT
TAGAGGTAGTATTGTTTTAACTTTGGTTTTTTGGGATTTTTTGGGGGTTAGTAGTTTTTTTTTGGTT
TTGTTTTATGGTAATGTTAGTGCTCGAAGGGGTGCTATGAGTACTGTGTTTACTAATCGTATTGGTG
ATTTTTGTATTTTTTTGTTTTTTAATGGTTTTGTTTTGTTTTCTATGAGTTTTTTGTCTTATCAGTT
TTTTGGTTCTTTGTTAGTTTTTATGTTGTTTGTTTCTTCTGTTATTAAGGGTGGTCAGTATCCTTTT
GGTAGCTGGTTGCCTAAGGCTATGGCTGCTCCTACTCCTGTTAGCTGTTTGGTTCATAGTAGTACTT
TAGTTACTGCTGGTGTTATGTTGATGGATTGTTATGTTTATATTTCTTTGAATTCTGATGTTTTGTC
TTTTGTTTTTTATGTTGGTTTTTTTACTATAGTTTTTTCTGGTTTTTGTGCTTTGGTGGAGGAGGAT
GCTAAGAAGATTGTTGCTTTGAGTACTATGTCTCAGATTGGTTTTTGTTTTTTGGCTATTGGTAGGG
GTTTGCATTATTTGTCTTATGTTCATATGATTAGGCATTCTTTTTTTAAGAGATTGTTGTTTATGCA
GATAGGTTATTTGATTTTTATTAATTTTGGTCAACAGGATTATCGTGGTTATTCTTTTTTTGGTTTT

TGTGCTCCGGTTTTAGTTCAGTTGCAGATTTTTTTATCTGTGTTTTGTTTGTGTGGTTTGTTGTTTA
CTAGAGGTAGTTGTAGTAAGGAATATTTTATATCTCGTTTTTATTATGATTCTTATGGTTTTTTTT
AGTTTTTTTTTATTTTTTTGGTGTGTTTTTGACTTTTTGTTATTGTTATCGGATGTTTTTTTTGTTT
CGTGTGGGGGCTTTTGGTTTTGATTATGTGGGTTTTTCTAGTAAGTTANTTTATTTTTCTTGTTTT
TTTTTGGTTTTTTTTTCTGTTGTTTTTACTTTTTGGTGGGTTTTTGGTTTGTTATCTTTTTCTGCGG
CTTTTAATCGTTTTGAGTTTTTGGTTGTTTATTTTTATTTGTTTTTTGTTTATTGTTTTTGTGGTTA
TTTTTTTCGTTATTTTTTTTTCGTTATTTTGTGAAATTTTTTATGGATCATTATGCTTGTTTTATTT
ATAAAATTTTTCCTAGTTTTTTTTATTTTGATGTTTTTATTATGGGTTTTAATTATTTTTTTTTTGG
GTTATTTCGGTTATTTTCTTTTTTTTTTTTTTCTTGGTTTCGGGGGTTTTATCATGTTGGTGTTTTA
ATTGTTTTTTTTTTTATGTTGTTTTTTTTGTTTTTTTAGATTTTGTTATTTTTTGTTTTTTTTTTAT
TATTGTTTTTGAGTTTTATTAATTTTTGTGTTGTTGATTATATTGTTTGGTGGAGGATTTTTGTTAT
TTGTACTTTTGTTTTTGTTTTTTTTGTTGGTGGTGAGTTAGGTTTTGGGGGTTATTTGGTTAATTAN
TATGTTATTCAGGAAGTTTGTGGTTATTATTTTTTGGTTTTTGATGGTTGGAAGTTGCAATTTTTAT
TGCTTATGTTGAAGTCTGGTTCTTCTCCTTTTCATTTTTGACTTTTTAGTGTTTTGGGTGGTTTGGA
TAAGTGGTTTATTTTGTGGTTTTTAACTTTGCAAAAATTGCCTTATTTTGTTGTTTTGGTTAATTTT
TGTGGTGATTTTTTTTTTTTGTTTTTGTTTTTTGGTATAATTTTTTGTTATTTTCAATTTTTTTTGT
TGCGTAGTTATCGTGATTTGTTAGTTGTGGGTTCTGCTGAATCTTTTAATTGGTTATTGTTATTGGG
TATTTTTTCTTTTAATGAAGTATTTGTTTTGTTTTTTTTTTATTATTTTGTTATGTTTTTTGTTGTC
TCTTATGTGTATGGGGGATTTTTAAATTTTTTGAGTTTAGAGATATTGATGTTTTTTTTTAATGTTC
CTTTGAGAATTACTTTTTTTTTAAAGGTGATTGTGTTGTTTGGTTCTTCTTTTTTTGTTGGTTTTTA
TTATTTATTTTTGTTGTTGTTTATGCCTTTGATGTCTTTGGGTATGGGTTATTTGTTTTTTTTGGTT
TCGATGATGAGTTTTAATTATGGTTTTAAGTATTATGATTATTTTGTTTATGTTTTGTTTTGTGTTG
GGTTGTTGTCTTGTTTTTAGTTGTTGTATTTATTTTTTTTTGTTGTTTTGTTGTTTTTTTTTAGTCC
TTTTTTATTTTTTGTTTTTTTTATGTTTTTTGTTTTATATGGTTTTTTTGATTGTTCATGGTTTGGT
TGTTTGTTTTTTTTTGATTCTTTTAATTTTGTTTTTTTGTCTTTTATGAGTGTTTTTGTTATGGGGT
TTATTTGTGTGTCTGAATTGTTGAGTGGCTTAGTTTTTTATAGTTGTCTTGTAGTGTTTTTTAGTGT
TTGTTTTTTTTATTCTGGTAGTTTTTTGATATTGTATGTTTTTTATGAGTTGACTATAGTACCTATT
TTGTTTTGTTTGTTAGGGTATGGTCGTCAGGTGGAGAAGGTTAGGGCTTGTTATTATTTAATTTTTT
ATACTTTGTTTTTTGGGATGCCTTATTTGTTTTTGTATAGTCATGTTTTTTTTTTTTTTGAATTTTGT
TTATTATGATTTTTTTGTTTCTTATGAATTTATTTTTTTGTTGAGTTTGTGTTTTTTAGTTAAGTTT
CCTGTTTATTTTTTTCATGTTTGATTACCTAAGGTTCATGTTGAGGCNCCTACTAGTGCTAGAATGA
TTTTGGCTGGTGTTATGTTGAAGTTGGGNGGAGCAGGTGTTTATCGTATTAGTAAGTCTTTGAATTT
TTTTGGTTTTGAAATGTTGATTTTTTTTTTCTTTGATTAGGATGGTTTTTTGTTCTTTTATTTGTGTT
GTTCAGAGTGATTGTAAGTCTTTGGCGGCTTATTCTTCTGTTTGTCATATGGGTTTTGTATTGCTCT
CTGAGATTAGTATGGTTTATTATGGTAAGTCTATGGCTTTGGTGATGATGTTGGCTCATGGTTATAC
TTCTGTTTTAATGTTTTATTTTATTGGTGAGTTTTATCATATTGCTAATAGGCGTTTAATTTATTAT
TTGCGTGGATATTTTAATGTTAGTATGTTGTTTTGTTTGATGTTTTGTTTGACTATGGTTTCTAATT
TTAGTTTTCCTGTATCTATTTCTTTTTTTTTCTGAGTATTTGATGTTGAATTTTTTTAGGTCTGTTTT
TTATGTTGGTTTTTTTGTTTTTGTTTTTTTATTATTTGGTTTCTTTTTATTATTCTGTTTATATTTTG
GTTTGTTTTTTAGTTGGGGATAAGGTGAGTTATGTTTGTGATGGTCGTAGTGTTGTTTGTTTACCTT
TGGTTTTTATGATATATAATTTTTTTTTGGTTTATTTTTGTTATTTAATTTTAAGTTTGATTTTGGTT
TAGGTTGTATTAAGATAGTATTACTTATTTTTAGTTTATTTAATGTGTTATTTTTTGTACACTGGTA
GTTTTTTGATTGTTTTATTAACGTTCCAGAATAATCGGCTATGCGTTTTAATTTTTGACTCTATTTG
TTGTGGTGCTATGAGTTTTTAGTTTGTTTTTATGTTGTTTTTTGTAAAATATTTTGATTTTTTTTAG
TTTCTTGTGGTATCAAAAATTTGTTTTTTGAACTGGATTAGTACCCAGGTAATCAAAATTTAATAAT
TCGGGAGTAAAGTTTTGTTTAAACCGAAAAAATATTGACTGACTTTAGATTTTTCTTTGGAACATGT
GATTTGCTGGAGAGCCCTCTTTTTTTGGTGAATTTTGTTGGCACATGTATGATTGTTTAGTTTTTATT
TTATTTTGTAATGCTTTGTTGTTTTGGCATTAAAAACAGATATATATTTGGCTTATGAATTTATGTT
TCATGTGTTACTATTATGAATTTTTTTTGGATTAGTTTTTTATTTTTTTTTTGAAATTGGAAAAGAAA
GTAATTTTTTTTTAATGTNTTAATGAATTTAATAAATAAGGTGGTACAAACCATCCGTCAATGGCCT
AAAGGGGCGTAAGTTGTAGTATGGTAGAAGTAAGGAAACTTGTTTCTATTTTTTGAAGTTTTTTTGT
TTTTTTAAGTTTTATTATTTGGTATTGCATATCAGTAGAAGTTTTTATCATAGTTATGAGTAATAGA

ATTAAAATGGTTAAATTGAATTTGTTTTTTTTTTACGAAATTAATAATATTTTTATTTTAGTATTGA
TATAACGTATTTTTATTTCTGTTTATTTGTTATTTATAGGTTAAGTTATACTGTTTTTGTTTGTAGA
TTTTTAAATTTTTGTTGCTGTTTTATAAATGTTGTGTGTTTTACATAGATTTTTATTTTTTTTTTTT
TTTTTTTTTTGATTAATTTTTTAATTAATTTATGATTTTTACTTTAGTAATTTTTATTATTTTGAGG
GGTGTTTGTTTTTGAATTTATGTTTTTGAACTGGTTTTGTTGCTAAATGTTTATTAAAAACTTAGGT
TTTTATGTAAAATTGTCTTCTGCTCTATGAGTTTTTAAATGGCAGCCTTAGCGTGATGGCGTAAAAG
TAGCGTAAGTGATTTGTTTTTTTAATGGTTTCAAGTATGAATGAAGTTTTTAGCAGTTTTTTTATTT
ACTTTTTATTTGAATTATTTTTTTGATTAAAAATTATTAGTTAAGGTATTACAAAGATAAGTCTTCG
GAAATTTTGTTTTGAATTTTGAAATTTTTGTTTTTAATTTTTTCTTGGGGATGGATTTTAAGAAAGT
TTTATACTATTGTTATTATTAAAAATTACTCCGGAGTTAACAGGGTTGTAGACATATAAATAGATTT
TTATATTAGTGTGCTGCGCTACATCGATGTTGTATATTTTTTTTGATAATGGAGAGGTTTTTTTTAT
TTTGAGACTGTTCTTCTTGTATAAAAAAATTGACTTGATATTAGTTTAGTTCGTCGTGAGACAGAGCG
GTTTATCTTGTGTATTTTTTGGTTTTTGGCGGTGTTAGTACGAAAGGAATGCAATGTGGGTTTATATT
TATGACTTTTTTATTTTGATGGGTTTTT

>O.ochengiF1
ATTTTTTGTGGAATGACTTTTGGTAATGGTATGAAGCAGAGTATTATTAATACTGTGAATCATAAGA
CTATTGGTACTTATTATATTGTTTTAGGTTATTGGGCTGGTTTAGGTGGTTCTGTTTTATCTATGTT
GATTCGTTTTGAATTGTCTAGTCCTGGTGGTTATTTGTTTTTTGGAAGTGGTCAGGTTTATAATTCT
GTTCTTACTATGCATGGTGTTTTGATGATTTTTTTTTTGGTTATGCCTATTTTGATTGGTGGCTTTG
GTAATTGGATGTTGCCTTTAATATTAGGGGCTCCTGAGATGGCGTTTCCTCGGGTAAATGCTTTATC
TTTTTGATTTACTTTTGTGGCTTTGTTGATAGTTTATCAGTCTTTTTTTATTGGGGGTGGCCCTGGT
AGAAGTTGGACTTTTTATCCTCCTCTTAGGGTTGAAGGCCAACCAGAATTGTCTTTAGATACTATGA
TTTTAGGTTTACATACTGTAGGAATTGGTTCTTTGTTGGGTGCTATTAATTTTATGGTAACTACTCA
GAATATACGGTCTACTGCTGTGACTTTGGATCAAATTAGTATGTTTGTTTGGACTTCTTATTTGACT
TCTTTTTTGTTAGTTTTGTCTGTGCCTGTTTTGGCTGGTTCTTTATTGTTTTTGTTGTTGGATCGTA
ATTTTAATACTTCTTTTTATGATACTAAGAAGGGGGGTAATCCTTTGTTGTATCAGCATTTGTTTTG
ATTTTTTGGTCATCCTGAGGTGTATGTTATTATTTTACCTGTTTTTGGTATTATTAGGGAAGCGGTT
TTATTTTTGACTGATAAGGATCGTTTGTTTGGTCAGACTAGGATAACTTTTGCTTCTATTTGGATTG
CTGTTTTAGGTACTTCTGTGTGAGGTCATCATATGTATACGGCTGGTTTGGATATTGATACTCGTAC
TTATTTTAGTGCTGCTACTATGATTATCGCTATTCCTAGAGCTGTTAAGATTTTTAATTGGTTAGGT
ACTTTTTTTGGTTCTAGTCAAAAGGTGCAGCCGTTATGATGTTGAACTTATAGTTTTATTTTTCTTT
TTACTGTGGGTGGATTAAGTGGAATTATTCTGAGGGCTGCTAGTTTGGATATTATTTTGCACGATAC
TTATTATGTTGTGGCTCATTTTCATTATACTTTGAGTTTGGGTGCTATTTATGGTATTTTTTGTGGT
TTTTGTTTGTGACTTCCTTATATGTATGGTATTTCTTTTGATAGGGTTATGATAATAGCTGTTTTTG
TTTGTTTTTTTGTTGGTACTAATATGACATTTTTTCCTATGCATTTTGCTGGTTTGCAGGGTATGCC
TCGTAAGATTTTGGATTATCCTGATTGTTATTCTACTTTTCAGATTATTTCTTCTTTAGGTTCTGTT
ATTACTTTTGTTGGTTTTGTTTTGTTTAATTATTTGTTGGTTGATTCTATTTTTTTTTTCTCGTTTTT
TGGGGGTTTCTTTTTATAATTATCATAGTCCGGCTTATGCTTTAAATGTTCCTCCTTTGCCGGATTC
TTTTACTGAAGAGGCTTTTATTATAGGTCTTCATTGGAAGATTATTAGTAAGGATACTCCTTCTTAT
AGGTATCGTCGGGTTGGTTATGGTTATCATAGTAAGTAAATTTTTTTTTTATGTTAGGGTATTTTTTG
CTTTTGTTTTTTTTTGTTTGAGTTTTTTGGATTGGGATCCGTTGAAGAGTTGTGTTATGATGTGTTT
GGGTATTATGTCTATAAGTTGTTATGTTTCTTTGGGTATTCATGTATGATATTCTTATTTTGTTGTT
TTAATTTTTTTTAGTGGTATTTTTTCTTTGTTGACTTATTTTTGTAGTATGAGTAATTTTGTTTTTT
ATTATAATTATTTTTTTTTTTTTTTCTTTGTTTTTGGTTAGTTTTTTTTTTGTATTTGTTGTAGATTT
TGATTTTTTTTTGTTTTTTTGTGATTTTAATTTTCTTTATGTTTGTTATGATTTTAGTTATTATTAT
GTTTTTTGAGTAGTTTTTGTTTTGTTTTTGTTTTTGGTTTTGGTTAGATTTAGGTTTAATGGTTTTG
GTTATATGCGTAGCTTGTAGATTGTTATTTTTAATTCTTTAGTTTTTTTGCCTGCTAGTTTTACTTT
GAGTTATATGTGAAATTTTGGTAGTATGTTGGGTATTATGTTGATGTCTCAGATTTTAACTGGTTTT
TTTTTGACTTTTTACTATACGGCTGGGGAAGCTTTTAGGTCTGTTCAGTATATTATGTTTGAGGTTA
ATTTGGGTTGGTTGTTGCGTATTATGCATTCTAATGGGGCTTCTATGTTTTTTTTTGTTTATTTATTT
ACATATTTTTAAGGGTCTGATTTATGGTAGATATCGTCTTATTGGTGTATGGTTGAGTGGTATTTTT

ATTTATTTTTTATTGATAGGTATTGCTTTTACTGGTTATGTTTTGATTTGAGGTCAAATAAGTTATT
GGGCGGCAGTAGTTATTACTAGTTTAATAACTTCTGTTCCTTATTTAGGTAAGTATTTAGTTTGATG
GATTTGGGGGAGTTTTAGTGTTTGTGAGAACACTTTAAAGTTTTTTTATTCTGTTCATTTTATTTTA
CCTTGGTCTTTGATAGTTTTAGTTGTTTTTCATTTGTTTTTTTTGCATTTTACTGGTTCTAGTTCTA
GTTTGTATTGTCACGGGGATTATGATAAGATTCATTTTTTTCCTAGTTTTTGATTGAAGGATGGTTT
TGATATTTTTTTTTTATTTTTTTTTGATTTTGTTTAGACTTTATTTTTCTTTTGATTTAAGTGATCCT
ATGATTTTTGTGGAGTCTGATTCTATGGCTAGTCCTGCGCATGTTGTACCTGAGTGATATTTTTTAT
TTGCTTTTACTATCTTACGTTCTGTTCCTAGTAAGTTATTAGGGGTTATTTTAATATTTAGTTCTGT
TTTTGTATTGACTATTCTTGTTTGACCTGATAGTTATCAGTCTATTTTGGATAATTTTTTATATTTT
TTTGTTATGTGTTTTGTTTGGATTTTTTTTTGGTTAACTTGAGCTGGTCATTATCCTACTGATTATC
CTTTTAACTATTTTAATTTGTTTTGTACTTTTTTTTTATTTTTGTTGTATTTTTTTTGTTTGTTTAAT
TAATTTTTTTTAGTGATAAGTTGTTTAGTTAAATTTTGTTGAAGTTTCGTAAGTACCATAAAATGGAG
TATAGTTATTATCCTTTGATAGTTGGGGCGGGTATTTTAGGTTTTGATGTTAGTTTGGTTTTATTTA
TAAGTATAGGTATGTTTTATTCTATTTTTATTTGTTTTTTGTATTTGGTTTATGTTTTTTTTTTGTG
AATCAAGGATGTTATTTTAGAGGACATTAGTGGTCAATATTCTTTTTATGACTATCGTATGTTTAAT
CAAGGTTTTCGTTTGTTTCTTTTTAGTGAGTTAACTTTGTTTGTTTCTATTTTTTGAACTTTTTTGG
ATACAGCTTTGTGTCCTTTAACTTGGTTAGGTGGGGTTTGGTCTCCATTTGGGATTTTATCTCCTGA
TTATTTGGGTTTGAATGGTATGGCTAGTTTGTTTTTAATGATAAATAGGCAAGTTTTGAAGTATTCT
CGTCGTTATTTGTGTTTGAGTAGTTCTAAGTGTGAAGAGTTTTTGTTAGTTTGTATTTTTGTTGGAG
TTGGTTTTTTATGTTTTCAGTTTTATGAATATAATAACAATTCGTTTGTTATGAGTGATAGTGTTTA
TGGTAGTATTTTTTTATATGGGTACTGGTTTACATGGTTTGCATGTTTTTATTGGTGTTTGTTTTCTT
ATTGTTAATTTTTTTCGTGTTAAGTTGTTCAATTTTAATTGATATCATGTTCAGGCTTATGATATAT
CTATTGATTATTGGCGTTTTTTAGAATGAATGTGAGGTGTTATGTTTTGTTTATTATATGTTTGAGG
TTCTTAATTGGTTATTTTTTATTATTTGGGGTTGTTGGTTATGATTGTTTTTATTTTGCAGGCTATT
GCTTTTTTAACTTTGTTGGAGCGCCATTTTTTGGGTGGTTCTCAGTGTCGTGTTGGTCCTAATAAGG
TGGGTTATTCTGGTGTTTTGCAGGCTTTATTTGATGGTTTAAAGTTGTTAAAGAAGGAGCAGTTGTT
GTTGTGTTTTTCTTCTTGATTATCTTTTTTGTTTATGCCTGTTTGTGGTTTTGTTTTGATGGTTTTT
TTTTGATTTACTTTGCCTTATTTTTTTTTCTTTTTTGTCTTTTGAGTACTCTGGTGTTTTTTTGTTTT
GTCTTATAGGGGTTTCTGTTTATTTTATTATGCTTTCTGGTGTTTTTAGCGGTAGTAAATATTCTTT
TGTTGGTGGATTGCGTGCTTGCGTTCAGAGTTATTCTTATGAGATTGCTTTTTCTATTTATTTGTTG
GTTTTTTTGTTGTTTAATAAGGGTCTATGTTTGTCTTTTAGTTTTTGTTTATTTTTTTTTTTTGTTTT
TTTTTCCTTTTTTTTGTTTAGTTCTTGTTGATTTGCATCGGGCTCCTTTTGATTTTTCTGAGTGTGA
AAGTGAGTTGGTAAGGGGGTTTAATGTTGAGTATTCGGGAGTTGGTTTTGCTGCTTTGTTTTTAGGG
GAGTATGGTAATTTACTTTATTTTGGTTGTTTGACTTCTAGTTTGTTTTTTGGTATAAGTTTTTTTT
TTTTTTATTTTATTGTATGTATGATTGTTTTTTCTCGTAGAGCTTATCCTCGTTTTCGTTTTGATAA
GTTGATGGGTGTTTGTTGGTTTTTGTTTTTGCCTGTCGGTTTTTATTTTTTTGGTTTGTCTTTTGTT
GTTTTTATGTTGTGCTTATTTAGTTTAATTTTTTAGATTTTGTTATTTTTTTTTTATTTGGTTTTGAT
TAGTTTTTTATTTGTTTTATATGGAGTTGAGTAAGTTTAGTAGTTTGGGAGTTTTAGGTGTTTTTGT
TAATGTTTTAGTTTCTGGGTTTTCTCATCAGGGTTTTCAGTCTAGTGTTTTTTTTTAAGTTTGTTGTT
TTTTTTCTTTTGGTTTTTTGAATGAGTGGGTTATTGTTTCCTTTGTTTAGTCCTTGGGCTTGTGTGG
GTTTTTTGTTTTTTGTTACTAATTTTTCTTGATTGGGTGTTCGTACTTTTATTTTAGCTGTAGATAG
TTTTTTGATTTTTTTTGAGGGGGATCACTCTTGGGAGTGGTTTTCTAGGTTAGTTATGTTTTTTTCT
CATTGGTTGAGATTTTTGATAAGGGGGGTGGCTTTAACTTTGCGTATTAGTATTATTTTTTTAATTG
GTCATTTTTTAATGTTTACTGTTTTGGATATGAGTGTATTTTATTCTTTGTTTTTTTTGTTGTTTGT
GGTTCCGGTGGAGTTGTTTTTTGCTTTTTTACAAAGTTATATTTTTTTGACTTTGGTTTGTATGTTT
TTACTTAATATGATTTAGATTTATTTGCAGAATTATGTGTTTCCTATTCCTGGAAATTCTTATGTGT
TTTGTTGTTATTATATCCATAATTATTATTCTCATATTATTTTTTTTGGTTTTTTTGTGATGTTTTT
GGTTAGTGGTGGGGTTTATTTTTTTGGCAATTCTTTTAAGTTTAACTTGAAGCGGAGAGATAGTCGT
ATAATTGAATTAGTTTTACAGGTGTTGATTGTTAATTTTTTGATTATGATGGCAGGTCCTGGTTTTT
GGTTGATTCAGTATCAGGGACGTATGTTTCGTCAATCTGAGTTGGCTTTGAAGGTTATTGGTCATCA
ATGGTATTGGAGTTATGAGTATGGTGATAGTGGAAAATTATGTTTTGATTCATTTATGAAGTCTTTA
GATGATTTGTCTTTAGGGGATTTTCGGTTATTTGATGTTGATAATCGGTGTGTTTTGCCTGTAGGTG

TGAATGTTGGAGTGTATTGTACTTCTAGTGATGTTATTCATTCTTTTGCTATTCCTAAGTGTTTTAT
TAAGATGGATGCTTTGAATGGTTTGTTAACTAAGGTTACTTGTAATTTTTCTTGTTCTGGTTTGTTT
TTTGGGCAGTGTTCTGAAATTTGTGGTGCTAATCATAGGTTTATGCCTATTGTGTTGGAGTTGACTT
CTTTGGAGTGTTGGAAGGGTTGATCAGTTAATTATTTGCTGGGTTAACTTTTTTTAAATTTTATTGT
TATTTTTTTTTTTTCTTTTTTGGTTCCTTTTGGTATGTATTTGTTGTCTTTTTTTGTGTCTTTTAAG
GATTTTTATGGTGCTAAATTAAGTTCTTATGAATGTGGTTTTGATGTTGTGAAGAAGGTTCATGTTG
GTTTTAATTTGGTTTTTTTTTCTATTGTTTTGTTGTTTGTTGTTTTTGAGTTGGAAGTTTTAATTTT
TATTATTTTGATTCAGGGTGATTTTTATAGTTTATTGTCTTTTTTTTGTTTTTTTTTTTATGTTGTT
TTTAGTTTTTATATGGAGTGGTATTTTGGTAAGTTGATTTGGTTTTGTTAGTTGTTATTGTTTGTTT
GATATGTTCTTATTTTTTATTTTTTTTTGGTTGTTGTTTTTTTGTTTGTTCCTTATGGTAAATGGAG
TTATAGTTTTGGTTTTAGTGATTATTTTAATTTTACTTTTGTTTATAATTTTGAAGTTTGTTTGTTT
TTTTTAGTTTTGTTGTTGGTTTCTTTTATGGTTTTTGTTTATGGTTCTTTTTATATGGTTGGGGTTT
CTCGTTTGTTTTATTTTTTTTTTTTTTTTATTTTTGTTTGTGTTGAGGATGGGTGGCTTGATTGTTTT
TAGAGGTAGTATTGTTTTAACTTTGGTTTTTTGGGATTTTTTGGGGGTTAGTAGTTTTTTTTTGGTT
TTGTTTTATGGTAATGTTAGTGCTCGAAGGGGTGCTATGAGTACTGTGTTTACTAATCGTATTGGTG
ATTTTTGTATTTTTTTTGTTTTTTAATGGTTTTGTTTTGTTTTCTATGAGTTTTTTGTCTTATCAGTT
TTTTGGTTCTTTGTTAGTTTTTATGTTGTTTGTTTCTTCTGTTATTAAGGGTGGTCAGTATCCTTTT
GGTAGCTGGTTGCCTAAGGCTATGGCTGCTCCTACTCCTGTTAGCTGTTTGGTTCATAGTAGTACTT
TAGTTACTGCTGGTGTTATGTTGATGGATTGTTATGTTTATATTTCTTTGAATTCTGATGTTTTGTC
TTTTGTTTTTTATGTTGGTTTTTTTACTATAGTTTTTTCTGGTTTTTGTGCTTTGGTGGAGGAGGAT
GCTAAGAAGATTGTTGCTTTGAGTACTATGTCTCAGATTGGTTTTTGTTTTTTGGCTATTGGTAGGG
GTTTGCATTATTTGTCTTATGTTCATATGATTAGGCATTCTTTTTTTAAGAGATTGTTGTTTATGCA
GATAGGTTATTTGATTTTTATTAATTTTGGTCAACAGGATTATCGTGGTTATTCTTTTTTTTGGTTTT
TGTGCTCCGGTTTTAGTTCAGTTGCAGATTTTTTTATCTGTGTTTTGTTTGTGTGGTTTGTTGTTTA
CTAGAGGTAGTTGTAGTAAGGAATATTTTATATCTCGTTTTTATTATGATTCTTATGGTTTTTTTTT
AGTTTTTTTTTATTTTTTTTGGTGTGTTTTTGACTTTTTGTTATTGTTATCGGATGTTTTTTTTTGTTT
CGTGTGGGGGCTTTTGGTTTTGATTATGTGGGTTTTTCTAGTAAGTTANTTTTATTTTTCTTGTTTT
TTTTTGGTTTTTTTTTCTGTTGTTTTTACTTTTTGGTGGGTTTTTGGTTTGTTATCTTTTTCTGCGG
CTTTTAATCGTTTTGAGTTTTTGGTTGTTTATTTTTATTTGTTTTTTGTTTATTGTTTTTGTGGTTA
TTTTTTTCGTTATTTTTTTTTCGTTATTTTGTGAAATTTTTTATGGATCATTATGCTTGTTTTATTT
ATAAAATTTTTCCTAGTTTTTTTTTATTTTGATGTTTTTATTATGGGTTTTAATTATTTTTTTTTTGG
GTTATTTCGGTTATTTTCTTTTTTTTTTTTTTTCTTGGTTTCGGGGGTTTTATCATGTTGGTGTTTTA
ATTGTTTTTTTTTTTTATGTTGTTTTTTTTTGTTTTTTTAGATTTTGTTATTTTTTGTTTTTTTTTTAT
TATTGTTTTTGAGTTTTATTAATTTTTGTGTTGTTGATTATATTGTTTGGTGGAGGATTTTTGTTAT
TTGTACTTTTGTTTTTGTTTTTTTTTGTTGGTGGTGAGTTAGGTTTTGGGGGTTATTTGGTTAATTAT
TATGTTATTCAGGAAGTTTGTGGTTATTATTTTTTGGTTTTTGATGGTTGGAAGTTGCAATTTTTAT
TGCTTATGTTGAAGTCTGGTTCTTCTCCTTTTCATTTTTGACTTTTTAGTGTTTTGGGTGGTTTGGA
TAAGTGGTTTATTTTGTGGTTTTTAACTTTGCAAAAATTGCCTTATTTTGTTGTTTTGGTTAATTTT
TGTGGTGATTTTTTTTTTTTTGTTTTTGTTTTTTGGTATAATTTTTTGTTATTTTCAATTTTTTTTGT
TGCGTAGTTATCGTGATTTGTTAGTTGTGGGTTCTGCTGAATCTTTTAATTGGTTATTGTTATTGGG
TATTTTTTCTTTTAATGAAGTATTTGTTTTGTTTTTTTTTTTATTATTTTGTTATGTTTTTTGTTGTC
TCTTATGTGTATGGGGGATTTTTAAATTTTTTGAGTTTAGAGATATTGATGTTTTTTTTTAATGTTC
CTTTGAGAATTACTTTTTTTTTTAAAGGTGATTGTGTTGTTTGGTTCTTCTTTTTTTGTTGGTTTTTA
TTATTTATTTTTGTTGTTGTTTATACCTTTGATGTCTTTGGGTATGGGTTATTTGTTTTTTTTTGGTT
TCGATGATGAGTTTTAATTATGGTTTTAAGTATTATGATTATTTTGTTTATGTTTTGTTTTGTGTTG
GGTTGTTGTCTTGTTTTTAGTTGTTGTATTTATTTTTTTTTTGTTGTTTTGTTGTTTTTTTTTAGTCC
TTTTTTATTTTTTGTTTTTTTTTATGTTTTTTGTTTTATATGGTTTTTTTGATTGTTCATGGTTTGGT
TGTTTGTTTTTTTTTGATTCTTTTAATTTTGTTTTTTTGTCTTTTATGAGTGTTTTTGTTATGGGGT
TTATTTGTGTGTCTGAATTGTTGAGTGGCTTAGTTTTTTATAGTTGTCTTGTAGTGTTTTTTAGTGT
TTGTTTTTTTTATTCTGGTAGTTTTTTGATATTGTATGTTTTTTATGAGTTGACTATAGTACCTATT
TTGTTTTGTTTGTTAGGGTATGGTCGTCAGGTGGAGAAGGTTAGGGCTTGTTATTATTTAATTTTTT
ATACTTTGTTTTTTGGGATGCCTTACTTGTTTTTGTATAGTCATGTTTTTTTTTTTTTTGAATTTTGT

TTATTATGATTTTTTTGTTTCTTATGAATTTATTTTTTTGTTGAGTTTGTGTTTTTTAGTTAAGTTT
CCTGTTTATTTTTTTCATGTTTGATTACCTAAGGTTCATGTTGAGGCTCCTACTAGTGCTAGAATGA
TTTTGGCTGGTGTTATGTTGAAGTTGGGAGGAGCAGGTGTTTATCGTATTAGTAAGTCTTTGAATTT
TTTTGGTTTTGAAATGTTGATTTTTTTTTCTTTGATTAGGATGGTTTTTTGTTCTTTTATTTGTGTT
GTTCAGAGTGATTGTAAGTCTTTGGCGGCTTATTCTTCTGTTTGTCATATGGGTTTTGTATTGCTCT
CTGAGATTAGTATGGTTTATTATGGTAAGTCTATGGCTTTGGTGATGATGTTGGCTCATGGTTATAC
TTCTGTTTTAATGTTTTATTTTATTGGTGAGTTTTATCATATTGCTAATAGGCGTTTAATTTATTAT
TTGCGTGGATATTTTAATGTTAGTATGTTGTTTTGTTTGATGTTTTGTTTGACTATGGTTTCTAATT
TTAGTTTTCCTGTATCTATTTCTTTTTTTTCTGAGTATTTGATGTTGAATTTTTTTAGGTCTGTTTT
TTATGTTGGTTTTTTGTTTTTGTTTTTTTATTATTTGGTTTCTTTTTATTATTCTGTTTATATTTTG
GTTTGTTTTTTAGTTGGGGATAAGGTGAGTTATGTTTGTGATGGTTGTAGTGTTGTTTGTTTACCTT
TGGTTTTTATGATATATAATTTTTTTTGGTTTATTTTTGTTATTTAATTTTAAGTTTGATTTTGGTT
TAGGTTGTATTAAGATAGTATTACTTATTTTTAGTTTATTTAATGTGTTATTTTTTGTACACTGGTA
GTTTTTTGATTGTTTTATTAACGTTCCAGAATAATCGGCTATGCGTTTTAATTTTTGACTCTATTTG
TTGTGGTGCTATGAGTTTTTAGTTTGTTTTTATGTTGTTTTTTGTAAAATATTTTGATTTTTTTTAG
TTTCTTGTGGTATCAAAAATTTGTTTTTTGAACTGGATTAGTACCCAGGTAATCAAAATTTAATAAT
TCGGGAGTAAAGTTTTGTTTAAACCGAAAAAATATTGACTGACTTTAGATTTTTCTTTGGAACATGT
GATTTGCTGGAGAGCCCTCTTTTTTGGTGAATTTTGTTGGCACATGTATGATTGTTTAGTTTTTATT
TTATTTTGTAATGCTTTGTTGTTTTGGCATTAAAAACAGATATATATTTGGCTTATGAATTTATGTT
TCATGTGTTACTATTATGAATTTTTTTTGGATTAGTTTTTTATTTTTTTTTGAAATTGGAAAAGAAA
GTAATTTTTTTTTAATGTTTTAATGAATTTAATAAATAAGGTGGTACAAACCATCCGTCAATGGCCT
AAAGGGGCGTAAGTTGTAGTATGGTAGAAGTAAGGAAACTTGTTTCTATTTTTTGAAGTTTTTTTGT
TTTTTTAAGTTTTATTATTTGGTATTGCATATCAGTAGAAGTTTTTATCATAGTTATGAGTAATAGA
ATTAAAATGGTTAAATTGAATTTGTTTTTTTTTTACGAAATTAATAATATTTTTATTTTAGTATTGA
TATAACGTATTTTTATTTCTGTTTATTTGTTATTTATAGGTTAAGTTATACTGTTTTTGTTTGTAGA
TTTTTAAATTTTTGTTGCTGTTTTATAAATGTTGTGTGTTTTACATAGATTTTTATTTTTTTTTTTT
TTTTTTTTTTGATTAATTTTTTAATTAATTTATGATTTTTACTTTAGTAATTTTTATTATTTTGAGG
GGTGTTTGTTTTTGAATTTATGTTTTTGAACTGGTTTTGTTGCTAAATGTTTATTAAAAACTTAGGT
TTTTATGTAAAATTGTCTTCTGCTCTATGAGTTTTTAAATGGCAGCCTTAGCGTGATGGCGTAAAAG
TAGCGTAAGTGATTTGTTTTTTTAATGGTTTCAAGTATGAATGAAGTTTTTAGCAGTTTTTTTATTT
ACTTTTTATTTGAATTATTTTTTTGATTAAAAATTATTAGTTAAGGTATTACAAAGATAAGTCTTCG
GAAATTTTGTTTTGAATTTTGAAATTTTTGTTTTTAATTTTTTCTTGGGGATGGATTTTAAGAAAGT
TTTATACTATTGTTATTATTAAAAATTACTCCGGAGTTAACAGGGTTGTAGACATATAAATAGATTT
TTATATTAGTGTGCTGCGCTACATCGATGTTGTATATTTTTTTGATAATGGAGAGGTTTTTTTAT
TTTGAGACTGTTCTTCTTGTATAAAAAATTGACTTGATATTAGTTTAGTTCGTCGTGAGACAGAGCG
GTTTATCTTGTGTATTTTTGGTTTTTGGCGGTGTTAGTACGAAAGGAATGCAATGTGGGTTTATATT
TATGACTTTTTTATTTTGATGGGTTTTT

>O.ochengiF7
ATTTTTTGTGGAATGACTTTTGGTAATGGTATGAAGCAGAGTATTATTAATACTGTGAATCATAAGA
CTATTGGTACTTATTATATTGTTTTAGGTTATTGGGCTGGTTTAGGTGGTTCTGTTTTATCTATGTT
GATTCGTTTTGAATTGTCTAGTCCTGGTGGTTATTTGTTTTTTGGAAGTGGTCAGGTTTATAATTCT
GTTCTTACTATGCATGGTGTTTTGATGATTTTTTTTTTTGGTTATGCCTATTTTGATTGGTGGCTTTG
GTAATTGGATGTTGCCTTTAATATTAGGGGCTCCTGAGATGGCGTTTCCTCGGGTAAATGCTTTATC
TTTTTGATTTACTTTTGTGGCTTTGTTGATAGTTTATCAGTCTTTTTTTATTGGGGGTGGCCCTGGT
AGAAGTTGGACTTTTTATCCTCCTCTTAGGGTTGAAGGCCAACCAGAATTGTCTTTAGATACTATGA
TTTTAGGTTTACATACTGTAGGAATTGGTTCTTTGTTGGGTGCTATTAATTTTATGGTAACTACTCA
GAATATACGGTCTACTGCTGTGACTTTGGATCAAATTAGTATGTTTGTTTGGACTTCTTATTTGACT
TCTTTTTTGTTAGTTTTGTCTGTGCCTGTTTTGGCTGGTTCTTTATTGTTTTTGTTGTTGGATCGTA
ATTTTAATACTTCTTTTTATGATACTAAGAAGGGGGGTAATCCTTTGTTGTATCAGCATTTNTTTTG
ATTNTTTGGTCATCCTGAGGTGTATGTTATTATTTTACCTGTTTTTGGTATTATTAGGGAAGCGGTT
TTATTTTTGACTGATAAGGATCGTTTGTTTGGTCAGACTAGGATAACTTTTGCTTCTATTTGGATTG

CTGTTTTAGGTACTTCTGTGTGAGGTCATCATATGTATACGGCTGGTTTGGATATTGATACTCGTAC
TTATTTTAGTGCTGCTACTATGATTATCGCTATTCCTAGAGCTGTTAAGATTTTTAATTGGTTAGGT
ACTTTTTTTGGTTCTAGTCAAAAGGTGCAGCCGTTATGATGTTGAACTTATAGTTTTATTTTTCTTT
TTACTGTGGGTGGATTAAGTGGAATTATTCTGAGGGCTGCTAGTTTGGATATTATTTTGCACGATAC
TTATTATGTTGTGGCTCATTTTCATTATACTTTGAGTTTGGGTGCTATTTATGGTATTTTTTGTGGT
TTTTGTTTGTGACTTCCTTATATGTATGGTATTTCTTTTGATAGGGTTATGATAATAGCTGTTTTTG
TTTGTTTTTTTGTTGGTACTAATATGACATTTTTTCCTATGCATTTTGCTGGTTTGCAGGGTATGCC
TCGTAAGATTTTGGATTATCCTGATTGTTATTCTACTTTTCAGATTATTTCTTCTTTAGGTTCTGTT
ATTACTTTTGTTGGTTTTGTTTTGTTTAATTATTTGTTGGTTGATTCTATTTTTTTTTCTCGTTTTT
TGGGGGTTTCTTTTTATAATTATCATAGTCCGGCTTATGCTTTAAATGTTCCTCCTTTGCCGGATTC
TTTTACTGAAGAGGCTTTTATTATAGGTCTTCATTGGAAGATTATTAGTAAGGATACTCCTTCTTAT
AGGTATCGTCGGGTTGGTTATGGTTATCATAGTAAGTAAATTTTTTTTTATGTTAGGGTATTTTTTG
CTTTTGTTTTTTTTTGTTTGAGTTTTTTGGATTGGGATCTGTTGAAGAGTTGTGTTATGATGTGTTT
GGGTATTATGTCTATAAGTTGTTATGTTTCTTTGGGTATTCATGTATGATATTCTTATTTTGTTGTT
TTAATTTTTTTTAGTGGTATTTTTTCTTTGTTGACTTATTTTTGTAGTATGAGTAATTTTGTTTTTT
ATTATAATTATTTTTTTTTTTTTTCTTTGTTTTTGGTTAGTTTTTTTTTTGTATTTGTTGTAGATTT
TGATTTTTTTTTGTTTTTTTGTGATTTTAATTTTCTTTATGTTTGTTATGATTTTAGTTATTATTAT
GTTTTTTGAGTAGTTTTTGTTTTGTTTTTGTTTTTGGTTTTGGTTAGATTTAGGTTTAATGGTTTTG
GTTATATGCGTAGCTTGTAGATTGTTATTTTTAATTCTTTAGTTTTTTTGCCTGCTAGTTTTACTTT
GAGTTATATGTGAAATTTTGGTAGTATGTTGGGTATTATGTTGATGTCTCAGATTTTAACTGGTTTT
TTTTTGACTTTTTACTATACGGCTGGGGAAGCTTTTAGGTCTGTTCAGTATATTATGTTTGAGGTTA
ATTTGGGTTGGTTGTTGCGTATTATGCATTCTAATGGGGCTTCTATGTTTTTTTTGTTTATTTATTT
ACATATTTTTAAGGGTCTGATTTATGGTAGATATCGTCTTATTGGTGTATGGTTGAGTGGTATTTTT
ATTTATTTTTTATTGATAGGTATTGCTTTTACTGGTTATGTTTTGATTTGAGGTCAAATAAGTTATT
GGGCGGCAGTAGTTATTACTAGTTTAATAACTTCTGTTCCTTATTTAGGTAAGTATTTAGTTTGATG
GATTTGGGGGAGTTTTAGTGTTTGTGAGAACACTTTAAAGTTTTTTTATTCTGTTCATTTTATTTTA
CCTTGGTCTTTGATAGTTTTAGTTGTTTTTCATTTGTTTTTTTTGCATTTTACTGGTTCTAGTTCTA
GTTTGTATTGTCACGGGGATTATGATAAGATTCATTTTTTTCCTAGTTTTTGATTGAAGGATGGTTT
TGATATTTTTTTTTATTTTTTTTTGATTTTGTTTAGACTTTATTTTTCTTTTGATTTAAGTGATCCT
ATGATTTTTGTGGAGTCTGATTCTATGGCTAGTCCTGCGCATGTTGTACCTGAGTGATATTTTTTAT
TTGCTTTTACTATCTTACGTTCTGTTCCTAGTAAGTTATTAGGGGTTATTTTAATATTTAGTTCTGT
TTTTGTATTGACTATTCTTGTTTGACCTGATAGTTATCAGTCTATTTTGGATAATTTTTTATATTTT
TTTGTTATGTGTTTTGTTTGGATTTTTTTTTGGTTAACTTGAGCTGGTCATTATCCTACTGATTATC
CTTTTAACTATTTTAATTTGTTTTGTACTTTTTTTTATTTTTGTTGTATTTTTTTTTGTTTGTTTAAT
TAATTTTTTTAGTGATAAGTTGTTTAGTTAAATTTTGTTGAAGTTTCGTAAGTACCATAAAATGGAG
TATAGTTATTATCCTTTGATAGTTGGGGCGGGTATTTTAGGTTTTGATGTTAGTTTGGTTTTATTTA
TAAGTATAGGTATGTTTTATTCTATTTTTATTTGTTTTTTGTATTTGGTTTATGTTTTTTTTTTTGTG
AATCAAGGATGTTATTTTAGAGGACATTAGTGGTCAATATTCTTTTTATGACTATCGTATGTTTAAT
CAAGGTTTTCGTTTGTTTCTTTTTAGTGAGTTAACTTTGTTTGTTTCTATTTTTTGAACTTTTTTGG
ATACAGCTTTGTGTCCTTTAACTTGGTTAGGTGGGGTTTGGTCTCCATTTGGGATTTTATCTCCTGA
TTATTTGGGTTTGAATGGTATGGCTAGTTTGTTTTTAATGATAAATAGGCAAGTTTTGAAGTATTCT
CGTCGTTATTTGTGTTTGAGTAGTTCTAAGTGTGAAGAGTTTTTGTTAGTTTGTATTTTTGTTGGAG
TTGGTTTTTTATGTTTTCAGTTTTATGAATATAATAACAATTCGTTTGTTATGAGTGATAGTGTTTA
TGGTAGTATTTTTTATATGGGTACTGGTTTACATGGTTTGCATGTTTTTATTGGTGTTTGTTTTCTT
ATTGTTAATTTTTTTCGTGTTAAGTTGTTCAATTTTAATTGATATCATGTTCAGGCTTATGATATAT
CTATTGATTATTGGCGTTTTTTAGAATGAATGTGAGGTGTTATGTTTTGTTTATTATATGTTTGAGG
TTCTTAATTGGTTATTTTTTATTATTTGGGGTTGTTGGTTATGATTGTTTTTATTTTGCAGGCTATT
GCTTTTTTAACTTTGTTGGAGCGCCATTTTTTGGGTGGTTCTCAGTGTCGTGTTGGTCCTAATAAGG
TGGGTTATTCTGGTGTTTTGCAGGCTTTATTTGATGGTTTAAAGTTGTTAAAGAAGGAGCAGTTGTT
GTTGTGTTTTTCTTCTTGATTATCTTTTTTGTTTATGCCTGTTTGTGGTTTTGTTTTGATGGTTTTT
TTTTGATTTACTTTGCCTTATTTTTTTTTCTTTTTTGTCTTTTGAGTACTCTGGTGTTTTTTTGTTTT
GTCTTATAGGGGTTTCTGTTTATTTTATTATGCTTTCTGGTGTTTTTAGCGGTAGTAAATATTCTTT

TGTTGGTGGATTGCGTGCTTGCGTTCAGAGTTATTCTTATGAGATTGCTTTTTCTATTTATTTGTTG
GTTTTTTTGTTGTTTAATAAGGGTCTATGTTTGTCTTTTAGTTTTTGTTTATTTTTTTTTTTTGTTTT
TTTTTCCTTTTTTTTGTTTAGTTCTTGTTGATTTGCATCGGGCTCCTTTTGATTTTTCTGAGTGTGA
AAGTGAGTTGGTAAGGGGGTTTAATGTTGAGTATTCGGGAGTTGGTTTTGCTGCTTTGTTTTTAGGG
GAGTATGGTAATTTACTTTATTTTGGTTGTTTGACTTCTAGTTTGTTTTTTGGTATAAGTTTTTTTT
TTTTTTATTTTATTGTATGTATGATTGTTTTTTCTCGTAGAGCTTATCCTCGTTTTCGTTTTGATAA
GTTGATGGGTGTTTGTTGGTTTTTGTTTTTGCCTGTCGGTTTTTATTTTTTTGGTTTGTCTTTTGTT
GTTTTTATGTTGTGCTTATTTAGTTTAATTTTTTAGATTTTGTTATTTTTTTTTTATTTGGTTTTGAT
TAGTTTTTTATTTGTTTTATATGGAGTTGAGTAAGTTTAGTAGTTTGGGAGTTTTAGGTGTTTTTGT
TAATGTTTTAGTTTCTGGATTTTCTCATCAGGGTTTTCAGTCTAGTGTTTTTTTTAAGTTTGTTGTT
TTTTTTCTTTTGGTTTTTTGAATGAGTGGGTTATTGTTTCCTTTGTTTAGTCCTTGGGCTTGTGTGG
GTTTTTTGTTTTTTGTTACTAATTTTTCTTGATTGGGTGTTCGTACTTTTATTTTAGCTGTAGATAG
TTTTTTGATTTTTTTTGAGGGGGATCACTCTTGGGAGTGGTTTTCTAGGTTAGTTATGTTTTTTTCT
CATTGGTTGAGATTTTTGATAAGGGGGGTGGCTTTAACTTTGCGTATTAGTATTATTTTTTTAATTG
GTCATTTTTTAATGTTTACTGTTTTGGATATGAGTGTATTTTATTCTTTGTTTTTTTTGTTGTTTGT
GGTTCCGGTGGAGTTGTTTTTTGCTTTTTTACAGAGTTATATTTTTTTGACTTTGGTTTGTATGTTT
TTACTTAATATGATTTAGATTTATTTGCAGAATTATGTGTTTCCTATTCCTGGAAATTCTTATGTGT
TTTGTTGTTATTATATCCATAATTATTATTCTCATATTATTTTTTTTGGTTTTTTTGTGATGTTTTT
GGTTAGTGGTGGGGTTTATTTTTTTTGGCAATTCTTTTAAGTTTAACTTGAAGCGGAGAGATAGTCGT
ATAATTGAATTAGTTTTACAGGTGTTGATTGTTAATTTTTTGATTATGATGGCAGGTCCTGGTTTTT
GGTTGATTCAGTATCAGGGACGTATGTTTCGTCAATCTGAGTTGGCTTTGAAGGTTATTGGTCATCA
ATGGTATTGGAGTTATGAGTATGGTGATAGTGGAAAATTATGTTTTGATTCATTTATGAAGTCTTTA
GATGATTTGTCTTTAGGGGATTTTCGGTTATTTGATGTTGATAATCGGTGTGTTTTGCCTGTAGGTG
TGAATGTTGGAGTGTATTGTACTTCTAGTGATGTTATTCATTCTTTTGCTATTCCTAAGTGTTTTAT
TAAGATGGATGCTTTGAATGGTTTGTTAACTAAGGTTACTTGTAATTTTTCTTGTTCTGGTTTGTTT
TTTGGGCAGTGTTCTGAAATTTGTGGTGCTAATCATAGGTTTATGCCTATTGTGTTGGAGTTGACTT
CTTTGGAGTGTTGGAAGGGTTGATCAGTTAATTATTTGCTGGGTTAACTTTTTTTAAATTTTATTGT
TATTTTTTTTTTTTCTTTTTTGGTTCCTTTTGGTATGTATTTGTTGTCTTTTTTTGTGTCTTTTAAG
GATTTTTATGGTGCTAAATTAAGTTCTTATGAATGTGGTTTTGATGTTGTGAAGAAGGTTCATGTTG
GTTTTAATTTGGTTTTTTTTTCTATTGTTTTGTTGTTTGTTGTTTTTGAGTTGGAAGTTTTAATTTT
TATTATTTTGATTCAGGGTGATTTTTATAGTTTATTGTCTTTTTTTTGTTTTTTTTTTTATGTTGTT
TTTAGTTTTTATATGGAGTGGTATTTTGGTAAGTTGATTTGGTTTTGTTAGTTGTTATTGTTTGTTT
GATATGTTCTTATTTTTTATTTTTTTTTGGTTGTTGTTTTTTTGTTTGTTCCTTATGGTAAATGGAG
TTATAGTTTTGGTTTTAGTGATTATTTTAATTTTACTTTTGTTTATAATTTTGAAGTTTGTTTGTTT
TTTTTAGTTTTGTTGTTGGTTTCTTTTATGGTTTTTGTTTATGGTTCTTTTTATATGGTTGGGGTTT
CTCGTTTGTTTTATTTTTTTTTTTTTTTTTATTTTTGTTTGTGTTGAGGATGGGTGGCTTGATTGTTTT
TAGAGGTAGTATTGTTTTAACTTTGGTTTTTTGGGATTTTTTGGGGGTTAGTAGTTTTTTTTTGGTT
TTGTTTTATGGTAATGTTAGTGCTCGAAGGGGTGCTATGAGTACTGTGTTTACTAATCGTATTGGTG
ATTTTTGTATTTTTTTGTTTTTTAATGGTTTTGTTTTGTTTTCTATGAGTTTTTTGTCTTATCAGTT
TTTTGGTTCTTTGTTAGTTTTTATGTTGTTTGTTTCTTCTGTTATTAAGGGTGGTCAGTATCCTTTT
GGTAGCTGGTTGCCTAAGGCTATGGCTGCTCCTACTCCTGTTAGCTGTTTGGTTCATAGTAGTACTT
TAGTTACTGCTGGTGTTATGTTGATGGATTGTTATGTTTATATTTCTTTGAATTCTGATGTTTTGTC
TTTTGTTTTTTATGTTGGTTTTTTTACTATAGTTTTTTCTGGTTTTTGTGCTTTGGTGGAGGAGGAT
GCTAAGAAGATTGTTGCTTTGAGTACTATGTCTCAGATTGGTTTTTGTTTTTTGGCTATTGGTAGGG
GTTTGCATTATTTGTCTTATGTTCATATGATTAGGCATTCTTTTTTTAAGAGATTGTTGTTTATGCA
GATAGGTTATTTGATTTTTATTAATTTTGGTCAACAGGATTATCGTGGTTATTCTTTTTTTGGTTTT
TGTGCTCCGGTTTTAGTTCAGTTGCAGATTTTTTTATCTGTGTTTTGTTTGTGTGGTTTGTTGTTTA
CTAGAGGTAGTTGTAGTAAGGAATATTTTATATCTCGTTTTTATTATGATTCTTATGGTTTTTTTTT
AGTTTTTTTTTATTTTTTTGGTGTGTTTTTGACTTTTTGTTATTGTTATCGGATGTTTTTTTTTGTTT
CGTGTGGGGGCTTTTGGTTTTGATTATGTGGGTTTTTCTAGTAAGTTANTTTTATTTTTCTTGTTTT
TTTTTGGTTTTTTTTTCTGTTGTTTTTACTTTTTGGTGGGTTTTTGGTTTGTTATCTTTTTCTGCGG
CTTTTAATCGTTTTGAGTTTTTGGTTGTTTATTTTTATTTGTTTTTTGTTTATTGTTTTTGTGGTTA

TTTTTTTCGTTATTTTTTTTTTCGTTATTTTGTGAAATTTTTTATGGATCATTATGCTTGTTTTATTT
ATAAAATTTTTCCTAGTTTTTTTTATTTTGATGTTTTTATTATGGGTTTTAATTATTTTTTTTTTGG
GTTATTTCGGTTATTTTCTTTTTTTTTTTTTTTCTTGGTTTCGGGGGTTTTATCATGTTGGTGTTTTA
ATTGTTTTTTTTTTTTATGTTGTTTTTTTTGTTTTTTTAGATTTTGTTATTTTTTGTTTTTTTTTTTAT
TATTGTTTTTGAGTTTTATTAATTTTTGTGTTGTTGATTATATTGTTTGGTGGAGGATTTTTGTTAT
TTGTACTTTTGTTTTTGTTTTTTTTGTTGGTGGTGAGTTAGGTTTTGGGGGTTATTTGGTTAATTAT
TATGTTATTCAGGAAGTTTGTGGTTATTATTTTTTGGTTTTTGATGGTTGGAAGTTGCAATTTTTAT
TGCTTATGTTGAAGTCTGGTTCTTCTCCTTTTCATTTTTGACTTTTTAGTGTTTTGGGTGGTTTGGA
TAAGTGGTTTATTTTGTGGTTTTTAACTTTGCAAAAATTGCCTTATTTTGTTGTTTTGGTTAATTTT
TGTGGTGATTTTTTTTTTTTTGTTTTTGTTTTTTGGTATAATTTTTTGTTATTTTCAATTTTTTTTGT
TGCGTAGTTATCGTGATTTGTTAGTTGTGGGTTCTGCTGAATCTTTTAATTGGTTATTGTTATTGGG
TATTTTTTCTTTTAATGAAGTATTTGTTTTGTTTTTTTTTTATTATTTTGTTATGTTTTTTGTTGTC
TCTTATGTGTATGGGGGATTTTTAAATTTTTTGAGTTTAGAGATATTGATGTTTTTTTTTAATGTTC
CTTTGAGAATTACTTTTTTTTTAAAGGTGATTGTGTTGTTTGGTTCTTCTTTTTTTGTTGGTTTTTA
TTATTTATTTTTGTTGTTGTTTATGCCTTTGATGTCTTTGGGTATGGGTTATTTGTTTTTTTTTGGTT
TCGATGATGAGTTTTAATTATGGTTTTAAGTATTATGATTATTTTGTTTATGTTTTGTTTTGTGTTG
GGTTGTTGTCTTGTTTTTAGTTGTTGTATTTATTTTTTTTTTGTTGTTTTGTTGTTTTTTTTTAGTCC
TTTTTTATTTTTTGTTTTTTTTATGTTTTTTGTTTTATATGGTTTTTTTGATTGTTCATGGTTTGGT
TGTTTGTTTTTTTTTGATTCTTTTAATTTTGTTTTTTTGTCTTTTATGAGTGTTTTTGTTATGGGGT
TTATTTGTGTGTCTGAATTGTTGAGTGGCTTAGTTTTTTATAGTTGTCTTGTAGTGTTTTTTAGTGT
TTGTTTTTTTTTATTCTGGTAGTTTTTTGATATTGTATGTTTTTTTATGAGTTGACTATAGTACCTATT
TTGTTTTGTTTGTTAGGGTATGGTCGTCAGGTGGAGAAGGTTAGGGCTTGTTATTATTTAATTTTTT
ATACTTTGTTTTTTGGGATGCCTTACTTGTTTTTGTATAGTCATGTTTTTTTTTTTTTGAATTTTGT
TTATTATGATTTTTTTTGTTTCTTATGAATTTATTTTTTTGTTGAGTTTGTGTTTTTTAGTTAAGTTT
CCTGTTTATTTTTTTCATGTTTGATTACCTAAGGTTCATGTTGAGGCTCCTACTAGTGCTAGAATGA
TTTTGGCTGGTGTTATGTTGAAGTTGGGAGGAGCAGGTGTTTATCGTATTAGTAAGTCTTTGAATTT
TTTTGGTTTTGAAATGTTGATTTTTTTTTTCTTTGATTAGGATGGTTTTTTGTTCTTTTATTTGTGTT
GTTCAGAGTGATTGTAAGTCTTTGGCGGCTTATTCTTCTGTTTGTCATATGGGTTTTGTATTGCTCT
CTGAGATTAGTATGGTTTATTATGGTAAGTCTATGGCTTTGGTGATGATGTTGGCTCATGGTTATAC
TTCTGTTTTAATGTTTTATTTTATTGGTGAGTTTTATCATATTGCTAATAGGCGTTTAATTTATTAT
TTGCGTGGATATTTTAATGTTAGTATGTTGTTTTGTTTGATGTTTTGTTTGACTATGGTTTCTAATT
TTAGTTTTCCTGTATCTATTTCTTTTTTTTCTGAGTATTTGATGTTGAATTTTTTTAGGTCTGTTTT
TTATGTTGGTTTTTTGTTTTTGTTTTTTTATTATTTGGTTTCTTTTTATTATTCTGTTTATATTTTG
GTTTGTTTTTTAGTTGGGGATAAGGTGAGTTATGTTTGTGATGGTCGTAGTGTTGTTTGTTTACCTT
TGGTTTTTATGATATATAATTTTTTTTGGTTTATTTTTGTTATTTAATTTTAAGTTTGATTTTGGTT
TAGGTTGTATTAAGATAGTATTACTTATTTTTAGTTTATTTAATGTGTTATTTTTTGTACACTGGTA
GTTTTTTGATTGTTTTATTAACGTTCCAGAATAATCGGCTATGCGTTTTAATTTTTGACTCTATTTG
TTGTGGTGCTATGAGTTTTTAGTTTGTTTTTATGTTGTTTTTTGTAAAATATTTTGATTTTTTTTAG
TTTCTTGTGGTATCAAAAATTTGTTTTTTGAACTGGATTAGTACCCAGGTAATCAAAATTTAATAAT
TCGGGAGTAAAGTTTTGTTTAAACCGAAAAAATATTGACTGACTTTAGATTTTTCTTTGGAACATGT
GATTTGCTGGAGAGCCCTCTTTTTTGGTGAATTTTGTTGGCACATGTATGATTGTTTAGTTTTTATT
TTATTTTGTAATGCTTTGTTGTTTTGGCATTAAAAACAGATATATATTTGGCTTATGAATTTATGTT
TCATGTGTTACTATTATGAATTTTTTTTGGATTAGTTTTTTATTTTTTTTTGAAATTGGAAAAGAAA
GTAATTTTTTTTTAATGTTTTAATGAATTTAATAAATAAGGTGGTACAAACCATCCGTCAATGGCCT
AAAGGGGCGTAAGTTGTAGTATGGTAGAAGTAAGGAAACTTGTTTCTATTTTTTGAAGTTTTTTTGT
TTTTTTAAGTTTTATTATTTGGTATTGCATATCAGTAGAAGTTTTTATCATAGTTATGAGTAATAGA
ATTAAAATGGTTAAATTGAATTTGTTTTTTTTTTACGAAATTAATAATATTTTTATTTTAGTATTGA
TATAACGTATTTTTATTTCTGTTTATTTGTTATTTATAGGTTAAGTTATACTGTTTTTGTTTGTAGA
TTTTTAAATTTTTGTTGCTGTTTTATAAATGTTGTGTGTTTTACATAGATTTTTATTTTTTTTTTTT
TTTTTTTTTTGATTAATTTTTTAATTAATTTATGATTTTTACTTTAGTAATTTTTATTATTTTGAGG
GGTGTTTGTTTTTGAATTTATGTTTTTGAACTGGTTTTGTTGCTAAATGTTTATTAAAAACTTAGGT
TTTTATGTAAAATTGTCTTCTGCTCTATGAGTTTTTAAATGGCAGCCTTAGCGTGATGGCGTAAAAG

TAGCGTAAGTGATTTGTTTTTTTAATGGTTTCAAGTATGAATGAAGTTTTTAGCAGTTTTTTTATTT
ACTTTTTATTTGAATTATTTTTTTGATTAAAAATTATTAGTTAAGGTATTACAAAGATAAGTCTTCG
GAAATTTTGTTTTGAATTTTGAAATTTTTGTTTTTAATTTTTTCTTGGGGATGGATTTTAAGAAAGT
TTTATACTATTGTTATTATTAAAAATTACTCCGGAGTTAACAGGGTTGTAGACATATAAATAGATTT
TTATATTAGTGTGCTGCGCTACATCGATGTTGTATATTTTTTTTGATAATGGAGAGGTTTTTTTTAT
TTTGAGACTGTTCTTCTTGTATAAAAAATTGACTTGATATTAGTTTAGTTCGTCGTGAGACAGAGCG
GTTTATCTTGTGTATTTTTGGTTTTTGGCGGTGTTAGTACGAAAGGAATGCAATGTGGGTTTATATT
TATGACTTTTTTATTTTGATGGGTTTTT

>O.ochengiF8
ATTTTTTGTGGAATGACTTTTGGTAATGGTATGAAGCAGAGTATTATTAATACTGTGAATCATAAGA
CTATTGGTACTTATTATATTGTTTTAGGTTATTGGGCTGGTTTAGGTGGTTCTGTTTTATCTATGTT
GATTCGTTTTGAATTGTCTAGTCCTGGTGGTTATTTGTTTTTTGGAAGTGGTCAGGTTTATAATTCT
GTTCTTACTATGCATGGTGTTTTGATGATTTTTTTTTTGGTTATGCCTATTTTGATTGGTGGCTTTG
GTAATTGGATGTTGCCTTTAATATTAGGGGCTCCTGAGATGGCGTTTCCTCGGGTAAATGCTTTATC
TTTTTGATTTACTTTTGTGGCTTTGTTGATAGTTTATCAGTCTTTTTTTATTGGGGGTGGCCCTGGT
AGAAGTTGGACTTTTTATCCTCCTCTTAGGGTTGAAGGTCAACCAGAATTGTCTTTAGATACTATGA
TTTTAGGTTTACATACTGTAGGAATTGGTTCTTTGTTGGGTGCTATTAATTTTATGGTAACTACTCA
GAATATACGGTCTACTGCTGTGACTTTGGATCAAATTAGTATGTTTGTTTGGACTTCTTATTTGACT
TCTTTTTTGTTAGTTTTGTCTGTGCCTGTTTTGGCTGGTTCTTTATTGTTTTTGTTGTTGGATCGTA
ATTTTAATACTTCTTTTTATGATACTAAGAAGGGGGGTAATCCTTTGTTGTATCAGCATTTGTTTTG
ATTTTTTGGTCATCCTGAGGTGTATGTTATTATTTTACCTGTTTTTGGTATTATTAGGGAAGCGGTT
TTATTTTTGACTGATAAGGATCGTTTGTTTGGTCAGACTAGGATAACTTTTGCTTCTATTTGGATTG
CTGTTTTAGGTACTTCTGTGTGAGGTCATCATATGTATACGGCTGGTTTGGATATTGATACTCGTAC
TTATTTTAGTGCTGCTACTATGATTATCGCTATTCCTAGAGCTGTTAAGATTTTTAATTGGTTAGGT
ACTTTTTTTGGTTCTAGTCAAAAGGTGCAGCCGTTATGATGTTGAACTTATAGTTTTATTTTTCTTT
TTACTGTGGGTGGATTAAGTGGAATTATTCTGAGGGCTGCTAGTTTGGATATTATTTTGCACGATAC
TTATTATGTTGTGGCTCATTTTCATTATACTTTGAGTTTGGGTGCTATTTATGGTATTTTTTGTGGT
TTTTGTTTGTGACTTCCTTATATGTATGGTATTTCTTTTGATAGGGTTATGATAATAGCTGTTTTTG
TTTGTTTTTTTGTTGGTACTAATATGACATTTTTTCCTATGCATTTTGCTGGTTTGCAGGGTATGCC
TCGTAAGATTTTGGATTATCCTGATTGTTATTCTACTTTTCAGATTATTTCTTCTTTAGGTTCTGTT
ATTACTTTTGTTGGTTTTGTTTTGTTTAATTATTTGTTGGTTGATTCTATTTTTTTTTTCTCGTTTTT
TGGGGGTTTCTTTTTATAATTATCATAGTCCGGCTTATGCTTTAAATGTTCCTCCTTTGCCGGATTC
TTTTACTGAAGAGGCTTTTATTATAGGTCTTCATTGGAAGATTATTAGTAAGGATACTCCTTCTTAT
AGGTATCGTCGGGTTGGTTATGGTTATCATAGTAAGTAAATTTTTTTTTATGTTAGGGTATTTTTTG
CTTTTGTTTTTTTTTGTTTGAGTTTTTTGGATTGGGATCCGTTGAAGAGTTGTGTTATGATGTGTTT
GGGTATTATGTCTATAAGTTGTTATGTTTCTTTGGGTATTCATGTATGATATTCTTATTTTGTTGTT
TTAATTTTTTTTAGTGGTATTTTTTCTTTGTTGACTTATTTTTGTAGTATGAGTAATTTTGTTTTTT
ATTATAATTATTTTTTTTTTTTTTNTTTGTTTTTGGTTAGTTTTTTTTTTGTATTTGTTGTAGATTT
TGATTTTTTTTTGTTTTTTTGTGATTTTAATTTTCTTTATGTTTGTTATGATTTTAGTTATTATTAT
GTTTTTTGAGTAGTTTTTGTTTTGTTTTTGTTTTTGGTTTTGGTTAGATTTAGGTTTAATGGTTTTG
GTTATATGCGTAGCTTGTAGATTGTTATTTTTAATTCTTTAGTTTTTTTGCCTGCTAGTTTTACTTT
GAGTTATATGTGAAATTTTGGTAGTATGTTGGGTATTATGTTGATGTCTCAGATTTTAACTGGTTTT
TTTTTGACTTTTTACTATACGGCTGGGGAAGCTTTTAGGTCTGTTCAGTATATTATGTTTGAGGTTA
ATTTGGGTTGGTTGTTGCGTATTATGCATTCTAATGGGGCTTCTATGTTTTTTTTTGTTTATTTATTT
ACATATTTTTAAGGGTCTGATTTATGGTAGATATCGTCTTATTGGTGTATGGTTGAGTGGTATTTTT
ATTTATTTTTTATTGATAGGTATTGCTTTTACTGGTTATGTTTTGATTTGAGGTCAAATAAGTTATT
GGGCGGCAGTAGTTATTACTAGTTTAATAACTTCTGTTCCTTATTTAGGTAAGTATTTAGTTTGATG
GATTTGGGGGAGTTTTAGTGTTTGTGAGAACACTTTAAAGTTTTTTTATTCTGTTCATTTTATTTTA
CCTTGGTCTTTGATAGTTTTAGTTGTTTTTCATTTGTTTTTTTTGCATTTTACTGGTTCTAGTTCTA
GTTTGTATTGTCACGGGGATTATGATAAGATTCATTTTTTTCCTAGTTTTTGATTGAAGGATGGTTT
TGATATTTTTTTTTATTTTTTTTTGATTTGTTTAGACTTTATTTTTCTTTTGATTTAAGTGATCCT

ATGATTTTTGTGGAGTCTGATTCTATGGCTAGTCCTGCGCATGTTGTACCTGAGTGATATTTTTTAT
TTGCTTTTACTATCTTACGTTCTGTTCCTAGTAAGTTATTAGGGGTTATTTTAATATTTAGTTCTGT
TTTTGTATTGACTATTCTTGTTTGACCTGATAGTTATCAGTCTATTTTGGATAATTTTTTATATTTT
TTTGTTATGTGTTTTGTTTGGATTTTTTTTTGGTTAACTTGAGCTGGTCATTATCCTACTGATTATC
CTTTTAACTATTTTAATTTGTTTTGTACTTTTTTTTATTTTTGTTGTATTTTTTTTGTTTGTTTAAT
TAATTTTTTTAGTGATAAGTTGTTTAGTTAAATTTTGTTGAAGTTTCGTAAGTACCATAAAATGGAG
TATAGTTATTATCCTTTGATAGTTGGGGCGGGTATTTTAGGTTTTGATGTTAGTTTGGTTTTATTTA
TAAGTATAGGTATGTTTTATTCTATTTTTATTTGTTTTTTGTATTTGGTTTATGTTTTTTTTTTGTG
AATCAAGGATGTTATTTTAGAGGACATTAGTGGTCAATATTCTTTTTATGACTATCGTATGTTTAAT
CAAGGTTTTCGTTTGTTTCTTTTTAGTGAGTTAACTTTGTTTGTTTCTATTTTTTGAACTTTTTTGG
ATACAGCTTTGTGTCCTTTAACTTGGTTAGGTGGGGTTTGGTCTCCATTTGGGATTTTATCTCCTGA
TTATTTGGGTTTGAATGGTATGGCTAGTTTGTTTTTAATGATAAATAGGCAAGTTTTGAAGTATTCT
CGTCGTTATTTGTGTTTGAGTAGTTCTAAGTGTGAAGAGTTTTTGTTAGTTTGTATTTTTGTTGGAG
TTGGTTTTTTATGTTTTCAGTTTTATGAATATAATAACAATTCGTTTGTTATGAGTGATAGTGTTTA
TGGTAGTATTTTTTATATGGGTACTGGTTTACATGGTTTGCATGTTTTTATTGGTGTTTGTTTTCTT
ATTGTTAATTTTTTTCGTGTTAAGTTGTTCAATTTTAATTGATATCATGTTCAGGCTTATGATATAT
CTATTGATTATTGGCGTTTTTTAGAATGAATGTGAGGTGTTATGTTTTGTTTATTATATGTTTGAGG
TTCTTAATTGGTTATTTTTTATTATTTGGGGTTGTTGGTTATGATTGTTTTTATTTTGCAGGCCATT
GCTTTTTTAACTTTGTTGGAGCGCCATTTTTTGGGTGGTTCTCAGTGTCGTGTTGGTCCTAATAAGG
TGGGTTATTCTGGTGTTTTGCAGGCTTTATTTGATGGTTTAAAGTTGTTAAAGAAGGAGCAGTTGTT
GTTGTGTTTTTCTTCTTGATTATCTTTTTTGTTTATGCCTGTTTGTGGTTTTGTTTTGATGGTTTTT
TTTTGATTTACTTTGCCTTATTTTTTTTTCTTTTTTGTCTTTTGAGTACTCTGGTGTTTTTTTGTTTT
GTCTTATAGGGGTTTCTGTTTATTTTATTATGCTTTCTGGTGTTTTTAGCGGTAGTAAATATTCTTT
TGTTGGTGGATTGCGTGCTTGCGTTCAGAGTTATTCTTATGAGATTGCTTTTTCTATTTATTTGTTG
GTTTTTTTGTTGTTTAATAAGGGTCTATGTTTGTCTTTTAGTTTTTGTTTATTTTTTTTTTTTGTTTT
TTTTTCCTTTTTTTTGTTTAGTTCTTGTTGATTTGCATCGGGCTCCTTTTGATTTTTCTGAGTGTGA
AAGTGAGTTGGTAAGGGGGTTTAATGTTGAGTATTCGGGAGTTGGTTTTGCTGCTTTGTTTTTAGGG
GAGTATGGTAATTTACTTTATTTTGGTTGTTTGACTTCTAGTTTGTTTTTTGGTATAAGTTTTTTTT
TTTTTTATTTTATTGTATGTATGATTGTTTTTTCTCGTAGAGCTTATCCTCGTTTTCGTTTTGATAA
GTTGATGGGTGTTTGTTGGTTTTTGTTTTTGCCTGTCGGTTTTTATTTTTTTGGTTTGTCTTTTGTT
GTTTTTATGTTGTGCTTATTTAGTTTAATTTTTTAGATTTTGTTATTTTTTTTTTATTTGGTTTTGAT
TAGTTTTTTATTTGTTTTATATGGAGTTGAGTAAGTTTAGTAGTTTGGGAGTTTTAGGTGTTTTTGT
TAATGTTTTAGTTTCTGGATTTTCTCATCAGGGTTTTCAGTCTAGTGTTTTTTTTAAGTTTGTTGTT
TTTTTTCTTTTGGTTTTTTGAATGAGTGGGTTATTGTTTCCTTTGTTTAGTCCTTGGGCTTGTGTGG
GTTTTTTGTTTTTTGTTACTAATTTTTCTTGATTGGGTGTTCGTACTTTTATTTTAGCTGTAGATAG
TTTTTTGATTTTTTTTGAGGGGGATCACTCTTGGGAGTGGTTTTCTAGGTTAGTTATGTTTTTTTCT
CATTGGTTGAGATTTTTGATAAGGGGGGTGGCTTTAACTTTGCGTATTAGTATTATTTTTTTAATTG
GTCATTTTTTAATGTTTACTGTTTTGGATATGAGTGTATTTTATTCTTTGTTTTTTTTGTTGTTTGT
GGTTCCGGTGGAGTTGTTTTTTGCTTTTTTACAGAGTTATATTTTTTTGACTTTGGTTTGTATGTTT
TTACTTAATATGATTTAGATTTATTTGCAGAATTATGTGTTTCCTATTCCTGGAAATTCTTATGTGT
TTTGTTGTTATTATATCCATAATTATTATTCTCATATTATTTTTTTTGGTTTTTTTGTGATGTTTTT
GGTTAGTGGTGGGGTTTATTTTTTTGGCAATTCTTTTAAGTTTAACTTGAAGCGGAGAGATAGTCGT
ATAATTGAATTAGTTTTACAGGTGTTGATTGTTAATTTTTTGATTATGATGGCAGGTCCTGGTTTTT
GGTTGATTCAGTATCAGGGACGTATGTTTCGTCAATCTGAGTTGGCTTTGAAGGTTATTGGTCATCA
ATGGTATTGGAGTTATGAGTATGGTGATAGTGGAAAATTATGTTTTGATTCATTTATGAAGTCTTTA
GATGATTTGTCTTTAGGGGATTTTCGGTTATTTGATGTTGATAATCGGTGTGTTTTGCCTGTAGGTG
TGAATGTTGGAGTGTATTGTACTTCTAGTGATGTTATTCATTCTTTTGCTATTCCTAAGTGTTTTAT
TAAGATGGATGCTTTGAATGGTTTGTTAACTAAGGTTACTTGTAATTTTTCTTGTTCTGGTTTGTTT
TTTGGGCAGTGTTCTGAAATTTGTGGTGCTAATCATAGGTTTATGCCTATTGTGTTGGAGTTGACTT
CTTTGGAGTGTTGGAAGGGTTGATCAGTTAATTATTTGCTGGGTTAACTTTTTTTAAATTTTATTGT
TATTTTTTTTTTTTTCTTTTTTGGTTCCTTTTGGTATGTATTTGTTGTCTTTTTTTTGTGTCTTTTAAG
GATTTTTATGGTGCTAAATTAAGTTCTTATGAATGTGGTTTTGATGTTGTGAAGAAGGTTCATGTTG

GTTTTAATTTGGTTTTTTTTTCTATTGTTTTGTTGTTTGTTGTTTTTGAGTTGGAAGTTTTAATTTT
TATTATTTTGATTCAGGGTGATTTTTATAGTTTATTGTCTTTTTTTTGTTTTTTTTTTTATGTTGTT
TTTAGTTTTTATATGGAGTGGTATTTTGGTAAGTTGATTTGGTTTTGTTAGTTGTTATTGTTTGTTT
GATATGTTCTTATTTTTTATTTTTTTTTGGTTGTTGTTTTTTTGTTTGTTCCTTATGGTAAATGGAG
TTATAGTTTTGGTTTTAGTGATTATTTTAATTTTACTTTTGTTTATAATTTTGAAGTTTGTTTGTTT
TTTTTAGTTTTGTTGTTGGTTTCTTTTATGGTTTTTGTTTATGGTTCTTTTTATATGGTTGGGGTTT
CTCGTTTGTTTTATTTTTTTTTTTTTTTATTTTTGTTTGTGTTGAGGATGGGTGGCTTGATTGTTTT
TAGAGGTAGTATTGTTTTAACTTTGGTTTTTTGGGATTTTTTGGGGGTTAGTAGTTTTTTTTTGGTT
TTGTTTTATGGTAATGTTAGTGCTCGAAGGGGTGCTATGAGTACTGTGTTTACTAATCGTATTGGTG
ATTTTTGTATTTTTTTGTTTTTTAATGGTTTTGTTTTGTTTTCTATGAGTTTTTTGTCTTATCAGTT
TTTTGGTTCTTTGTTAGTTTTTATGTTGTTTGTTTCTTCTGTTATTAAGGGTGGTCAGTATCCTTTT
GGTAGCTGGTTGCCTAAGGCTATGGCTGCTCCTACTCCTGTTAGCTGTTTGGTTCATAGTAGTACTT
TAGTTACTGCTGGTGTTATGTTGATGGATTGTTATGTTTATATTTCTTTGAATTCTGATGTTTTGTC
TTTTGTTTTTTATGTTGGTTTTTTTACTATAGTTTTTTCTGGTTTTTGTGCTTTGGTGGAGGAGGAT
GCTAAGAAGATTGTTGCTTTGAGTACTATGTCTCAGATTGGTTTTTGTTTTTTGGCTATTGGTAGGG
GTTTGCATTATTTGTCTTATGTTCATATGATTAGGCATTCTTTTTTTAAGAGATTGTTGTTTATGCA
GATAGGTTATTTGATTTTTATTAATTTTGGTCAACAGGATTATCGTGGTTATTCTTTTTTTGGTTTT
TGTGCTCCGGTTTTAGTTCAGTTGCAGATTTTTTTATCTGTGTTTTGTTTGTGTGGTTTGTTGTTTA
CTAGAGGTAGTTGTAGTAAGGAATATTTTATATCTCGTTTTTATTATGATTCTTATGGTTTTTTTTT
AGTTTTTTTTTATTTTTTTGGTGTGTTTTTGACTTTTTGTTATTGTTATCGGATGTTTTTTTTTGTTT
CGTGTGGGGGCTTTTGGTTTTGATTATGTGGGTTTTTCTAGTAAGTTANTTTTATTTTTCTTGTTTT
TTTTTGGTTTTTTTTTCTGTTGTTTTTACTTTTTGGTGGGTTTTTGGTTTGTTATCTTTTTCTGCGG
CTTTTAATCGTTTTGAGTTTTTGGTTGTTTATTTTTATTTGTTTTTTGTTTATTGTTTTTGTGGTTA
TTTTTTTCGTTATTTTTTTTTCGTTATTTTGTGAAATTTTTTATGGATCATTATGCTTGTTTTATTT
ATAAAATTTTTCCTAGTTTTTTTTATTTTGATGTTTTTATTATGGGTTTTAATTATTTTTTTTTTGG
GTTATTTCGGTTATTTTCTTTTTTTTTTTTTTCTTGGTTTCGGGGGTTTTATCATGTTGGTGTTTTA
ATTGTTTTTTTTTTTTATGTTGTTTTTTTTTGTTTTTTTAGATTTTGTTATTTTTTGTTTTTTTTTTAT
TATTGTTTTTGAGTTTTATTAATTTTTGTGTTGTTGACTATATTGTTTGGTGGAGGATTTTTGTTAT
TTGTACTTTTGTTTTTGTTTTTTTTTGTTGGTGGTGAGTTAGGTTTTGGGGGTTATTTGGTTAATTAT
TATGTTATTCAGGAAGTTTGTGGTTATTATTTTTTGGTTTTTGATGGTTGGAAGTTGCAATTTTTAT
TGCTTATGTTGAAGTCTGGTTCTTCTCCTTTTCATTTTTGACTTTTTAGTGTTTTGGGTGGTTTGGA
TAAGTGGTTTATTTTGTGGTTTTTAACTTTGCAAAAATTGCCTTATTTTGTTGTTTTGGTTAATTTT
TGTGGTGATTTTTTTTTTTTTGTTTTTGTTTTTTGGTATAATTTTTTGTTATTTTCAATTTTTTTTGT
TGCGTAGTTATCGTGATTTGTTAGTTGTGGGTTCTGCTGAATCTTTTAATTGGTTATTGTTATTGGG
TATTTTTTCTTTTAATGAAGTATTTGTTTTGTTTTTTTTTTTATTATTTTGTTATGTTTTTTTGTTGTC
TCTTATGTGTATGGGGGATTTTTAAATTTTTTGAGTTTAGAGATATTGATGTTTTTTTTTTAATGTTC
CTTTGAGAATTACTTTTTTTTTTAAAGGTGATTGTGTTGTTTGGTTCTTCTTTTTTTTGTTGGTTTTTA
TTATTTATTTTTGTTGTTGTTTATGCCTTTGATGTCTTTGGGTATGGGTTATTTGTTTTTTTTTGGTT
TCGATGATGAGTTTTAATTATGGTTTTAAGTATTATGATTATTTTGTTTATGTTTTGTTTTGTGTTG
GGTTGTTGTCTTGTTTTTAGTTGTTGTATTTATTTTTTTTTGTTGTTTTGTTGTTTTTTTTTAGTCC
TTTTTTATTTTTTGTTTTTTTTTATGTTTTTTGTTTTATATGGTTTTTTTGATTGTTCATGGTTTGGT
TGTTTGTTTTTTTTTGATTCTTTTAATTTTGTTTTTTTGTCTTTTATGAGTGTTTTTTGTTATGGGGT
TTATTTGTGTGTCTGAATTGTTGAGTGGTTTAGTTTTTTATAGTTGTCTTGTAGTGTTTTTTAGTGT
TTGTTTTTTTTATTCTGGTAGTTTTTTGATATTGTATGTTTTTTTATGAGTTGACTATAGTACCTATT
TTGTTTTGTTTGTTAGGGTATGGTCGTCAGGTGGAGAAGGTTAGGGCTTGTTATTATTTAATTTTTT
ATACTTTGTTTTTTGGGATGCCTTATTTGTTTTTGTATAGTCATGTTTTTTTTTTTTTGAATTTTGT
TTATTATGATTTTTTTTGTTTCTTATGAATTTATTTTTTTGTTGAGTTTGTGTTTTTTTAGTTAAGTTT
CCTGTTTATTTTTTTCATGTTTGATTACCTAAGGTTCATGTTGAGGCTCCTACTAGTGCTAGAATGA
TTTTGGCTGGTGTTATGTTGAAGTTGGGAGGAGCAGGTGTTTATCGTATTAGTAAGTCTTTGAATTT
TTTTGGTTTTGAAATGTTGATTTTTTTTTTCTTTGATTAGGATGGTTTTTTGTTCTTTTATTTGTGTT
GTTCAGAGTGATTGTAAGTCTTTGGCGGCTTATTCTTCTGTTTGTCATATGGGTTTTGTATTGCTCT
CTGAGATTAGTATGGTTTATTATGGTAAGTCTATGGCTTTGGTGATGATGTTGGCTCATGGTTATAC

TTCTGTTTTAATGTTTTATTTTATTGGTGAGTTTTATCATATTGCTAATAGGCGTTTAATTTATTAT
TTGCGTGGATATTTTAATGTTAGTATGTTGTTTTGTTTGATGTTTTGTTTGACTATGGTTTCTAATT
TTAGTTTTCCTGTGTCTATTTCTTTTTTTTTCTGAGTATTTGATGTTGAATTTTTTTAGGTCTGTTTT
TTATGTTGGTTTTTTGTTTTTGTTTTTTTATTATTTGGTTTCTTTTTATTATTCTGTTTATATTTTG
GTTTGTTTTTTAGTTGGGGATAAGGTGAGTTATGTTTGTGATGGTCGTAGTGTTGTTTGTTTACCTT
TGGTTTTTATGATATATAATTTTTTTTGGTTTATTTTTGTTATTTAATTTTAAGTTTGATTTTGGTT
TAGGTTGTATTAAGATAGTATTACTTATTTTTAGTTTATTTAATGTGTTATTTTTTGTACACTGGTA
GTTTTTTGATTGTTTTATTAACGTTCCAGAATAATCGGCTATGCGTTTTAATTTTTGACTCTATTTG
TTGTGGTGCTATGAGTTTTTAGTTTGTTTTTATGTTGTTTTTTGTAAAATATTTTGATTTTTTTTAG
TTTCTTGTGGTATCAAAAATTTGTTTTTTGAACTGGATTAGTACCCAGGTAATCAAAATTTAATAAT
TCGGGAGTAAAGTTTTGTTTAAACCGAAAAAATATTGACTGACTTTAGATTTTTCTTTGGAACATGT
GATTTGCTGGAGAGCCCTCTTTTTTGGTGAATTTTGTTGGCACATGTATGATTGTTTAGTTTTTATT
TTATTTTGTAATGCTTTGTTGTTTTGGCATTAAAAACAGATATATATTTGGCTTATGAATTTATGTT
TCATGTGTTACTATTATGAATTTTTTTTGGATTAGTTTTTTATTTTTTTTTGAAATTGGAAAAGAAA
GTAATTTTTTTTTAATGTTTTAATGAATTTAATAAATAAGGTGGTACAAACCATCCGTCAATGGCCT
AAAGGGGCGTAAGTTGTAGTATGGTAGAAGTAAGGAAACTTGTTTCTATTTTTTGAAGTTTTTTTGT
TTTTTTAAGTTTTATTATTTGGTATTGCATATCAGTAGAAGTTTTTATCATAGTTATGAGTAATAGA
ATTAAAATGGTTAAATTGAATTTGTTTTTTTTTTACGAAATTAATAATATTTTTATTTTAGTATTGA
TATAACGTATTTTTATTTCTGTTTATTTGTTATTTATAGGTTAAGTTATACTGTTTTTGTTTGTAGA
TTTTTAAATTTTTGTTGCTGTTTTATAAATGTTGTGTGTTTTACATAGATTTTTATTTTTTTTTTTT
TTTTTTTTTTGATTAATTTTTTAATTAATTTATGATTTTTACTTTAGTAATTTTTATTATTTTGAGG
GGTGTTTGTTTTTGAATTTATGTTTTTGAACTGGTTTTGTTGCTAAATGTTTATTAAAAACTTAGGT
TTTTATGTAAAATTGTCTTCTGCTCTATGAGTTTTTAAATGGCAGCCTTAGCGTGATGGCGTAAAAG
TAGCGTAAGTGATTTGTTTTTTTAATGGTTTCAAGTATGAATGAAGTTTTTAGCAGTTTTTTTATTT
ACTTTTTATTTGAATTATTTTTTTGATTAAAAATTATTAGTTAAGGTATTACAAAGATAAGTCTTCG
GAAATTTTGTTTTGAATTTTGAAATTTTTGTTTTTAATTTTTTCTTGGGGATGGATTTTAAGAAAGT
TTTATACTATTGTTATTATTAAAAATTACTCCGGAGTTAACAGGGTTGTAGACATATAAATAGATTT
TTATATTAGTGTGCTGCGCTACATCGATGTTGTATATTTTTTTTGATAATGGAGAGGTTTTTTTTAT
TTTGAGACTGTTCTTCTTGTATAAAAAATTGACTTGATATTAGTTTAGTTCGTCGTGAGACAGAGCG
GTTTATCTTGTGTATTTTTGGTTTTTGGCGGTGTTAGTACGAAAGGAATGCAATGTGGGTTTATATT
TATGACTTTTTTATTTTGATGGGTTTTT

>O.ochengiF4
ATTTTTTGTGGAATGACTTTTGGTAATGGTATGAAGCAGAGTATTATTAATACTGTGAATCATAAGA
CTATTGGTACTTATTATATTGTTTTAGGTTATTGGGCTGGTTTAGGTGGTTCTGTTTTATCTATGTT
GATTCGTTTTGAATTGTCTAGTCCTGGTGGTTATTTGTTTTTTGGAAGTGGTCAGGTTTATAATTCT
GTTCTTACTATGCATGGTGTTTTGATGATTTTTTTTTTTGGTTATGCCTATTTTGATTGGTGGCTTTG
GTAATTGGATGTTGCCTTTAATATTAGGGGCTCCTGAGATGGCGTTTCCTCGGGTAAATGCTTTATC
TTTTTGATTTACTTTTGTGGCTTTGTTGATAGTTTATCAGTCTTTTTTTATTGGGGGTGGCTCTGGT
AGAAGTTGGACTTTTTATCCTCCTCTTAGGGTTGAAGGTCAACCAGAATTGTCTTTAGATACTATGA
TTTTAGGTTTACATACTGTAGGAATTGGTTCTTTGTTGGGTGCTATTAATTTTATGGTAACTACTCA
GAATATACGGTCTACTGCTGTGACTTTGGATCAAATTAGTATGTTTGTTTGGACTTCTTATTTGACT
TCTTTTTTGTTAGTTTTGTCTGTGCCTGTTTTGGCTGGTTCTTTATTGTTTTTGTTGTTGGATCGTA
ATTTTAATACTTCTTTTTATGATACTAAGAAGGGGGGTAATCCTTTGTTGTATCAGCATTTGTTTTG
ATTTTTTGGTCATCCTGAGGTGTATGTTATTATTTTACCTGTTTTTGGTATTATTAGGGAAGCGGTT
TTATTTTTGACTGATAAGGATCGTTTGTTTGGTCAGACTAGGATAACTTTTGCTTCTATTTGGATTG
CTGTTTTAGGTACTTCTGTGTGAGGTCATCATATGTATACGGCTGGTTTGGATATTGATACTCGTAC
TTATTTTAGTGCTGCTACTATGATTATCGCTATTCCTAGAGCTGTTAAGATTTTTAATTGGTTAGGT
ACTTTTTTTGGTTCTAGTCAAAAGGTGCAGCCGTTATGATGTTGGACTTATAGTTTTATTTTTCTTT
TTACTGTGGGTGGATTAAGTGGAATTATTCTGAGGGCTGCTAGTTTGGATATTATTTTGCACGATAC
TTATTATGTTGTGGCTCATTTTCATTATACTTTGAGTTTGGGTGCTATTTATGGTATTTTTTGTGGT
TTTTGTTTGTGACTTCCTTATATGTATGGTATTTCTTTTGATAGGGTTATGATAATAGCTGTTTTTG

TTTGTTTTTTTGTTGGTACTAATATGACATTTTTTCCTATGCATTTTGCTGGTTTGCAGGGTATGCC
TCGTAAGATTTTGGATTATCCTGATTGTTATTCTACTTTTCAGATTATTTCTTCTTTAGGTTCTGTT
ATTACTTTTGTTGGTTTTGTTTTGTTTAATTATTTGTTGGTTGATTCTATTTTTTTTTCTCGTTTTT
TGGGGGTTTCTTTTTATAATTATCATAGTCCGGCTTATGCTTTAAATGTTCCTCCTTTGCCGGATTC
TTTTACTGAAGAGGCTTTTATTATAGGTCTTCATTGGAAGATTATTAGTAAGGATACTCCTTCTTAT
AGGTATCGTCGGGTTGGTTATGGTTATCATAGTAAGTAAATTTTTTTTTATGTTAGGGTATTTTTTG
CTTTTGTTTTTTTTTGTTTGAGTTTTTTGGATTGGGATCCGTTGAAGAGTTGTGTTATGATGTGTTT
GGGTATTATGTCTATAAGTTGTTATGTTTCTTTGGGTATTCATGTATGATATTCTTATTTTGTTGTT
TTAATTTTTTTTAGTGGTATTTTTTCTTTGTTGACTTATTTTTGTAGTATGAGTAATTTTGTTTTTT
ATTATAATTATTTTTTTTTTTTTTTCTTTGTTTTTGGTTAGTTTTTTTTTTGTATTTGTTGTAGATTT
TGATTTTTTTTTGTTTTTTTGTGATTTTAATTTTCTTTATGTTTGTTATGATTTTAGTTATTATTAT
GTTTTTTGAGTAGTTTTTGTTTTGTTTTTGTTTTTGGTTTTGGTTAGATTTAGGTTTAATGGTTTTG
GTTATATGCGTAGCTTGTAGATTGTTATTTTTAATTCTTTAGTTTTTTTGCCTGCTAGTTTTACTTT
GAGTTATATGTGAAATTTTGGTAGTATGTTGGGTATTATGTTGATGTCTCAGATTTTAACTGGTTTT
TTTTTGACTTTTTACTATACGGCTGGGGAAGCTTTTAGGTCTGTTCAGTATATTATGTTTGAGGTTA
ATTTGGGTTGGTTGTTGCGTATTATGCATTCTAATGGGGCTTCTATGTTTTTTTTGTTTATTTATTT
ACATATTTTTAAGGGTCTGATTTATGGTAGATATCGTCTTATTGGTGTATGGTTGAGTGGTATTTTT
ATTTATTTTTTATTGATAGGTATTGCTTTTACTGGTTATGTTTTGATTTGAGGTCAAATAAGTTATT
GGGCGGCAGTAGTTATTACTAGTTTAATAACTTCTGTTCCTTATTTAGGTAAGTATTTAGTTTGATG
GATTTGGGGGAGTTTTAGTGTTTGTGAGAACACTTTAAAGTTTTTTTATTCTGTTCATTTTATTTTA
CCTTGGTCTTTGATAGTTTTAGTTGTTTTTCATTTGTTTTTTTTGCATTTTACTGGTTCTAGTTCTA
GTTTGTATTGTCACGGGGATTATGATAAGATTCATTTTTTTCCTAGTTTTTGATTGAAGGATGGTTT
TGATATTTTTTTTTATTTTTTTTTGATTTTGTTTAGACTTTATTTTTCTTTTGATTTAAGTGATCCT
ATGATTTTTGTGGAGTCTGATTCTATGGCTAGTCCTGCGCATGTTGTACCTGAGTGATATTTTTTAT
TTGCTTTTACTATCTTACGTTCTGTTCCTAGTAAGTTATTAGGGGTTATTTTAATATTTAGTTCTGT
TTTTGTATTGACTATTCTTGTTTGACCTGATAGTTATCAGTCTATTTTGGATAATTTTTTATATTTT
TTTGTTATGTGTTTTGTTTGGATTTTTTTTTGGTTAACTTGAGCTGGTCATTATCCTACTGATTATC
CTTTTAACTATTTTAATTTGTTTTGTACTTTTTTTTATTTTTGTTGTATTTTTTTTGTTTGTTTAAT
TAATTTTTTTAGTGATAAGTTGTTTAGTTAAATTTTGTTGAAGTTTCGTAAGTACCATAAAATGGAG
TATAGTTATTATCCTTTGATAGTTGGGGCGGGTATTTTAGGTTTTGATGTTAGTTTGGTTTTATTTA
TAAGTATAGGTATGTTTTATTCTATTTTTATTTGTTTTTTGTATTTGGTTTATGTTTTTTTTTTTGTG
AATCAAGGATGTTATTTTAGAGGACATTAGTGGTCAATATTCTTTTTATGACTATCGTATGTTTAAT
CAAGGTTTTCGTTTGTTTCTTTTTAGTGAGTTAACTTTGTTTGTTTCTATTTTTTGAACTTTTTTGG
ATACAGCTTTGTGTCCTTTAACTTGGTTAGGTGGGGTTTGGTCTCCATTTGGGATTTTATCTCCTGA
TTATTTGGGTTTGAATGGTATGGCTAGTTTGTTTTTAATGATAAATAGGCAAGTTTTGAAGTATTCT
CGTCGTTATTTGTGTTTGAGTAGTTCTAAGTGTGAAGAGTTTTTGTTAGTTTGTATTTTTGTTGGAG
TTGGTTTTTTATGTTTTCAGTTTTATGAATATAATAACAATTCGTTTGTTATGAGTGATAGTGTTTA
TGGTAGTATTTTTTATATGGGTACTGGTTTACATGGTTTGCATGTTTTTATTGGTGTTTGTTTTCTT
ATTGTTAATTTTTTTCGTGTTAAGTTGTTCAATTTTAATTGATATCATGTTCAGGCTTATGATATAT
CTATTGATTATTGGCGTTTTTTAGAATGAATGTGAGGTGTTATGTTTTGTTTATTATATGTTTGAGG
TTCTTAATTGGTTATTTTTTATTATTTGGGGTTGTTGGTTATGATTGTTTTTATTTTGCAGGCTATT
GCTTTTTTAACTTTGTTGGAGCGCCATTTTTTGGGTGGTTCTCAGTGTCGTGTTGGTCCTAATAAGG
TGGGTTATTCTGGTGTTTTGCAGGCTTTATTTGATGGTTTAAAGTTGTTAAAGAAGGAGCAGTTGTT
GTTGTGTTTTTCTTCTTGATTATCTTTTTTGTTTATGCCTGTTTGTGGTTTTGTTTTGATGGTTTTT
TTTTGATTTACTTTGCCTTATTTTTTTTTCTTTTTTGTCTTTTGAGTACTCTGGTGTTTTTTTGTTTT
GTCTTATAGGGGTTTCTGTTTATTTTATTATGCTTTCTGGTGTTTTTAGCGGTAGTAAATATTCTTT
TGTTGGTGGATTGCGTGCTTGCGTTCAGAGTTATTCTTATGAGATTGCTTTTTCTATTTATTTGTTG
GTTTTTTTGTTGTTTAATAAGGGTCTATGTTTGTCTTTTAGTTTTTGTTTATTTTTTTTTTTTGTTTT
TTTTTCCTTTTTTTTGTTTAGTTCTTGTTGATTTGCATCGGGCTCCTTTTGATTTTTCTGAGTGTGA
AAGTGAGTTGGTAAGGGGGTTTAATGTTGAGTATTCGGGAGTTGGTTTTGCTGCTTTGTTTTTAGGG
GAGTATGGTAATTTACTTTATTTTGGTTGTTTGACTTCTAGTTTGTTTTTTGGTATAAGTTTTTTTT
TTTTTTATTTTATTGTATGTATGATTGTTTTTTCTCGTAGAGCTTATCCTCGTTTTCGTTTTGATAA

GTTGATGGGTGTTTGTTGGTTTTTGTTTTTGCCTGTCGGTTTTTATTTTTTTGGTTTGTCTTTTGTT
GTTTTTATGTTGTGCTTATTTAGTTTAATTTTTTAGATTTTGTTATTTTTTTTTTATTTGGTTTTGAT
TAGTTTTTTATTTGTTTTATATGGAGTTGAGTAAGTTTAGTAGTTTGGGAGTTTTAGGTGTTTTTGT
TAATGTTTTAGTTTCTGGATTTTCTCATCAGGGTTTTCAGTCTAGTGTTTTTTTTAAGTTTGTTGTT
TTTTTTCTTTTGGTTTTTTGAATGAGTGGGTTATTGTTTCCTTTGTTTAGTCCTTGGGCTTGTGTGG
GTTTTTTGTTTTTTGTTACTAATTTTTCTTGATTGGGTGTTCGTACTTTTATTTTAGCTGTAGATAG
TTTTTTGATTTTTTTTGAGGGGGATCACTCTTGGGAGTGGTTTTCTAGGTTAGTTATGTTTTTTTCT
CATTGGTTGAGATTTTTGATAAGGGGGGTGGCTTTAACTTTGCGTATTAGTATTATTTTTTTAATTG
GTCATTTTTTAATGTTTACTGTTTTGGATATGAGTGTATTTTATTCTTTGTTTTTTTTGTTGTTTGT
GGTTCCGGTGGAGTTGTTTTTTGCTTTTTTACAGAGTTATATTTTTTTGACTTTGGTTTGTATGTTT
TTACTTAATATGATTTAGATTTATTTGCAGAATTATGTGTTTCCTATTCCTGGAAATTCTTATGTGT
TTTGTTGTTATTATATCCATAATTATTATTCTCATATTATTTTTTTTGGTTTTTTTGTGATGTTTTT
GGTTAGTGGTGGGGTTTATTTTTTTGGCAATTCTTTTAAGTTTAACTTGAAGCGGAGAGATAGTCGT
ATAATTGAATTAGTTTTACAGGTGTTGATTGTTAATTTTTTGATTATGATGGCAGGTCCTGGTTTTT
GGTTGATTCAGTATCAGGGACGTATGTTTCGTCAATCTGAGTTGGCTTTGAAGGTTATTGGTCATCA
ATGGTATTGGAGTTATGAGTATGGTGATAGTGGAAAATTATGTTTTGATTCATTTATGAAGTCTTTA
GATGATTTGTCTTTAGGGGATTTTCGGTTATTTGATGTTGATAATCGGTGTGTTTTGCCTGTAGGTG
TGAATGTTGGAGTGTATTGTACTTCTAGTGATGTTATTCATTCTTTTGCTATTCCTAAGTGTTTTAT
TAAGATGGATGCTTTGAATGGTTTGTTAACTAAGGTTACTTGTAATTTTTCTTGTTCTGGTTTGTTT
TTTGGGCAGTGTTCTGAAATTTGTGGTGCTAATCATAGGTTTATGCCTATTGTGTTGGAGTTGACTT
CTTTGGAGTGTTGGAAGGGTTGATCAGTTAATTATTTGCTGGGTTAACTTTTTTTAAATTTTATTGT
TATTTTTTTTTTTTCTTTTTTGGTTCCTTTTGGTATGTATTTGTTGTCTTTTTTTGTGTCTTTTAAG
GATTTTTATGGTGCTAAATTAAGTTCTTATGAATGTGGTTTTGATGTTGTGAAGAAGGTTCATGTTG
GTTTTAATTTGGTTTTTTTTTCTATTGTTTTGTTGTTTGTTGTTTTTGAGTTGGAAGTTTTAATTTT
TATTATTTTGATTCAGGGTGATTTTTATAGTTTATTGTCTTTTTTTTGTTTTTTTTTTTATGTTGTT
TTTAGTTTTTATATGGAGTGGTATTTTGGTAAGTTGATTTGGTTTTGTTAGTTGTTATTGTTTGTTT
GATATGTTCTTATTTTTTATTTTTTTTTTGGTTGTTGTTTTTTTGTTTGTTCCTTATGGTAAATGGAG
TTATAGTTTTGGTTTTAGTGATTATTTTAATTTTACTTTTGTTTATAATTTTGAAGTTTGTTTGTTT
TTTTTAGTTTTGTTGTTGGTTTCTTTTATGGTTTTTGTTTATGGTTCTTTTTATATGGTTGGGGTTT
CTCGTTTGTTTTATTTTTTTTTTTTTTTATTTTTGTTTGTGTTGAGGATGGGTGGCTTGATTGTTTT
TAGAGGTAGTATTGTTTTAACTTTGGTTTTTTGGGATTTTTTGGGGGTTAGTAGTTTTTTTTTGGTT
TTGTTTTATGGTAATGTTAGTGCTCGAAGGGGTGCTATGAGTACTGTGTTTACTAATCGTATTGGTG
ATTTTTGTATTTTTTTTGTTTTTTAATGGTTTTGTTTTGTTTTCTATGAGTTTTTTGTCTTATCAGTT
TTTTGGTTCTTTGTTAGTTTTTATGTTGTTTGTTTCTTCTGTTATTAAGGGTGGTCAGTATCCTTTT
GGTAGCTGGTTGCCTAAGGCTATGGCTGCTCCTACTCCTGTTAGCTGTTTGGTTCATAGTAGTACTT
TAGTTACTGCTGGTGTTATGTTGATGGATTGTTATGTTTATATTTCTTTGAATTCTGATGTTTTGTC
TTTTGTTTTTTATGTTGGTTTTTTTACTATAGTTTTTTCTGGTTTTTGTGCTTTGGTGGAGGAGGAT
GCTAAGAAGATTGTTGCTTTGAGTACTATGTCTCAGATTGGTTTTTGTTTTTTGGCTATTGGTAGGG
GTTTGCATTATTTGTCTTATGTTCATATGATTAGGCATTCTTTTTTTAAGAGATTGTTGTTTATGCA
GATAGGTTATTTGATTTTTATTAATTTTGGTCAACAGGATTATCGTGGTTATTCTTTTTTTGGTTTT
TGTGCTCCGGTTTTAGTTCAGTTGCAGATTTTTTTATCTGTGTTTTGTTTGTGTGGTTTGTTGTTTA
CTAGAGGTAGTTGTAGTAAGGAATATTTTATATCTCGTTTTTATTATGATTCTTATGGTTTTTTTTT
AGTTTTTTTTTATTTTTTTGGTGTGTTTTTGACTTTTTGTTATTGTTATCGGATGTTTTTTTTTGTTT
CGTGTGGGGGCTTTTGGTTTTGATTATGTGGGTTTTTCTAGTAAGTTANTTTTATTTTTCTTGTTTT
TTTTTGGTTTTTTTTTTCTGTTGTTTTTACTTTTTGGTGGGTTTTTGGTTTGTTATCTTTTTCTGCGG
CTTTTAATCGTTTTGAGTTTTTGGTTGTTTATTTTTATTTGTTTTTTGTTTATTGTTTTTGTGGTTA
TTTTTTTCGTTATTTTTTTTTTCGTTATTTTGTGAAATTTTTTATGGATCATTATGCTTGTTTTATTT
ATAAAATTTTTCCTAGTTTTTTTTTATTTTGATGTTTTTATTATGGGTTTTAATTATTTTTTTTTTGG
GTTATTTCGGTTATTTTCTTTTTTTTTTTTTTTCTTGGTTTCGGGGGTTTTATCATGTTGGTGTTTTA
ATTGTTTTTTTTTTTTATGTTGTTTTTTTTTGTTTTTTTAGATTTTGTTATTTTTTGTTTTTTTTTTAT
TATTGTTTTTGAGTTTTATTAATTTTTGTGTTGTTGACTATATTGTTTGGTGGAGGATTTTTGTTAT
TTGTACTTTTGTTTTTGTTTTTTTTGTTGGTGGTGAGTTAGGTTTTGGGGGTTATTTGGTTAATTAT

TATGTTATTCAGGAAGTTTGTGGTTATTATTTTTTGGTTTTTGATGGTTGGAAGTTGCAATTTTTAT
TGCTTATGTTGAAGTCTGGTTCTTCTCCTTTTCATTTTTGACTTTTTAGTGTTTTGGGTGGTTTGGA
TAAGTGGTTTATTTTGTGGTTTTTAACTTTGCAAAAATTGCCTTATTTTGTTGTTTTGGTTAATTTT
TGTGGTGATTTTTTTTTTTTGTTTTTGTTTTTTGGTATAATTTTTTGTTATTTTCAATTTTTTTTGT
TGCGTAGTTATCGTGATTTGTTAGTTGTGGGTTCTGCTGAATCTTTTAATTGGTTATTGTTATTGGG
TATTTTTTCTTTTAATGAAGTATTTGTTTTGTTTTTTTTTTATTATTTTGTTATGTTTTTTGTTGTC
TCTTATGTGTATGGGGGATTTTTAAATTTTTTGAGTTTAGAGATATTGATGTTTTTTTTTTAATGTTC
CTTTGAGAATTACTTTTTTTTTAAAGGTGATTGTGTTGTTTGGTTCTTCTTTTTTTGTTGGTTTTTA
TTATTTATTTTTGTTGTTGTTTATGCCTTTGATGTCTTTGGGTATGGGTTATTTGTTTTTTTTTGGTT
TCGATGATGAGTTTTAATTATGGTTTTAAGTATTATGATTATTTTGTTTATGTTTTGTTTTGTGTTG
GGTTGTTGTCTTGTTTTTAGTTGTTGTATTTATTTTTTTTTTGTTGTTTTGTTGTTTTTTTTTTAGTCC
TTTTTTATTTTTTGTTTTTTTTTATGTTTTTTGTTTTATATGGTTTTTTTGATTGTTCATGGTTTGGT
TGTTTGTTTTTTTTTGATTCTTTTAATTTTGTTTTTTTGTCTTTTATGAGTGTTTTTGTTATGGGGT
TTATTTGTGTGTCTGAATTGTTGAGTGGTTTAGTTTTTTATAGTTGTCTTGTAGTGTTTTTTAGTGT
TTGTTTTTTTTTATTCTGGTAGTTTTTTGATATTGTATGTTTTTTATGAGTTGACTATAGTACCTATT
TTGTTTTGTTTGTTAGGGTATGGTCGTCAGGTGGAGAAGGTTAGGGCTTGTTATTATTTAATTTTTT
ATACTTTGTTTTTTGGGATGCCTTATTTGTTTTTGTATAGTCATGTTTTTTTTTTTTTTGAATTTTGT
TTATTATGATTTTTTTGTTTCTTATGAATTTATTTTTTTGTTGAGTTTGTGTTTTTTAGTTAAGTTT
CCTGTTTATTTTTTTCATGTTTGATTACCTAAGGTTCATGTTGAGGCTCCTACTAGTGCTAGAATGA
TTTTGGCTGGTGTTATGTTGAAGTTGGGAGGAGCAGGTGTTTATCGTATTAGTAAGTCTTTGAATTT
TTTTGGTTTTGAAATGTTGATTTTTTTTTTCTTTGATTAGGATGGTTTTTTGTTCTTTTATTTGTGTT
GTTCAGAGTGATTGTAAGTCTTTGGCGGCTTATTCTTCTGTTTGTCATATGGGTTTTGTATTGCTCT
CTGAGATTAGTATGGTTTATTATGGTAAGTCTATGGCTTTGGTGATGATGTTGGCTCATGGTTATAC
TTCTGTTTTAATGTTTTATTTTATTGGTGAGTTTTATCATATTGCTAATAGGCGTTTAATTTATTAT
TTGCGTGGATATTTTAATGTTAGTATGTTGTTTTGTTTGATGTTTTGTTTGACTATGGTTTCTAATT
TTAGTTTTCCTGTATCTATTTCTTTTTTTTCTGAGTATTTGATGTTGAATTTTTTTAGGTCTGTTTT
TTATGTTGGTTTTTTGTTTTTGTTTTTTTATTATTTGGTTTCTTTTTATTATTCTGTTTATATTTTG
GTTTGTTTTTTAGTTGGGGATAAGGTGAGTTATGTTTGTGATGGTAGTAGTGTTGTTTGTTTACCTT
TGGTTTTTATGATATATAATTTTTTTTGGTTTATTTTTGTTATTTAATTTTAAGTTTGATTTTGGTT
TAGGTTGTATTAAGATAGTATTACTTATTTTTAGTTTATTTAATGTGTTATTTTTTGTACACTGGTA
GTTTTTTGATTGTTTTATTAACGTTCCAGAATAATCGGCTATGCGTTTTAATTTTTGACTCTATTTG
TTGTGGTGCTATGAGTTTTTAGTTTGTTTTTATGTTGTTTTTTGTAAAATATTTTGATTTTTTTTAG
TTTCTTGTGGTATCAAAAATTTGTTTTTTGAACTGGATTAGTACCCAGGTAATCAAAATTTAATAAT
TCGGGAGTAAAGTTTTGTTTAAACCGAAAAAATATTGACTGACTTTAGATTTTTCTTTGGAACATGT
GATTTGCTGGAGAGCCCTCTTTTTTGGTGAATTTTGTTGGCACATGTATGATTGTTTAGTTTTTATT
TTATTTTGTAATGCTTTGTTGTTTTGGCATTAAAAACAGATATATATTTGGCTTATGAATTTATGTT
TCATGTGTTACTATTATGAATTTTTTTTGGATTAGTTTTTTATTTTTTTTTTGAAATTGGAAAAGAAA
GTAATTTTTTTTTAATGTTTTAATGAATTTAATAAATAAGGTGGTACAAACCATCCGTCAATGGCCT
AAAGGGGCGTAAGTTGTAGTATGGTAGAAGTAAGGAAACTTGTTTCTATTTTTTGAAGTTTTTTTGT
TTTTTTAAGTTTTATTATTTGGTATTGCATATCAGTAGAAGTTTTTATCATAGTTATGAGTAATAGA
ATTAAAATGGTTAAATTGAATTTGTTTTTTTTTTTACGAAATTAATAATATTTTTATTTTAGTATTGA
TATAACGTATTTTTATTTCTGTTTATTTGTTATTTATAGGTTAAGTTATACTGTTTTTGTTTGTAGA
TTTTTAAATTTTTGTTGCTGTTTTATAAATGTTGTGTGTTTTACATAGATTTTTATTTTTTTTTTTT
TTTTTTTTTTGATTAATTTTTTAATTAATTTATGATTTTTACTTTAGTAATTTTTATTATTTTGAGG
GGTGTTTGTTTTTGAATTTATGTTTTTGAACTGGTTTTGTTGCTAAATGTTTATTAAAAACTTAGGT
TTTTATGTAAAATTGTCTTCTGCTCTATGAGTTTTTAAATGGCAGCCTTAGCGTGATGGCGTAAAAG
TAGCGTAAGTGATTTGTTTTTTTTAATGGTTTCAAGTATGAATGAAGTTTTTAGCAGTTTTTTTATTT
ACTTTTTATTTGAATTATTTTTTTGATTAAAAATTATTAGTTAAGGTATTACAAAGATAAGTCTTCG
GAAATTTTGTTTTGAATTTTGAAATTTTTGTTTTTAATTTTTTCTTGGGGATGGATTTTAAGAAAGT
TTTATACTATTGTTATTATTAAAAATTACTCCGGAGTTAACAGGGTTGTAGACATATAAATAGATTT
TTATATTAGTGTGCTGCGCTACATCGATGTTGTATATTTTTTTTGATAATGGAGAGGTTTTTTTTAT
TTTGAGACTGTTCTTCTTGTATAAAAAATTGACTTGATATTAGTTTAGTTCGTCGTGAGACAGAGCG

GTTTATCTTGTGTATTTTTGGTTTTTGGCGGTGTTAGTACGAAAGGAATGCAATGTGGGTTTATATT
TATGACTTTTTTATTTTGATGGGTTTTT

>O.ochengiF6
ATTTTTTGTGGAATGACTTTTGGTAATGGTATGAAGCAGAGTATTATTAATACTGTGAATCATAAGA
CTATTGGTACTTATTATATTGTTTTAGGTTATTGGGCTGGTTTAGGTGGTTCTGTTTTATCTATGTT
GATTCGTTTTGAATTGTCTAGTCCTGGTGGTTATTTGTTTTTTGGAAGTGGTCAGGTTTATAATTCT
GTTCTTACTATGCATGGTGTTTTGATGATTTTTTTTTTGGTTATGCCTATTTTGATTGGTGGCTTTG
GTAATTGGATGTTGCCTTTAATATTAGGGGCTCCTGAGATGGCGTTTCCTCGGGTAAATGCTTTATC
TTTTTGATTTACTTTTGTGGCTTTGTTGATAGTTTATCAGTCTTTTTTTATTGGGGGTGGCCCTGGT
AGAAGTTGGACTTTTTATCCTCCTCTTAGGGTTGAAGGTCAACCAGAATTGTCTTTAGATACTATGA
TTTTAGGTTTACATACTGTAGGAATTGGTTCTTTGTTGGGTGCTATTAATTTTATGGTAACTACTCA
GAATATACGGTCTACTGCTGTGACTTTGGATCAAATTAGTATGTTTGTTTGGACTTCTTATTTGACT
TCTTTTTTGTTAGTTTTGTCTGTGCCTGTTTTGGCTGGTTCTTTATTGTTTTTGTTGTTGGATCGTA
ATTTTAATACTTCTTTTTATGATACTAAGAAGGGGGGTAATCCTTTGTTGTATCAGCATTTGTTTTG
ATTTTTTGGTCATCCTGAGGTGTATGTTATTATTTTACCTGTTTTTGGTATTATTAGGGAAGCGGTT
TTATTTTTGACTGATAAGGATCGTTTGTTTGGTCAGACTAGGATAACTTTTGCTTCTATTTGGATTG
CTGTTTTAGGTACTTCTGTGTGAGGTCATCATATGTATACGGCTGGTTTGGATATTGATACTCGTAC
TTATTTTAGTGCTGCTACTATGATTATCGCTATTCCTAGAGCTGTTAAGATTTTTAATTGGTTAGGT
ACTTTTTTTGGTTCTAGTCAAAAGGTGCAGCCGTTATGATGTTGAACTTATAGTTTTATTTTTCTTT
TTACTGTGGGTGGATTAAGTGGAATTATTCTGAGGGCTGCTAGTTTGGATATTATTTTGCACGATAC
TTATTATGTTGTGGCTCATTTTCATTATACTTTGAGTTTGGGTGCTATTTATGGTATTTTTTGTGGT
TTTTGTTTGTGACTTCCTTATATGTATGGTATTTCTTTTGATAGGGTTATGATAATAGCTGTTTTTG
TTTGTTTTTTTGTTGGTACTAATATGACATTTTTTCCTATGCATTTTGCTGGTTTGCAGGGTATGCC
TCGTAAGATTTTGGATTATCCTGATTGTTATTCTACTTTTCAGATTATTTCTTCTTTAGGTTCTGTT
ATTACTTTTGTTGGTTTTGTTTTGTTTAATTATTTGTTGGTTGATTCTATTTTTTTTTCTCGTTTTT
TGGGGGTTTCTTTTTATAATTATCATAGTCCGGCTTATGCTTTAAATGTTCCTCCTTTGCCGGATTC
TTTTACTGAAGAGGCTTTTATTATAGGTCTTTATTGGAAGATTATCAGTAAGGATACTCCTTCTTAT
AGGTATCGTCGGGTTGGTTATGGTTATCATAGTAAGTAAATTTTTTTTTATGTTAGGGTATTTTTTG
CTTTTGTTTTTTTTTGTTTGAGTTTTTTGGATTGGGATCCGTTGAAGAGTTGTGTTATGATGTGTTT
GGGTATTATGTCTATAAGTTGTTATGTTTCTTTGGGTATTCATGTATGATATTCTTATTTTGTTGTT
TTAATTTTTTTTAGTGGTATTTTTTCTTTGTTGACTTATTTTTGTAGTATGAGTAATTTTGTTTTTT
ATTATAATTATTTTTTTTTTTTTTTCTTTGTTTTTGGTTAGTTTTTTTTTTGTATTTGTTGTAGATTT
TGATTTTTTTTTGTTTTTTTGTGATTTTAATTTCTTTATGTTTGTTATGATTTTAGTTATTATTAT
GTTTTTTGAGTAGTTTTTGTTTTGTTTTTGTTTTTGGTTTTGGTTAGATTTAGGTTTAATGGTTTTG
GTTATATGCGTAGCTTGTAGATTGTTATTTTTAATTCTTTAGTTTTTTTGCCTGCTAGTTTTACTTT
GAGTTATATGTGAAATTTTGGTAGTATGTTGGGTATTATGTTGATGTCTCAGATTTTAACTGGTTTT
TTTTTGACTTTTTACTATACGGCTGGGGAAGCTTTTAGGTCTGTTCAGTATATTATGTTTGAGGTTA
ATTTGGGTTGGTTGTTGCGTATTATGCATTCTAATGGGGCTTCTATGTTTTTTTTGTTTATTTATTT
ACATATTTTTAAGGGTCTGATTTATGGTAGATATCGTCTTATTGGTGTATGGTTGAGTGGTATTTTT
ATTTATTTTTTATTGATAGGTATTGCTTTTACTGGTTATGTTTTGATTTGAGGTCAAATAAGTTATT
GGGCGGCAGTAGTTATTACTAGTTTAATAACTTCTGTTCCTTATTTAGGTAAGTATTTAGTTTGATG
GATTTGGGGGAGTTTTAGTGTTTGTGAGAACACTTTAAAGTTTTTTTATTCTGTTCATTTTATTTTA
CCTTGGTCTTTGATAGTTTTAGTTGTTTTTCATTTGTTTTTTTTGCATTTTACTGGTTCTAGTTCTA
GTTTGTATTGTCACGGGGATTATGATAAGATTCATTTTTTTCCTAGTTTTTGATTGAAGGATGGTTT
TGATATTTTTTTTTTATTTTTTTTTTGATTTTGTTTAGACTTTATTTTTCTTTTGATTTAAGTGATCCT
ATGATTTTTGTGGAGTCTGATTCTATGGCTAGTCCTGCGCATGTTGTACCTGAGTGATATTTTTTTAT
TTGCTTTTACTATCTTACGTTCTGTTCCTAGTAAGTTATTAGGGGTTATTTTAATATTTAGTTCTGT
TTTTGTATTGACTATTCTTGTTTGACCTGATAGTTATCAGTCTATTTTGGATAATTTTTTATATTTT
TTTGTTATGTGTTTTGTTTGGATTTTTTTTTGGTTAACTTGAGCTGGTCATTATCCTACTGATTATC
CTTTTAACTATTTTAATTTGTTTTGTACTTTTTTTTTATTTTTGTTGTATTTTTTTTTGTTTGTTTAAT
TAATTTTTTTAGTGATAAGTTGTTTAGTTAAATTTTGTTGAAGTTTCGTAAGTACCATAAAATGGAG

TATAGTTATTATCCTTTGATAGTTGGGGTGGGTATTTTAGGTTTTGATGTTAGTTTGGTTTTATTTA
TAAGTATAGGTATGTTTTATTCTATTTTTATTTGTTTTTTGTATTTGGTTTATGTTTTTTTTTTGTG
AATCAAGGATGTTATTTTAGAGGACATTAGTGGTCAATATTCTTTTTATGACTATCGTATGTTTAAT
CAAGGTTTTCGTTTGTTTCTTTTTAGTGAGTTAACTTTGTTTGTTTCTATTTTTTGAACTTTTTTGG
ATACAGCTTTGTGTCCTTTAACTTGGTTAGGTGGGGTTTGGTCTCCATTTGGGATTTTATCTCCTGA
TTATTTGGGTTTGAATGGTATGGCTAGTTTGTTTTTAATGATAAATAGGCAAGTTTTGAAGTATTCT
CGTCGTTATTTGTGTTTGAGTAGTTCTAAGTGTGAAGAGTTTTTGTTAGTTTGTATTTTTGTTGGAG
TTGGTTTTTTATGTTTTCAGTTTTATGAATATAATAACAATTCGTTTGTTATGAGTGATAGTGTTTA
TGGTAGTATTTTTTATATGGGTACTGGTTTACATGGTTTGCATGTTTTTATTGGTGTTTGTTTTCTT
ATTGTTAATTTTTTTCGTGTTAAGTTGTTCAATTTTAATTGATATCATGTTCAGGCTTATGATATAT
CTATTGATTATTGGCGTTTTTTAGAATGAATGTGAGGTGTTATGTTTTGTTTATTATATGTTTGAGG
TTCTTAATTGGTTATTTTTTATTATTTGGGGTTGTTGGTTATGATTGTTTTTATTTTGCAGGCTATT
GCTTTTTTAACTTTGTTGGAGCGCCATTTTTTGGGTGGTTCTCAGTGTCGTGTTGGTCCTAATAAGG
TGGGTTATTCTGGTGTTTTGCAGGCTTTATTTGATGGTTTAAAGTTGTTAAAGAAGGAGCAGTTGTT
GTTGTGTTTTTCTTCTTGATTATCTTTTTTGTTTATGCCTGTTTGTGGTTTTGTTTTGATGGTTTTT
TTTTGATTTACTTTGCCTTATTTTTTTTCTTTTTTGTCTTTTGAGTACTCTGGTGTTTTTTTGTTTT
GTCTTATAGGGGTTTCTGTTTATTTTATTATGCTTTCTGGTGTTTTTAGCGGTAGTAAATATTCTTT
TGTTGGTGGATTGCGTGCTTGCGTTCAGAGTTATTCTTATGAGATTGCTTTTTCTATTTATTTGTTG
GTTTTTTTGTTGTTTAATAAGGGTCTATGTTTGTCTTTTAGTTTTTGTTTATTTTTTTTTTTTGTTTT
TTTTTCCTTTTTTTTGTTTAGTTCTTGTTGATTTGCATCGGGCTCCTTTTGATTTTTCTGAGTGTGA
AAGTGAGTTGGTAAGGGGGTTTAATGTTGAGTATTCGGGAGTTGGTTTTGCTGCTTTGTTTTTAGGG
GAGTATGGTAATTTACTTTATTTTGGTTGTTTGACTTCTAGTTTGTTTTTTGGTATAAGTTTTTTTT
TTTTTTATTTTATTGTATGTATGATTGTTTTTTCTCGTAGAGCTTATCCTCGTTTTCGTTTTGATAA
GTTGATGGGTGTTTGTTGGTTTTTGTTTTTGCCTGTCGGTTTTTATTTTTTTGGTTTGTCTTTTGTT
GTTTTTATGTTGTGCTTATTTAGTTTAATTTTTTAGATTTTGTTATTTTTTTTTTATTTGGTTTTGAT
TAGTTTTTTATTTGTTTTATATGGAGTTGAGTAAGTTTAGTAGTTTGGGAGTTTTAGGTGTTTTTGT
TAATGTTTTAGTTTCTGGATTTTCTCATCAGGGTTTTCAGTCTAGTGTTTTTTTTTAAGTTTGTTGTT
TTTTTTCTTTTGGTTTTTTGAATGAGTGGGTTATTGTTTCCTTTGTTTAGTCCTTGGGCTTGTGTGG
GTTTTTTGTTTTTTGTTACTAATTTTTCTTGATTGGGTGTTCGTACTTTTATTTTAGCTGTAGATAG
TTTTTTGATTTTTTTTGAGGGGGATCACTCTTGGGAGTGGTTTTCTAGGTTAGTTATGTTTTTTTCT
CATTGGTTGAGATTTTTGATAAGGGGGGTGGCTTTAACTTTGCGTATTAGTATTATTTTTTTAATTG
GTCATTTTTTAATGTTTACTGTTTTGGATATGAGTGTATTTTATTCTTTGTTTTTTTTGTTGTTTGT
GGTTCCGGTGGAGTTGTTTTTTGCTTTTTTACAGAGTTATATTTTTTTGACTTTGGTTTGTATGTTT
TTACTTAATATGATTTAGATTTATTTGCAGAATTATGTGTTTCCTATTCCTGGAAATTCTTATGTGT
TTTGTTGTTATTATATCCATAATTATTATTCTCATATTATTTTTTTTGGTTTTTTTGTGATGTTTTT
GGTTAGTGGTGGGGTTTATTTTTTTGGCAATTCTTTTAAGTTTAACTTGAAGCGGAGAGATAGTCGT
ATAATTGAATTAGTTTTACAGGTGTTGATTGTTAATTTTTTGATTATGATGGCAGGTCCTGGTTTTT
GGTTGATTCAGTATCAGGGACGTATGTTTCGTCAATCTGAGTTGGCTTTGAAGGTTATTGGTCATCA
ATGGTATTGGAGTTATGAGTATGGTGATAGTGGAAAATTATGTTTTGATTCATTTATGAAGTCTTTA
GATGATTTGTCTTTAGGGGATTTTCGGTTATTTGATGTTGATAATCGGTGTGTTTTGCCTGTAGGTG
TGAATGTTGGAGTGTATTGTACTTCTAGTGATGTTATTCATTCTTTTGCTATTCCTAAGTGTTTTAT
TAAGATGGATGCTTTGAATGGTTTGTTAACTAAGGTTACTTGTAATTTTTCTTGTTCTGGTTTGTTT
TTTGGGCAGTGTTCTGAAATTTGTGGTGCTAATCATAGGTTTATGCCTATTGTGTTGGAGTTGACTT
CTTTGGAGTGTTGGAAGGGTTGATCAGTTAATTATTTGCTGGGTTAACTTTTTTTAAATTTTATTGT
TATTTTTTTTTTTTTCTTTTTTGGTTCCTTTTGGTATGTATTTGTTGTCTTTTTTTTGTGTCTTTTAAG
GATTTTTATGGTGCTAAATTAAGTTCTTATGAATGTGGTTTTGATGTTGTGAAGAAGGTTCATGTTG
GTTTTAATTTGGTTTTTTTTTCTATTGTTTTGTTGTTTGTTGTTTTTGAGTTGGAAGTTTTAATTTT
TATTATTTTGATTCAGGGTGATTTTTATAGTTTATTGTCTTTTTTTTTGTTTTTTTTTTTATGTTGTT
TTTAGTTTTTATATGGAGTGGTATTTTGGTAAGTTGATTTGGTTTTGTTAGTTGTTATTGTTTGTTT
GATATGTTCTTATTTTTTATTTTTTTTTGGTTGTTGTTTTTTTGTTTGTTCCTTATGGTAAATGGAG
TTATAGTTTTGGTTTTAGTGATTATTTTAATTTTACTTTTGTTTATAATTTTGAAGTTTGTTTGTTT
TTTTTAGTTTTGTTGTTGGTTTCTTTTATGGTTTTTGTTTATGGTTCTTTTTATATGGTTGGGGTTT

CTCGTTTGTTTTATTTTTTTTTTTTTTTTATTTTTGTTTGTGTTGAGGATGGGTGGCTTGATTGTTTT
TAGAGGTAGTATTGTTTTAACTTTGGTTTTTTGGGATTTTTTGGGGGTTAGTAGTTTTTTTTTGGTT
TTGTTTTATGGTAATGTTAGTGCTCGAAGGGGTGCTATGAGTACTGTGTTTACTAATCGTATTGGTG
ATTTTTGTATTTTTTTGTTTTTTAATGGTTTTGTTTTGTTTTCTATGAGTTTTTTGTCTTATCAGTT
TTTTGGTTCTTTGTTAGTTTTTATGTTGTTTGTTTCTTCTGTTATTAAGGGTGGTCAGTATCCTTTT
GGTAGCTGGTTGCCTAAGGCTATGGCTGCTCCTACTCCTGTTAGCTGTTTGGTTCATAGTAGTACTT
TAGTTACTGCTGGTGTTATGTTGATGGATTGTTATGTTTATATTTCTTTGAATTCTGATGTTTTGTC
TTTTGTTTTTTATGTTGGTTTTTTTACTATAGTTTTTTCTGGTTTTTGTGCTTTGGTGGAGGAGGAT
GCTAAGAAGATTGTTGCTTTGAGTACTATGTCTCAGATTGGTTTTTGTTTTTTGGCTATTGGTAGGG
GTTTGCATTATTTGTCTTATGTTCATATGATTAGGCATTCTTTTTTTAAGAGATTGTTGTTTATGCA
GATAGGTTATTTGATTTTTATTAATTTTGGTCAACAGGATTATCGTGGTTATTCTTTTTTTGGTTTT
TGTGCTCCGGTTTTAGTTCAGTTGCAGATTTTTTTATCTGTGTTTTGTTTGTGTGGTTTGTTGTTTA
CTAGAGGTAGTTGTAGTAAGGAATATTTTATATCTCGTTTTTATTATGATTCTTATGGTTTTTTTTT
AGTTTTTTTTTATTTTTTTGGTGTGTTTTTGACTTTTTGTTATTGTTATCGGATGTTTTTTTTTGTTT
CGTGTGGGGGCTTTTGGTTTTGATTATGTGGGTTTTTCTAGTAAGTTANTTTTATTTTTCTTGTTTT
TTTTTGGTTTTTTTTTCTGTTGTTTTTACTTTTTGGTGGGTTTTTGGTTTGTTATCTTTTTCTGCGG
CTTTTAATCGTTTTGAGTTTTTGGTTGTTTATTTTTATTTGTTTTTTGTTTATTGTTTTTGTGGTTA
TTTTTTTCGTTATTTTTTTTTCGTTATTTTGTGAAATTTTTTATGGATCATTATGCTTGTTTTATTT
ATAAAATTTTTCCTAGTTTTTTTTATTTTGATGTTTTTATTATGGGTTTTAATTATTTTTTTTTTGG
GTTATTTCGGTTATTTTCTTTTTTTTTTTTTTCTTGGTTTCGGGGGTTTTATCATGTTGGTGTTTTA
ATTGTTTTTTTTTTTATGTTGTTTTTTTTTGTTTTTTTAGATTTTGTTATTTTTTGTTTTTTTTTTAT
TATTGTTTTTGAGTTTTATTAATTTTTGTGTTGTTGATTATATTGTTTGGTGGAGGATTTTTGTTAT
TTGTACTTTTGTTTTTGTTTTTTTTGTTGGTGGTGAGTTAGGTTTTGGGGGTTATTTGGTTAATTAT
TATGTTATTCAGGAAGTTTGTGGTTATTATTTTTTGGTTTTTGATGGTTGGAAGTTGCAATTTTTAT
TGCTTATGTTGAAGTCTGGTTCTTCTCCTTTTCATTTTTGACTTTTTAGTGTTTTGGGTGGTTTGGA
TAAGTGGTTTATTTTGTGGTTTTTAACTTTGCAAAAATTGCCTTATTTTGTTGTTTTGGTTAATTTT
TGTGGTGATTTTTTTTTTTTTGTTTTTGTTTTTTGGTATAATTTTTTGTTATTTTCAATTTTTTTTGT
TGCGTAGTTATCGTGATTTGTTAGTTGTGGGTTCTGCTGAATCTTTTAATTGGTTATTGTTATTGGG
TATTTTTTCTTTTAATGAAGTATTTGTTTTGTTTTTTTTTTTATTATTTGTTATGTTTTTTGTTGTC
TCTTATGTGTATGGGGGATTTTTAAATTTTTTGAGTTTAGAGATATTGATGTTTTTTTTTAATGTTC
CTTTGAGAATTACTTTTTTTTTAAAGGTGATTGTGTTGTTTGGTTCTTCTTTTTTTGTTGGTTTTTA
TTATTTATTTTTGTTGTTGTTTATGCCTTTGATGTCTTTGGGTATGGGTTATTTGTTTTTTTTGGTT
TCGATGATGAGTTTTAATTATGGTTTTAAGTATTATGATTATTTTGTTTATGTTTTGTTTTGTGTTG
GGTTGTTGTCTTGTTTTTAGTTGTTGTATTTATTTTTTTTTGTTGTTTTGTTGTTTTTTTTTAGTCC
TTTTTTATTTTTTGTTTTTTTTATGTTTTTTGTTTTATATGGTTTTTTTGATTGTTCATGGTTTGGT
TGTTTGTTTTTTTTTGATTCTTTTAATTTTGTTTTTTTGTCTTTTATGAGTGTTTTTGTTATGGGGT
TTATTTGTGTGTCTGAATTGTTGAGTGGCTTAGTTTTTTATAGTTGTCTTGTAGTGTTTTTTAGTGT
TTGTTTTTTTTATTCTGGTAGTTTTTTGATATTGTATGTTTTTTATGAGTTGACTATAGTACCTATT
TTGTTTTGTTTGTTAGGGTATGGTCGTCAGGTGGAGAAGGTTAGGGCTTGTTATTATTTAATTTTTT
ATACTTTGTTTTTTGGGATGCCTTATTTGTTTTTGTATAGTCATGTTTTTTTTTTTTTGAATTTTGT
TTATTATGATTTTTTTGTTTCTTATGAATTTATTTTTTTGTTGAGTTTGTGTTTTTTAGTTAAGTTT
CCTGTTTATTTTTTTCATGTTTGATTACCTAAGGTTCATGTTGAGGCTCCTACTAGTGCTAGAATGA
TTTTGGCTGGTGTTATGTTGAAGTTGGGAGGAGCAGGTGTTTATCGTATTAGTAAGTCTTTGAATTT
TTTTGGTTTTGAAATGTTGATTTTTTTTTCTTTGATTAGGATGGTTTTTTGTTCTTTTATTTGTGTT
GTTCAGAGTGATTGTAAGTCTTTGGCGGCTTATTCTTCTGTTTGTCATATGGGTTTTGTATTGCTCT
CTGAGATTAGTATGGTTTATTATGGTAAGTCTATGGCTTTGGTGATGATGTTGGCTCATGGTTATAC
TTCTGTTTTAATGTTTTATTTTATTGGTGAGTTTTATCATATTGCTAATAGGCGTTTAATTTATTAT
TTGCGTGGATATTTTAATGTTAGTATGTTGTTTTGTTTGATGTTTTGTTTGACTATGGTTTCTAATT
TTAGTTTTCCTGTATCTATTTCTTTTTTTTTCTGAGTATTTGATGTTGAATTTTTTTAGGTCTGTTTT
TTATGTTGGTTTTTTGTTTTTGTTTTTTTATTATTTGGTTTCTTTTTATTATTCTGTTTATATTTTG
GTTTGTTTTTTAGTTGGGGATAAGGTGAGTTATGTTTGTGATGGTCGTAGTGTTGTTTGTTTACCTT
TGGTTTTTATGATATATAATTTTTTTTGGTTTATTTTTGTTATTTAATTTTAAGTTTGATTTTGGTT

TAGGTTGTATTAAGATAGTATTACTTATTTTTAGTTTATTTAATGTGTTATTTTTTGTACACTGGTA
GTTTTTTGATTGTTTTATTAGCGTTCCAGAATAATCGGCTATGCGTTTTAATTTTTGACTCTATTTG
TTGTGGTGCTATGAGTTTTTAGTTTGTTTTTATGTTGTTTTTTGTAAAATATTTTGATTTTTTTTAG
TTTCTTGTGGTATCAAAAATTTGTTTTTTGAACTGGATTAGTACCCAGGTAATCAAAATTTAATAAT
TCGGGAGTAAAGTTTTGTTTAAACCGAAAAAATATTGACTGACTTTAGATTTTTCTTTGGAACATGT
GATTTGCTGGAGAGCCCTCTTTTTTGGTGAATTTTGTTGGCACATGTATGATTGTTTAGTTTTTATT
TTATTTTGTAATGCTTTGTTGTTTTGGCATTAAAAACAGATATATATTTGGCTTATGAATTTATGTT
TCATGTGTTACTATTATGAATTTTTTTTGGATTAGTTTTTTATTTTTTTTTGAAATTGGAAAAGAAA
GTAATTTTTTTTTAATGTTTTAATGAATTTAATAAATAAGGTGGTACAAACCATCCGTCAATGGCCT
AAAGGGGCGTAAGTTGTAGTATGGTAGAAGTAAGGAAACTTGTTTCTATTTTTTGAAGTTTTTTTGT
TTTTTTAAGTTTTATTATTTGGTATTGCATATCAGTAGAAGTTTTTATCATAGTTATGAGTAATAGA
ATTAAAATGGTTAAATTGAATTTGTTTTTTTTTTACGAAATTAATAATATTTTTATTTTAGTATTGA
TATAACGTATTTTTATTTCTGTTTATTTGTTATTTATAGGTTAAGTTATACTGTTTTTGTTTGTAGA
TTTTTAAATTTTTGTTGCTGTTTTATAAATGTTGTGTGTTTTACATAGATTTTTATTTTTTTTTTTT
TTTTTTTTTTGATTAATTTTTTAATTAATTTATGATTTTTACTTTAGTAATTTTTATTATTTTGAGG
GGTGTTTGTTTTTGAATTTATGTTTTTGAACTGGTTTTGTTGCTAAATGTTTATTAAAAACTTAGGT
TTTTATGTAAAATTGTCTTCTGCTCTATGAGTTTTTAAATGGCAGCCTTAGCGTGATGGCGTAAAAG
TAGCGTAAGTGATTTGTTTTTTTAATGGTTTCAAGTATGAATGAAGTTTTTAGCAGTTTTTTTATTT
ACTTTTTATTTGAATTATTTTTTTGATTAAAAATTATTAGTTAAGGTATTACAAAGATAAGTCTTCG
GAAATTTTGTTTTGAATTTTGAAATTTTTGTTTTTAATTTTTTCTTGGGGATGGATTTTAAGAAAGT
TTTATACTATTGTTATTATTAAAAATTACTCCGGAGTTAACAGGGTTGTAGACATATAAATAGATTT
TTATATTAGTGTGCTGCGCTACATCGATGTTGTATATTTTTTTGATAATGGAGAGGTTTTTTTTAT
TTTGAGACTGTTCTTCTTGTATAAAAAATTGACTTGATATTAGTTTAGTTCGTCGTGAGACAGAGCG
GTTTATCTTGTGTATTTTTGGTTTTTGGCGGTGTTAGTACGAAAGGAATGCAATGTGGGTTTATATT
TATGACTTTTTTATTTTGATGGGTTTTT

>O.ochengiF2
ATTTTTTGTGGAATGACTTTTGGTAATGGTATGAAGCAGAGTATTATTAATACTGTGAATCATAAGA
CTATTGGTACTTATTATATTGTTTTAGGTTATTGGGCTGGTTTAGGTGGTTCTGTTTTATCTATGTT
GATTCGTTTTGAATTGTCTAGTCCTGGTGGTTATTTGTTTTTTGGAAGTGGTCAGGTTTATAATTCT
GTTCTTACTATGCATGGTGTTTTGATGATTTTTTTTTTTGGTTATGCCTATTTTGATTGGTGGCTTTG
GTAATTGGATGTTGCCTTTAATATTAGGGGCTCCTGAGATGGCGTTTCCTCGGGTAAATGCTTTATC
TTTTTGATTTACTTTTGTGGCTTTGTTGATAGTTTATCAGTCTTTTTTTATTGGGGGTGGCCCTGGT
AGAAGTTGGACTTTTTATCCTCCTCTTAGGGTTGAAGGTCAACCAGAATTGTCTTTAGATACTATGA
TTTTAGGTTTACATACTGTAGGAATTGGTTCTTTGTTGGGTGCTATTAATTTTATGGTAACTACTCA
GAATATACGGTCTACTGCTGTGACTTTGGATCAAATTAGTATGTTTGTTTGGACTTCTTATTTGACT
TCTTTTTTGTTAGTTTTGTCTGTGCCTGTTTTGGCTGGTTCTTTATTGTTTTTGTTGTTGGATCGTA
ATTTTAATACTTCTTTTTATGATACTAAGAAGGGGGGTAATCCTTTGTTGTATCAGCATTTGTTTTG
ATTTTTTGGTCATCCTGAGGTGTATGTTATTATTTTACCTGTTTTTGGTATTATTAGGGAAGCGGTT
TTATTTTTGACTGATAAGGATCGTTTGTTTGGTCAGACTAGGATAACTTTTGCTTCTATTTGGATTG
CTGTTTTAGGTACTTCTGTGTGAGGTCATCATATGTATACGGCTGGTTTGGATATTGATACTCGTAC
TTATTTTAGTGCTGCTACTATGATTATCGCTATTCCTAGAGCTGTTAAGATTTTTAATTGGTTAGGT
ACTTTTTTTGGTTCTAGTCAAAAGGTGCAGCCGTTATGATGTTGAACTTATAGTTTTATTTTTCTTT
TTACTGTGGGTGGATTAAGTGGAATTATTCTGAGGGCTGCTAGTTTGGATATTATTTTGCACGATAC
TTATTATGTTGTGGCTCATTTTCATTATACTTTGAGTTTGGGTGCTATTTATGGTATTTTTTGTGGT
TTTTGTTTGTGACTTCCTTATATGTATGGTATTTCTTTTGATAGGGTTATGATAATAGCTGTTTTTG
TTTGTTTTTTTGTTGGTACTAATATGACATTTTTTCCTATGCATTTTGCTGGTTTGCAGGGTATGCC
TCGTAAGATTTTGGATTATCCTGATTGTTATTCTACTTTTCAGATTATTTCTTCTTTAGGTTCTGTT
ATTACTTTTGTTGGTTTTGTTTTGTTTAATTATTTGTTGGTTGATTCTATTTTTTTTTTCTCGTTTTT
TGGGGGTTTCTTTTTATAATTATCATAGTCCGGCTTATGCTTTAAATGTTCCTCCTTTGCCGGATTC
TTTTACTGAAGAGGCTTTTATTATAGGTCTTCATTGGAAGATTATTAGTAAGGATACTCCTTCTTAT
AGGTATCGTCGGGTTGGTTATGGTTATCATAGTAAGTAAATTTTTTTTTTATGTTAGGGTATTTTTTG

CTTTTGTTTTTTTTTGTTTGAGTTTTTTGGATTGGGATCCGTTGAAGAGTTGTGTTATGATGTGTTT
GGGTATTATGTCTATAAGTTGTTATGTTTCTTTGGGTATTCATGTATGATATTCTTATTTTGTTGTT
TTAATTTTTTTTAGTGGTATTTTTTCTTTGTTGACTTATTTTTGTAGTATGAGTAATTTTGTTTTTT
ATTATAATTATTTTTTTTTTTTTTNTTTGTTTTTGGTTAGTTTTTTTTTTGTATTTGTTGTAGATTT
TGATTTTTTTTTGTTTTTTTGTGATTTTAATTTTCTTTATGTTTGTTATGATTTTAGTTATTATTAT
GTTTTTTGAGTAGTTTTTGTTTTGTTTTTGTTTTTGGTTTTGGTTAGATTTAGGTTTAATGGTTTTG
GTTATATGCGTAGCTTGTAGATTGTTATTTTTAATTCTTTAGTTTTTTTGCCTGCTAGTTTTACTTT
GAGTTATATGTGAAATTTTGGTAGTATGTTGGGTATTATGTTGATGTCTCAGATTTTAACTGGTTTT
TTTTTGACTTTTTACTATACGGCTGGGGAAGCTTTTAGGTCTGTTCAGTATATTATGTTTGAGGTTA
ATTTGGGTTGGTTGTTGCGTATTATGCATTCTAATGGGGCTTCTATGTTTTTTTTGTTTATTTATTT
ACATATTTTTAAGGGTCTGATTTATGGTAGATATCGTCTTATTGGTGTATGGTTGAGTGGTATTTTT
ATTTATTTTTTATTGATAGGTATTGCTTTTACTGGTTATGTTTTGATTTGAGGTCAAATAAGTTATT
GGGCGGCAGTAGTTATTACTAGTTTAATAACTTCTGTTCCTTATTTAGGTAAGTATTTAGTTTGATG
GATTTGGGGGAGTTTTAGTGTTTGTGAGAACACTTTAAAGTTTTTTTATTCTGTTCATTTTATTTTA
CCTTGGTCTTTGATAGTTTTAGTTGTTTTTCATTTGTTTTTTTTGCATTTTACTGGTTCTAGTTCTA
GTTTGTATTGTCACGGGGATTATGATAAGATTCATTTTTTTCCTAGTTTTTGATTGAAGGATGGTTT
TGATATTTTTTTTTATTTTTTTTTGATTTTGTTTAGACTTTATTTTTCTTTTGATTTAAGTGATCCT
ATGATTTTTGTGGAGTCTGATTCTATGGCTAGTCCTGCGCATGTTGTACCTGAGTGATATTTTTTAT
TTGCTTTTACTATCTTACGTTCTGTTCCTAGTAAGTTATTAGGGGTTATTTTAATATTTAGTTCTGT
TTTTGTATTGACTATTCTTGTTTGACCTGATAGTTATCAGTCTATTTTGGATAATTTTTTATATTTT
TTTGTTATGTGTTTTGTTTGGATTTTTTTTTGGTTAACTTGAGCTGGTCATTATCCTACTGATTATC
CTTTTAACTATTTTAATTTGTTTTGTACTTTTTTTTATTTTTGTTGTATTTTTTTTGTTTGTTTAAT
TAATTTTTTTTAGTGATAAGTTGTTTAGTTAAATTTTGTTGAAGTTTCGTAAGTACCATAAAATGGAG
TATAGTTATTATCCTTTGATAGTTGGGGCGGGTTTTTTAGGTTTTGATGTTAGTTTGGTTTTATTTA
TAAGTATAGGTATGTTTTATTCTATTTTTATTTGTTTTTTGTATTTGGTTTATGTTTTTTTTTTGTG
AATCAAGGATGTTATTTTAGAGGACATTAGTGGTCAATATTCTTTTTATGACTATCGTATGTTTAAT
CAAGGTTTTCGTTTGTTTCTTTTTAGTGAGTTAACTTTGTTTGTTTCTATTTTTTGAACTTTTTTGG
ATACAGCTTTGTGTCCTTTAACTTGGTTAGGTGGGGTTTGGTCTCCATTTGGGATTTTATCTCCTGA
TTATTTGGGTTTGAATGGTATGGCTAGTTTGTTTTTAATGATAAATAGGCAAGTTTTGAAGTATTCT
CGTCGTTATTTGTGTTTGAGTAGTTCTAAGTGTGAAGAGTTTTTGTTAGTTTGTATTTTTGTTGGAG
TTGGTTTTTTATGTTTTCAGTTTTATGAATATAATAACAATTCGTTTGTTATGAGTGATAGTGTTTA
TGGTAGTATTTTTTATATGGGTACTGGTTTACATGGTTTGCATGTTTTTATTGGTGTTTGTTTTCTT
ATTGTTAATTTTTTTCGTGTTAAGTTGTTCAATTTTAATTGATATCATGTTCAGGCTTATGATATAT
CTATTGATTATTGGCGTTTTTTAGAATGAATGTGAGGTGTTATGTTTTGTTTATTATATGTTTGAGG
TTCTTAATTGGTTATTTTTTATTATTTGGGGTTGTTGGTTATGATTGTTTTTATTTTGCAGGCTATT
GCTTTTTTAACTTTGTTGGAGCGCCATTTTTTGGGTGGTTCTCAGTGTCGTGTTGGTCCTAATAAGG
TGGGTTATTCTGGTGTTTTGCAGGCTTTATTTGATGGTTTAAAGTTGTTAAAGAAGGAGCAGTTGTT
GTTGTGTTTTTCTTCTTGATTATCTTTTTTGTTTATGCCTGTTTGTGGTTTTGTTTTGATGGTTTTT
TTTTGATTTACTTTGCCTTATTTTTTTTTCTTTTTTGTCTTTTGAGTACTCTGGTGTTTTTTTGTTTT
GTCTTATAGGGGTTTCTGTTTATTTTATTATGCTTTCTGGTGTTTTTAGCGGTAGTAAATATTCTTT
TGTTGGTGGATTGCGTGCTTGCGTTCAGAGTTATTCTTATGAGATTGCTTTTTCTATTTATTTGTTG
GTTTTTTTGTTGTTTAATAAGGGTCTATGTTTGTCTTTTAGTTTTTGTTTATTTTTTTTTTTGTTTT
TTTTTCCTTTTTTTTGTTTAGTTCTTGTTGATTTGCATCGGGCTCCTTTTGATTTTTCTGAGTGTGA
AAGTGAGTTGGTAAGGGGGTTTAATGTTGAGTATTCGGGAGTTGGTTTTGCTGCTTTGTTTTTAGGG
GAGTATGGTAATTTACTTTATTTTGGTTGTTTGACTTCTAGTTTGTTTTTTGGTATAAGTTTTTTTT
TTTTTTATTTTATTGTATGTATGATTGTTTTTTCTCGTAGAGCTTATCCTCGTTTTCGTTTTGATAA
GTTGATGGGTGTTTGTTGGTTTTTGTTTTTGCCTGTCGGTTTTTATTTTTTTGGTTTGTCTTTTGTT
GTTTTTATGTTGTGCTTATTTAGTTTAATTTTTTAGATTTTGTTATTTTTTTTTTATTTGGTTTTGAT
TAGTTTTTTATTTGTTTTATATGGAGTTGAGTAAGTTTAGTAGTTTGGGAGTTTTAGGTGTTTTTGT
TAATGTTTTAGTTTCTGGATTTTCTCATCAGGGTTTTCAGTCTAGTGTTTTTTTTAAGTTTGTTGTT
TTTTTTCTTTTGGTTTTTTGAATGAGTGGGTTATTGTTTCCTTTGTTTAGTCCTTGGGCTTGTGTGG
GTTTTTTGTTTTTTGTTACTAATTTTTCTTGATTGGGTGTTCGTACTTTTATTTTAGCTGTAGATAG

TTTTTTGATTTTTTTTGAGGGGGATCACTCTTGGGAGTGGTTTTCTAGGTTAGTTATGTTTTTTTCT
CATTGGTTGAGATTTTTGATAAGGGGGGTGGCTTTAACTTTGCGTATTAGTATTATTTTTTTAATTG
GTCATTTTTTAATGTTTACTGTTTTGGATATGAGTGTATTTTATTCTTTGTTTTTTTTGTTGTTTGT
GGTTCCGGTGGAGTTGTTTTTTGCTTTTTTACAGAGTTATATTTTTTTGACTTTGGTTTGTATGTTT
TTACTTAATATGATTTAGATTTATTTGCAGAATTATGTGTTTCCTATTCCTGGAAATTCTTATGTGT
TTTGTTGTTATTATATCCATAATTATTATTCTCATATTATTTTTTTTGGTTTTTTTGTGATGTTTTT
GGTTAGTGGTGGGGTTTATTTTTTTTGGCAATTCTTTTAAGTTTAACTTGAAGCGGAGAGATAGTCGT
ATAATTGAATTAGTTTTACAGGTGTTGATTGTTAATTTTTTGATTATGATGGCAGGTCCTGGTTTTT
GGTTGATTCAGTATCAGGGACGTATGTTTCGTCAATCTGAGTTGGCTTTGAAGGTTATTGGTCATCA
ATGGTATTGGAGTTATGAGTATGGTGATAGTGGAAAATTATGTTTTGATTCATTTATGAAGTCTTTA
GATGATTTGTCTTTAGGGGATTTTCGGTTATTTGATGTTGATAATCGGTGTGTTTTGCCTGTAGGTG
TGAATGTTGGAGTGTATTGTACTTCTAGTGATGTTATTCATTCTTTTGCTATTCCTAAGTGTTTTAT
TAAGATGGATGCTTTGAATGGTTTGTTAACTAAGGTTACTTGTAATTTTTCTTGTTCTGGTTTGTTT
TTTGGGCAGTGTTCTGAAATTTGTGGTGCTAATCATAGGTTTATGCCTATTGTGTTGGAGTTGACTT
CTTTGGAGTGTTGGAAGGGTTGATCAGTTAATTATTTGCTGGGTTAACTTTTTTTAAATTTTATTGT
TATTTTTTTTTTTTCTTTTTTGGTTCCTTTTGGTATGTATTTGTTGTCTTTTTTTGTGTCTTTTAAG
GATTTTTATGGTGCTAAATTAAGTTCTTATGAATGTGGTTTTGATGTTGTGAAGAAGGTTCATGTTG
GTTTTAATTTGGTTTTTTTTTCTATTGTTTTGTTGTTTGTTGTTTTTGAGTTGGAAGTTTTAATTTT
TATTATTTTGATTCAGGGTGATTTTTATAGTTTATTGTCTTTTTTTTGTTTTTTTTTTTATGTTGTT
TTTAGTTTTTATATGGAGTGGTATTTTGGTAAGTTGATTTGGTTTTGTTAGTTGTTATTGTTTGTTT
GATATGTTCTTATTTTTTATTTTTTTTTGGTTGTTGTTTTTTTGTTTGTTCCTTATGGTAAATGGAG
TTATAGTTTTGGTTTTAGTGATTATTTTAATTTTACTTTTGTTTATAATTTTGAAGTTTGTTTGTTT
TTTTTAGTTTTGTTGTTGGTTTCTTTTATGGTTTTTGTTTATGGTTCTTTTTATATGGTTGGGGTTT
CTCGTTTGTTTTATTTTTTTTTTTTTTTTATTTTTGTTTGTGTTGAGGATGGGTGGCTTGATTGTTTT
TAGAGGTAGTATTGTTTTAACTTTGGTTTTTTGGGATTTTTTGGGGGTTAGTAGTTTTTTTTTGGTT
TTGTTTTATGGTAATGTTAGTGCTCGAAGGGGTGCTATGAGTACTGTGTTTACTAATCGTATTGGTG
ATTTTTGTATTTTTTTGTTTTTTAATGGTTTTGTTTTGTTTTCTATGAGTTTTTTGTCTTATCAGTT
TTTTGGTTCTTTGTTAGTTTTTATGTTGTTTGTTTCTTCTGTTATTAAGGGTGGTCAGTATCCTTTT
GGTAGCTGGTTGCCTAAGGCTATGGCTGCTCCTACTCCTGTTAGCTGTTTGGTTCATAGTAGTACTT
TAGTTACTGCTGGTGTTATGTTGATGGATTGTTATGTTTATATTTCTTTGAATTCTGATGTTTTGTC
TTTTGTTTTTTTATGTTGGTTTTTTTACTATAGTTTTTTCTGGTTTTTGTGCTTTGGTGGAGGAGGAT
GCTAAGAAGATTGTTGCTTTGAGTACTATGTCTCAGATTGGTTTTTGTTTTTTGGCTATTGGTAGGG
GTTTGCATTATTTGTCTTATGTTCATATGATTAGGCATTCTTTTTTTAAGAGATTGTTGTTTATGCA
GATAGGTTATTTGATTTTTATTAATTTTGGTCAACAGGATTATCGTGGTTATTCTTTTTTTGGTTTT
TGTGCTCCGGTTTTAGTTCAGTTGCAGATTTTTTTATCTGTGTTTTGTTTGTGTGGTTTGTTGTTTA
CTAGAGGTAGTTGTAGTAAGGAATATTTTATATCTCGTTTTTATTATGATTCTTATGGTTTTTTTTT
AGTTTTTTTTTATTTTTTTGGTGTGTTTTTGACTTTTTGTTATTGTTATCGGATGTTTTTTTTGTTT
CGTGTGGGGGCTTTTGGTTTTGATTATGTGGGTTTTTCTAGTAAGTTANTTTTATTTTTCTTGTTTT
TTTTTGGTTTTTTTTTCTGTTGTTTTTACTTTTTGGTGGGTTTTTGGTTTGTTATCTTTTTCTGCGG
CTTTTAATCGTTTTGAGTTTTTGGTTGTTTATTTTTATTTGTTTTTTGTTTATTGTTTTTGTGGTTA
TTTTTTTCGTTATTTTTTTTTCGTTATTTTGTGAAATTTTTTATGGATCATTATGCTTGTTTTATTT
ATAAAATTTTTCCTAGTTTTTTTTATTTTGATGTTTTTATTATGGGTTTAATTATTTTTTTTTTTGG
GTTATTTCGGTTATTTTCTTTTTTTTTTTTTTCTTGGTTTCGGGGGTTTTATCATGTTGGTGTTTTA
ATTGTTTTTTTTTTTATGTTGTTTTTTTTGTTTTTTTAGATTTGTTATTTTTTGTTTTTTTTTTAT
TATTGTTTTTGAGTTTTATTAATTTTTGTGTTGTTGACTATATTGTTTGGTGGAGGATTTTTGTTAT
TTGTACTTTTGTTTTTGTTTTTTTTGTTGGTGGTGAGTTAGGTTTTGGGGGTTATTTGGTTAATTAT
TATGTTATTCAGGAAGTTTGTGGTTATTATTTTTTGGTTTTTGATGGTTGGAAGTTGCAATTTTTAT
TGCTTATGTTGAAGTCTGGTTCTTCTCCTTTTCATTTTTGACTTTTTAGTGTTTTGGGTGGTTTGGA
TAAGTGGTTTATTTTGTGGTTTTTAACTTTGCAAAAATTGCCTTATTTTGTTGTTTTGGTTAATTTT
TGTGGTGATTTTTTTTTTTTTGTTTTTGTTTTTTGGTATAATTTTTTGTTATTTTCAATTTTTTTTGT
TGCGTAGTTATCGTGATTTGTTAGTTGTGGGTTCTGCTGAATCTTTTAATTGGTTATTGTTATTGGG
TATTTTTTCTTTTAATGAAGTATTTGTTTTGTTTTTTTTTTATTATTTGTTATGTTTTTTGTTGTC

TCTTATGTGTATGGGGGATTTTTAAATTTTTTGAGTTTAGAGATATTGATGTTTTTTTTTTAATGTTC
CTTTGAGAATTACTTTTTTTTTTAAAGGTGATTGTGTTGTTTGGTTCTTCTTTTTTTGTTGGTTTTTA
TTATTTATTTTTGTTGTTGTTTATGCCTTTGATGTCTTTGGGTATGGGTTATTTGTTTTTTTTTGGTT
TCGATGATGAGTTTTAATTATGGTTTTAAGTATTATGATTATTTTGTTTATGTTTTGTTTTGTGTTG
GGTTGTTGTCTTGTTTTTAGTTGTTGTATTTATTTTTTTTTTGTTGTTTTGTTGTTTTTTTTTTAGTCC
TTTTTTATTTTTTGTTTTTTTTATGTTTTTTGTTTTATATGGTTTTTTTGATTGTTCATGGTTTGGT
TGTTTGTTTTTTTTTGATTCTTTTAATTTTGTTTTTTTGTCTTTTATGAGTGTTTTTGTTATGGGGT
TTATTTGTGTGTCTGAATTGTTGAGTGGTTTAGTTTTTTATAGTTGTCTTGTAGTGTTTTTTAGTGT
TTGTTTTTTTTATTCTGGTAGTTTTTTGATATTGTATGTTTTTTATGAGTTGACTATAGTACCTATT
TTGTTTTGTTTGTTAGGGTATGGTCGTCAGGTGGAGAAGGTTAGGGCTTGTTATTATTTAATTTTTT
ATACTTTGTTTTTTGGGATGCCTTATTTGTTTTTGTATAGTCATGTTTTTTTTTTTTTGAATTTTGT
TTATTATGATTTTTTTGTTTCTTATGAATTTATTTTTTTGTTGAGTTTGTGTTTTTTAGTTAAGTTT
CCTGTTTATTTTTTTCATGTTTGATTACCTAAGGTTCATGTTGAGGCTCCTACTAGTGCTAGAATGA
TTTTGGCTGGTGTTATGTTGAAGTTGGGAGGAGCAGGTGTTTATCGTATTAGTAAGTCTTTGAATTT
TTTTGGTTTTGAAATGTTGATTTTTTTTTCTTTGATTAGGATGGTTTTTTGTTCTTTTATTTGTGTT
GTTCAGAGTGATTGTAAGTCTTTGGCGGCTTATTCTTCTGTTTGTCATATGGGTTTTGTATTGCTCT
CTGAGATTAGTATGGTTTATTATGGTAAGTCTATGGCTTTGGTGATGATGTTGGCTCATGGTTATAC
TTCTGTTTTAATGTTTTATTTTATTGGTGAGTTTTATCATATTGCTAATAGGCGTTTAATTTATTAT
TTGCGTGGATATTTTAATGTTAGTATGTTGTTTTGTTTGATGTTTTGTTTGACTATGGTTTCTAATT
TTAGTTTTCCTGTATCTATTTCTTTTTTTTCTGAGTATTTGATGTTGAATTTTTTTAGGTCTGTTTT
TTATGTTGGTTTTTTGTTTTTGTTTTTTTATTATTTGGTTTCTTTTTATTATTCTGTTTATATTTTG
GTTTGTTTTTTAGTTGGGGATAAGGTGAGTTATGTTTGTGATGGTCGTAGTGTTGTTTGTTTACCTT
TGGTTTTTATGATATATAATTTTTTTTGGTTTATTTTTGTTATTTAATTTTAAGTTTGATTTTGGTT
TAGGTTGTATTAAGATAGTATTACTTATTTTTAGTTTATTTAATGTGTTATTTTTTGTACACTGGTA
GTTTTTTGATTGTTTTATTAACGTTCCAGAATAATCGGCTATGCGTTTTAATTTTTGACTCTATTTG
TTGTGGTGCTATGAGTTTTTAGTTTGTTTTTATGTTGTTTTTTGTAAAATATTTTGATTTTTTTTAG
TTTCTTGTGGTATCAAAAATTTGTTTTTTGAACTGGATTAGTACCCAGGTAATCAAAATTTAATAAT
TCGGGAGTAAAGTTTTGTTTAAACCGAAAAAAATATTGACTGACTTTAGATTTTTCTTTGGAACATGT
GATTTGCTGGAGAGCCCTCTTTTTTTGGTGAATTTTGTTGGCACATGTATGATTGTTTAGTTTTTATT
TTATTTTGTAATGCTTTGTTGTTTTGGCATTAAAAACAGATATATATTTGGCTTATGAATTTATGTT
TCATGTGTTACTATTATGAATTTTTTTTGGATTAGTTTTTTATTTTTTTTTTGAAATTGGAAAAGAAA
GTAATTTTTTTTTAATGTTTTAATGAATTTAATAAATAAGGTGGTACAAACCATCCGTCAATGGCCT
AAAGGGGCGTAAGTTGTAGTATGGTAGAAGTAAGGAAACTTGTTTCTATTTTTTGAAGTTTTTTTGT
TTTTTTAAGTTTTATTATTTGGTATTGCATATCAGTAGAAGTTTTTATCATAGTTATGAGTAATAGA
ATTAAAATGGTTAAATTGAATTTGTTTTTTTTTTACGAAATTAATAATATTTTTATTTTAGTATTGA
TATAACGTATTTTTATTTCTGTTTATTTGTTATTTATAGGTTAAGTTATACTGTTTTTGTTTGTAGA
TTTTTAAATTTTTGTTGCTGTTTTATAAATGTTGTGTGTTTTACATAGATTTTTATTTTTTTTTTTT
TTTTTTTTTTGATTAATTTTTTAATTAATTTATGATTTTTACTTTAGTAATTTTTATTATTTTGAGG
GGTGTTTGTTTTTGAATTTATGTTTTTGAACTGGTTTTGTTGCTAAATGTTTATTAAAAACTTAGGT
TTTTATGTAAAATTGTCTTCTGCTCTATGAGTTTTTAAATGGCAGCCTTAGCGTGATGGCGTAAAAG
TAGCGTAAGTGATTTGTTTTTTTAATGGTTTCAAGTATGAATGAAGTTTTTAGCAGTTTTTTTATTT
ACTTTTTATTTGAATTATTTTTTTGATTAAAAATTATTAGTTAAGGTATTACAAAGATAAGTCTTCG
GAAATTTTGTTTTGAATTTTGAAATTTTTGTTTTTAATTTTTTCTTGGGGATGGATTTTAAGAAAGT
TTTATACTATTGTTATTATTAAAAATTACTCCGGAGTTAACAGGGTTGTAGACATATAAATAGATTT
TTATATTAGTGTGCTGCGCTACATCGATGTTGTATATTTTTTTTGATAATGGAGAGGTTTTTTTTAT
TTTGAGACTGTTCTTCTTGTATAAAAAATTGACTTGATATTAGTTTAGTTCGTCGTGAGACAGAGCG
GTTTATCTTGTGTATTTTTTGGTTTTTGGCGGTGTTAGTACGAAAGGAATGCAATGTGGGTTTATATT
TATGACTTTTTTATTTTGATGGGTTTTT

>O.ochengiF3
ATTTTTTGTGGAATGACTTTTGGTAATGGTATGAAGCAGAGTATTATTAATACTGTGAATCATAAGA
CTATTGGTACTTATTATATTGTTTTAGGTTATTGGGCTGGTTTAGGTGGTTCTGTTTTATCTATGTT

GATTCGTTTTGAATTGTCTAGTCCTGGTGGTTATTTGTTTTTTGGAAGTGGTCAGGTTTATAATTCT
GTTCTTACTATGCATGGTGTTTTGATGATTTTTTTTTTGGTTATGCCTATTTTGATTGGTGGCTTTG
GTAATTGGATGTTGCCTTTAATATTAGGGGCTCCTGAGATGGCGTTTCCTCGGGTAAATGCTTTATC
TTTTTGATTTACTTTTGTGGCTTTGTTGATAGTTTATCAGTCTTTTTTTATTGGGGGTGGCCCTGGT
AGAAGTTGGACTTTTTATCCTCCTCTTAGGGTTGAAGGCCAACCAGAATTGTCTTTAGATACTATGA
TTTTAGGTTTACATACTGTAGGAATTGGTTCTTTGTTGGGTGCTATTAATTTTATGGTAACTACTCA
GAATATACGGTCTACTGCTGTGACTTTGGATCAAATTAGTATGTTTGTTTGGACTTCTTATTTGACT
TCTTTTTTGTTAGTTTTGTCTGTGCCTGTTTTGGCTGGTTCTTTATTGTTTTTGTTGTTGGATCGTA
ATTTTAATACTTCTTTTTATGATACTAAGAAGGGGGGTAATCCTTTGTTGTATCAGCATTTGTTTTG
ATTTTTTGGTCATCCTGAGGTGTATGTTATTATTTTACCTGTTTTTGGTATTATTAGGGAAGCGGTT
TTATTTTTGACTGATAAGGATCGTTTGTTTGGTCAGACTAGGATAACTTTTGCTTCTATTTGGATTG
CTGTTTTAGGTACTTCTGTGTGAGGTCATCATATGTATACGGCTGGTTTGGATATTGATACTCGTAC
TTATTTTAGTGCTGCTACTATGATTATCGCTATTCCTAGAGCTGTTAAGATTTTTAATTGGTTAGGT
ACTTTTTTTGGTTCTAGTCAAAAGGTGCAGCCGTTATGATGTTGAACTTATAGTTTTATTTTTCTTT
TTACTGTGGGTGGATTAAGTGGAATTATTCTGAGGGCTGCTAGTTTGGATATTATTTTGCACGATAC
TTATTATGTTGTGGCTCATTTTCATTATACTTTGAGTTTGGGTGCTATTTATGGTATTTTTTGTGGT
TTTTGTTTGTGACTTCCTTATATGTATGGTATTTCTTTTGATAGGGTTATGATAATAGCTGTTTTTG
TTTGTTTTTTTGTTGGTACTAATATGACATTTTTTCCTATGCATTTTGCTGGTTTGCAGGGTATGCC
TCGTAAGATTTTGGATTATCCTGATTGTTATTCTACTTTTCAGATTATTTCTTCTTTAGGTTCTGTT
ATTACTTTTGTTGGTTTTGTTTTGTTTAATTATTTGTTGGTTGATTCTATTTTTTTTTCTCGTTTTT
TGGGGGTTTCTTTTTATAATTATCATAGTCCGGCTTATGCTTTAAATGTTCCTCCTTTGCCGGATTC
TTTTACTGAAGAGGCTTTTATTATAGGTCTTCATTGGAAGATTATTAGTAAGGATACTCCTTCTTAT
AGGTATCGTCGGGTTGGTTATGGTTATCATAGTAAGTAAATTTTTTTTTATGTTAGGGTATTTTTTG
CTTTTGTTTTTTTTTGTTTGAGTTTTTTGGATTGGGATCCGTTGAAGAGTTGTGTTATGATGTGTTT
GGGTATTATGTCTATAAGTTGTTATGTTTCTTTGGGTATTCATGTATGATATTCTTATTTTGTTGTT
TTAATTTTTTTTAGTGGTATTTTTTCTTTGTTGACTTATTTTTGTAGTATGAGTAATTTTGTTTTTT
ATTATAATTATTTTTTTTTTTTTTNTTTGTTTTTGGTTAGTTTTTTTTTTGTATTTGTTGTAGATTT
TGATTTTTTTTTGTTTTTTTGTGATTTTAATTTTCTTTATGTTTGTTATGATTTTAGTTATTATTAT
GTTTTTTGAGTAGTTTTTGTTTTGTTTTTGTTTTTGGTTTTGGTTAGATTTAGGTTTAATGGTTTTG
GTTATATGCGTAGCTTGTAGATTGTTATTTTTAATTCTTTAGTTTTTTTGCCTGCTAGTTTTACTTT
GAGTTATATGTGAAATTTTGGTAGTATGTTGGGTATTATGTTGATGTCTCAGATTTTAACTGGTTTT
TTTTTGACTTTTTACTATACGGCTGGGGAAGCTTTTAGGTCTGTTCAGTATATTATGTTTGAGGTTA
ATTTGGGTTGGTTGTTGCGTATTATGCATTCTAATGGGGCTTCTATGTTTTTTTTGTTTATTTATTT
ACATATTTTTAAGGGTCTGATTTATGGTAGATATCGTCTTATTGGTGTATGGTTGAGTGGTATTTTT
ATTTATTTTTTATTGATAGGTATTGCTTTTACTGGTTATGTTTTGATTTGAGGTCAAATAAGTTATT
GGGCGGCAGTAGTTATTACTAGTTTAATAACTTCTGTTCCTTATTTAGGTAAGTATTTAGTTTGATG
GATTTGGGGGAGTTTTAGTGTTTGTGAGAACACTTTAAAGTTTTTTTATTCTGTTCATTTTATTTTA
CCTTGGTCTTTGATAGTTTTAGTTGTTTTTCATTTGTTTTTTTTGCATTTTACTGGTTCTAGTTCTA
GTTTGTATTGTCACGGGGATTATGATAAGATTCATTTTTTTCCTAGTTTTTGATTGAAGGATGGTTT
TGATATTTTTTTTTATTTTTTTTTGATTTTGTTTAGACTTTATTTTTCTTTTGATTTAAGTGATCCT
ATGATTTTTGTGGAGTCTGATTCTATGGCTAGTCCTGCGCATGTTGTACCTGAGTGATATTTTTTAT
TTGCTTTTACTATCTTACGTTCTGTTCCTAGTAAGTTATTAGGGGTTATTTTAATATTTAGTTCTGT
TTTTGTATTGACTATTCTTGTTTGACCTGATAGTTATCAGTCTATTTTGGATAATTTTTTATATTTT
TTTGTTATGTGTTTTGTTTGGATTTTTTTTTGGTTAACTTGAGCTGGTCATTATCCTACTGATTATC
CTTTTAACTATTTTAATTTGTTTTGTACTTTTTTTTTATTTTTGTTGTATTTTTTTTTGTTTGTTTAAT
TAATTTTTTTAGTGATAAGTTGTTTAGTTAAATTTTGTTGAAGTTTCGTAAGTACCATAAAATGGAG
TATAGTTATTATCCTTTGATAGTTGGGGCGGGTATTTTAGGTTTTGATGTTAGTTTGGTTTTATTTA
TAAGTATAGGTATGTTTTATTCTATTTTTATTTGTTTTTTGTATTTGGTTTATGTTTTTTTTTTGTG
AATCAAGGATGTTATTTTAGAGGACATTAGTGGTCAATATTCTTTTTATGACTATCGTATGTTTAAT
CAAGGTTTTCGTTTGTTTCTTTTTAGTGAGTTAACTTTGTTTGTTTCTATTTTTTGAACTTTTTTGG
ATACAGCTTTGTGTCCTTTAACTTGGTTAGGTGGGGTTTGGTCTCCATTTGGGATTTTATCTCCTGA
TTATTTGGGTTTGAATGGTATGGCTAGTTTGTTTTTAATGATAAATAGGCAAGTTTTGAAGTATTCT

CGTCGTTATTTGTGTTTGAGTAGTTCTAAGTGTGAAGAGTTTTTGTTAGTTTGTATTTTTGTTGGAG
TTGGTTTTTTATGTTTTCAGTTTTATGAATATAATAACAATTCGTTTGTTATGAGTGATAGTGTTTA
TGGTAGTATTTTTTATATGGGTACTGGTTTACATGGTTTGCATGTTTTTATTGGTGTTTGTTTTCTT
ATTGTTAATTTTTTTCGTGTTAAGTTGTTCAATTTTAATTGATATCATGTTCAGGCTTATGATATAT
CTATTGATTATTGGCGTTTTTTAGAATGAATGTGAGGTGTTATGTTTTGTTTATTATATGTTTGAGG
TTCTTAATTGGTTATTTTTTATTATTTGGGGTTGTTGGTTATGATTGTTTTTATTTTGCAGGCTATT
GCTTTTTTAACTTTGTTGGAGCGCCATTTTTTGGGTGGTTCTCAGTGTCGTGTTGGTCCTAATAAGG
TGGGTTATTCTGGTGTTTTGCAGGCTTTATTTGATGGTTTAAAGTTGTTAAAGAAGGAGCAGTTGTT
GTTGTGTTTTTCTTCTTGATTATCTTTTTTGTTTATGCCTGTTTGTGGTTTTGTTTTGATGGTTTTT
TTTTGATTTACTTTGCCTTATTTTTTTTCTTTTTTGTCTTTTGAGTACTCTGGTGTTTTTTTGTTTT
GTCTTATAGGGGTTTCTGTTTATTTTATTATGCTTTCTGGTGTTTTTAGCGGTAGTAAATATTCTTT
TGTTGGTGGATTGCGTGCTTGCGTTCAGAGTTATTCTTATGAGATTGCTTTTTCTATTTATTTGTTG
GTTTTTTTGTTGTTTAATAAGGGTCTATGTTTGTCTTTTAGTTTTTGTTTATTTTTTTTTTTTGTTTT
TTTTTCCTTTTTTTTGTTTAGTTCTTGTTGATTTGCATCGGGCTCCTTTTGATTTTTCTGAGTGTGA
AAGTGAGTTGGTAAGGGGGTTTAATGTTGAGTATTCGGGAGTTGGTTTTGCTGCTTTGTTTTTAGGG
GAGTATGGTAATTTACTTTATTTTGGTTGTTTGACTTCTAGTTTGTTTTTTGGTATAAGTTTTTTTT
TTTTTTATTTTATTGTATGTATGATTGTTTTTTCTCGTAGAGCTTATCCTCGTTTTCGTTTTGATAA
GTTGATGGGTGTTTGTTGGTTTTTGTTTTTGCCTGTCGGTTTTTATTTTTTTGGTTTGTCTTTTGTT
GTTTTTATGTTGTGCTTATTTAGTTTAATTTTTTAGATTTTGTTATTTTTTTTTTATTTGGTTTTGAT
TAGTTTTTTATTTGTTTTATATGGAGTTGAGTAAGTTTAGTAGTTTGGGAGTTTTAGGTGTTTTTGT
TAATGTTTTAGTTTCTGGATTTTCTCATCAGGGTTTTCAGTCTAGTGTTTTTTTTAAGTTTGTTGTT
TTTTTTCTTTTGGTTTTTTGAATGAGTGGGTTATTGTTTCCTTTGTTTAGTCCTTGGGCTTGTGTGG
GTTTTTTGTTTTTTGTTACTAATTTTTCTTGATTGGGTGTTCGTACTTTTATTTTAGCTGTAGATAG
TTTTTTGATTTTTTTTGAGGGGGATCACTCTTGGGAGTGGTTTTCTAGGTTAGTTATGTTTTTTTCT
CATTGGTTGAGATTTTTGATAAGGGGGGTGGCTTTAACTTTGCGTATTAGTATTATTTTTTTAATTG
GTCATTTTTTAATGTTTACTGTTTTGGATATGAGTGTATTTTATTCTTTGTTTTTTTTGTTGTTTGT
GGTTCCGGTGGAGTTGTTTTTTGCTTTTTTACAGAGTTATATTTTTTTGACTTTGGTTTGTATGTTT
TTACTTAATATGATTTAGATTTATTTGCAGAATTATGTGTTTCCTATTCCTGGAAATTCTTATGTGT
TTTGTTGTTATTATATCCATAATTATTATTCTCATATTATTTTTTTTGGTTTTTTTGTGATGTTTTT
GGTTAGTGGTGGGGTTTATTTTTTTGGCAATTCTTTTAAGTTTAACTTGAAGCGGAGAGATAGTCGT
ATAATTGAATTAGTTTTACAGGTGTTGATTGTTAATTTTTTGATTATGATGGCAGGTCCTGGTTTTT
GGTTGATTCAGTATCAGGGACGTATGTTTCGTCAATCTGAGTTGGCTTTGAAGGTTATTGGTCATCA
ATGGTATTGGAGTTATGAGTATGGTGATAGTGGAAAATTATGTTTTGATTCATTTATGAAGTCTTTA
GATGATTTGTCTTTAGGGGATTTTCGGTTATTTGATGTTGATAATCGGTGTGTTTTGCCTGTAGGTG
TGAATGTTGGAGTGTATTGTACTTCTAGTGATGTTATTCATTCTTTTGCTATTCCTAAGTGTTTTAT
TAAGATGGATGCTTTGAATGGTTTGTTAACTAAGGTTACTTGTAATTTTTCTTGTTCTGGTTTGTTT
TTTGGGCAGTGTTCTGAAATTTGTGGTGCTAATCATAGGTTTATGCCTATTGTGTTGGAGTTGACTT
CTTTGGAGTGTTGGAAGGGTTGATCAGTTAATTATTTGCTGGGTTAACTTTTTTTAAATTTTATTGT
TATTTTTTTTTTTTCTTTTTTGGTTCCTTTTGGTATGTATTTGTTGTCTTTTTTTGTGTCTTTTAAG
GATTTTTATGGTGCTAAATTAAGTTCTTATGAATGTGGTTTTGATGTTGTGAAGAAGGTTCATGTTG
GTTTTAATTTGGTTTTTTTTTCTATTGTTTTGTTGTTTGTTGTTTTTGAGTTGGAAGTTTTAATTTT
TATTATTTTGATTCAGGGTGATTTTTATAGTTTATTGTCTTTTTTTTGTTTTTTTTTTTATGTTGTT
TTTAGTTTTTATATGGAGTGGTATTTTGGTAAGTTGATTTGGTTTTGTTAGTTGTTATTGTTTGTTT
GATATGTTCTTATTTTTTATTTTTTTTTGGTTGTTGTTTTTTTGTTTGTTCCTTATGGTAAATGGAG
TTATAGTTTTGGTTTTAGTGATTATTTTAATTTTACTTTTGTTTATAATTTTGAAGTTTGTTTGTTT
TTTTTAGTTTTGTTGTTGGTTTCTTTTATGGTTTTTGTTTATGGTTCTTTTTATATGGTTGGGGTTT
CTCGTTTGTTTTATTTTTTTTTTTTTTTTATTTTTGTTTGTGTTGAGGATGGGTGGCTTGATTGTTTT
TAGAGGTAGTATTGTTTTAACTTTGGTTTTTTGGGATTTTTTGGGGGTTAGTAGTTTTTTTTTGGTT
TTGTTTTATGGTAATGTTAGTGCTCGAAGGGGTGCTATGAGTACTGTGTTTACTAATCGTATTGGTG
ATTTTTGTATTTTTTTGTTTTTTAATGGTTTTGTTTTGTTTTCTATGAGTTTTTTGTCTTATCAGTT
TTTTGGTTCTTTGTTAGTTTTTATGTTGTTTGTTTCTTCTGTTATTAAGGGTGGTCAGTATCCTTTT
GGTAGCTGGTTGCCTAAGGCTATGGCTGCTCCTACTCCTGTTAGCTGTTTGGTTCATAGTAGTACTT

TAGTTACTGCTGGTGTTATGTTGATGGATTGTTATGTTTATATTTCTTTGAATTCTGATGTTTTGTC
TTTTGTTTTTTATGTTGGTTTTTTTACTATAGTTTTTTCTGGTTTTTGTGCTTTGGTGGAGGAGGAT
GCTAAGAAGATTGTTGCTTTGAGTACTATGTCTCAGATTGGTTTTTGTTTTTTTGGCTATTGGTAGGG
GTTTGCATTATTTGTCTTATGTTCATATGATTAGGCATTCTTTTTTTAAGAGATTGTTGTTTATGCA
GATAGGTTATTTGATTTTTATTAATTTTGGTCAACAGGATTATCGTGGTTATTCTTTTTTTGGTTTT
TGTGCTCCGGTTTTAGTTCAGTTGCAGATTTTTTTATCTGTGTTTTGTTTGTGTGGTTTGTTGTTTA
CTAGAGGTAGTTGTAGTAAGGAATATTTTATATCTCGTTTTTATTATGATTCTTATGGTTTTTTTTT
AGTTTTTTTTTATTTTTTTGGTGTGTTTTTGACTTTTTGTTATTGTTATCGGATGTTTTTTTTGTTT
CGTGTGGGGGCTTTTGGTTTTGATTATGTGGGTTTTTCTAGTAAGTTANTTTATTTTTCTTGTTTT
TTTTTGGTTTTTTTTTCTGTTGTTTTTACTTTTTGGTGGGTTTTTGGTTTGTTATCTTTTTCTGCGG
CTTTTAATCGTTTTGAGTTTTTGGTTGTTTATTTTTATTTGTTTTTTTGTTTATTGTTTTTGTGGTTA
TTTTTTTCGTTATTTTTTTTTCGTTATTTTGTGAAATTTTTTATGGATCATTATGCTTGTTTTATTT
ATAAAATTTTTCCTAGTTTTTTTTATTTTGATGTTTTTATTATGGGTTTTAATTATTTTTTTTTTGG
GTTATTTCGGTTATTTTCTTTTTTTTTTTTTTCTTGGTTTCGGGGGTTTTATCATGTTGGTGTTTTA
ATTGTTTTTTTTTTTTATGTTGTTTTTTTTTGTTTTTTTAGATTTTGTTATTTTTTGTTTTTTTTTTTAT
TATTGTTTTTGAGTTTTATTAATTTTTGTGTTGTTGATTATATTGTTTGGTGGAGGATTTTTGTTAT
TTGTACTTTTGTTTTTGTTTTTTTTTGTTGGTGGTGAGTTAGGTTTTGGGGGTTATTTGGTTAATTAT
TATGTTATTCAGGAAGTTTGTGGTTATTATTTTTTGGTTTTTGATGGTTGGAAGTTGCAATTTTTAT
TGCTTATGTTGAAGTCTGGTTCTTCTCCTTTTCATTTTTGACTTTTTAGTGTTTTGGGTGGTTTGGA
TAAGTGGTTTATTTTGTGGTTTTTAACTTTGCAAAAATTGCCTTATTTTGTTGTTTTGGTTAATTTT
TGTGGTGATTTTTTTTTTTTTGTTTTTGTTTTTTGGTATAATTTTTTGTTATTTTCAATTTTTTTTGT
TGCGTAGTTATCGTGATTTGTTAGTTGTGGGTTCTGCTGAATCTTTTAATTGGTTATTGTTATTGGG
TATTTTTTCTTTTAATGAAGTATTTGTTTTGTTTTTTTTTTTATTATTTTGTTATGTTTTTTTGTTGTC
TCTTATGTGTATGGGGGATTTTTAAATTTTTTGAGTTTAGAGATATTGATGTTTTTTTTTTAATGTTC
CTTTGAGAATTACTTTTTTTTTTAAAGGTGATTGTGTTGTTTGGTTCTTCTTTTTTTTGTTGGTTTTTA
TTATTTATTTTTGTTGTTGTTTATGCCTTTGATGTCTTTGGGTATGGGTTATTTGTTTTTTTTTGGTT
TCGATGATGAGTTTTAATTATGGTTTTAAGTATTATGATTATTTTGTTTATGTTTTGTTTTGTGTTG
GGTTGTTGTCTTGTTTTTAGTTGTTGTATTTATTTTTTTTTGTTGTTTTGTTGTTTTTTTTTTAGTCC
TTTTTTATTTTTTGTTTTTTTTTATGTTTTTTGTTTTATATGGTTTTTTTGATTGTTCATGGTTTGGT
TGTTTGTTTTTTTTTTGATTCTTTTAATTTTGTTTTTTTGTCTTTTATGAGTGTTTTTGTTATGGGGT
TTATTTGTGTGTCTGAATTGTTGAGTGGCTTAGTTTTTTATAGTTGTCTTGTAGTGTTTTTTAGTGT
TTGTTTTTTTTTATTCTGGTAGTTTTTTGATATTGTATGTTTTTTATGAGTTGACTATAGTACCTATT
TTGTTTTGTTTGTTAGGGTATGGTCGTCAGGTGGAGAAGGTTAGGGCTTGTTATTATTTAATTTTTT
ATACTTTGTTTTTTGGGATGCCTTACTTGTTTTTGTATAGTCATGTTTTTTTTTTTTTGAATTTTGT
TTATTATGATTTTTTTTGTTTCTTATGAATTTATTTTTTTGTTGAGTTTGTGTTTTTTTAGTTAAGTTT
CCTGTTTATTTTTTTCATGTTTGATTACCTAAGGTTCATGTTGAGGCTCCTACTAGTGCTAGAATGA
TTTTGGCTGGTGTTATGTTGAAGTTGGGAGGAGCAGGTGTTTATCGTATTAGTAAGTCTTTGAATTT
TTTTGGTTTTGAAATGTTGATTTTTTTTTTCTTTGATTAGGATGGTTTTTTGTTCTTTTATTTGTGTT
GTTCAGAGTGATTGTAAGTCTTTGGCGGCTTATTCTTCTGTTTGTCATATGGGTTTTGTATTGCTCT
CTGAGATTAGTATGGTTTATTATGGTAAGTCTATGGCTTTGGTGATGATGTTGGCTCATGGTTATAC
TTCTGTTTTAATGTTTTATTTTATTGGTGAGTTTTATCATATTGCTAATAGGCGTTTAATTTATTAT
TTGCGTGGATATTTTAATGTTAGTATGTTGTTTTGTTTGATGTTTTGTTTGACTATGGTTTCTAATT
TTAGTTTTCCTGTATCTATTTCTTTTTTTTTCTGAGTATTTGATGTTGAATTTTTTTAGGTCTGTTTT
TTATGTTGGTTTTTTGTTTTTGTTTTTTTTATTATTTGGTTTCTTTTTATTATTCTGTTTATATTTTG
GTTTGTTTTTTAGTTGGGGATAAGGTGAGTTATGTTTGTGATGGTCGTAGTGTTGTTTGTTTACCTT
TGGTTTTTATGATATATAATTTTTTTTGGTTTATTTTTGTTATTTAATTTTAAGTTTGATTTTGGTT
TAGGTTGTATTAAGATAGTATTACTTATTTTTAGTTTATTTAATGTGTTATTTTTTGTACACTGGTA
GTTTTTTGATTGTTTTATTAACGTTCCAGAATAATCGGCTATGCGTTTTAATTTTTGACTCTATTTG
TTGTGGTGCTATGAGTTTTTAGTTTGTTTTTATGTTGTTTTTTGTAAAATATTTTGATTTTTTTTAG
TTTCTTGTGGTATCAAAAATTTGTTTTTTGAACTGGATTAGTACCCAGGTAATCAAAATTTAATAAT
TCGGGAGTAAAGTTTTGTTTAAACCGAAAAAATATTGACTGACTTTAGATTTTTCTTTGGAACATGT
GATTTGCTGGAGAGCCCTCTTTTTTGGTGAATTTTGTTGGCACATGTATGATTGTTTAGTTTTTATT

TTATTTTGTAATGCTTTGTTGTTTTGGCATTAAAAACAGATATATATTTGGCTTATGAATTTATGTT
TCATGTGTTACTATTATGAATTTTTTTTGGATTAGTTTTTTATTTTTTTTTGAAATTGGAAAAGAAA
GTAATTTTTTTTTAATGTTTTAATGAATTTAATAAATAAGGTGGTACAAACCATCCGTCAATGGCCT
AAAGGGGCGTAAGTTGTAGTATGGTAGAAGTAAGGAAACTTGTTTCTATTTTTTGAAGTTTTTTTGT
TTTTTTAAGTTTTATTATTTGGTATTGCATATCAGTAGAAGTTTTTATCATAGTTATGAGTAATAGA
ATTAAAATGGTTAAATTGAATTTGTTTTTTTTTTACGAAATTAATAATATTTTTATTTTAGTATTGA
TATAACGTATTTTTATTTCTGTTTATTTGTTATTTATAGGTTAAGTTATACTGTTTTTGTTTGTAGA
TTTTTAAATTTTTGTTGCTGTTTTATAAATGTTGTGTGTTTTACATAGATTTTTATTTTTTTTTTTT
TTTTTTTTTTGATTAATTTTTTAATTAATTTATGATTTTTACTTTAGTAATTTTTATTATTTTGAGG
GGTGTTTGTTTTTGAATTTATGTTTTTGAACTGGTTTTGTTGCTAAATGTTTATTAAAAACTTAGGT
TTTTATGTAAAATTGTCTTCTGCTCTATGAGTTTTTAAATGGCAGCCTTAGCGTGATGGCGTAAAAG
TAGCGTAAGTGATTTGTTTTTTTAATGGTTTCAAGTATGAATGAAGTTTTTAGCAGTTTTTTTATTT
ACTTTTTATTTGAATTATTTTTTTGATTAAAAATTATTAGTTAAGGTATTACAAAGATAAGTCTTCG
GAAATTTTGTTTTGAATTTTGAAATTTTTGTTTTTAATTTTTTCTTGGGGATGGATTTTAAGAAAGT
TTTATACTATTGTTATTATTAAAAATTACTCCGGAGTTAACAGGGTTGTAGACATATAAATAGATTT
TTATATTAGTGTGCTGCGCTACATCGATGTTGTATATTTTTTTTGATAATGGAGAGGTTTTTTTTAT
TTTGAGACTGTTCTTCTTGTATAAAAAATTGACTTGATATTAGTTTAGTTCGTCGTGAGACAGAGCG
GTTTATCTTGTGTATTTTTGGTTTTTGGCGGTGTTAGTACGAAAGGAATGCAATGTGGGTTTATATT
TATGACTTTTTTATTTTGATGGGTTTTT

>O.ochengi(M.Blaxter)
ATTTTTTGTGGAATGACTTTTGGTAATGGTATGAAGCAGAGTATTATTAATACTGTGAATCATAAGA
CTATTGGTACTTATTATATTGTTTTAGGTTATTGGGCTGGTTTAGGTGGTTCTGTTTTATCTATGTT
GATTCGTTTTGAATTGTCTAGTCCTGGTGGTTATTTGTTTTTTGGAAGTGGTCAGGTTTATAATTCT
GTTCTTACTATGCATGGTGTTTTGATGATTTTTTTTTTTGGTTATGCCTATTTTGATTGGTGGCTTTG
GTAATTGGATGTTGCCTTTAATATTAGGGGCTCCTGAGATGGCGTTTCCTCGGGTAAATGCTTTATC
TTTTTGATTTACTTTTGTGGCTTTGTTGATAGTTTATCAGTCTTTTTTTATTGGGGGTGGCCCTGGT
AGAAGTTGGACTTTTTATCCTCCTCTTAGGGTTGAAGGTCAACCAGAATTGTCTTTAGATACTATGA
TTTTAGGTTTACATACTGTAGGAATTGGTTCTTTGTTGGGTGCTATTAATTTTATGGTAACTACTCA
GAATATACGGTCTACTGCTGTGACTTTGGATCAAATTAGTATGTTTGTTTGGACTTCTTATTTGACT
TCTTTTTTGTTAGTTTTGTCTGTGCCTGTTTTGGCTGGTTCTTTATTGTTTTTGTTGTTGGATCGTA
ATTTTAATACTTCTTTTTATGATACTAAGAAGGGGGGTAATCCTTTGTTGTATCAGCATTTGTTTTG
ATTTTTTGGTCATCCTGAGGTGTATGTTATTATTTTACCTGTTTTTGGTATTATTAGGGAAGCGGTT
TTATTTTTGACTGATAAGGATCGTTTGTTTGGTCAGACTAGGATAACTTTTGCTTCTATTTGGATTG
CTGTTTTAGGTACTTCTGTGTGAGGTCATCATATGTATACGGCTGGTTTGGATATTGATACTCGTAC
TTATTTTAGTGCTGCTACTATGATTATCGCTATTCCTAGAGCTGTTAAGATTTTTAATTGGTTAGGT
ACTTTTTTTGGTTCTAGTCAAAAGGTGCAGCCGTTATGATGTTGAACTTATAGTTTTATTTTTCTTT
TTACTGTGGGTGGATTAAGTGGAATTATTCTGAGGGCTGCTAGTTTGGATATTATTTTGCACGATAC
TTATTATGTTGTGGCTCATTTTCATTATACTTTGAGTTTGGGTGCTATTTATGGTATTTTTTGTGGT
TTTTGTTTGTGACTTCCTTATATGTATGGTATTTCTTTTGATAGGGTTATGATAATAGCTGTTTTTG
TTTGTTTTTTTGTTGGTACTAATATGACATTTTTTCCTATGCATTTTGCTGGTTTGCAGGGTATGCC
TCGTAAGATTTTGGATTATCCTGATTGTTATTCTACTTTTCAGATTATTTCTTCTTTAGGTTCTGTT
ATTACTTTTGTTGGTTTTGTTTTGTTTAATTATTTGTTGGTTGATTCTATTTTTTTTTCTCGTTTTT
TGGGGGTTTCTTTTTATAATTATCATAGTCCGGCTTATGCTTTAAATGTTCCTCCTTTGCCGGATTC
TTTTACTGAAGAGGCTTTTATTATAGGTCTTCATTGGAAGATTATTAGTAAGGATACTCCTTCTTAT
AGGTATCGTCGGGTTGGTTATGGTTATCATAGTAAGTAAATTTTTTTTTTATGTTAGGGTATTTTTTG
CTTTTGTTTTTTTTTTGTTTGAGTTTTTTGGATTGGGATCCGTTGAAGAGTTGTGTTATGATGTGTTT
GGGTATTATGTCTATAAGTTGTTATGTTTCTTTGGGTATTCATGTATGATATTCTTATTTTGTTGTT
TTAATTTTTTTTTAGTGGTATTTTTTTCTTTGTTGACTTATTTTTGTAGTATGAGTAATTTTGTTTTTT
ATTATAATTATTTTTTTTTTTTTTTCTTTGTTTTTGGTTAGTTTTTTTTTTTGTATTTGTTGTAGATTT
TGATTTTTTTTTTGTTTTTTTTGTGATTTTAATTTTCTTTATGTTTGTTATGATTTTAGTTATTATTAT
GTTTTTTGAGTAGTTTTTGTTTTGTTTTTGTTTTTGGTTTTGGTTAGATTTAGGTTTAATGGTTTTG

GTTATATGCGTAGCTTGTAGATTGTTATTTTTAATTCTTTAGTTTTTTTGCCTGCTAGTTTTACTTT
GAGTTATATGTGAAATTTTGGTAGTATGTTGGGTATTATGTTGATGTCTCAGATTTTAACTGGTTTT
TTTTTGACTTTTTACTATACGGCTGGGGAAGCTTTTAGGTCTGTTCAGTATATTATGTTTGAGGTTA
ATTTGGGTTGGTTGTTGCGTATTATGCATTCTAATGGGGCTTCTATGTTTTTTTTGTTTATTTATTT
ACATATTTTTAAGGGTCTGATTTATGGTAGATATCGTCTTATTGGTGTATGGTTGAGTGGTATTTTT
ATTTATTTTTTATTGATAGGTATTGCTTTTACTGGTTATGTTTTGATTTGAGGTCAAATAAGTTATT
GGGCGGCAGTAGTTATTACTAGTTTAATAACTTCTGTTCCTTATTTAGGTAAGTATTTAGTTTGATG
GATTTGGGGGAGTTTTAGTGTTTGTGAGAACACTTTAAAGTTTTTTTATTCTGTTCATTTTATTTTA
CCTTGGTCTTTGATAGTTTTAGTTGTTTTTCATTTGTTTTTTTTGCATTTTACTGGTTCTAGTTCTA
GTTTGTATTGTCACGGGGATTATGATAAGATTCATTTTTTTCCTAGTTTTTGATTGAAGGATGGTTT
TGATATTTTTTTTTATTTTTTTTTTGATTTTGTTTAGACTTTATTTTTCTTTTGATTTAAGTGATCCT
ATGATTTTTGTGGAGTCTGATTCTATGGCTAGTCCTGCGCATGTTGTACCTGAGTGATATTTTTTAT
TTGCTTTTACTATCTTACGTTCTGTTCCTAGTAAGTTATTAGGGGTTATTTTAATATTTAGTTCTGT
TTTTGTATTGACTATTCTTGTTTGACCTGATAGTTATCAGTCTATTTTGGATAATTTTTTATATTTT
TTTGTTATGTGTTTTGTTTGGATTTTTTTTTGGTTAACTTGAGCTGGTCATTATCCTACTGATTATC
CTTTTAACTATTTTAATTTGTTTTGTACTTTTTTTTTATTTTTGTTGTATTTTTTTTTGTTTGTTTAAT
TAATTTTTTTAGTGATAAGTTGTTTAGTTAAATTTTGTTGAAGTTTCGTAAGTACCATAAAATGGAG
TATAGTTATTATCCTTTGATAGTTGGGGCGGGTATTTTAGGTTTTGATGTTAGTTTGGTTTTATTTA
TAAGTATAGGTATGTTTTATTCTATTTTTATTTGTTTTTTGTATTTGGTTTATGTTTTTTTTTTGTG
AATCAAGGATGTTATTTTAGAGGACATTAGTGGTCAATATTCTTTTTATGACTATCGTATGTTTAAT
CAAGGTTTTCGTTTGTTTCTTTTTAGTGAGTTAACTTTGTTTGTTTCTATTTTTTGAACTTTTTTGG
ATACAGCTTTGTGTCCTTTAACTTGGTTAGGTGGGGTTTGGTCTCCATTTGGGATTTTATCTCCTGA
TTATTTGGGTTTGAATGGTATGGCTAGTTTGTTTTTAATGATAAATAGGCAAGTTTTGAAGTATTCT
CGTCGTTATTTGTGTTTGAGTAGTTCTAAGTGTGAAGAGTTTTTGTTAGTTTGTATTTTTGTTGGAG
TTGGTTTTTTATGTTTTCAGTTTTATGAATATAATAACAATTCGTTTGTTATGAGTGATAGTGTTTA
TGGTAGTATTTTTTATATGGGTACTGGTTTACATGGTTTGCATGTTTTTATTGGTGTTTGTTTTCTT
ATTGTTAATTTTTTTCGTGTTAAGTTGTTCAATTTTAATTGATATCATGTTCAGGCTTATGATATAT
CTATTGATTATTGGCGTTTTTTAGAATGAATGTGAGGTGTTATGTTTTGTTTATTATATGTTTGAGG
TTCTTAAGAATTTTTGTTTTTTTGGTTATTTTTTATTATTTGGGGTTGTTGGTTATGATTGTTTTTA
TTTTGCAGGCTATTGCTTTTTTAACTTTGTTGGAGCGCCATTTTTTGGGTGGTTCTCAGTGTCGTGT
TGGTCCTAATAAGGTGGGTTATTCTGGTGTTTTGCAGGCTTTATTTGATGGTTTAAAGTTGTTAAAG
AAGGAGCAGTTGTTGTTGTGTTTTTCTTCTTGATTATCTTTTTTGTTTATGCCTGTTTGTGGTTTTG
TTTTGATGGTTTTTTTTTGATTTACTTTGCCTTATTTTTTTTCTTTTTTGTCTTTTGAGTACTCTGG
TGTTTTTTTGTTTTGTCTTATAGGGGTTTCTGTTTATTTTATTATGCTTTCTGGTGTTTTTAGCGGT
AGTAAATATTCTTTTGTTGGTGGATTGCGTGCTTGCGTTCAGAGTTATTCTTATGAGATTGCTTTTT
CTATTTATTTGTTGGTTTTTTTGTTGTTTAATAAGGGTCTATGTTTGTCTTTTAGTTTTTGTTTATT
TTTTTTTTTGTTTTTTTTTCCTTTTTTTTGTTTAGTTCTTGTTGATTTGCATCGGGCTCCTTTTGAT
TTTTCTGAGTGTGAAAGTGAGTTGGTAAGGGGGTTTAATGTTGAGTATTCGGGAGTTGGTTTTGCTG
CTTTGTTTTTAGGGGAGTATGGTAATTTACTTTATTTTGGTTGTTTGACTTCTAGTTTGTTTTTTGG
TATAAGTTTTTTTTTTTTTATTTTATTGTATGTATGATTGTTTTTTCTCGTAGAGCTTATCCTCGT
TTTCGTTTTGATAAGTTGATGGGTGTTTGTTGGTTTTTGTTTTTGCCTGTCGGTTTTTATTTTTTTG
GTTTGTCTTTTGTTGTTTTTATGTTGTATTTTGTTATTTTTTTTTTATTTGGTTTTGATTAGTTTTTT
ATTTGTTTTATATGGAGTTGAGTAAGTTTAGTAGTTTGGGAGTTTTAGGTGTTTTTGTTAATGTTTT
AGTTTCTGGATTTTCTCATCAGGGTTTTCAGTCTAGTGTTTTTTTTAAGTTTGTTGTTTTTTTTCTT
TTGGTTTTTTGAATGAGTGGGTTATTGTTTCCTTTGTTTAGTCCTTGGGCTTGTGTGGGTTTTTTGT
TTTTTGTTACTAATTTTTCTTGATTGGGTGTTCGTACTTTTATTTTAGCTGTAGATAGTTTTTTGAT
TTTTTTTGAGGGGGATCACTCTTGGGAGTGGTTTTCTAGGTTAGTTATGTTTTTTTCTCATTGGTTG
AGATTTTTGATAAGGGGGGTGGCTTTAACTTTGCGTATTAGTATTATTTTTTTAATTGGTCATTTTT
TAATGTTTACTGTTTTGGATATGAGTGTATTTTATTCTTTGTTTTTTTTGTTGTTTGTGGTTCCGGT
GGAGTTGTTTTTTGCTTTTTTACAGAGTTATATTTTTTTGACTTTGGTTTGTATGTTTTTACTTAAT
ATGATTTAGATTTATTTGCAGAATTATGTGTTTCCTATTCCTGGAAATTCTTATGTGTTTTGTTGTT
ATTATATCCATAATTATTATTCTCATATTATTTTTTTTGGTTTTTTTGTGATGTTTTTGGTTAGTGG

TGGGGTTTATTTTTTTGGCAATTCTTTTAAGTTTAACTTGAAGCGGAGAGATAGTCGTATAATTGAA
TTAGTTTTACAGGTGTTGATTGTTAATTTTTTGATTATGATGGCAGGTCCTGGTTTTTGGTTGATTC
AGTATCAGGGACGTATGTTTCGTCAATCTGAGTTGGCTTTGAAGGTTATTGGTCATCAATGGTATTG
GAGTTATGAGTATGGTGATAGTGGAAAATTATGTTTTGATTCATTTATGAAGTCTTTAGATGATTTG
TCTTTAGGGGATTTTCGGTTATTTGATGTTGATAATCGGTGTGTTTTGCCTGTAGGTGTGAATGTTG
GAGTGTATTGTACTTCTAGTGATGTTATTCATTCTTTTGCTATTCCTAAGTGTTTTATTAAGATGGA
TGCTTTGAATGGTTTGTTAACTAAGGTTACTTGTAATTTTTCTTGTTCTGGTTTGTTTTTTGGGCAG
TGTTCTGAAATTTGTGGTGCTAATCATAGGTTTATGCCTATTGTGTTGGAGTTGACTTCTTTGGAGT
GTTGGAAGGGTTGATCAGTTAATTATTTGCTGGGTTAACTTTTTTTAAATTTTATTGTTATTTTTTT
TTTTTCTTTTTTGGTTCCTTTTGGTATGTATTTGTTGTCTTTTTTTGTGTCTTTTAAGGATTTTTAT
GGTGCTAAATTAAGTTCTTATGAATGTGGTTTTGATGTTGTGAAGAAGGTTCATGTTGGTTTTAATT
TGGTTTTTTTTTCTATTGTTTTGTTGTTTGTTGTTTTTGAGTTGGAAGTTTTAATTTTTATTATTTT
GATTCAGGGTGATTTTTATAGTTTATTGTCTTTTTTTTGTTTTTTTTTTTATGTTGTTTTTAGTTTT
TATATGGAGTGGTATTTTGGTAAGTTGATTTGGTTTTGTTAGTTGTTATTGTTTGTTTGATATGTTC
TTATTTTTTATTTTTTTTTGGTTGTTGTTTTTTTGTTTGTTCCTTATGGTAAATGGAGTTATAGTTT
TGGTTTTAGTGATTATTTTAATTTTACTTTTGTTTATAATTTTGAAGTTTGTTTGTTTTTTTTAGTT
TTGTTGTTGGTTTCTTTTATGGTTTTTGTTTATGGTTCTTTTTATATGGTTGGGGTTTCTCGTTTGT
TTTATTTTTTTTTTTTTTTATTTTTGTTTGTGTTGAGGATGGGTGGCTTGATTGTTTTTAGAGGTAG
TATTGTTTTAACTTTGGTTTTTTGGGATTTTTTGGGGGTTAGTAGTTTTTTTTTGGTTTTGTTTTAT
GGTAATGTTAGTGCTCGAAGGGGTGCTATGAGTACTGTGTTTACTAATCGTATTGGTGATTTTTGTA
TTTTTTTGTTTTTTAATGGTTTTGTTTTGTTTTCTATGAGTTTTTTGTCTTATCAGTTTTTTGGTTC
TTTGTTAGTTTTTATGTTGTTTGTTTCTTCTGTTATTAAGGGTGGTCAGTATCCTTTTGGTAGCTGG
TTGCCTAAGGCTATGGCTGCTCCTACTCCTGTTAGCTGTTTGGTTCATAGTAGTACTTTAGTTACTG
CTGGTGTTATGTTGATGGATTGTTATGTTTATATTTCTTTGAATTCTGATGTTTTGTCTTTTGTTTT
TTATGTTGGTTTTTTTACTATAGTTTTTTCTGGTTTTTGTGCTTTGGTGGAGGAGGATGCTAAGAAG
ATTGTTGCTTTGAGTACTATGTCTCAGATTGGTTTTTGTTTTTTGGCTATTGGTAGGGGTTTGCATT
ATTTGTCTTATGTTCATATGATTAGGCATTCTTTTTTTAAGAGATTGTTGTTTATGCAGATAGGTTA
TTTGATTTTTATTAATTTTGGTCAACAGGATTATCGTGGTTATTCTTTTTTTGGTTTTTGTGCTCCG
GTTTTAGTTCAGTTGCAGATTTTTTTATCTGTGTTTTGTTTGTGTGGTTTGTTGTTTACTAGAGGTA
GTTGTAGTAAGGAATATTTTATATCTCGTTTTTATTATGATTCTTATGGTTTTTTTTTAGTTTTTTT
TTATTTTTTTGGTGTGTTTTTGACTTTTTGTTATTGTTATCGGATGTTTTTTTTGTTTCGTGTGGGG
GCTTCTGGTTTTGATTATGTGGGTTTTTCTAGTAAGTTATTTTATTTTTCTTGTTTTTTTTTGGTTT
TTTTTTCTGTTGTTTTTACTTTTTGGTGGGTTTTTGGTTTGTTATCTTTTTCTGCGGCTTTTAATCG
TTTTGAGTTTTTGGTTGTTTATTTTTATTTGTTTTTTGTTTATTGTTTTTGTGGTTATTTTTTTCGT
TATTATTTTGTTTCGTTATTTTGTAAATTTTTTATGGATCATTATGCTTGTTTTATTTATAAAATTT
TTCCTAGTTTTTTTTATTTTGATGTTTTTATTATGGGTTTTAATTATTTTTTTTTTGGGTTATTTCG
GTTATTTTCTTTTTTTTTTTTTTTCTTGGTTTCGGGGGTTTTATCATGTTGGTGTTTTAATTGTTTTT
TTTTTTATGTTGTTTTTTTTGTTTTTTTAGATTTGTTATTTTTTGTTTTTTTTTTATTATTGTTTT
TGAGTTTTATTAATTTTTGTGTTGTTGATTATATTGTTTGGTGGAGGATTTTTGTTATTTGTACTTT
TGTTTTTGTTTTTTTTGTTGGTGGTGAGTTAGGTTTTGGGGGTTATTTGGTTAATTATTATGTTATT
CAGGAAGTTTGTGGTTATTATTTTTTGGTTTTTGATGGTTGGAAGTTGCAATTTTTATTGCTTATGT
TGAAGTCTGGTTCTTCTCCTTTTCATTTTTGACTTTTTAGTGTTTTGGGTGGTTTGGATAAGTGGTT
TATTTTGTGGTTTTTAACTTTGCAAAAATTGCCTTATTTTGTTGTTTTGGTTAATTTTTGTGGTGAT
TTTTTTTTTTTGTTTTTGTTTTTTGGTATAATTTTTTGTTATTTTCAATTTTTTTTGTTGCGTAGTT
ATCGTGATTTGTTAGTTGTGGGTTCTGCTGAATCTTTTAATTGGTTATTGTTATTGGGTATTTTTTC
TTTTAATGAAGTATTTGTTTTGTTTTTTTTTTTATTATTTTGTTATGTTTTTTGTTGTCTCTTATGTG
TATGGGGGATTTTTAAATTTTTTGAGTTTAGAGATATTGATGTTTTTTTTTAATGTTCCTTTGAGAA
TTACTTTTTTTTTAAAGGTGATTGTGTTGTTTGGTTCTTCTTTTTTTTGTTGGTTTTTATTATTTATT
TTTGTTGTTGTTTATGCCTTTGATGTCTTTGGGTATGGGTTATTTGTTTTTTTTTGGTTTCGATGATG
AGTTTTAATTATGGTTTTAAGTATTATGATTATTTTGTTTATGTTTTGTTTTGTGTTGGGTTGTTGT
CTTGTTTTTAGTTGTTGTATTTATTTTTTTTTTGTTGTTTTGTTGTTTTTTTTTAGTCCTTTTTTATT
TTTTGTTTTTTTTATGTTTTTTGTTTTATATGGTTTTTTTGATTGTTCATGGTTTGGTTGTTTGTTT

TTTTTTGATTCTTTTAATTTTGTTTTTTTGTCTTTTATGAGTGTTTTTGTTATGGGGTTTATTTGTG
TGTCTGAATTGTTGAGTGGCTTAGTTTTTTATAGTTGTCTTGTAGTGTTTTTTAGTGTTTGTTTTTT
TTATTCTGGTAGTTTTTTGATATTGTATGTTTTTTATGAGTTGACTATAGTACCTATTTTGTTTTGT
TTGTTAGGGTATGGTCGTCAGGTGGAGAAGGTTAGGGCTTGTTATTATTTAATTTTTTATACTTTGT
TTTTTGGGATGCCTTATTTGTTTTTGTATAGTCATGTTTTTTTTTTTTTGAATTTTGTTTATTATGA
TTTTTTTGTTTCTTATGAATTTATTTTTTTGTTGAGTTTGTGTTTTTTAGTTAAGTTTCCTGTTTAT
TTTTTTCATGTTTGATTACCTAAGGTTCATGTTGAGGCTCCTACTAGTGCTAGAATGATTTTGGCTG
GTGTTATGTTGAAGTTGGGAGGAGCAGGTGTTTATCGTATTAGTAAGTCTTTGAATTTTTTTGGTTT
TGAAATGTTGATTTTTTTTTCTTTGATTAGGATGGTTTTTTGTTCTTTTATTTGTGTTGTTCAGAGT
GATTGTAAGTCTTTGGCGGCTTATTCTTCTGTTTGTCATATGGGTTTTGTATTGCTCTCTGAGATTA
GTATGGTTTATTATGGTAAGTCTATGGCTTTGGTGATGATGTTGGCTCATGGTTATACTTCTGTTTT
AATGTTTTATTTTATTGGTGAGTTTTATCATATTGCTAATAGGCGTTTAATTTATTATTTGCGTGGA
TATTTTAATGTTAGTATGTTGTTTTGTTTGATGTTTTGTTTGACTATGGTTTCTAATTTTAGTTTTC
CTGTATCTATTTCTTTTTTTTCTGAGTATTTGATGTTGAATTTTTTTAGGTCTGTTTTTTATGTTGG
TTTTTTGTTTTTGTTTTTTTATTATTTGGTTTCTTTTTATTATTCTGTTTATATTTTGGTTTGTTTT
TTAGTTGGGGATAAGGTGAGTTATGTTTGTGATGGTCGTAGTGTTGTTTGTTTACCTTTGGTTTTTA
TGATATATAATTTTTTTTGGTTTATTTTTGTTATTTAATTTTAAGTTTGATTTTGGTTTAGGTTGTA
TTAAGATAGTATTACTTATTTTTAGTTTATTTAATGTGTTATTTTTTGTACACTGGTAGTTTTTTGA
TTGTTTTATTAACGTTCCAGAATAATCGGCTATGCGTTTTAATTTTTGACTCTATTTGTTGTGGTGC
TATGAGTTTTTAGTTTGTTTTTATGTTGTTTTTTGTAAAATATTTTGATTTTTTTTAGTTTCTTGTG
GTATCAAAAATTTGTTTTTTGAACTGGATTAGTACCCAGGTAATCAAAATTTAATAATTCGGGAGTA
AAGTTTTGTTTAAACCGAAAAAATATTGACTGACTTTAGATTTTTCTTTGGAACATGTGATTTGCTG
GAGAGCCCTCTTTTTTGGTGAATTTTGTTGGCACATGTATGATTGTTTAGTTTTTATTTTATTTTGT
AATGCTTTGTTGTTTTGGCATTAAAAACAGATATATATTTGGCTTATGAATTTATGTTTCATGTGTT
ACTATTATGAATTTTTTTTGGATTAGTTTTTTATTTTTTTTTGAAATTGGAAAAGAAAGTAATTTTT
TTTTAATGTTTTAATGAATTTAATAAATAAGGTGGTACAAACCATCCGTCAATGGCCTAAAGGGGCG
TAAGTTGTAGTATGGTAGAAGTAAGGAAACTTGTTTCTATTTTTTGAAGTTTTTTTGTTTTTTTAAG
TTTTATTATTTGGTATTGCATATCAGTAGAAGTTTTTATCATAGTTATGAGTAATAGAATTAAAATG
GTTAAATTGAATTTGTTTTTTTTTTTACGAAATTAATAATATTTTTATTTTAGTATTGATATAACGTA
TTTTTATTTCTGTTTATTTGTTATTTATAGGTTAAGTTATACTGTTTTTGTTTGTAGATTTTTAAAT
TTTTGTTGCTGTTTTATAAATGTTGTGTGTTTTACATAGATTTTTATTTTTTTTTTTTTTTTTTTTT
TGATTAATTTTTTAATTAATTTATGATTTTTACTTTAGTAATTTTTATTATTTTGAGGGGTGTTTGT
TTTTGAATTTATGTTTTTGAACTGGTTTTGTTGCTAAATGTTTATTAAAAACTTAGGTTTTTATGTA
AAATTGTCTTCTGCTCTATGAGTTTTTAAATGGCAGCCTTAGCGTGATGGCGTAAAAGTAGCGTAAG
TGATTTGTTTTTTTTAATGGTTTCAAGTATGAATGAAGTTTTTAGCAGTTTTTTTATTTACTTTTTAT
TTGAATTATTTTTTTGATTAAAAATTATTAGTTAAGGTATTACAAAGATAAGTCTTCGGAAATTTTG
TTTTGAATTTTGAAATTTTTGTTTTTAATTTTTTCTTGGGGATGGATTTTAAGAAAGTTTTATACTA
TTGTTATTATTAAAAATTACTCCGGAGTTAACAGGGTTGTAGACATATAAATAGATTTTTATATTAG
TGTGCTGCGCTACATCGATGTTGTATATTTTTTTTGATAATGGAGAGGTTTTTTTTATTTTGAGACT
GTTCTTCTTGTATAAAAAATTGACTTGATATTAGTTTAGTTCGTCGTGAGACAGAGCGGTTTATCTT
GTGTATTTTTGGTTTTTGGCGGTGTTAGTACGAAAGGAATGCAATGTGGGTTTATATTTATGACTTT
TTTATTTTGATGGGTTTTT

>O.ochengiM3(KX181289)
ATTTTTTGTGGAATGACTTTTGGTAATGGTATGAAGCAGAGTATTATTAATACTGTGAATCATAAGA
CTATTGGTACTTATTATATTGTTTTAGGTTATTGGGCTGGTTTAGGTGGTTCTGTTTTATCTATGTT
GATTCGTTTTGAATTGTCTAGTCCTGGTGGTTATTTGTTTTTTTGGAAGTGGTCAGGTTTATAATTCT
GTTCTTACTATGCATGGTGTTTTGATGATTTTTTTTTTTGGTTATGCCTATTTTGATTGGTGGCTTTG
GTAATTGGATGTTGCCTTTAATATTAGGGGCTCCTGAGATGGCGTTTCCTCGGGTAAATGCTTTATC
TTTTTGATTTACTTTTGTGGCTTTGTTGATAGTTTATCAGTCTTTTTTTATTGGGGGTGGCCCTGGT
AGAAGTTGGACTTTTTATCCTCCTCTTAGGGTTGAAGGTCAACCAGAATTGTCTTTAGATACTATGA
TTTTAGGTTTACATACTGTAGGAATTGGTTCTTTGTTGGGTGCTATTAATTTTATGGTAACTACTCA

GAATATACGGTCTACTGCTGTGACTTTGGATCAAATTAGTATGTTTGTTTGGACTTCTTATTTGACT
TCTTTTTTGTTAGTTTTGTCTGTGCCTGTTTTGGCTGGTTCTTTATTGTTTTTGTTGTTGGATCGTA
ATTTTAATACTTCTTTTTATGATACTAAGAAGGGGGGTAATCCTTTGTTGTATCAGCATTTGTTTTG
ATTTTTTGGTCATCCTGAGGTGTATGTTATTATTTTACCTGTTTTTGGTATTATTAGGGAAGCGGTT
TTATTTTTGACTGATAAGGATCGTTTGTTTGGTCAGACTAGGATAACTTTTGCTTCTATTTGGATTG
CTGTTTTAGGTACTTCTGTGTGAGGTCATCATATGTATACGGCTGGTTTGGATATTGATACTCGTAC
TTATTTTAGTGCTGCTACTATGATTATCGCTATTCCTAGAGCTGTTAAGATTTTTAATTGGTTAGGT
ACTTTTTTTGGTTCTAGTCAAAAGGTGCAGCCGTTATGATGTTGAACTTATAGTTTTATTTTTCTTT
TTACTGTGGGTGGATTAAGTGGAATTATTCTGAGGGCTGCTAGTTTGGATATTATTTTGCACGATAC
TTATTATGTTGTGGCTCATTTTCATTATACTTTGAGTTTGGGTGCTATTTATGGTATTTTTTGTGGT
TTTTGTTTGTGACTTCCTTATATGTATGGTATTTCTTTTGATAGGGTTATGATAATAGCTGTTTTTG
TTTGTTTTTTTGTTGGTACTAATATGACATTTTTTCCTATGCATTTTGCTGGTTTGCAGGGTATGCC
TCGTAAGATTTTGGATTATCCTGATTGTTATTCTACTTTTCAGATTATTTCTTCTTTAGGTTCTGTT
ATTACTTTTGTTGGTTTTGTTTTGTTTAATTATTTGTTGGTTGATTCTATTTTTTTTTCTCGTTTTT
TGGGGGTTTCTTTTTATAATTATCATAGTCCGGCTTATGCTTTAAATGTTCCTCCTTTGCCGGATTC
TTTTACTGAAGAGGCTTTTATTATAGGTCTTCATTGGAAGATTATTAGTAAGGATACTCCTTCTTAT
AGGTATCGTCGGGTTGGTTATGGTTATCATAGTAAGTAAATTTTTTTTTATGTTAGGGTATTTTTTG
CTTTTGTTTTTTTTTGTTTGAGTTTTTTGGATTGGATCCGTTGAAGAGTTGTGTTATGATGTGTTT
GGGTATTATGTCTATAAGTTGTTATGTTTCTTTGGGTATTCATGTATGATATTCTTATTTTGTTGTT
TTAATTTTTTTTAGTGGTATTTTTTCTTTGTTGACTTATTTTTGTAGTATGAGTAATTTTGTTTTTT
ATTATAATTATTTTTTTTTTTTTTTCTTTGTTTTTGGTTAGTTTTTTTTTTGTATTTGTTGTAGATTT
TGATTTTTTTTTGTTTTTTTGTGATTTTAATTTCTTTATGTTTGTTATGATTTTAGTTATTATTAT
GTTTTTTGAGTAGTTTTTGTTTTGTTTTTGTTTTTGGTTTTGGTTAGATTTAGGTTTAATGGTTTTG
GTTATATGCGTAGCTTGTAGATTGTTATTTTTAATTCTTTAGTTTTTTTGCCTGCTAGTTTTACTTT
GAGTTATATGTGAAATTTTGGTAGTATGTTGGGTATTATGTTGATGTCTCAGATTTTAACTGGTTTT
TTTTTGACTTTTTACTATACGGCTGGGGAAGCTTTTAGGTCTGTTCAGTATATTATGTTTGAGGTTA
ATTTGGGTTGGTTGTTGCGTATTATGCATTCTAATGGGGCTTCTATGTTTTTTTTTGTTTATTTATTT
ACATATTTTTAAGGGTCTGATTTATGGTAGATATCGTCTTATTGGTGTATGGTTGAGTGGTATTTTT
ATTTATTTTTTATTGATAGGTATTGCTTTTACTGGTTATGTTTTGATTTGAGGTCAAATAAGTTATT
GGGCGGCAGTAGTTATTACTAGTTTAATAACTTCTGTTCCTTATTTAGGTAAGTATTTAGTTTGATG
GATTTGGGGGAGTTTTAGTGTTTGTGAGAACACTTTAAAGTTTTTTTATTCTGTTCATTTTATTTTA
CCTTGGTCTTTGATAGTTTTAGTTGTTTTTCATTTGTTTTTTTTGCATTTTACTGGTTCTAGTTCTA
GTTTGTATTGTCACGGGGATTATGATAAGATTCATTTTTTTCCTAGTTTTTGATTGAAGGATGGTTT
TGATATTTTTTTTTATTTTTTTTTGATTTTGTTTAGACTTTATTTTTCTTTTGATTTAAGTGATCCT
ATGATTTTTGTGGAGTCTGATTCTATGGCTAGTCCTGCGCATGTTGTACCTGAGTGATATTTTTTAT
TTGCTTTTACTATCTTACGTTCTGTTCCTAGTAAGTTATTAGGGGTTATTTTAATATTTAGTTCTGT
TTTTGTATTGACTATTCTTGTTTGACCTGATAGTTATCAGTCTATTTTGGATAATTTTTTATATTTT
TTTGTTATGTGTTTTGTTTGGATTTTTTTTTGGTTAACTTGAGCTGGTCATTATCCTACTGATTATC
CTTTTAACTATTTTAATTTGTTTTGTACTTTTTTTTTATTTTTGTTGTATTTTTTTTTGTTTGTTTAAT
TAATTTTTTTTAGTGATAAGTTGTTTAGTTAAATTTTGTTGAAGTTTCGTAAGTACCATAAAATGGAG
TATAGTTATTATCCTTTGATAGTTGGGGCGGGTATTTTAGGTTTTGATGTTAGTTTGGTTTTATTTA
TAAGTATAGGTATGTTTTATTCTATTTTTATTTGTTTTTTGTATTTGGTTTATGTTTTTTTTTTGTG
AATCAAGGATGTTATTTTAGAGGACATTAGTGGTCAATATTCTTTTTATGACTATCGTATGTTTAAT
CAAGGTTTTCGTTTGTTTCTTTTTAGTGAGTTAACTTTGTTTGTTTCTATTTTTTGAACTTTTTTGG
ATACAGCTTTGTGTCCTTTAACTTGGTTAGGTGGGGTTTGGTCTCCATTTGGGATTTTATCTCCTGA
TTATTTGGGTTTGAATGGTATGGCTAGTTTGTTTTTAATGATAAATAGGCAAGTTTTGAAGTATTCT
CGTCGTTATTTGTGTTTGAGTAGTTCTAAGTGTGAAGAGTTTTTGTTAGTTTGTATTTTTGTTGGAG
TTGGTTTTTTATGTTTTCAGTTTTATGAATATAATAACAATTCGTTTGTTATGAGTGATAGTGTTTA
TGGTAGTATTTTTTATATGGGTACTGGTTTACATGGTTTGCATGTTTTTATTGGTGTTTGTTTTCTT
ATTGTTAATTTTTTTCGTGTTAAGTTGTTCAATTTTAATTGATATCATGTTCAGGCTTATGATATAT
CTATTGATTATTGGCGTTTTTTAGAATGAATGTGAGGTGTTATGTTTTGTTTATTATATGTTTGAGG
TTCTTAATTGGTTATTTTTTATTATTTGGGGTTGTTGGTTATGATTGTTTTTATTTTGCAGGCTATT

GCTTTTTTAACTTTGTTGGAGCGCCATTTTTTGGGTGGTTCTCAGTGTCGTGTTGGTCCTAATAAGG
TGGGTTATTCTGGTGTTTTGCAGGCTTTATTTGATGGTTTAAAGTTGTTAAAGAAGGAGCAGTTGTT
GTTGTGTTTTTCTTCTTGATTATCTTTTTTGTTTATGCCTGTTTGTGGTTTTGTTTTGATGGTTTTT
TTTTGATTTACTTTGCCTTATTTTTTTTCTTTTTTGTCTTTTGAGTACTCTGGTGTTTTTTTGTTTT
GTCTTATAGGGGTTTCTGTTTATTTTATTATGCTTTCTGGTGTTTTTAGCGGTAGTAAATATTCTTT
TGTTGGTGGATTGCGTGCTTGCGTTCAGAGTTATTCTTATGAGATTGCTTTTTCTATTTATTTGTTG
GTTTTTTTGTTGTTTAATAAGGGTCTATGTTTGTCTTTTAGTTTTTGTTTATTTTTTTTTTTGTTTT
TTTTTCCTTTTTTTTGTTTAGTTCTTGTTGATTTGCATCGGGCTCCTTTTGATTTTTCTGAGTGTGA
AAGTGAGTTGGTAAGGGGGTTTAATGTTGAGTATTCGGGAGTTGGTTTTGCTGCTTTGTTTTTAGGG
GAGTATGGTAATTTACTTTATTTTGGTTGTTTGACTTCTAGTTTGTTTTTTGGTATAAGTTTTTTTT
TTTTTTATTTTATTGTATGTATGATTGTTTTTTCTCGTAGAGCTTATCCTCGTTTTCGTTTTGATAA
GTTGATGGGTGTTTGTTGGTTTTTGTTTTTGCCTGTCGGTTTTTATTTTTTTGGTTTGTCTTTTGTT
GTTTTTATGTTGTGCTTATTTAGTTTAATTTTTTAGATTTTGTTATTTTTTTTTTATTTGGTTTTGAT
TAGTTTTTTATTTGTTTTATATGGAGTTGAGTAAGTTTAGTAGTTTGGGAGTTTTAGGTGTTTTTGT
TAATGTTTTAGTTTCTGGATTTTCTCATCAGGGTTTTCAGTCTAGTGTTTTTTTTAAGTTTGTTGTT
TTTTTTCTTTTGGTTTTTTGAATGAGTGGGTTATTGTTTCCTTTGTTTAGTCCTTGGGCTTGTGTGG
GTTTTTTGTTTTTTGTTACTAATTTTTCTTGATTGGGTGTTCGTACTTTTATTTTAGCTGTAGATAG
TTTTTTGATTTTTTTTGAGGGGGATCACTCTTGGGAGTGGTTTTCTAGGTTAGTTATGTTTTTTTCT
CATTGGTTGAGATTTTTGATAAGGGGGGTGGCTTTAACTTTGCGTATTAGTATTATTTTTTTAATTG
GTCATTTTTTAATGTTTACTGTTTTGGATATGAGTGTATTTTATTCTTTGTTTTTTTTGTTGTTTGT
GGTTCCGGTGGAGTTGTTTTTTGCTTTTTTACAGAGTTATATTTTTTTGACTTTGGTTTGTATGTTT
TTACTTAATATGATTTAGATTTATTTGCAGAATTATGTGTTTCCTATTCCTGGAAATTCTTATGTGT
TTTGTTGTTATTATATCCATAATTATTATTCTCATATTATTTTTTTTGGTTTTTTTGTGATGTTTTT
GGTTAGTGGTGGGGTTTATTTTTTTGGCAATTCTTTTAAGTTTAACTTGAAGCGGAGAGATAGTCGT
ATAATTGAATTAGTTTTACAGGTGTTGATTGTTAATTTTTTGATTATGATGGCAGGTCCTGGTTTTT
GGTTGATTCAGTATCAGGGACGTATGTTTCGTCAATCTGAGTTGGCTTTGAAGGTTATTGGTCATCA
ATGGTATTGGAGTTATGAGTATGGTGATAGTGGAAAATTATGTTTTGATTCATTTATGAAGTCTTTA
GATGATTTGTCTTTAGGGGATTTTCGGTTATTTGATGTTGATAATCGGTGTGTTTTGCCTGTAGGTG
TGAATGTTAGAGTGTATTGTACTTCTAGTGATGTTATTCATTCTTTTGCTATTCCTAAGTGTTTTAT
TAAGATGGATGCTTTGAATGGTTTGTTAACTAAGGTTACTTGTAATTTTTCTTGTTCTGGTTTGTTT
TTTGGGCAGTGTTCTGAAATTTGTGGTGCTAATCATAGGTTTATGCCTATTGTGTTGGAGTTGACTT
CTTTGGAGTGTTGGAAGGGTTGATCAGTTAATTATTTGCTGGGTTAACTTTTTTTAAATTTTATTGT
TATTTTTTTTTTTTCTTTTTTGGTTCCTTTTGGTATGTATTTGTTGTCTTTTTTTGTGTCTTTTAAG
GATTTTTATGGTGCTAAATTAAGTTCTTATGAATGTGGTTTTGATGTTGTGAAGAAGGTTCATGTTG
GTTTTAATTTGGTTTTTTTTTCTATTGTTTTGTTGTTTGTTGTTTTTGAGTTGGAAGTTTTAATTTT
TATTATTTTGATTCAGGGTGATTTTTATAGTTTATTGTCTTTTTTTTGTTTTTTTTTTTATGTTGTT
TTTAGTTTTTATATGGAGTGGTATTTTGGTAAGTTGATTTGGTTTTGTTAGTTGTTATTGTTTGTTT
GATATGTTCTTATTTTTTATTTTTTTTTGGTTGTTGTTTTTTTGTTTGTTCCTTATGGTAAATGGAG
TTATAGTTTTGGTTTTAGTGATTATTTTAATTTTACTTTTGTTTATAATTTTGAAGTTTGTTTGTTT
TTTTTAGTTTTGTTGTTGGTTTCTTTTATGGTTTTTGTTTATGGTTCTTTTTATATGGTTGGGGTTT
CTCGTTTGTTTTATTTTTTTTTTTTTTTTATTTTTGTTTGTGTTGAGGATGGGTGGCTTGATTGTTTT
TAGAGGTAGTATTGTTTTAACTTTGGTTTTTTGGGATTTTTTGGGGGTTAGTAGTTTTTTTTTGGTT
TTGTTTTATGGTAATGTTAGTGCTCGAAGGGGTGCTATGAGTACTGTGTTTACTAATCGTATTGGTG
ATTTTTGTATTTTTTTGTTTTTTAATGGTTTTGTTTTGTTTTCTATGAGTTTTTTGTCTTATCAGTT
TTTTGGTTCTTTGTTAGTTTTTATGTTGTTTGTTTCTTCTGTTATTAAGGGTGGTCAGTATCCTTTT
GGTAGCTGGTTGCCTAAGGCTATGGCTGCTCCTACTCCTGTTAGCTGTTTGGTTCATAGTAGTACTT
TAGTTACTGCTGGTGTTATGTTGATGGATTGTTATGTTTATATTTCTTTGAATTCTGATGTTTTGTC
TTTTGTTTTTTATGTTGGTTTTTTTACTATAGTTTTTTCTGGTTTTTGTGCTTTGGTGGAGGAGGAT
GCTAAGAAGATTGTTGCTTTGAGTACTATGTCTCAGATTGGTTTTTGTTTTTTGGCTATTGGTAGGG
GTTTGCATTATTTGTCTTATGTTCATATGATTAGGCATTCTTTTTTTAAGAGATTGTTGTTTATGCA
GATAGGTTATTTGATTTTTATTAATTTTGGTCAACAGGATTATCGTGGTTATTCTTTTTTTGGTTTT
TGTGCTCCGGTTTTAGTTCAGTTGCAGATTTTTTTATCTGTGTTTTGTTTGTGTGGTTTGTTGTTTA

CTAGAGGTAGTTGTAGTAAGGAATATTTTATATCTCGTTTTTATTATGATTCTTATGGTTTTTTTTT
AGTTTTTTTTTATTTTTTTGGTGTGTTTTTGACTTTTTGTTATTGTTATCGGATGTTTTTTTTGTTT
CGTGTGGGGGCTTTTGGTTTTGATTATGTGGGTTTTTCTAGTAAGTTANTTTTATTTTTCTTGTTTT
TTTTTGGTTTTTTTTTCTGTTGTTTTTACTTTTTGGTGGGTTTTTGGTTTGTTATCTTTTTCTGCGG
CTTTTAATCGTTTTGAGTTTTTGGTTGTTTATTTTTATTTGTTTTTTGTTTATTGTTTTTGTGGTTA
TTTTTTTCGTTATTTTTTTTTCGTTATTTTGTGAAATTTTTTATGGATCATTATGCTTGTTTTATTT
ATAAAATTTTTCCTAGTTTTTTTTTATTTTGATGTTTTTATTATGGGTTTTAATTATTTTTTTTTTGG
GTTATTTCGGTTATTTTCTTTTTTTTTTTTTTCTTGGTTTCGGGGGTTTTATCATGTTGGTGTTTTA
ATTGTTTTTTTTTTTTATGTTGTTTTTTTTTGTTTTTTTAGATTTTGTTATTTTTTGTTTTTTTTTTAT
TATTGTTTTTGAGTTTTATTAATTTTTGTGTTGTTGACTATATTGTTTGGTGGAGGATTTTTGTTAT
TTGTACTTTTGTTTTTGTTTTTTTTTGTTGGTGGTGAGTTAGGTTTTGGGGGTTATTTGGTTAATTAT
TATGTTATTCAGGAAGTTTGTGGTTATTATTTTTTGGTTTTTGATGGTTGGAAGTTGCAATTTTTAT
TGCTTATGTTGAAGTCTGGTTCTTCTCCTTTTCATTTTTGACTTTTTAGTGTTTTGGGTGGTTTGGA
TAAGTGGTTTATTTTGTGGTTTTTAACTTTGCAAAAATTGCCTTATTTTGTTGTTTTGGTTAATTTT
TGTGGTGATTTTTTTTTTTTTGTTTTTGTTTTTTGGTATAATTTTTTGTTATTTTCAATTTTTTTTGT
TGCGTAGTTATCGTGATTTGTTAGTTGTGGGTTCTGCTGAATCTTTTAATTGGTTATTGTTATTGGG
TATTTTTTCTTTTAATGAAGTATTTGTTTTGTTTTTTTTTTTATTATTTTGTTATGTTTTTTGTTGTC
TCTTATGTGTATGGGGGATTTTTAAATTTTTTGAGTTTAGAGATATTGATGTTTTTTTTTAATGTTC
CTTTGAGAATTACTTTTTTTTTTAAAGGTGATTGTGTTGTTTGGTTCTTCTTTTTTTGTTGGTTTTTA
TTATTTATTTTTGTTGTTGTTTATGCCTTTGATGTCTTTGGGTATGGGTTATTTGTTTTTTTTTGGTT
TCGATGATGAGTTTTAATTATGGTTTTAAGTATTATGATTATTTTGTTTATGTTTTGTTTTGTGTTG
GGTTGTTGTCTTGTTTTTAGTTGTTGTATTTATTTTTTTTTTGTTGTTTTGTTGTTTTTTTTTAGTCC
TTTTTTATTTTTTGTTTTTTTTTATGTTTTTTGTTTTATATGGTTTTTTTGATTGTTCATGGTTTGGT
TGTTTGTTTTTTTTTTGATTCTTTTAATTTTGTTTTTTTGTCTTTTATGAGTGTTTTTGTTATGGGGT
TTATTTGTGTGTCTGAATTGTTGAGTGGTTTAGTTTTTTATAGTTGTCTTGTAGTGTTTTTTAGTGT
TTGTTTTTTTTATTCTGGTAGTTTTTTGATATTGTATGTTTTTTATGAGTTGACTATAGTACCTATT
TTGTTTTGTTTGTTAGGGTATGGTCGTCAGGTGGAGAAGGTTAGGGCTTGTTATTATTTAATTTTTT
ATACTTTGTTTTTTGGGATGCCTTATTTGTTTTTGTATAGTCATGTTTTTTTTTTTTTGAATTTTGT
TTATTATGATTTTTTTGTTTCTTATGAATTTATTTTTTTGTTGAGTTTGTGTTTTTTAGTTAAGTTT
CCTGTTTATTTTTTTCATGTTTGATTACCTAAGGTTCATGTTGAGGCTCCTACTAGTGCTAGAATGA
TTTTGGCTGGTGTTATGTTGAAGTTGGGAGGAGCAGGTGTTTATCGTATTAGTAAGTCTTTGAATTT
TTTTGGTTTTGAAATGTTGATTTTTTTTTCTTTGATTAGGATGGTTTTTTGTTCTTTTATTTGTGTT
GTTCAGAGTGATTGTAAGTCTTTGGCGGCTTATTCTTCTGTTTGTCATATGGGTTTTGTATTGCTCT
CTGAGATTAGTATGGTTTATTATGGTAAGTCTATGGCTTTGGTGATGATGTTGGCTCATGGTTATAC
TTCTGTTTTAATGTTTTATTTTATTGGTGAGTTTTATCATATTGCTAATAGGCGTTTAATTTATTAT
TTGCGTGGATATTTTAATGTTAGTATGTTGTTTTGTTTGATGTTTTGTTTGACTATGGTTTCTAATT
TTAGTTTTCCTGTATCTATTTCTTTTTTTTTCTGAGTATTTGATGTTGAATTTTTTTAGGTCTGTTTT
TTATGTTGGTTTTTTGTTTTTGTTTTTTTTATTATTTGGTTTCTTTTTATTATTCTGTTTATATTTTG
GTTTGTTTTTTAGTTGGGGATAAGGTGAGTTATGTTTGTGATGGTCGTAGTGTTGTTTGTTTACCTT
TGGTTTTTATGATATATAATTTTTTTTGGTTTATTTTTGTTATTTAATTTTAAGTTTGATTTTGGTT
TAGGTTGTATTAAGATAGTATTACTTATTTTTAGTTTATTTAATGTGTTATTTTTTGTACACTGGTA
GTTTTTTGATTGTTTTATTAACGTTCCAGAATAATCGGCTATGCGTTTTAATTTTTGACTCTATTTG
TTGTGGTGCTATGAGTTTTTAGTTTGTTTTTATGTTGTTTTTTGTAAAATATTTTGATTTTTTTTAG
TTTCTTGTGGTATCAAAAATTTGTTTTTTGAACTGGATTAGTACCCAGGTAATCAAAATTTAATAAT
TCGGGAGTAAAGTTTTGTTTAAACCGAAAAAATATTGACTGACTTTAGATTTTTCTTTGGAACATGT
GATTTGCTGGAGAGCCCTCTTTTTTTGGTGAATTTTGTTGGCACATGTATGATTGTTTAGTTTTTATT
TTATTTTGTAATGCTTTGTTGTTTTGGCATTAAAAACAGATATATATTTGGCTTATGAATTTATGTT
TCATGTGTTACTATTATGAATTTTTTTTGGATTAGTTTTTTATTTTTTTTTGAAATTGGAAAAGAAA
GTAATTTTTTTTTAATGTTTTAATGAATTTAATAAATAAGGTGGTACAAACCATCCGTCAATGGCCT
AAAGGGGCGTAAGTTGTAGTATGGTAGAAGTAAGGAAACTTGTTTCTATTTTTTGAAGTTTTTTTGT
TTTTTTAAGTTTTATTATTTGGTATTGCATATCAGTAGAAGTTTTTATCATAGTTATGAGTAATAGA
ATTAAAATGGTTAAATTGAATTTGTTTTTTTTTTTACGAAATTAATAATATTTTTATTTTAGTATTGA

TATAACGTATTTTTATTTCTGTTTATTTGTTATTTATAGGTTAAGTTATACTGTTTTTGTTTGTAGA
TTTTTAAATTTTTGTTGCTGTTTTATAAATGTTGTGTGTTTTACATAGATTTTTATTTTTTTTTTTT
TTTTTTTTTTGATTAATTTTTTAATTAATTTATGATTTTTACTTTAGTAATTTTTATTATTTTGAGG
GGTGTTTGTTTTTGAATTTATGTTTTTGAACTGGTTTTGTTGCTAAATGTTTATTAAAAACTTAGGT
TTTTATGTAAAATTGTCTTCTGCTCTATGAGTTTTTAAATGGCAGCCTTAGCGTGATGGCGTAAAAG
TAGCGTAAGTGATTTGTTTTTTTAATGGTTTCAAGTATGAATGAAGTTTTTAGCAGTTTTTTTATTT
ACTTTTTATTTGAATTATTTTTTTGATTAAAAATTATTAGTTAAGGTATTACAAAGATAAGTCTTCG
GAAATTTTGTTTTGAATTTTGAAATTTTTGTTTTTAATTTTTTCTTGGGGATGGATTTTAAGAAAGT
TTTATACTATTGTTATTATTAAAAATTACTCCGGAGTTAACAGGGTTGTAGACATATAAATAGATTT
TTATATTAGTGTGCTGCGCTACATCGATGTTGTATATTTTTTTTGATAATGGAGAGGTTTTTTTTAT
TTTGAGACTGTTCTTCTTGTATAAAAAATTGACTTGATATTAGTTTAGTTCGTCGTGAGACAGAGCG
GTTTATCTTGTGTATTTTTTGGTTTTTGGCGGTGTTAGTACGAAAGGAATGCAATGTGGGTTTATATT
TATGACTTTTTTATTTTGATGGGTTTTT

>O.ochengiF5
ATTTTTTGTGGAATGACTTTTGGTAATGGTATGAAGCAGAGTATTATTAATACTGTGAATCATAAGA
CTATTGGTACTTATTATATTGTTTTAGGTTATTGGGCTGGTTTAGGTGGTTCTGTTTTATCTATGTT
GATTCGTTTTGAATTGTCTAGTCCTGGTGGTTATTTGTTTTTTGGAAGTGGTCAGGTTTATAATTCT
GTTCTTACTATGCATGGTGTTTTGATGATTTTTTTTTTTAGTTATGCCTATTTTGATTGGTGGTTTTG
GTAATTGGATGTTGCCTTTGATGTTAGGGGCTCCTGAGATGGCGTTTCCTCGGGTAAATGCTTTGTC
TTTTTGATTTACTTTTGTGGCTTTGTTGATAGTTTATCAGTCTTTTTTTATCGGAGGTGGTCCTGGT
AGAAGTTGGACTTTTTATCCTCCTCTTAGGGTTGAAGGCCAACCAGAATTGTCTTTGGATACTATGA
TTTTAGGTTTACATACTGTAGGAATTGGTTCTTTGTTGGGTGCTATTAATTTTATGGTAACTACTCA
GAATATACGGTCCACTGCTGTGACTTTGGATCAAATTAGTATGTTTGTTTGGACTTCTTATTTGACT
TCTTTTTTATTAGTTTTGTCTGTGCCTGTTTTGGCTGGTTCTTTATTGTTTTTGTTGTTGGATCGTA
ATTTTAATACTTCTTTTTATGATACTAAGAAGGGGGGTAATCCTTTGTTGTATCAGCATTTGTTTTG
ATTTTTTGGTCATCCTGAGGTGTATGTTATTATTTTACCTGTTTTTGGTATTATTAGGGAAGCGGTT
TTATTTTTGACTGATAAGGATCGTTTGTTTGGTCAGACTAGGATGACTTTTGCTTCTATTTGAATTG
CTGTTTTAGGCACTTCTGTGTGAGGTCATCATATGTATACGGCTGGTTTGGATATTGATACTCGTAC
TTATTTTAGTGCTGCTACTATGATTATCGCTATTCCTAGAGCTGTTAAGATTTTTAATTGGTTAGGT
ACTTTTTTTGGTTCTAGTCAAAAGGTGCAGCCGTTATGATGTTGAACTTATAGTTTTATTTTTCTTT
TTACTGTGGGTGGATTAAGTGGAATTATTTTGAGAGCTGCTAGTTTGGATATTATTTTGCACGATAC
TTATTATGTTGTGGCTCATTTTCATTATACTTTGAGTTTGGGTGCTATTTATGGTATTTTTTGTGGT
TTTTGTTTGTGACTTCCTTATATGTATGGTATTTCTTTTGATAGGGTTATGATAATAGCTGTTTTTG
TTTGTTTTTTTTGTTGGTACTAATATGACATTTTTTCCTATGCATTTTGCTGGTTTGCAGGGTATGCC
TCGTAAGATTTTGGATTATCCTGATTGTTATTCTACTTTTCAGATTATTTCTTCTTTAGGTTCTGTT
ATTACTTTTGTTGGTTTTGTTTTGTTTAATTATTTGTTGGTTGATTCTATTTTTTTTTTCTCGTTTTT
TAGGGGTTTCTTTTTATAATTATCATAGTCCGGCTTATGCTTTAAATGTTCCTCCTTTGCCGGATTC
TTTTACTGAAGAGGCTTTTATTATAGGTCTTCATTGGAAGATTATTAGTAAGGACACTCCTTCTTAT
AGGTATCGTCGGGTTGGTTATGGTTATCATAGTAAGTAAATTTTTTTTTTATGTTAGGGTATTTTTTG
CTTTTGTTTTTTTTTTGTTTGAGTTTTTTGGATTGGGATCCGTTGAAGAGTTGTGTTATGATGTGTTT
GGGTGTTATGTCTATAAGTTGTTATGTTTCTTTGGGTATTCATGTATGGTATTCTTATTTTGTTGTT
TTAATTTTTTTTTAGTGGTATTTTTTTCTTTGTTGACTTATTTTTGTAGTATGAGTAATTTTGTTTTTT
ATTATAATTGTTTTTTTTTTTTTTTTNTTTGTTTTTGGTTAGTTTTTTTTTTTGTACTTGTTGTAGATTT
TGATTTTTTTTTTGTTTTTTTTGTGATTTTAATTTTCTTTATGTTTGTTATGATTTTAGTTATTATTAT
GTTTTTTGAGTAGTTTTTGTTTTGTTTTTGTTTTTGATTTTGGTTAGATTTAGGTTTAATGGTTTTG
GCTATATGCGTAGTTTGTAGATTGTTATTTTTAATTCTTTAGTTTTTTTGCCTGCTAGTTTTACTTT
GAGTTATATGTGAAATTTTGGTAGTATGTTGGGTATTATGTTGATGTCTCAGATTTTAACTGGTTTT
TTTTTGACTTTTTACTATACGGCTGGGGAGGCTTTTAGGTCTGTTCAGTATATTATGTTTGAGGTTA
ATTTGGGTTGGTTGTTGCGTATTATGCATTCTAATGGGGCTTCTATGTTTTTTTTGTTTATTTATTT
ACATATTTTTAAGGGTTTGATTTATGGTAGATATCGTCTTATTGGTGTATGATTGAGTGGTATTTTT
ATTTATTTTTTATTGATAGGTATTGCTTTTACTGGTTATGTTTTGATTTGAGGTCAAATAAGTTATT

GGGCGGCAGTAGTTATTACTAGTTTAATAACTTCTGTTCCTTATTTAGGTAAGTATTTAGTTTGATG
AATTTGGGGGAGTTTTAGTGTTTGTGAGAACACTTTGAAGTTTTTTTATTCTGTTCATTTTATTTTA
CCTTGGTCTTTAATAGTTTTAGTTATTTTTCATTTGTTTTTTTTGCATTTTACTGGTTCCAGTTCTA
GTTTGTATTGTCATGGAGATTATGATAAGATTCATTTTTTTCCTAGTTTTTGATTGAAGGATGGTTT
TGATATTTTTTTTTTATTTTTTTTTGATTTTGTTTAGGCTTTACTTTTCTTTTGATTTAAGTGATCCT
ATGATTTTTGTGGAGTCTGATTCTATGGCTAGTCCTGCGCATGTTGTACCTGAGTGATATTTTTTAT
TTGCTTTTACTATTTTACGTTCTGTTCCTAGTAAGTTATTGGGGGTTATTTTAATATTTAGTTCTGT
TTTTGTATTGACTATTCTTGTTTGACCTGATAGTTATCAGTCTATTTTGGATAATTTTTTATATTTT
TTTGTTATGTGTTTTGTTTGGGTTTTTTTTTGGTTAACTTGAGCTGGTCATTATCCTACTGATTATC
CTTTTAACTATTTTAATTTGTTTTGTACTTTTTTTTATTTTTGTTGTATTTTTTTTGTTTGTTTAAT
TAATTTTTTTAGTGATAAGTTGTTTAGTTAAATTTTGTTGAAGTTTCGTAAGTATCATAAAATGGAG
TATAGTTATTATCCTTTGATAGTTGGGGCGGGTATTTTAGGTTTTGATGTTAGTTTGGTTTTATTTA
TAGGTATAGGTATGTTTTATTCTATTTTTATTTGTTTTTTGTATTTGGTTTATGTTTTTTTTCTGTG
GATCAAGGATGTTATTTTAGAGGACATTAGTGGTCAATATTCTTTTTATGATTATCGTATGTTTAAT
CAAGGTTTTCGTTTGTTTCTTTTTAGTGAATTAACTTTGTTTGTTTCTGTTTTTTGGACTTTTTTGG
ATACAGCTTTGTGTCCTTTAACTTGGTTAGGTGGGGTTTGGTCTCCACTTGGGATTTTATCTCCTGA
TTATTTGGGTTTGAATGGTATGGCTAGTTTGTTTTTAATGATAAATAGGCAAGTTTTGAAGTATTCT
CGTCGTTATTTGTGTTTGAGTAGTTCTAAGTGTGAAGAATTTTTGTTAGTTTGTATTTTTATTGGAG
TTGGTTTTTTATGTTTTCAGTTTTATGAATATAATAACAATTCGTTTGTTATGAGTGATAGTGTTTA
TGGTAGTATTTTTTATATGGGTACTGGTTTACATGGTTTGCATGTTTTTGTTGGTGTTTGTTTTCTT
ATTGTTAATTTTTTTCGTGTTAAGTTGTTTAATTTTAATTGATATCATGTTCAGGCTTATGATATGT
CTATTGATTATTGGCGTTTTTTAGAGTGAATGTGAGGTGTTATGTTTTGTTTATTATATGTTTGAGG
TTCTTAATTGGTTATTTTTTATTATTTGGGGTTGTTGGTTATGATTGTTTTTATTTTGCAGGCTATT
GCTTTTTTGACTTTGTTGGAGCGTCATTTTTTGGGTGGTTCTCAGTGTCGTGTTGGTCCTAATAAGG
TAGGTTATTCTGGTGTTTTGCAGGCCTTATTTGATGGTTTAAAGTTGTTAAAGAAGGAGCAGTTGTT
GTTGTGTTTTTCTTCTTGATTATCTTTTTTGTTTATGCCTGTTTGTGGTTTTGTTTTGATGGTTTTT
TTTTGATTTACTTTGCCTTATTTTTTTTTCTTTTTTATCTTTTGAGTACTCTGGTGTTTTTTTGTTTT
GTCTTATAGGGGTTTCTGTTTATTTTATTATGCTTTCTGGTGTTTTTAGTGGTAGTAAGTATTCTTT
TGTTGGTGGATTGCGTGCTTGCGTTCAGAGTTATTCTTATGAGATTGCTTTTTCTATTTATTTGTTA
GTTTTTTTGCTGTTTAATAAGGGTCTATGTTTGTCTTTTAGTTTTTGTTTATTTTTTTTTTTTGTTTT
TTTTTCCTTTTTTCTGTTTAGTTCTTGTTGATTTGCATCGGGCTCCTTTTGATTTTTCTGAGTGTGA
AAGTGAGCTGGTAAGGGGGTTTAATGTTGAGTATTCGGGAGTTGGTTTTGCTGCTTTGTTTTTAGGG
GAGTATGGTAACTTACTTTATTTTGGTTGTTTGACTTCTAGTTTGTTTTTTGGTATAAGTTTTTTTT
TTTTTTATTTTATTGTATGTATGATTGTTTTTTCTCGTAGAGCTTATCCTCGTTTTCGTTTTGATAA
GTTGATGAGTGTTTGTTGGTTTTTGTTTTTGCCTGTTGGTTTTTATTTTTTTGGTTTGTCTTTTGTT
GTTTTTATGTTGTGCTTATTTAGTTTAATTTTTTAGATTTTGTTATTTTTTTTTTATTTGGTTTTGAT
TAGTTTTTTATTTGTTTTATATGGAGTTGAGCAAGTTTAGTAGTTTGGGAATTTTAGGTGTTTTTGT
TGATGTTTTAGTTTCTGGATTTTCTCATCAGGGTTTTCAGTCTAGTGTTTTTTTTAAGTTTGTTGTT
TTTTTTCTTTTGGTTTTTTGAATGAGGGGGTTATTGTTTCCTTTGTTTAGTCCTTGGGCTTGTGTAG
GTTTTTTGTTTTTTGTTACTAATTTTTCTTGATTGGGTGTTCGTACTTTTATTTTAGCTGTAGATAG
TTTTTTAATTTTTTTTGAGGGGGATCACTCTTGGGAATGATTTTCTAGGTTAGTTATGTTTTTTTCT
CATTGGTTGAGATTTTTGATAAGGGGGGTGGCTTTAACTTTGCGTATTAGTATTATTTTTTTAATTG
GTCATTTTTTAATGTTTACTGTTTTAGATATGAGTGTATTTTATTCTTTGTTTTTTTTGTTGCTTGT
GGTTCCGGTGGAGTTGTTTTTTGCTTTTTTACAGAGTTATATTTTTTTGACTTTGGTTTGTATGTTT
TTACTTAATATGATTTAGATTTATTTGCAGAATTATGTGTTTCCTATTCCTGGAAATTCTTATGTGT
TTTGTTGTTATTATATCCATAATTATTATTCTCATATTATTTTTTTTGGTTTTTTTGTAATGTTTTT
GGTTAGTGGTGGGGTTTATTTTTTTGGTAATTCTTTTAAGTTTAACTTGAAGCGTAGAGATAGTCGT
ATAATTGAATTAGTTTTACAGGTGTTGATTGTTAATTTTTTGATTATGATGGCAGGTCCTGGTTTTT
GGTTGATTCAGTATCAGGGACGTATGTTTCGTCAATCTGAGTTAGCTTTGAAGGTTATTGGTCATCA
ATGGTATTGGAGTTATGAGTATGGCGATAGTGGAAAATTATGTTTTGATTCATTTATGAAGTCTTTA
GATGATTTGTCTTTAGGGGATTTTCGGTTATTTGATGTTGATAATCGGTGTGTTTTGCCTGTAGGTG
TGAATGTTGGTGTGTATTGTACTTCTAGCGATGTTATTCATTCTTTTGCTATTCCTAAGTGTTTTAT

TAAGATGGATGCTTTGAATGGTTTGTTAACTAAGGTTACTTGTAATTTTTCTTGTTCTGGTTTGTTT
TTTGGGCAGTGTTCTGAAATTTGTGGTGCTAATCATAGGTTTATGCCTATTGTGTTGGAGTTGACTT
CTTTGGAGTGTTGGAAGGGTTGATCAGTTAATTATTTGCTGGGTTAACTTTTTTTAAATTTTGTTGT
TATTTTTTTTTTTTCTTTTTTGGTTCCTTTTGGTATGTATTTGTTGTCTTTTTTTGTGTCTTTTAAG
GATTTTTATGGTGCTAAATTGAGTTCTTATGAATGTGGTTTTGATGTTGTGAAGAAGGTTCATGTTG
GTTTTAATTTGGTTTTTTTTTCTATTGTTTTGTTGTTTGTTGTTTTTGAGTTGGAAGTTTTAATTTT
TATTATTTTGATTCAGGGTGATTTTTATAGTTTGTTGTCTTTTTTTTGTTTTTTTTTTTATGTTGTT
TTTAGTTTTTATATGGAGTGGTATTTTGGTAAGTTGATTTGGTTTTGTTAGTTGTTATTGTTTGTTT
GATATGTTCTTATTTTTTATTTTTTTTTGGTTGTTGTTTTTTTGTTTGTTCCTTATGGTAAATGGAG
TTATAGTTTTGGTTTTAGTGATTATTTTAATTTTACTTTTGTTTATAATTTTGAAGTTTGTTTGTTT
TTTTTAGTTTTGTTGTTGGTTTCTTTTATGGTTTTTGTTTATGGTTCTTTTTATATGGTTGGGGTTT
CTCGTTTGTTTTATTTTTTTTTTTTTTTTATTTTTGTTTGTGTTGAGGATGGGAGGTTTGATTGTTTT
TAGAGGTAGTATTGTTTTAACTTTGGTTTTTTGGGATTTTTTGGGGGTTAGTAGTTTTTTTTTGGTT
TTGTTTTATGGTAATGTTAGTGCTCGAAGGGGTGCTATGAGTACTGTATTTACTAATCGTATTGGTG
ATTTTTGTATTTTTTTTGTTTTTTAATGGTTTTGTTTTGTTTTCTATGAGTTTTTTGTCTTATCAGTT
TTTTGGTTCTTTGTTAGTTTTTATGTTGTTTGTCTCTTCTATTATTAAGGGTGGTCAGTATCCTTTT
GGTAGTTGGTTGCCTAAGGCTATGGCTGCTCCTACTCCTGTTAGTTGTTTAGTTCATAGTAGTACTT
TAGTTACTGCTGGTGTTATGTTGATGGATTGTTATGTTTATGTTTCTTTGAATTCTGATGTTTTGTC
TTTTGTTTTTTATGTTGGTTTTTTTACTATGGTTTTTTCTGGTTTTTGTGCTTTGGTAGAGGAGGAT
GCTAAGAAGATTGTTGCTTTGAGTACTATGTCTCAAATTGGTTTTTGTTTTTTGGCTATTGGTAGGG
GTTTGCATTATTTGTCTTATGTTCATATAATTAGGCATTCTTTTTTTAAGAGGTTGTTGTTTATACA
GATAGGTTATTTGATTTTTATTAATTTTGGTCAACAGGATTATCGTGGCTATTCTTTTTTTGGTTTT
TGTGCCCCGGTTTTAGTTCAGTTGCAGATTTTTTTATCTGTGTTTTGTTTGTGTGGTTTGTTGTTTA
CTAGAGGTAGTTGTAGTAAGGAATATTTTATATCTCGTTTTTATTATGATTCTTATGGTTTTTTTTT
AGTTTTTTTTTATTTTTTTGGTGTGTTTTTGACTTTTTGTTATTGTTATCGGATGTTTTTTTTGTTT
CGTGTGGGGGCTTCTGGTTTTGATTATGTGGGTTTTTCTAGTAAGTTGNTTTTATTTTTCTTGTTTT
TTTTTGGTTTTTTTTTCTGTTGTTTTTACTTTTTGGTGGGTTTTTGGTTTGTTGTCTTTTTCTGCGG
CTTTTAATCGTTTTGAGTTTTTGGTTGTTTATTTTTATTTGTTTTTTGTTTATTGTTTTTGTGGTTA
TTTTTTTCGTTATTTTTTTTTCGTTATTTTGTGAAATTTTTTATAGATCATTATGCTTGTTTTATTT
ATAAAATTTTTCCTAGTTTTTTTTTATTTTGATGTTTTTATTATGGGTTTTAATTATTTTTTTTTTGG
GTTATTTCGGTTATTTTCTTTTTTTTTTTTTTCTTGGTTTCGGGGGTTTTATCATGTTGGTGTTTTA
ATTGTTTTTTTTTTTATGTTGTTTTTTTTGTTTTTTTAGATTTTGTTATTTTTTGTTTTTTTTTTAT
TGTTGTTTTTGAGTTTTATTAATTTTTGTGTTGTTGATTATATTGTTTGATGGAGGATTTTTGTTAT
TTGTACTTTTGTTTTTGTTTTTTTTGTTGGTGGTGAGTTAGGTTTTGGGGGTTACTTGGTTAATTAT
TATGTTATTCAGGAAGTTTGTGGTTACTATTTTTTGGTTTTTGATGGTTGGAAGTTGCAATTTTTAT
TGCTTATGTTGAAGTCTGGTTCTTCTCCTTTTCATTTTTGACTTTTTAGTGTTTTGGGTGGTTTGGA
TAAGTGGTTTATTTTGTGGTTTTTAACTTTGCAAAAATTGCCTTATTTTGTTGTTTTGGTTAATTTT
TGTGGTGATTTTTTTTTTTTTGTTTTTGTTTTTTGGTATAATTTTTTGTTATTTTCAATTTTTTTTGT
TGCGTAGTTATCGTGATTTGTTAGTTGTGGGTTCTGCTGAATCTTTTAATTGGTTGTTGTTGTTGGG
TATTTTTTCTTTTAATGAAGTATTTGTTTTGTTTTTTTTTTTATTATTTTGTTATGTTTTTTGTTGTC
TCTTATGTGTATGGGGGATTTTTAAATTTTTTGAGTTTAGAGATATTGATGTTTTTTTTTTAATGTTC
CTTTGAGAATTACTTTTTTTTTTAAAGGTGATTGTGTTGTTTGGTTCTTCTTTTTTTTGTTGGTTTTTA
TTATTTATTTTTGTTGTTGTTTATGCCTTTGATATCTTTGGGTATGGGTTATTTGTTTTTTTTTGGTT
TCGATGATGAGTTTTAATTGTGGTTTTAAGTATTATGATTATTTTGTTTATGTTTTGTTTTGTGTTG
GGTTGTTGTCTTGTTTTTAGTTGTTGTATTTATTTTTTTTTTGTTGTTTTGTTGTTTTTTTTTTAGTCC
TTTTTTATTTTTTGTTTTTTTTTATGTTTTTTGTTTTATATGGTTTTTTTGATTGTTCATGGTTTGGT
TGTTTGTTTTTTTTTTGATTCTTTTAATTTTGTTTTTTTGTCTTTTATGAGTGTTTTTGTTATGGGGT
TTATTTGTGTGTCTGAATTGTTGAGTGGCTTAGTTTTTTATAGTTGTCTTGTAGTGTTTTTTAGTGT
TTGTTTTTTTTATTCTGGTAGTTTTTTGATATTGTATGTTTTTTTATGAGTTGACTATAGTACCTATT
TTGTTTTGTTTGTTAGGGTACGGTCGTCAGGTGGAGAAGATTAGGGCTTGTTATTATTTAATTTTTT
ATACTTTGTTTTTTGGGATGCCTTATTTGTTTTTGTATAGTCATGTTTTTTTTTTTTTTGAATTTTGT
TTATTATGATTTTTTTGTTTCTTATGAATTTATTTTTTTGTTGAGTTTGTGTTTTTTAGTTAAGTTT

CCTGTTTATTTTTTTCATGTTTGATTACCTAAGGTTCATGTTGAGGCTCCTACTAGTGCTAGAATGA
TTTTAGCTGGTGTTATGTTGAAGTTGGGAGGGGCAGGTGTTTATCGTATTAGTAAGTCTTTGAATTT
TTTTGGTTTTGAAATGTTGATTTTTTTTTTCTTTGATTAGGATGGTTTTTTGTTCTTTTATTTGTGTT
GTTCAGAGTGATTGTAAGTCTTTGGCGGCTTATTCTTCTGTTTGTCATATGGGTTTTGTGTTGCTTT
CTGAGATTAGTATGGTTTATTATGGTAAGTCTATGGCTTTGGTGATGATGTTGGCTCATGGTTATAC
TTCTGTTTTAATGTTTTATTTTATTGGTGAGTTTTACCATATTGCTAATAGGCGTTTAATTTATTAT
TTGCGTGGATATTTTAATGTTAGTATGTTGTTTTGTTTGATGTTTTGTTTGACTATGGTTTCTAATT
TTAGTTTTCCTGTATCTATTTCTTTTTTTTCTGAGTATTTGATGTTGAATTTTTTTAGTTCTGTTTT
TTATGTTGGTTTTTTGTTTTTGTTTTTTTATTATTTGGTTTCTTTTTATTATTCTGTTTATATTTTG
GTTTGTTTTTTAGTTGGGGATAAGGTGAGTTATGTTTGTGATGGTCGTAGTGTTGTTTGTTTACCTT
TGGTTTTTATGATATATAATTTTTTTTTGGTTTATTTTTGTTATTTAATTTTAAGTTTGATTTTGGTT
TAGGTTGTATTAAGATAGTATTACTTATTTTTAGTTTATTTAGTGTGTTATTTTTTGTACACTGGTA
GTTTTTTGATTGTTTTATTAACGTTCCAGAATAATCGGCTATGCGTTTTAATTTTTGACTCTATTTG
TTGTGGTGCTATGAGTTTTTATTTTGTTTTTATGTTGTTTTTTGTAAAATGTTTTAATTTTTTTTAG
TTTCTTGTGGTATCAAAAATTTGTTTTTTGAACTGGATTAGTACCCAGGTAATCAAAATTTAATAAT
TCGGGAGTAAAGTTTTGTTTAAACCGAAAAAATATTGACTGACTTTAGATTTTTCTTTGGAATATGT
GATTTGCTGGAGAGCCCTCTTTTTTGGTGAATTTTGTTGGCACATGTATGATTGTTTAGTTTTTATT
TTATTTTGTAATGCTTTGTTGTTTTGGCATTAAAAACAGATATATATTTGGCTTATGAATTTATGTT
TCATGTGTTACTATTATGAATTTTTTTTGGATTAGTTTTTTATTTTTTTTTGAAATTGGAAAAGAAA
GTAATTTTTTCTTAATGTTTTAATGAATTTAATAAATAAGGTGGTACAAACCATCCGTCAATGGCCT
AAAGGGGCGTAAGTTGTAGTATGGTAGAAGTAAGGAAACTTGTTTCTATTTTTTGAAGTTTTTTTGT
TTTTTTAAGTTTTATTATTTGGTATTGCATATCAGTAGAAGTTTTTATCATAGTTATGAGTAATAGA
ATAAAAATGGTTAAATTGAATTTGTTTTTTTTTTACGAAATTAATAATATTTTTATTTTAGTATTGA
TATAACGTATTTTTATTTCTGTTTATTTGTTATTTATAGGTTAAGTTATACTGTTTTTGTTTGTAGA
TTTTTAAATTTTTGTTGCTGTCTTGTAAATGTTGTGTGTTTTACATAGATTTTTATTTTTTTTTTTT
TTTTTTTTTGATTAATTTTTTAATTAATTTATGATTTTTACTTTAGTAATTTTTGTTATTTTGAGG
GGTGTTTGTTTTTGAATTTATGTTTTTGAACTGGTTTTGTTGCTAAATGTTTATTAAAAACTTAGGT
TTTTATGTAAAATTGTCTTCTGCTCTATGAGTTTTTAAATGGCAGCCTTAGCGTGATGGCGTAAAAG
TAGCGTAAGTGATTTGTTTTTTTAATGGTTTCAAGTATGAATGAAGTTTTTAGCAGCTTTTTTATTT
ACTTTTTATTTGAATTATTTTTTTGATTAAAAATTATTAGTTAAGGTATTACAAAGATAAGTCTTCG
GAAATTTTGTTTTGAATTTTGAAATTTTTATTTTTAATTTTTTCTTGGGGATGGATTTTAAGAAAGT
TTTATACTATTATTATTATTAAAAATTACTCCGGAGTTAACAGGGTTGTAGACATATAAATAGGTTT
TTATATTAGTGTGCTGCGCTACATCGATGTTGTATATTTTTTTTGATAATGGAGAGGTTTTTTTTAT
TTTGAGACTGTTCTTCTTGTATAAAAAATTGACTTGATATTAGTTTAGTTCGTCGTGAGACAGAGCG
GTTTATCTTGTGTATTTTTGGTGTTCGGCGGTGTTAGTACGAAAGGAATGCAATGTGGGTTTATATT
TATGACTTTTTTATTTTGATGGATTTTT

>O.ochengiM1(KX181290)
ATTTTTTGTGGAATGACTTTTGGTAATGGTATGAAGCAGAGTATTATTAATACTGTGAATCATAAGA
CTATTGGTACTTATTATATTGTTTTAGGTTATTGGGCTGGTTTAGGTGGTTCTGTTTTATCTATGTT
GATTCGTTTTGAATTGTCTAGTCCTGGTGGTTATTTGTTTTTTGGAAGTGGTCAGGTTTATAATTCT
GTTCTTACTATGCATGGTGTTTTGATGATTTTTTTTTTTAGTTATGCCTATTTTGATTGGTGGTTTTG
GTAATTGGATGTTGCCTTTGATGTTAGGGGCTCCTGAGATGGCGTTTCCTCGGGTAAATGCTTTGTC
TTTTTGATTTACTTTTGTGGCTTTGTTGATAGTTTATCAGTCTTTTTTTATCGGAGGTGGCCCTGGT
AGAAGTTGGACTTTTTATCCTCCTCTTAGGGTTGAAGGTCAACCAGAATTGTCTTTGGATACTATGA
TTTTAGGTTTACATACTGTAGGAATTGGTTCTTTGTTGGGTGCTATTAATTTTATGGTAACTACTCA
GAATATACGGTCCACTGCTGTGACTTTGGACCAAATTAGTATGTTTGTTTGGACTTCTTATTTGACT
TCTTTTTTATTAGTTTTGTCTGTGCCTGTTTTGGCTGGTTCTTTATTGTTTTTGTTGTTGGATCGTA
ATTTTAATACTTCTTTTTATGATACTAAGAAGGGGGGTAATCCTTTGTTGTATCAGCATTTGTTTTG
ATTTTTTGGTCATCCTGAGGTGTATGTTATTATTTTACCTGTTTTTGGTATTATTAGGGAAGCGGTT
TTATTTTTGACTGATAAGGATCGTTTGTTTGGTCAGACTAGGATGACTTTTGCTTCTATTTGAATTG
CTGTTTTAGGCACTTCTGTGTGAGGTCATCATATGTATACGGCTGGTTTGGATATTGATACTCGTAC

TTATTTTAGTGCTGCTACTATGATTATCGCTATTCCTAGAGCTGTTAAGATTTTTAATTGGTTAGGT
ACTTTTTTTGGTTCTAGTCAAAAGGTGCAGCCGTTATGATGTTGAACTTATAGTTTTATTTTTCTTT
TTACTGTGGGTGGATTAAGTGGAATTATTTTGAGAGCTGCTAGTTTGGATATTATTTTGCACGATAC
TTATTATGTTGTGGCTCATTTTCATTATACTTTGAGTTTGGGTGCTATTTATGGTATTTTTTGTGGT
TTTTGTTTGTGACTTCCTTATATGTATGGTATTTCTTTTGATAGGGTTATGATAATAGCTGTTTTTG
TTTGTTTTTTTGTTGGTACTAATATGACATTTTTTCCTATGCATTTTGCTGGTTTGCAGGGTATGCC
TCGTAAGATTTTGGATTATCCTGATTGTTATTCTACTTTTCAGATTATTTCTTCTTTAGGTTCTGTT
ATTACTTTTGTTGGTTTTGTTTTGTTTAATTATTTGTTGGTTGATTCTATTTTTTTTTCTCGTTTTT
TAGGGGTTTCTTTTTATAATTATCATAGTCCGGCTTATGCTTTAAATGTTCCTCCTTTGCCGGATTC
TTTTACTGAAGAGGCTTTTATTATAGGTCTTCATTGGAAGATTATTAGTAAGGATACTCCTTCTTAT
AGGTATCGTCGGGTTGGTTATGGTTATCATAGTAAGTAAATTTTTTTTTATGTTAGGGTATTTTTTG
CTTTTGTTTTTTTTTGTTTGAGTTTTTTGGATTGGGATCCGTTGAAGAGTTGTGTTATGATGTGTTT
GGGTGTTATGTCTATAAGTTGTTATGTTTCTTTGGGTATTCATGTATGGTATTCTTATTTTGTTGTT
TTAATTTTTTTTAGTGGTATTTTTTCTTTGTTGACTTATTTTTGTAGTATGAGTAATTTTGTTTTTT
ATTATAATTGTTTTTTTTTTTTTTTCTTTGTTTTTGGTTAGTTTTTTTTTTTGTACTTGTTGTAGATTT
TGATTTTTTTTTGTTTTTTTGTGATTTTAATTTTCTTTATGTTTGTTATGATTTTAGTTATTATTAT
GTTTTTTGAGTAGTTTTTGTTTTGTTTTTGTTTTTGATTTTGGTTAGATTTAGGTTTAATGGTTTTG
GCTATATGCGTAGTTTGTAGATTGTTATTTTTAATTCTTTAGTTTTTTTGCCTGCTAGTTTTACTTT
GAGTTATATGTGAAATTTTGGTAGTATGTTGGGTATTATGTTGATGTCTCAGATTTTAACTGGTTTT
TTTTTGACTTTTTACTATACGGCTGGGGAGGCTTTTAGGTCTGTTCAGTATATTATGTTTGAGGTTA
ATTTGGGTTGGTTGTTGCGTATTATGCATTCTAATGGGGCTTCTATGTTTTTTTTTGTTTATTTATTT
ACATATTTTTAAGGGTTTGATTTATGGTAGATATCGTCTTATTGGTGTATGATTGAGTGGTATTTTT
ATTTATTTTTTATTGATAGGTGTTGCTTTTACTGGTTATGTTTTGATTTGAGGTCAAATAAGTTATT
GGGCGGCAGTAGTTATTACTAGTTTAATAACTTCTGTTCCTTATTTAGGTAAGTATTTAGTTTGATG
AATTTGGGGGAGTTTTAGTGTTTGTGAGAACACTTTGAAGTTTTTTTATTCTGTTCATTTTATTTTA
CCTTGGTCTTTAATAGTTTTAGTTATTTTTCATTTGTTTTTTTTGCATTTTACTGGTTCCAGTTCTA
GTTTGTATTGTCATGGAGATTATGATAAGATTCATTTTTTTCCTAGTTTTTGATTGAAGGATGGTTT
TGATATTTTTTTTTATTTTTTTTTGATTTTGTTTAGGCTTTATTTTTCTTTTGATTTAAGTGATCCT
ATGATTTTTGTGGAGTCTGATTCTATGGCTAGTCCTGCGCATGTTGTACCTGAGTGATATTTTTTAT
TTGCTTTTACTATTTTACGTTCTGTTCCTAGTAAGTTATTGGGGGTTATTTTAATATTTAGTTCTGT
TTTTGTATTGACTATTCTTGTTTGACCTGATAGTTATCAGTCTATTTTGGATAATTTTTTATATTTT
TTTGTTATGTGTTTTGTTTGGGTTTTTTTTTGGTTAACTTGAGCTGGTCATTATCCTACTGATTATC
CTTTTAACTATTTTAATTTGTTTTGTACTTTTTTTTATTTTTGTTGTATTTTTTTTGTTTGTTTAAT
TAATTTTTTTAGTGATAAGTTGTTTAGTTAAATTTTGTTGAAGTTTCGTAAGTATCATAAAATGGAG
TATAGTTATTATCCTTTGATAGTTGGGGCGGGTATTTTAGGTTTTGATGTTAGTTTGGTTTTATTTA
TAGGTATAGGTATGTTTTATTCTATTTTTATTTGTTTTTTGTATTTGGTTTATGTTTTTTTTCTGTG
GATCAAGGATGTTATTTTAGAGGACATTAGTGGTCAATATTCTTTTTATGATTATCGTATGTTTAAT
CAAGGTTTTCGTTTGTTTCTTTTTAGTGAATTAACTTTGTTTGTTTCTGTTTTTTGGACTTTTTTGG
ATACAGCTTTGTGTCCTTTAACTTGGTTAGGTGGGGTTTGGTCTCCACTTGGGATTTTATCTCCTGA
TTATTTGGGTTTGAATGGTATGGCTAGTTTGTTTTTAATGATAAATAGGCAAGTTTTGAAGTATTCT
CGTCGTTATTTGTGTTTGAGTAGTTCTAAGTGTGAAGAATTTTTGTTAGTTTGTATTTTTATTGGAG
TTGGTTTTTTATGTTTTCAGTTTTATGAATATAATAACAATTCGTTTGTTATGAGTGATAGTGTTTA
TGGTAGTATTTTTTTATATGGGTACTGGTTTACATGGTTTGCATGTTTTTGTTGGTGTTTGTTTTCTT
ATTGTTAATTTTTTTCGTGTTAAGTTGTTTAATTTTAATTGATATCATGTTCAGGCTTATGATATAT
CTATTGATTATTGGCGTTTTTTAGAGTGAATGTGAGGTGTTATGTTTTGTTTATTATATGTTTGAGG
TTCTTAATTGGTTATTTTTTATTATTTGGGGTTGTTGGTTATGATTGTTTTTATTTTGCAGGCTATT
GCTTTTTTGACTTTGTTGGAGCGTCATTTTTTGGGTGGTTCTCAGTGTCGTGTTGGTCCTAATAAGG
TAGGTTATTCTGGTGTTTTGCAGGCCTTATTTGATGGTTTAAAGTTGTTAAAGAAGGAGCAGTTGTT
GTTGTGTTTTTCTTCTTGATTATCTTTTTTGTTTATGCCTGTTTGTGGTTTTGTTTTGATGGTTTTT
TTTTGATTTACTTTGCCTTATTTTTTTTCTTTTTTATCTTTTGAGTACTCTGGTGTTTTTTTGTTTT
GTCTTATAGGGGTTTCTGTTTATTTTATTATGCTTTCTGGTGTTTTTAGTGGTAGTAAGTATTCTTT
TGTTGGTGGATTGCGTGCTTGCGTTCAGAGTTATTCTTATGAGATTGCTTTTTCTATTTATTTGTTA

GTTTTTTTGCTGTTTAATAAGGGTCTATGTTTGTCTTTTAGTTTTTGTTTATTTTTTTTTTTTGTTTT
TTTTTCCTTTTTTTTGTTTAGTTCTTGTTGATTTGCATCGGGCTCCTTTTGATTTTTCTGAGTGTGA
AAGTGAGCTGGTAAGGGGGTTTAATGTTGAGTATTCGGGAGTTGGTTTTGCTGCTTTGTTTTTAGGG
GAGTATGGTAACTTACTTTATTTTGGTTGTTTGACTTCTAGTTTGTTTTTTGGTATAAGTTTTTTTT
TTTTTTATTTTATTGTATGTATGATTGTTTTTTCTCGTAGAGCTTATCCTCGTTTTCGTTTTGATAA
GTTGATGAGTGTTTGTTGGTTTTTGTTTTTGCCTGTTGGTTTTTATTTTTTTGGTTTGTCTTTTGTT
GTTTTTATATTGTGCTTATTTAGTTTAATTTTTTAGATTTTGTTATTTTTTTTTTATTTGGTTTTGAT
TAGTTTTTTATTTGTTTTATATGGAGTTGAGCAAGTTTAGTAGTTTGGGAATTTTAGGTGTTTTTGT
TGATGTTTTAGTTTCTGGATTTTCTCATCAGGGTTTTCAGTCTAGTGTTTTTTTTAAGTTTGTTGTT
TTTTTTCTTTTGGTTTTTTGAATGAGGGGGTTATTGTTTCCTTTGTTTAGTCCTTGGGCTTGTGTAG
GTTTTTTGTTTTTTGTTACTAATTTTTCTTGATTGGGTGTTCGTACTTTTATTTTAGCTGTAGATAG
TTTTTTAATTTTTTTTGAGGGGGATCACTCTTGGGAATGATTTTCTAGGTTAGTTATGTTTTTTTCT
CATTGGTTGAGATTTTTGATAAGGGGGGTGGCTTTAACTTTGCGTATTAGTATTATTTTTTTAATTG
GTCATTTTTTAATGTTTACTGTTTTAGATATGAGTGTATTTTATTCTTTGTTTTTTTTGTTGCTTGT
GGTTCCGGTGGAGTTGTTTTTTGCTTTTTTACAGAGTTATATTTTTTTGACTTTGGTTTGTATGTTT
TTACTTAATATGATTTAGATTTATTTGCAGAATTATGTGTTTCCTATTCCTGGAAATTCTTATGTGT
TTTGTTGTTATTATATCCATAATTATTATTCTCATATTATTTTTTTTGGTTTTTTTGTAATGTTTTT
GGTTAGTGGTGGGGTTTATTTTTTTGGTAATTCTTTTAAGTTTAACTTGAAGCGTAGAGATAGTCGT
ATAATTGAATTAGTTTTACAGGTGTTGATTGTTAATTTTTTGATTATGATGGCAGGTCCTGGTTTTT
GGTTGATTCAGTATCAGGGACGTATGTTTCGTCAATCTGAGTTAGCTTTGAAGGTTATTGGTCATCA
ATGGTATTGGAGTTATGAGTATGGCGATAGTGGAAAATTATGTTTTGATTCATTTATGAAGTCTTTA
GATGATTTGTCTTTAGGGGATTTTCGGTTATTTGATGTTGATAATCGGTGTGTTTTGCCTGTAGGTG
TGAATGTTGGTGTGTATTGTACTTCTAGCGATGTTATTCATTCTTTTGCTATTCCTAAGTGTTTTAT
TAAGATGGATGCTTTGAATGGTTTGTTAACTAAGGTTACTTGTAATTTTTCTTGTTCTGGTTTGTTT
TTTGGGCAGTGTTCTGAAATTTGTGGTGCTAATCATAGGTTTATGCCTATTGTGTTGGAGTTGACTT
CTTTGGAGTGTTGGAAGGGTTGATCAGTTAATTATTTGCTGGGTTAACTTTTTTTAAATTTTGTTGT
TATTTTTTTTTTTTTCTTTTTTGGTTCCTTTTGGTATGTATTTGTTGTCTTTTTTTTGTGTCTTTTAAG
GATTTTTATGGTGCTAAATTGAGTTCTTATGAATGTGGTTTTGATGTTGTGAAGAAGGTTCATGTTG
GTTTTAATTTGGTTTTTTTTTTCTATTGTTTTGTTGTTTGTTGTTTTTGAGTTGGAAGTTTTAATTTT
TATTATTTTGATTCAGGGTGATTTTTATAGTTTGTTGTCTTTTTTTTTGTTTTTTTTTTTATGTTGTT
TTTAGTTTTTATATGGAGTGGTATTTTGGTAAGTTGATTTGGTTTTGTTAGTTGTTATTGTTTGTTT
GATATGTTCTTATTTTTTATTTTTTTTTGGTTGTTGTTTTTTTGTTTGTTCCTTATGGTAAATGGAG
TTATAGTTTTGGTTTTAGTGATTATTTTAATTTTACTTTTGTTTATAATTTTGAAGTTTGTTTGTTT
TTTTTAGTTTTGTTGTTGGTTTCTTTTATGGTTTTTGTTTATGGTTCTTTTTATATGGTTGGGGTTT
CTCGTTTGTTTTATTTTTTTTTTTTTTTTATTTTTGTTTGTGTTGAGGATGGGAGGTTTGATTGTTTT
TAGAGGTAGTATTGTTTTAACTTTGGTTTTTTGGGATTTTTTGGGGGTTAGTAGTTTTTTTTTGGTT
TTGTTTTATGGTAATGTTAGTGCTCGAAGGGGTGCTATGAGTACTGTATTTACTAATCGTATTGGTG
ATTTTTGTATTTTTTTTGTTTTTTAATGGTTTTGTTTTGTTTTCTATGAGTTTTTTGTCTTATCAGTT
TTTTGGTTCTTTGTTAGTTTTTATGTTGTTTGTCTCTTCTATTATTAAGGGTGGTCAGTATCCTTTT
GGTAGTTGGTTGCCTAAGGCTATGGCTGCTCCTACTCCTGTTAGTTGTTTAGTTCATAGTAGTACTT
TAGTTACTGCTGGTGTTATGTTGATGGATTGTTATGTTTATGTTTCTTTGAATTCTGATGTTTTGTC
TTTTGTTTTTTATGTTGGTTTTTTTACTATGGTTTTTTCTGGTTTTTGTGCTTTGGTAGAGGAGGAT
GCTAAGAAGATTGTTGCTTTGAGTACTATGTCTCAAATTGGTTTTTGTTTTTTGGCTATTGGTAGGG
GTTTGCATTATTTGTCTTATGTTCATATAATTAGGCATTCTTTTTTTAAGAGGTTGTTGTTTATACA
GATAGGTTATTTGATTTTTATTAATTTTGGTCAACAGGATTATCGTGGCTATTCTTTTTTTGGTTTT
TGTGCCCCGGTTTTAGTTCAGTTGCAGATTTTTTTATCTGTGTTTTGTTTGTGTGGTTTGTTGTTTA
CTAGAGGTAGTTGTAGTAAGGAATATTTTATATCTCGTTTTTATTATGATTCTTATGGTTTTTTTTT
AGTTTTTTTTTATTTTTTTGGTGTGTTTTTGACTTTTTGTTATTGTTATCGGATGTTTTTTTTGTTT
CGTGTGGGGGCTTCTGGTTTTGATTATGTGGGTTTTTCTAGTAAGTTGNTTTTATTTTTCTTGTTTT
TTTTTGGTTTTTTTTTCTGTTGTTTTTACTTTTTGGTGGGTTTTTGGTTTGTTGTCTTTTTCTGCGG
CTTTTAATCGTTTTGAGTTTTTGGTTGTTTATTTTTATTTGTTTTTTGTTTATTGTTTTTGTGGTTA
TTTTTTTCGTTATTTTTTTTTCGTTATTTTGTGAAATTTTTTATAGATCATTATGCTTGTTTTATTT

ATAAAATTTTTCCTAGTTTTTTTTATTTTGATGTTTTTATTATGGGTTTTAATTATTTTTTTTTTTGG
GTTATTTCGGTTATTTTCTTTTTTTTTTTTTTTCTTGGTTTCGGGGGTTTTATCATGTTGGTGTTTTA
ATTGTTTTTTTTTTTTATGTTGTTTTTTTTTGTTTTTTTAGATTTTGTTATTTTTTGTTTTTTTTTTTAT
TGTTGTTTTTGAGTTTTATTAATTTTTGTGTTGTTGATTATATTGTTTGATGGAGGATTTTTGTTAT
TTGTACTTTTGTTTTTGTTTTTTTTTGTTGGTGGTGAGTTAGGTTTTGGGGGTTACTTGGTTAATTAT
TATGTTATTCAGGAAGTTTGTGGTTACTATTTTTTGGTTTTTGATGGTTGGAAGTTGCAATTTTTAT
TGCTTATGTTGAAGTCTGGTTCTTCTCCTTTTCATTTTTGACTTTTTAGTGTTTTGGGTGGTTTGGA
TAAGTGGTTTATTTTGTGGTTTTTAACTTTGCAAAAATTGCCTTATTTTGTTGTTTTGGTTAATTTT
TGTGGTGATTTTTTTTTTTTTGTTTTTGTTTTTTGGTATAATTTTTTGTTATTTTCAATTTTTTTTGT
TGCGTAGTTATCGTGATTTGTTAGTTGTGGGTTCTGCTGAATCTTTTAATTGGTTGTTGTTGTTGGG
TATTTTTTCTTTTAATGAAGTATTTGTTTTGTTTTTTTTTTATTATTTTGTTATGTTTTTTGTTGTC
TCTTATGTGTATGGGGGATTTTTAAATTTTTTGAGTTTAGAGATATTGATGTTTTTTTTTAATGTTC
CTTTGAGAATTACTTTTTTTTTAAAGGTGATTGTGTTGTTTGGTTCTTCTTTTTTTGTTGGTTTTTA
TTATTTATTTTTGTTGTTGTTTATGCCTTTGATGTCTTTGGGTATGGGTTATTTGTTTTTTTTTGGTT
TCGATGATGAGTTTTAATTGTGGTTTTAAGTATTATGATTATTTTGTTTATGTTTTGTTTTGTGTTG
GGTTGTTGTCTTGTTTTTAGTTGTTGTATTTATTTTTTTTTGTTGTTTTGTTGTTTTTTTTTAGTCC
TTTTTTATTTTTTGTTTTTTTTATGTTTTTTGTTTTATATGGTTTTTTTGATTGTTCATGGTTTGGT
TGTTTGTTTTTTTTTGATTCTTTTAATTTTGTTTTTTTGTCTTTTATGAGTGTTTTTGTTATGGGGT
TTATTTGTGTGTCTGAATTGTTGAGTGGCTTAGTTTTTTATAGTTGTCTTGTAGTGTTTTTTAGTGT
TTGTTTTTTTTATTCTGGTAGTTTTTTGATATTGTATGTTTTTTATGAGTTGACTATAGTACCTATT
TTGTTTTGTTTGTTAGGGTACGGTCGTCAGGTGGAGAAGATTAGGGCTTGTTATTATTTAATTTTTT
ATACTTTGTTTTTTGGGATGCCTTATTTGTTTTTGTATAGTCATGTTTTTTTTTTTTTGAATTTTGT
TTATTATGATTTTTTTGTTTCTTATGAATTTATTTTTTTATTGAGTTTGTGTTTTTTAGTTAAGTTT
CCTATTTATTTTTTTCATGTTTGATTACCTAAGGTTCATGTTGAGGCTCCTACTAGTGCTAGAATGA
TTTTGGCTGGTGTTATGTTGAAGTTGGGAGGGGCAGGTGTTTATCGTATTAGTAAGTCTTTGAATTT
TTTTGGTTTTGAAATGTTGATTTTTTTTTCTTTGATTAGGATGGTTTTTTGTTCTTTTATTTGTGTT
GTTCAGAGTGATTGTAAGTCTTTGGCGGCTTATTCTTCTGTTTGTCATATGGGTTTTGTGTTGCTTT
CTGAGATTAGTATGGTCTATTATGGTAAGTCTATGGCTTTGGTGATGATGTTGGCTCATGGTTATAC
TTCTGTTTTAATGTTTTATTTTATTGGTGAGTTTTACCATATTGCTAATAGGCGTTTAATTTATTAT
TTGCGTGGATATTTTAATGTTAGTATGTTGTTTTGTTTGATGTTTTGTTTGACTATGGTTTCTAATT
TTAGTTTTCCTGTATCTATTTCTTTTTTTTTCTGAGTATTTGATGTTGAATTTTTTTAGTTCTGTTTT
TTATGTTGGTTTTTTGTTTTTGTTTTTTTATTATTTGGTTTCTTTTTATTATTCTGTTTATATTTTG
GTTTGTTTTTTAGTTGGGGATAAGGTGAGTTATGTTTGTGATGGTCGTAGTGTTGTTTGTTTACCTT
TGGTTTTTATGATATATAATTTTTTTTGGTTTATTTTTGTTATTTAATTTTAAGTTTGATTTTGGTT
TAGGTTGTATTAAGATAGTATTACTTATTTTTAGTTTATTTAGTGTGTTATTTTTTGTACACTGGTA
GTTTTTTGATTGTTTTATTAACGTTCCAGAATAATCGGCTATGCGTTTTAATTTTTGACTCTATTTG
TTGTGGTGCTATGAGTTTTTATTTTGTTTTTATGTTGTTTTTTGTAAAATGTTTTGATTTTTTTTAG
TTTCTTGTGGTATCAAAAATTTGTTTTTTGAACTGGATTAGTACCCAGGTAATCAAAATTTAATAAT
TCGGGAGTAAAGTTTTGTTTAAACCGAAAAAATATTGACTGACTTTAGATTTTTCTTTGGAATATGT
GATTTGCTGGAGAGCCCTCTTTTTTGGTGAATTTTGTTGGCACATGTATGATTGTTTAGTTTTTATT
TTATTTTGTAATGCTTTGTTGTTTTGGCATTAAAAACAGATATATATTTGGCTTATGAATTTATGTT
TCATGTGTTACTATTATGAATTTTTTTTGGATTAGTTTTTTATTTTTTTTTGAAATTGGAAAAGAAA
GTAATTTTTTCTTAATGTTTTAATGAATTTAATAAATAAAGTGGTACAAACCATCCGTCAATGGCCT
AAAGGGGCGTAAGTTGTAGTATGGTAGAAGTAAGGAAACTTGTTTCTATTTTTTGAAGTTTTTTTGT
TTTTTTAAGTTTTATTATTTGGTATTGCATATCAGTAGAAGTTTTTATCATAGTTATGAGTAATAGA
ATTAAAATGGTTAAATTGAATTTGTTTTTTTTTTACGAAATTAATAATATTTTTATTTTAGTATTGA
TATAACGTATTTTTATTTCTGTTTATTTGTTATTTATAGGTTAAGTTATACTGTTTTTGTTTGTAGA
TTTTTAAATTTTTGTTGCTGTCTTGTAAATGTTGTGTGTTTTACATAGATTTTTATTTTTTTTTTTT
TTTTTTTTTTGATTAATTTTTTAATTAATTTATGATTTTTACTTTAGTAATTTTTGTTATTTTGAGG
GGTGTTTGTTTTTGAATTTATGTTTTTGAACTGGTTTTGTTGCTAAATGTTTATTAAAAACTTAGGT
TTTTATGTAAAATTGTCTTCTGCTCTATGAGTTTTTAAATGGCAGCCTTAGCGTGATGGCGTAAAAG
TAGCGTAAGTGATTTGTTTTTTTAATGGTTTCAAGTATGAATGAAGTTTTTAGCAGCTTTTTTATTT

ACTTTTTATTTGAATTATTTTTTTGATTAAAAATTATTAGTTAAGGTATTACAAAGATAAGTCTTCG
GAAATTTTGTTTTGAATTTTGAAATTTTTGTTTTTAATTTTTTCTTGGGGATGGATTTTAAGAAAGT
TTTATACTATTATTATTATTAAAAATTACTCCGGAGTTAACAGGGTTGTAGACATATAAATAGGTTT
TTATATTAGTGTGCTGCGCTACATCGATGTTGTATATTTTTTTTGATAATGGAGAGGTTTTTTTTAT
TTTGAGACTGTTCTTCTTGTATAAAAAATTGACTTGATATTAGTTTAGTTCGTCGTGAGACAGAGCG
GTTTATCTTGTGTATTTTTGGTGTTCGGCGGTGTTAGTACGAAAGGAATGCAATGTGGGTTTATATT
TATGACTTTTTTATTTTGATGGATTTTT
